# Drug-associated insomnia and sex-specific disproportionality in the FDA adverse event reporting system (2019–Q1 2025)

**DOI:** 10.3389/fphar.2026.1758403

**Published:** 2026-02-23

**Authors:** Hao Wen, Yuchuan Shen, Hai Li, Xiangbin Chen, Yanzhao Lin, Wei Bin

**Affiliations:** 1 Guangzhou University of Chinese Medicine, Guangzhou, China; 2 Chinese Medicine Guangdong Laboratory (Hengqin Laboratory), Zhuhai, China

**Keywords:** adverse drug reactions, disproportionality analysis, FAERS, insomnia, Pharmacoepidemiology, pharmacovigilance, sex differences

## Abstract

Insomnia is a frequent and clinically relevant adverse drug reaction that can impair quality of life, treatment adherence and long-term outcomes. Evidence on drug-associated insomnia has largely been derived from selected clinical trial populations or focused on individual drug classes, while comprehensive post-marketing assessments—particularly those considering potential sex-related heterogeneity—remain limited. Using the U.S. Food and Drug Administration (FDA) Adverse Event Reporting System (FAERS), we conducted a large-scale pharmacovigilance analysis to identify and characterise drug–insomnia signals of disproportionate reporting (SDRs) and to explore potential sex-related heterogeneity in reporting patterns. Individual case safety reports from January 2019 to March 2025 were analysed using a transparent, multi-step preprocessing pipeline, including removal of deleted cases, consolidation by case identifier and case version, and additional rule-based deduplication across case identifiers. Insomnia cases were identified using a narrow set of MedDRA Preferred Terms (PTs)—insomnia, initial insomnia, middle insomnia, terminal insomnia, and early morning awakening—and analyses were restricted to parent systemic drugs. Disproportionality was assessed using reporting odds ratios (RORs) in primary-suspect and any-suspect analyses. Sex-stratified RORs were estimated for female and male reports, and formal heterogeneity was evaluated using interaction-based metrics with false discovery rate control. The final analytic cohort comprised 2,935,560 unique reports, of which 74,444 contained insomnia reactions after exclusion of sleep-related indications. A broad spectrum of psychotropic and non-psychotropic agents showed SDRs for insomnia, spanning hypnotics, antineoplastic therapies, immunomodulators, endocrine agents and commonly used anti-infectives. Sex-stratified analyses revealed largely overlapping signal profiles between females and males, and formal heterogeneity testing identified few drug–insomnia pairs with robust evidence of sex-related heterogeneity after multiple-testing correction. These findings represent signals of disproportionate reporting rather than estimates of incidence or causal risk. Observed sex-related heterogeneity should therefore be interpreted as hypothesis-generating and may reflect heterogeneity in exposure prevalence, prescribing indications and reporting context rather than intrinsic biological susceptibility. Overall, this study provides a contemporary overview of drug-associated insomnia reporting in FAERS and highlights drug–sex combinations that may warrant further investigation in analytically adjusted pharmacoepidemiologic studies.

## Introduction

Insomnia—difficulty initiating or maintaining sleep or experiencing early morning awakenings—is a common complaint in clinical practice and a recognised adverse effect of many medications (Jain and Jain, 2011; Van Gastel, 2018; Doufas et al., 2017). Drugs from diverse classes, including psychotropics, respiratory agents, endocrine therapies and oncologic treatments, can disturb sleep through effects on multiple neurotransmitter and hormonal systems (Jain and Jain, 2011; Doufas et al., 2017; Natter et al., 2021). Drug-induced insomnia can impair quality of life, reduce adherence, exacerbate underlying psychiatric and somatic conditions and contribute to healthcare utilisation (Jain and Jain, 2011; Van Gastel, 2018; Doufas et al., 2017).

Spontaneous reporting systems such as the FDA Adverse Event Reporting System (FAERS) are key components of post-marketing safety surveillance (U.S. Food and Drug Administration, 2018; U.S. Food and Drug Administration, 2014). FAERS aggregates voluntary reports of suspected adverse drug reactions (ADRs) from healthcare professionals, consumers and manufacturers, and is routinely mined using disproportionality methods to detect signals of disproportionate reporting for specific drug–event combinations (Evans et al., 2001; Bate and Evans, 2009; Wisniewski et al., 2016; Cutroneo et al., 2023; Van der Heijden et al., 2002). Disproportionality methods quantify relative reporting of a target event for each drug within the database (e.g., ROR or PRR) and are widely used for signal screening (Evans et al., 2001; Bate and Evans, 2009; Wisniewski et al., 2016; Cutroneo et al., 2023; Van der Heijden et al., 2002). Although disproportionality analyses cannot establish causality and are sensitive to under-reporting, confounding and changes in reporting behaviour (Evans et al., 2001; Bate and Evans, 2009; Wisniewski et al., 2016; Cutroneo et al., 2023; Van der Heijden et al., 2002; Hammad et al., 2025), they are valuable tools for hypothesis generation and signal prioritisation.

Sex differences in pharmacokinetics, pharmacodynamics, disease epidemiology and reporting behaviour have been described for several ADRs (Watson et al., 2019; Yu et al., 2016; De Vries et al., 2019; Visser et al., 2021; Zucker et al., 2020). Aggregated analyses of pharmacovigilance databases have suggested that women contribute more ADR reports overall and may be at higher risk of some reactions, but the magnitude and direction of sex differences vary by drug class and reaction type (Watson et al., 2019; Yu et al., 2016; De Vries et al., 2019; Visser et al., 2021; Zucker et al., 2020). Evidence on sex-specific risks of insomnia across a broad range of drugs remains limited. Existing work has largely focused on selected hypnotics or on descriptive analyses of sleep-related ADRs (Jain and Jain, 2011; Van Gastel, 2018; Doufas et al., 2017; Natter et al., 2021), often without formal sex-stratified disproportionality or interaction testing. Beyond pharmacokinetic and pharmacodynamic differences, accumulating evidence indicates that sex differences are also present in the epidemiology, clinical presentation and neurobiological substrates of neurological and mental disorders, including sleep–wake regulation. Sex differences in sleep and insomnia are increasingly recognised at multiple biological levels, including brain network organisation, hormonal regulation and immune–neuroendocrine interactions, all of which may influence vulnerability to sleep disturbances and responses to pharmacological treatments (Riemann et al., 2015; Mong and Cusmano, 2016; Besedovsky et al., 2012). These sex-related differences have also been discussed in broader neurological and mental disorder frameworks, supporting the rationale for sex-aware safety evaluations (Zhu and Lv, 2025a). These observations highlight the importance of incorporating sex-aware perspectives into both clinical research and pharmacovigilance analyses of sleep-related adverse drug reactions.

The aims of this study were therefore two-fold. First, we sought to provide an up-to-date and comprehensive overview of parent systemic drugs associated with insomnia in FAERS during 2019–Q1 2025, using a harmonised parent-drug–level pipeline restricted to systemic routes of administration. Second, we aimed to characterise sex-related heterogeneity in these insomnia signals through stratified disproportionality analyses and formal interaction testing. By combining systematic signal screening with a contemporary observation window and sex-specific analyses, this work is intended to complement evidence from clinical trials and prescribing information and to generate hypotheses that may inform future sex-sensitive pharmacovigilance and pharmacoepidemiologic studies (European Medicines Agency, 2006; Wisniewski et al., 2016; Cutroneo et al., 2023; Fusaroli et al., 2024; Hammad et al., 2025).

## Methods

### Data source and study period

We analysed individual case safety reports from 1 January 2019 to 31 March 2025 in FAERS (U.S. Food and Drug Administration, 2018; U.S. Food and Drug Administration, 2014). This period was chosen to focus on contemporary reporting patterns and to minimise structural changes present in earlier FAERS data. It also encompasses the coronavirus disease 2019 (COVID-19) pandemic, during which substantial changes in healthcare utilisation, prescribing and reporting may have affected both the mix and volume of reports; this is considered in the interpretation of findings.

The quarterly ASCII archives (DEMO, DRUG, REAC, INDI and deleted-case files) were downloaded or accessed locally and parsed using the faers R package and custom scripts. Adverse events are coded using MedDRA, and drug information is recorded in multiple free-text and structured fields.

### Case processing and deduplication

**FIGURE 1:**
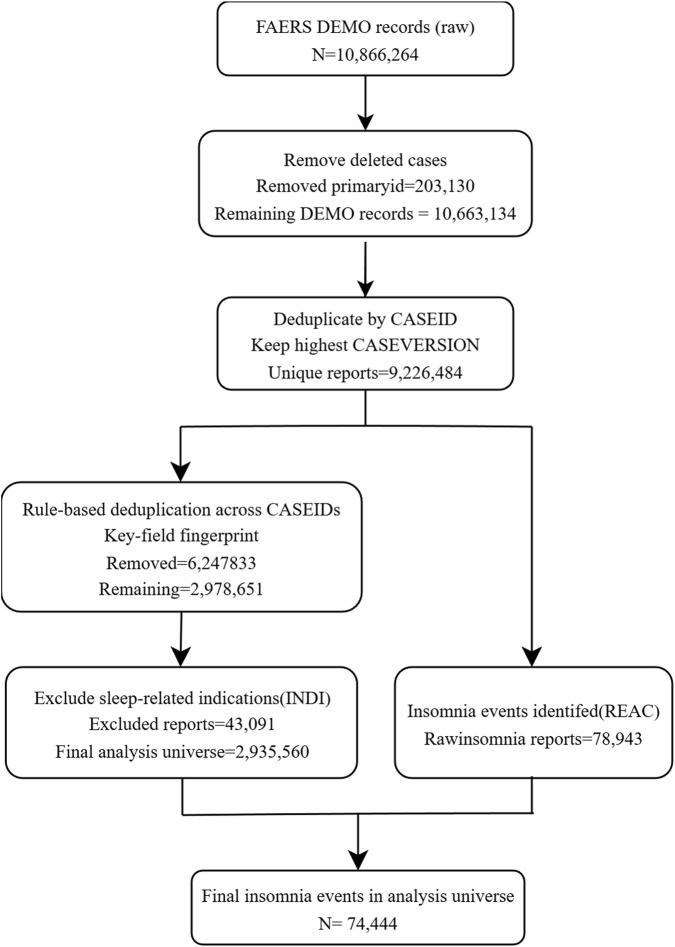
Construction of the analytic cohort from FAERS reports (2019–Q1 2025). Flowchart showing removal of deleted cases, deduplication by CASEID (retaining the highest CASEVERSION), rule-based deduplication across CASEIDs (key-field fingerprint), identification of insomnia reactions (REAC) and exclusion of sleep-related indications (INDI), resulting in a final analysis universe of 2,935,560 reports and 74,444 insomnia reports.

We first excluded reports flagged as deleted in the FDA deleted-case files (U.S. Food and Drug Administration, 2018; U.S. Food and Drug Administration, 2014). Remaining reports were deduplicated by case identifier (CASEID), retaining the record with the highest CASEVERSION, in accordance with FDA recommendations (U.S. Food and Drug Administration, 2018; U.S. Food and Drug Administration, 2014). We additionally applied rule-based deduplication across CASEIDs using a key-field fingerprint (Figure 1). After identification of insomnia reactions and exclusion of sleep-related indications, the final analysis universe comprised 2,935,560 unique reports (Figure 1).

Characteristics of the final analysis universe were summarized using standard descriptive statistics: continuous variables were reported as median (interquartile range, IQR), and categorical variables were reported as counts and percentages. Summaries were generated for demographics, reporter type, and recorded route of administration, with detailed results presented in the Results section and Supplementary Tables.

### Definition of insomnia cases

Insomnia cases were defined *a priori* using a narrow, clinically face-valid set of MedDRA Preferred Terms (PTs) in the REAC table: insomnia, initial insomnia, middle insomnia, terminal insomnia, and early morning awakening (Jain and Jain, 2011; Van Gastel, 2018; Doufas et al., 2017). This PT cluster is not a standardized MedDRA grouping (e.g., SMQ); it was selected to prioritize specificity and reduce contamination from loosely related sleep complaints, guided by clinical face validity and prior literature on drug-associated insomnia (Jain and Jain, 2011; Van Gastel, 2018; Doufas et al., 2017).

To minimize confounding by indication (i.e., insomnia recorded as the reason for prescribing rather than an adverse reaction), we excluded reports in which the INDI table contained sleep-related indications, including insomnia/sleeplessness and broader sleep disorder terminology (e.g., sleep disorder/disturbance, dyssomnia), circadian rhythm sleep–wake disorders (including non-24-h sleep–wake disorder), parasomnias, restless legs syndrome, and narcolepsy. This exclusion was implemented using a prespecified regular-expression pattern aligned with these indication categories (Figure 1), and the full operational pattern/term list is provided in Supplementary Tables.

### Drug exposure definition, routes and parent-drug normalisation

Drug exposures were derived from the DRUG table. We focused on parent systemic drugs administered via oral, intravenous (including unspecified intravenous and intravenous drip), intramuscular, subcutaneous, inhalation/respiratory, transdermal or nasal routes. Topical, ophthalmic, otic and other clearly local formulations were not considered parent systemic drugs for disproportionality analyses. Reports involving only non-systemic routes were excluded from the disproportionality analyses. Transdermal products were retained only when intended for systemic delivery, consistent with the route coding and product context in FAERS (Bate and Evans, 2009; European Medicines Agency, 2006; Wisniewski et al., 2016; Cutroneo et al., 2023; Van der Heijden et al., 2002; Hammad et al., 2025).

To minimise artificial splitting of signals across salt forms and formulations, proprietary names and active ingredient strings were normalised to parent drugs using a rule-based mapping function. This function removed salt/ion terms (e.g., hydrochloride, mesylate), hydrates, release modifiers (extended-release, controlled-release, XR, SR, etc.), species descriptors (e.g., porcine) and non-alphanumeric symbols, followed by lower-casing and whitespace trimming, and when possible was informed by inspection of high-frequency strings and cross-checked against WHO ATC/DDD and FDA Structured Product Labeling ingredient lists for common salts and modified-release formulations (Evans et al., 2001; Bate and Evans, 2009; European Medicines Agency, 2006; Wisniewski et al., 2016; Cutroneo et al., 2023; van der Heijden et al., 2002; Hammad et al., 2025). Fixed-dose combinations were not decomposed; such products were mapped to a composite parent label (e.g., “pseudoephedrine + antihistamine”), and signals for these parents should therefore be interpreted as relating to the combination rather than individual components. All disproportionality analyses were conducted at this parent-drug level.

### Disproportionality analyses

We constructed 2 × 2 contingency tables for each parent systemic drug and insomnia as the event of interest (Evans et al., 2001; Bate and Evans, 2009; Wisniewski et al., 2016; Cutroneo et al., 2023; Van der Heijden et al., 2002).

We selected the reporting odds ratio (ROR) as the primary disproportionality measure, with PRR reported as a complementary metric, because both are widely used, transparent, and facilitate comparison with prior pharmacovigilance studies and guidance documents (Evans et al., 2001; Bate and Evans, 2009; Wisniewski et al., 2016; Cutroneo et al., 2023; Van der Heijden et al., 2002). Bayesian shrinkage metrics (e.g., Information Component or EBGM) were not included in the primary analysis to preserve interpretability and consistency with the prespecified analytic plan; evaluating robustness with alternative disproportionality frameworks may be addressed in future work. The primary analysis used drugs coded as primary suspect (PS); a secondary analysis considered drug–report pairs in which the drug was coded as a primary or secondary suspect (ANY, i.e., PS + SS). Both PS and ANY analyses were restricted to parent systemic drugs with the routes defined above.

Let a denote insomnia reports with the drug of interest, b insomnia reports without the drug, c non-insomnia reports with the drug and d non-insomnia reports without the drug within the relevant “universe” of report IDs (Evans et al., 2001; Bate and Evans, 2009; Wisniewski et al., 2016; Cutroneo et al., 2023; Van der Heijden et al., 2002). To avoid contamination between analyses, event sets and drug-exposed IDs were intersected with the current universe of report identifiers for each analysis.

For inclusion in the PS set, we required a ≥5 and (a + c) ≥ 50, in line with previous FAERS and EudraVigilance work and regulatory guidance (Evans et al., 2001; Bate and Evans, 2009; European Medicines Agency, 2006; Wisniewski et al., 2016; Van der Heijden et al., 2002). To reduce instability from zero cells, Haldane–Anscombe 0.5 continuity corrections were applied to all 4 cells (Evans et al., 2001; Bate and Evans, 2009; European Medicines Agency, 2006; Wisniewski et al., 2016; Van der Heijden et al., 2002). For each drug we computed:

1. The reporting odds ratio (ROR) = (a·d)/(b·c) with standard error SE[log(ROR)] = √(1/a +1/b + 1/c + 1/d) and 95% CI = exp[log(ROR) ± 1.96 · SE];
2. The proportional reporting ratio (PRR) = [a/(a + b)]/[c/(c + d)] with an analogous log-scale SE and 95% CI.

Forest plots for the strongest signals were generated using ggplot2 on a logarithmic scale, with a vertical reference line at ROR = 1 (Figures 2, 3; Supplementary Figures S1–S6).

**FIGURE 2:**
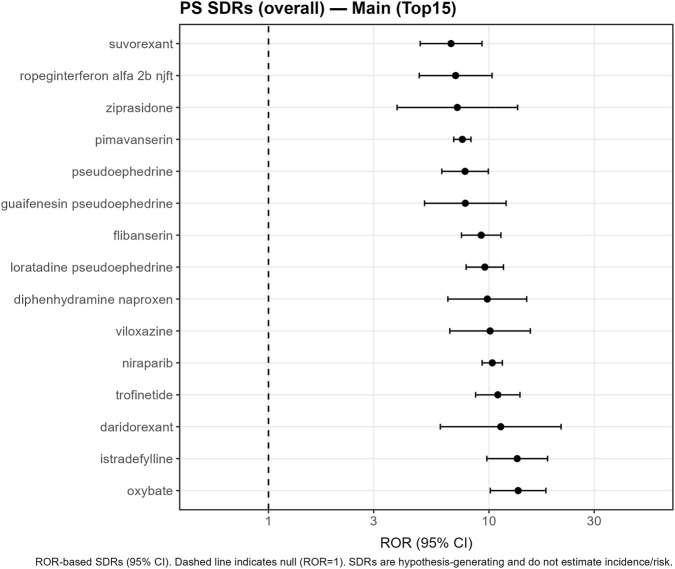
Disproportionality analysis of insomnia reporting in FAERS (2019–Q1 2025), primary-suspect analysis (PS set). Forest plot of RORs (95% CI) for the top 15 parent systemic drugs with the highest RORs for insomnia in the PS analysis, restricted to systemic routes of administration. Points and horizontal lines show the ROR and 95% confidence interval on a logarithmic scale; the vertical dashed line indicates ROR = 1. Numerical values correspond to those in Table 2.

**FIGURE 3:**
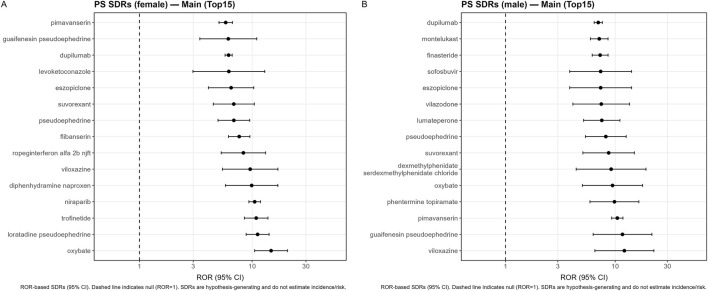
Sex-stratified disproportionality analysis of insomnia reporting (parent systemic drugs, PS set). **(A)** Female reports and **(B)** male reports. Each panel shows a forest plot of RORs (95% CI) for the top 15 signals in the corresponding sex stratum, plotted on a logarithmic scale with a reference line at ROR = 1.

### Sex-stratified analyses and heterogeneity testing

Sex-stratified PS analyses were conducted by repeating the above approach within female and male report subsets (Watson et al., 2019; Yu et al., 2016; de Vries et al., 2019; Visser et al., 2021; Zucker et al., 2020). For each drug we extracted the raw cell counts (aF, bF, cF, dF and aM, bM, cM, dM) and computed sex-specific RORs (ROR_F, ROR_M) with 0.5 corrections. Drugs with exposure limited to a single sex (e.g., finasteride, estrogens, testosterone) were classified as sex-specific and not subjected to interaction testing; these were tabulated separately (Supplementary Tables S3–S4b).

For drugs with non-zero exposure in both sexes and at least five insomnia reports in each sex stratum (aF ≥ 5 and aM ≥ 5), we evaluated sex-related heterogeneity using two complementary approaches:

1. Difference in log-ROR: logROR_diff = log(ROR_F) − log(ROR_M) with standard error SE_diff = √(SE_F^2^ + SE_M^2^). A z statistic (z_diff = logROR_diff/SE_diff) and two-sided p value (p_diff) were computed under the null hypothesis of equal RORs.
2. Breslow–Day test and Mantel–Haenszel common odds ratio: We formed a 2 × 2 × 2 array (event × drug × sex) and applied the Breslow–Day test for homogeneity of odds ratios across the female and male strata, accompanied by estimation of a common odds ratio (MH_OR) and corresponding p value via the Mantel–Haenszel test (Breslow and Day, 1980).

For both p_diff and BD_p, multiple testing was addressed using the Benjamini–Hochberg FDR method, controlling the expected proportion of false discoveries at 5% (Benjamini and Hochberg, 1995). To reduce spurious interaction signals, Breslow–Day tests were performed only for drugs meeting this minimum stratum-specific count criterion. Accordingly, sex-stratified disproportionality estimates are interpreted as exploratory indicators of potential heterogeneity rather than as measures of sex-specific causal risk.

### Statistical software and reporting

All analyses were conducted in R (version 4.3.2; R Foundation for Statistical Computing, Vienna, Austria) using the data.table, dplyr, stringr, ggplot2, readr and DescTools packages. The overall analysis was implemented in a reproducible pipeline, with code and non-identifiable summary outputs provided in the Supplementary Material. Reporting of methods and results was aligned, as far as possible, with guidance from the READUS-PV statement and related recommendations for transparent disproportionality analyses (European Medicines Agency, 2006; Wisniewski et al., 2016; Cutroneo et al., 2023; Fusaroli et al., 2024; Hammad et al., 2025).

## Results

### Descriptive characteristics

Figure 1 summarises construction of the analytic cohort. After removal of deleted cases, deduplication by CASEID (retaining the highest CASEVERSION), rule-based deduplication across CASEIDs using a key-field fingerprint, identification of insomnia reactions, and exclusion of sleep-related indications, the final analysis universe comprised 2,935,560 unique reports (Figure 1). Within this universe, 74,444 reports contained insomnia reactions (Figure 1). Characteristics of the final analysis universe are summarized in Table 1, and a detailed breakdown of recorded routes among insomnia reports is provided in Supplementary Table S1.

**TABLE 1:** Characteristics of insomnia reports in the final FAERS analysis universe (2019–Q1 2025).

Characteristic	Value
Total reports	2,935,560
Age, years (median [IQR])	58.0 [40.0–71.0]
Sex, n (%) — female	1,439,430 (49.0)
Sex, n (%) — male	1,175,658 (40.0)
Sex, n (%) — unknown	320,472 (10.9)
Insomnia reports with recorded systemic route (N)	104,980
Administration route, n (%) — oral	33,502 (31.9)
Administration route, n (%) — parenteral*	22,778 (21.7)
Administration route, n (%) — other/unknown	48,700 (46.5)

The final analysis universe was obtained after removing deleted cases, deduplicating by CASEID (retaining the highest CASEVERSION), applying rule-based deduplication across CASEIDs, and excluding reports with sleep-related indications (see Figure 1). Administration route is summarized only among insomnia reports that met the prespecified systemic-route framework and had a recorded route (N=104,980); percentages shown in the route section use this denominator. Reports without a usable route value are not included in the route denominator. Systemic routes were defined a priori as oral, parenteral, respiratory, ocular, auricular, or nasal; topical/transdermal/rectal/vaginal routes were excluded. Parenteral includes intravenous, intramuscular, and subcutaneous routes. Other/unknown includes uncommon systemic routes, multiple routes, and unspecified/unknown route values.

In the final analysis universe, the median age was 58 years (IQR 40–71), and females accounted for 49.0% of reports (Table 1).Route information was variably recorded; among reports contributing to the systemic-route analytic universe with a recorded route, oral administration was most common, followed by parenteral routes and other/unknown systemic routes (Table 1; Supplementary Table S1).

### Overall insomnia signals

In the PS analysis, parent systemic drugs meeting the minimum reporting thresholds were retained for signal screening. Figure 2 and Table 2 summarise the top 15 parent systemic drugs with the highest RORs for insomnia in the PS set. The strongest signals were observed for oxybate (ROR 13.56, 95% CI 10.14–18.13) and istradefylline (13.43, 9.78–18.44), followed by daridorexant (11.31, 6.02–21.24), trofinetide (10.97, 8.70–13.82) and niraparib (10.35, 9.31–11.50) (Table 2; Figure 2). Additional high-ranking signals included viloxazine, diphenhydramine + naproxen, loratadine + pseudoephedrine, flibanserin, guaifenesin + pseudoephedrine, pseudoephedrine, pimavanserin, ziprasidone, ropeginterferon alfa-2b-njft, and suvorexant (Table 2; Figure 2). These disproportionality estimates represent signals of disproportionate reporting rather than incidence or causal risk.

**TABLE 2:** Top 15 parent systemic drugs with disproportionate reporting of insomnia (PS analysis).

Parent systemic drug	Insomnia reports (a)	Exposed PS reports (a+c)	ROR (95% CI)	PRR (95% CI)
Oxybate	61	235	13.56 (10.14–18.13)	13.55 (10.13–18.11)
Istradefylline	51	198	13.43 (9.78–18.44)	13.42 (9.77–18.43)
Daridorexant	12	54	11.31 (6.02–21.24)	11.30 (6.02–21.24)
Trofinetide	92	416	10.97 (8.70–13.82)	10.96 (8.70–13.80)
Niraparib	437	2,070	10.35 (9.31–11.50)	10.29 (9.26–11.44)
Viloxazine	27	131	10.12 (6.65–15.40)	10.11 (6.65–15.39)
Diphenhydramine naproxen	28	139	9.83 (6.51–14.83)	9.82 (6.51–14.82)
Loratadine pseudoephedrine	126	634	9.58 (7.88–11.64)	9.56 (7.87–11.61)
Flibanserin	112	581	9.22 (7.51–11.33)	9.21 (7.50–11.31)
Guaifenesin pseudoephedrine	25	150	7.81 (5.10–11.96)	7.81 (5.10–11.95)
Pseudoephedrine	78	465	7.79 (6.11–9.93)	7.79 (6.11–9.92)
Pimavanserin	570	3,483	7.58 (6.92–8.29)	7.53 (6.88–8.23)
Ziprasidone	11	72	7.19 (3.83–13.49)	7.19 (3.83–13.49)
Ropeginterferon alfa 2b njft	31	202	7.06 (4.83–10.33)	7.06 (4.83–10.32)
Suvorexant	43	291	6.73 (4.88–9.29)	6.73 (4.88–9.28)

Primary-suspect (PS) disproportionality analysis restricted to parent systemic drugs and prespecified systemic routes. Haldane–Anscombe continuity correction (0.5) was applied to all 2×2 cells for ROR/PRR estimation. ROR/PRR quantify disproportionate reporting within FAERS and do not estimate incidence or causal risk.

In the ANY analysis, which considered drugs coded as primary or secondary suspect, point estimates of insomnia association were generally similar or modestly attenuated compared with the PS analysis. Chlorhexidine showed an unusually high ROR driven by a relatively small cluster of insomnia reports (Supplementary Figure S6). Importantly, disproportionality screening in this study was restricted to the prespecified systemic-route framework; therefore, reports involving only clearly non-systemic routes were not part of the analytic universe for disproportionality analyses. The additional route stratification shown for chlorhexidine is descriptive and based on the recorded route field among included reports, highlighting that this cluster was enriched in potentially systemic, ambiguous, or poorly specified routes, whereas clearly topical-only contexts were uncommon within the included set. Given the small number of contributing reports, potential route misclassification, and atypical exposure context, this observation should be interpreted cautiously as hypothesis-generating (U.S. Food and Drug Administration, 2018; U.S. Food and Drug Administration, 2014; Bate and Evans, 2009; European Medicines Agency, 2006; Wisniewski et al., 2016; Cutroneo et al., 2023; Van der Heijden et al., 2002; Fusaroli et al., 2024; Hammad et al., 2025).

### Sex-stratified insomnia signals

Sex-stratified PS analyses yielded estimable RORs for a substantial proportion of drugs in both female and male reports. Forest plots of the strongest sex-specific PS signals are presented in Figure 3A (females) and Figure 3B (males). In females, leading signals included oxybate, trofinetide, niraparib, and several combination products containing pseudoephedrine (Figure 3A). In males, leading signals included dupilumab, montelukast, finasteride, sofosbuvir, es(z)opiclone, vilazodone, and stimulant-related exposures (Figure 3B). Drugs with exposure limited to a single sex (e.g., finasteride, estrogens, testosterone) were classified as sex-specific and not subjected to interaction testing; these are reported separately in Supplementary Tables S3–S4b.

### Sex heterogeneity in insomnia reporting

Among drugs with exposure in both sexes and sufficient stratum-specific counts for interaction testing, formal heterogeneity testing did not identify robust sex-related heterogeneity after Benjamini–Hochberg FDR correction. Consistent with this, the sex-heterogeneity volcano plot shows that essentially no drug–insomnia pairs exceed the FDR significance threshold (Supplementary Figure S7). These results should be interpreted as exploratory and hypothesis-generating.

## Discussion

In this large FAERS-based pharmacovigilance analysis of contemporary reports from 2019 to Q1 2025, we identified a broad spectrum of parent systemic drugs disproportionately reported with insomnia. After a transparent cohort construction process—including removal of deleted cases, CASEID/CASEVERSION consolidation, additional rule-based deduplication across CASEIDs, and exclusion of reports with sleep-related indications—we analysed 2,935,560 unique reports, among which 74,444 contained insomnia reactions (Figure 1). Using a harmonised parent-drug pipeline restricted to systemic routes, we observed signals of disproportionate reporting (SDRs) across diverse therapeutic areas, spanning psychotropic agents, hypnotics, antineoplastic therapies, immunomodulators, endocrine agents, anti-infectives and sympathomimetic-containing combinations (Table 2; Figures 2, 3). These findings emphasise that insomnia reporting may arise across a wide range of pharmacological classes rather than being confined to classic activating psychotropics (Jain and Jain, 2011; Van Gastel, 2018; Doufas et al., 2017; Natter et al., 2021; Bate and Evans, 2009; European Medicines Agency, 2006; Wisniewski et al., 2016; Cutroneo et al., 2023; Van der Heijden et al., 2002; Fusaroli et al., 2024; Hammad et al., 2025).

Several high-ranking SDRs involved drugs whose pharmacology is broadly consistent with potential effects on sleep–wake regulation or arousal pathways. Agents affecting orexin signalling, dopaminergic tone or immune-mediated pathways may plausibly contribute to sleep disruption in specific clinical contexts, either directly or indirectly via symptom burden and treatment-related behavioural changes (Doufas et al., 2017; Jiang et al., 2024). At the same time, marked disproportionality among non-psychotropic therapies suggests that insomnia reporting in pharmacovigilance databases often reflects complex real-world contexts, including systemic inflammation, cancer-related symptom clusters, endocrine dysregulation or concomitant use of stimulants and other activating co-treatments (Jain and Jain, 2011; Van Gastel, 2018; Doufas et al., 2017; Natter et al., 2021; Bate and Evans, 2009; European Medicines Agency, 2006; Wisniewski et al., 2016; Cutroneo et al., 2023; Van der Heijden et al., 2002; Hammad et al., 2025). Because FAERS captures suspected associations rather than verified causal effects, these SDRs should be interpreted cautiously as hypothesis-generating signals that may help prioritise drug–event pairs for further evaluation (Bate and Evans, 2009; European Medicines Agency, 2006; Wisniewski et al., 2016; Cutroneo et al., 2023; Van der Heijden et al., 2002; Fusaroli et al., 2024; Hammad et al., 2025).

A key interpretive issue in insomnia pharmacovigilance is the presence of insomnia SDRs for therapies used to treat sleep disorders, such as hypnotic agents. Although insomnia is a target symptom for these drugs, they may nonetheless appear among agents disproportionately reported with insomnia in spontaneous reporting systems (Jain and Jain, 2011; Van Gastel, 2018; Doufas et al., 2017; Bate and Evans, 2009; European Medicines Agency, 2006; Wisniewski et al., 2016; Cutroneo et al., 2023; Van der Heijden et al., 2002; Hammad et al., 2025). This apparently paradoxical pattern can arise through non-causal mechanisms, including co-reporting of treatment inefficacy, persistence or recurrence of symptoms despite therapy, rebound insomnia following dose changes or discontinuation, and indication-related reporting biases (Jain and Jain, 2011; Van Gastel, 2018; Doufas et al., 2017). Such mechanisms may persist even after efforts to minimise contamination from sleep-related indications, as indications are often incompletely captured or inconsistently coded (Bate and Evans, 2009; European Medicines Agency, 2006; Wisniewski et al., 2016; Cutroneo et al., 2023; Van der Heijden et al., 2002; Hammad et al., 2025). Accordingly, insomnia SDRs observed for hypnotics should be interpreted within their therapeutic and reporting context rather than as evidence of paradoxical pharmacological effects (Jain and Jain, 2011; Van Gastel, 2018; Doufas et al., 2017; Bate and Evans, 2009; European Medicines Agency, 2006; Wisniewski et al., 2016; Cutroneo et al., 2023; Van der Heijden et al., 2002; Fusaroli et al., 2024; Hammad et al., 2025).

In the any-suspect analysis, point estimates were generally similar to or modestly attenuated compared with the primary-suspect analysis, consistent with the broader and less specific nature of this definition (Bate and Evans, 2009; European Medicines Agency, 2006; Wisniewski et al., 2016; Cutroneo et al., 2023; Van der Heijden et al., 2002; Hammad et al., 2025). The conspicuous chlorhexidine SDR illustrates how systematic screening may surface unexpected drug–event combinations that merit closer scrutiny (Bate and Evans, 2009; European Medicines Agency, 2006; Wisniewski et al., 2016; Cutroneo et al., 2023; van der Heijden et al., 2002; Fusaroli et al., 2024; Hammad et al., 2025). Given the atypical nature of systemic exposure, the small number of contributing reports and the possibility of route misclassification, this observation should be interpreted with particular caution (U.S. Food and Drug Administration, 2018; U.S. Food and Drug Administration, 2014; Bate and Evans, 2009; European Medicines Agency, 2006; Wisniewski et al., 2016; Cutroneo et al., 2023; Van der Heijden et al., 2002; Hammad et al., 2025). More broadly, unusually extreme estimates in spontaneous reporting systems may reflect artefacts, atypical reporting clusters or unmeasured confounding, and should therefore be regarded as hypothesis-generating pending independent verification (Bate and Evans, 2009; European Medicines Agency, 2006; Wisniewski et al., 2016; Cutroneo et al., 2023; Van der Heijden et al., 2002; Fusaroli et al., 2024; Hammad et al., 2025).

Sex-stratified analyses showed that, for most drugs, insomnia reporting was broadly similar between female and male reports. Among drugs with exposure in both sexes and sufficient stratum-specific counts, formal heterogeneity testing identified little evidence of robust sex-related differences after correction for multiple testing (Benjamini and Hochberg, 1995; Fusaroli et al., 2024). This pattern does not contradict prior evidence of sex differences in overall ADR reporting but suggests that large, reproducible sex divergences in insomnia disproportionality may be uncommon at the level detectable with spontaneous-report data when conservative multiplicity control is applied (Watson et al., 2019; Yu et al., 2016; De Vries et al., 2019; Visser et al., 2021; Zucker et al., 2020).

Crucially, any observed sex differences in stratified RORs should not be interpreted as direct indicators of biological susceptibility. In FAERS, such estimates may be influenced by differences in exposure prevalence, prescribing indications, disease epidemiology and reporting behaviour (Bate and Evans, 2009; European Medicines Agency, 2006; Wisniewski et al., 2016; Cutroneo et al., 2023; van der Heijden et al., 2002; Hammad et al., 2025; Watson et al., 2019; Yu et al., 2016; De Vries et al., 2019; Visser et al., 2021; Zucker et al., 2020). While biological sex differences in sleep regulation and neuroendocrine–immune interactions are well described, spontaneous reporting data do not permit disentangling these mechanisms from contextual determinants of reporting (Riemann et al., 2015; Mong and Cusmano, 2016; Besedovsky et al., 2012). Accordingly, sex-specific SDRs are best viewed as hypotheses that may inform targeted pharmacoepidemiologic studies with validated sleep outcomes and appropriate confounding control (Bate and Evans, 2009; European Medicines Agency, 2006; Wisniewski et al., 2016; Cutroneo et al., 2023; van der Heijden et al., 2002; Fusaroli et al., 2024; Hammad et al., 2025).

From a clinical perspective, these findings underscore that insomnia should be considered as a potential adverse drug reaction across many therapeutic areas (Jain and Jain, 2011; Van Gastel, 2018; Doufas et al., 2017; Natter et al., 2021). Recognising possible drug-associated contributions to insomnia may support pragmatic management strategies, including counselling on sleep hygiene, reviewing dose timing, assessing the role of concomitant activating medications and considering alternative therapies when appropriate (Jain and Jain, 2011; Van Gastel, 2018; Doufas et al., 2017; Riemann et al., 2015; Mong and Cusmano, 2016; Besedovsky et al., 2012). At the same time, insomnia reports for symptom-targeting drugs may reflect persistent disease or treatment ineffectiveness rather than adverse drug effects (Jain and Jain, 2011; Van Gastel, 2018; Doufas et al., 2017; Bate and Evans, 2009; European Medicines Agency, 2006; Wisniewski et al., 2016; Cutroneo et al., 2023; van der Heijden et al., 2002; Hammad et al., 2025). Overall, by providing a contemporary overview of insomnia SDRs and a structured assessment of sex-related patterns using a transparent analytical pipeline, this study aims to support hypothesis generation and prioritisation for future validation studies rather than to define definitive risk profiles (Bate and Evans, 2009; European Medicines Agency, 2006; Wisniewski et al., 2016; Cutroneo et al., 2023; Van der Heijden et al., 2002; Fusaroli et al., 2024; Hammad et al., 2025).

### Strengths and limitations

This study has several strengths. First, we used a prespecified and transparent workflow to extract and preprocess FAERS data, including removal of deleted cases, CASEID/CASEVERSION consolidation, and additional rule-based deduplication across CASEIDs, followed by reproducible generation of summary tables and figures (U.S. Food and Drug Administration, 2018; U.S. Food and Drug Administration, 2014; Fusaroli et al., 2024). Second, we implemented parent-drug normalisation to reduce artificial fragmentation of signals across salts, hydrates, proprietary names and modified-release formulations, which is a common challenge in spontaneous reporting systems with heterogeneous drug naming (Bate and Evans, 2009; European Medicines Agency, 2006; Wisniewski et al., 2016; Cutroneo et al., 2023; Van der Heijden et al., 2002; Hammad et al., 2025). Third, the analyses were restricted to systemic routes of administration to improve biological plausibility and interpretability of insomnia SDRs, and we applied consistent inclusion thresholds and continuity corrections to mitigate instability in sparse cells (Evans et al., 2001; Bate and Evans, 2009; European Medicines Agency, 2006; Wisniewski et al., 2016; Cutroneo et al., 2023; Van der Heijden et al., 2002). Fourth, we used a narrow, clinically focused insomnia event definition and excluded reports with sleep-related indications, aiming to prioritise specificity and reduce conflation of underlying sleep disorders or symptom-directed prescribing with adverse reactions (Jain and Jain, 2011; Van Gastel, 2018; Doufas et al., 2017). Finally, sex-stratified analyses combined stratified ROR estimation with formal heterogeneity testing and multiplicity control, aligning reporting and interpretive principles with the READUS-PV framework (Benjamini and Hochberg, 1995; Fusaroli et al., 2024).

Important limitations are inherent to spontaneous reporting data and to disproportionality analyses. FAERS is subject to under-reporting, stimulated reporting, and temporal changes in reporting behaviour, all of which can affect disproportionality estimates independently of any causal relationship (Bate and Evans, 2009; European Medicines Agency, 2006; Wisniewski et al., 2016; Cutroneo et al., 2023; Van der Heijden et al., 2002; Hammad et al., 2025). Disproportionality metrics such as the ROR and PRR quantify SDRs within the reporting database rather than incidence or absolute risk in treated populations, and they cannot establish causality (Evans et al., 2001; Bate and Evans, 2009; European Medicines Agency, 2006; Wisniewski et al., 2016; Cutroneo et al., 2023; Van der Heijden et al., 2002; Fusaroli et al., 2024; Hammad et al., 2025). Accordingly, all results should be interpreted as hypothesis-generating signals rather than as definitive risk estimates.

Confounding by indication and comorbidity is a major concern for insomnia. Insomnia is influenced by multiple clinical and contextual factors (e.g., pain, anxiety/depression, cardiometabolic disease, substance use, and concurrent medications), many of which are incompletely captured and not fully adjustable in spontaneous reporting data (Zhu and Lv, 2025b). Insomnia reports may therefore reflect underlying disease activity, symptom burden, or care pathways rather than a drug effect *per se* (Jain and Jain, 2011; Van Gastel, 2018; Doufas et al., 2017; Natter et al., 2021). Although we excluded reports with sleep-related indications to reduce symptom-directed prescribing bias, indication fields in FAERS may be incomplete, inconsistently coded, or missing, and residual confounding is likely (Bate and Evans, 2009; European Medicines Agency, 2006; Wisniewski et al., 2016; Cutroneo et al., 2023; Van der Heijden et al., 2002; Hammad et al., 2025). In addition, concomitant medications were not adjusted for analytically; because many reports include multiple suspect and concomitant drugs and role assignment may vary across reporters, disproportionality methods alone cannot reliably disentangle independent contributions of co-administered therapies (Bate and Evans, 2009; European Medicines Agency, 2006; Wisniewski et al., 2016; Cutroneo et al., 2023; Van der Heijden et al., 2002; Hammad et al., 2025).

Limited availability and quality of covariates further constrain inference. Key demographic and clinical factors—most notably age, comorbidity burden, disease severity and duration—cannot be comprehensively adjusted for in routine disproportionality analyses, and age is particularly relevant because both insomnia prevalence and prescribing patterns vary substantially across the life course (Jain and Jain, 2011; Van Gastel, 2018; Doufas et al., 2017; Natter et al., 2021; Bate and Evans, 2009; European Medicines Agency, 2006; Wisniewski et al., 2016; Cutroneo et al., 2023; Van der Heijden et al., 2002). Missingness and misclassification in drug identity, route, reaction coding and reporter fields may also contribute to bias (U.S. Food and Drug Administration, 2018; U.S. Food and Drug Administration, 2014; Bate and Evans, 2009; European Medicines Agency, 2006; Wisniewski et al., 2016; Cutroneo et al., 2023; van der Heijden et al., 2002). Although we applied systemic-route restrictions and parent-drug normalisation, route misclassification remains possible, and fixed-dose combinations were treated as composite entities, so signals for these parents should be interpreted as relating to the combination rather than individual components (Bate and Evans, 2009; European Medicines Agency, 2006; Wisniewski et al., 2016; Cutroneo et al., 2023; Van der Heijden et al., 2002; Hammad et al., 2025). Finally, our study period includes the COVID-19 pandemic, during which changes in healthcare utilisation, prescribing and reporting could have influenced both the composition and volume of FAERS reports; these temporal factors may affect the comparability of signals across time and should be considered when interpreting results (U.S. Food and Drug Administration, 2018; U.S. Food and Drug Administration, 2014; Hammad et al., 2025).

Sex-specific analyses warrant additional caution. Sex-stratified RORs and interaction tests may be influenced by sex differences in exposure prevalence, prescribing indications and reporting behaviour, and therefore do not directly quantify biological susceptibility (Watson et al., 2019; Yu et al., 2016; De Vries et al., 2019; Visser et al., 2021; Zucker et al., 2020). Formal heterogeneity testing also requires sufficient stratum-specific counts; for less frequently reported drugs, power may be limited and estimates may be unstable despite continuity corrections (Evans et al., 2001; Bate and Evans, 2009; European Medicines Agency, 2006; Wisniewski et al., 2016; Cutroneo et al., 2023; Van der Heijden et al., 2002). Consequently, both positive and null findings for sex-related heterogeneity should be interpreted conservatively, and any drug–sex combinations of interest should be evaluated in independent longitudinal datasets with validated sleep outcomes and appropriate confounding control (Bate and Evans, 2009; European Medicines Agency, 2006; Wisniewski et al., 2016; Cutroneo et al., 2023; Van der Heijden et al., 2002; Fusaroli et al., 2024; Hammad et al., 2025).

## Conclusion

Using a large, contemporary FAERS dataset and a reproducible analytical workflow, we identified numerous parent systemic drugs with disproportionate reporting of insomnia and assessed sex-stratified patterns of disproportionality. While many high-ranking signals involved expected sleep-related pharmacology, a substantial number of non-psychotropic drugs also showed elevated disproportionality (Jain and Jain, 2011; Doufas et al., 2017; Natter et al., 2021; Jiang et al., 2024). For most drugs, female and male RORs were broadly similar, with little evidence of robust sex-related heterogeneity after multiple-testing correction. These findings represent disproportional reporting signals rather than causal risk estimates and should be viewed as hypothesis-generating. Validation in cohort studies with detailed adjustment for confounding will be essential to clarify the mechanisms and clinical relevance of drug-associated insomnia and its potential sex-related differences.

## Data Availability

The original contributions presented in the study are included in the article/Supplementary Material, further inquiries can be directed to the corresponding authors.
