# Supplementary material for: Drug-associated insomnia and sex-specific disproportionality in the FDA adverse event reporting system (2019–Q1 2025)
Source: Front Pharmacol. 2026 Feb 23;17:1758403. doi: 10.3389/fphar.2026.1758403 (PMC12968212; doi:10.3389/fphar.2026.1758403)
Supplement: Supplementary file 1 [file Table1.docx]

## Supplementary Tables

## Table S1. Distribution of administration routes among FAERS reports with insomnia recorded as a reaction (2019Q1–2025Q1).

| **Administration route** | **Reports (n)** | **Percent (%)** |
| --- | --- | --- |
| ORAL | 33,502 | 31.9 |
| SUBCUTANEOUS | 13,043 | 12.4 |
| INTRAVENOUS | 7,388 | 7.0 |
| INTRAMUSCULAR | 2,347 | 2.2 |
| OTHER | 1,344 | 1.3 |
| TOPICAL | 1,204 | 1.1 |
| INHALATION | 1,167 | 1.1 |
| TRANSDERMAL | 666 | 0.6 |
| INTRACARDIAC | 630 | 0.6 |
| OPHTHALMIC | 505 | 0.5 |
| BUCCAL | 505 | 0.5 |
| CUTANEOUS | 434 | 0.4 |
| ENDOCERVICAL | 403 | 0.4 |
| VAGINAL | 395 | 0.4 |
| INTRA ARTERIAL | 359 | 0.3 |
| NASAL | 279 | 0.3 |
| INTRA UTERINE | 237 | 0.2 |
| INTRA ARTICULAR | 180 | 0.2 |
| SUBLINGUAL | 171 | 0.2 |
| INTRAVESICAL | 161 | 0.2 |
| PERIARTICULAR | 144 | 0.1 |
| PARENTERAL | 125 | 0.1 |
| SUNCONJUNCTIVAL | 123 | 0.1 |
| TRANSPLACENTAL | 105 | 0.1 |
| INTRATHECAL | 97 | 0.1 |
| URETHRAL | 70 | 0.1 |
| OROPHARINGEAL | 49 | 0.0 |
| INTRAPERITONEAL | 38 | 0.0 |
| RECTAL | 35 | 0.0 |
| SUBDERMAL | 34 | 0.0 |
| INTRAOCULAR | 32 | 0.0 |
| INTRACAVERNOUS | 22 | 0.0 |
| EPIDURAL | 19 | 0.0 |
| OCCLUSIVE DRESSING TECHNIQUE | 14 | 0.0 |
| PERINEURAL | 13 | 0.0 |
| INTRACERVICAL | 12 | 0.0 |
| INTRAPERICARDIAL | 9 | 0.0 |
| EXTRA AMNIOTIC | 6 | 0.0 |
| TRANSMAMMARY | 5 | 0.0 |
| IRRIGATION | 4 | 0.0 |
| INTRADERMAL | 4 | 0.0 |
| SUBCONJUNCTIVAL | 4 | 0.0 |
| INTRATHORACIC | 4 | 0.0 |
| INTRA AURAL | 3 | 0.0 |
| AURICULAR OTIC | 3 | 0.0 |
| INTRASYNOVIAL | 3 | 0.0 |
| INTRACARDIAC USE | 3 | 0.0 |
| INTRALESIONAL | 2 | 0.0 |
| INTRADISCAL INTRASPINAL | 2 | 0.0 |
| DENTAL | 2 | 0.0 |
| INTRAARTERIAL USE | 2 | 0.0 |
| ENDOCERVICAL USE | 2 | 0.0 |
| CUTANEOUS USE | 2 | 0.0 |
| INTRASINAL | 1 | 0.0 |
| INTRACAVITY | 1 | 0.0 |
| INJECTION | 1 | 0.0 |
| INTRACISTERNAL | 1 | 0.0 |
| INTRA CORPUS CAVERNOSUM | 1 | 0.0 |
| RETROBULBAR | 1 | 0.0 |
| BUCCAL USE | 1 | 0.0 |
| VAGINAL USE | 1 | 0.0 |
| OCULAR USE | 1 | 0.0 |
| OROPHARYNGEAL USE | 1 | 0.0 |
| PERINEURAL USE | 1 | 0.0 |
| INTRAMENINGEAL | 1 | 0.0 |
| INTRAVESICAL USE | 1 | 0.0 |
| UNKNOWN | 38,980 | 37.6 |
| Total | 104,905 | 100.0 |

Notes: Route harmonization applied to FAERS DRUG.ROUTE; 'Total' row enforces 100.0% after rounding adjustment.

## Table S2. Full disproportionality results for insomnia (PS analysis; parent systemic drugs).

| **Parent systemic drug** | **a** | **b** | **c** | **d** | **PS reports (a+c)** | **ROR** | **ROR_low** | **ROR_high** | **PRR** | **PRR_low** | **PRR_high** |
| --- | --- | --- | --- | --- | --- | --- | --- | --- | --- | --- | --- |
| oxybate | 61 | 74,383 | 174 | 2,860,942 | 235 | 13.56 | 10.14 | 18.13 | 13.55 | 10.13 | 18.11 |
| istradefylline | 51 | 74,393 | 147 | 2,860,969 | 198 | 13.43 | 9.78 | 18.44 | 13.42 | 9.77 | 18.43 |
| daridorexant | 12 | 74,432 | 42 | 2,861,074 | 54 | 11.31 | 6.02 | 21.24 | 11.30 | 6.02 | 21.24 |
| trofinetide | 92 | 74,352 | 324 | 2,860,792 | 416 | 10.97 | 8.70 | 13.82 | 10.96 | 8.70 | 13.80 |
| niraparib | 437 | 74,007 | 1,633 | 2,859,483 | 2,070 | 10.35 | 9.31 | 11.50 | 10.29 | 9.26 | 11.44 |
| viloxazine | 27 | 74,417 | 104 | 2,861,012 | 131 | 10.12 | 6.65 | 15.40 | 10.11 | 6.65 | 15.39 |
| diphenhydramine naproxen | 28 | 74,416 | 111 | 2,861,005 | 139 | 9.83 | 6.51 | 14.83 | 9.82 | 6.51 | 14.82 |
| loratadine pseudoephedrine | 126 | 74,318 | 508 | 2,860,608 | 634 | 9.58 | 7.88 | 11.64 | 9.56 | 7.87 | 11.61 |
| flibanserin | 112 | 74,332 | 469 | 2,860,647 | 581 | 9.22 | 7.51 | 11.33 | 9.21 | 7.50 | 11.31 |
| guaifenesin pseudoephedrine | 25 | 74,419 | 125 | 2,860,991 | 150 | 7.81 | 5.10 | 11.96 | 7.81 | 5.10 | 11.95 |
| pseudoephedrine | 78 | 74,366 | 387 | 2,860,729 | 465 | 7.79 | 6.11 | 9.93 | 7.79 | 6.11 | 9.92 |
| pimavanserin | 570 | 73,874 | 2,913 | 2,858,203 | 3,483 | 7.58 | 6.92 | 8.29 | 7.53 | 6.88 | 8.23 |
| ziprasidone | 11 | 74,433 | 61 | 2,861,055 | 72 | 7.19 | 3.83 | 13.49 | 7.19 | 3.83 | 13.49 |
| ropeginterferon alfa 2b njft | 31 | 74,413 | 171 | 2,860,945 | 202 | 7.06 | 4.83 | 10.33 | 7.06 | 4.83 | 10.32 |
| suvorexant | 43 | 74,401 | 248 | 2,860,868 | 291 | 6.73 | 4.88 | 9.29 | 6.73 | 4.88 | 9.28 |
| levoketoconazole | 9 | 74,435 | 54 | 2,861,062 | 63 | 6.70 | 3.36 | 13.35 | 6.70 | 3.36 | 13.34 |
| eszopiclone | 36 | 74,408 | 212 | 2,860,904 | 248 | 6.60 | 4.65 | 9.38 | 6.60 | 4.65 | 9.38 |
| cenobamate | 51 | 74,393 | 308 | 2,860,808 | 359 | 6.42 | 4.78 | 8.62 | 6.42 | 4.78 | 8.62 |
| estrogens medroxyprogesterone | 32 | 74,412 | 197 | 2,860,919 | 229 | 6.33 | 4.37 | 9.17 | 6.32 | 4.36 | 9.16 |
| dupilumab | 1,504 | 72,940 | 9,478 | 2,851,638 | 10,982 | 6.21 | 5.87 | 6.56 | 6.10 | 5.78 | 6.44 |
| levothyroxine liothyronine | 43 | 74,401 | 273 | 2,860,843 | 316 | 6.12 | 4.44 | 8.42 | 6.11 | 4.44 | 8.42 |
| dexmethylphenidate serdexmethylphenidate chloride | 16 | 74,428 | 105 | 2,861,011 | 121 | 6.01 | 3.58 | 10.10 | 6.01 | 3.58 | 10.10 |
| bupropion naltrexone | 137 | 74,307 | 890 | 2,860,226 | 1,027 | 5.94 | 4.97 | 7.11 | 5.93 | 4.96 | 7.10 |
| fexofenadine pseudoephedrine | 10 | 74,434 | 68 | 2,861,048 | 78 | 5.89 | 3.08 | 11.28 | 5.89 | 3.08 | 11.28 |
| infliximab dyyb | 98 | 74,346 | 646 | 2,860,470 | 744 | 5.86 | 4.74 | 7.25 | 5.86 | 4.74 | 7.24 |
| finasteride | 177 | 74,267 | 1,204 | 2,859,912 | 1,381 | 5.67 | 4.85 | 6.64 | 5.66 | 4.84 | 6.63 |
| milnacipran | 13 | 74,431 | 91 | 2,861,025 | 104 | 5.67 | 3.20 | 10.04 | 5.67 | 3.20 | 10.04 |
| montelukast | 330 | 74,114 | 2,268 | 2,858,848 | 2,598 | 5.62 | 5.01 | 6.31 | 5.60 | 4.99 | 6.28 |
| lumateperone | 75 | 74,369 | 524 | 2,860,592 | 599 | 5.54 | 4.35 | 7.05 | 5.53 | 4.35 | 7.04 |
| vilazodone | 39 | 74,405 | 278 | 2,860,838 | 317 | 5.45 | 3.91 | 7.61 | 5.45 | 3.91 | 7.61 |
| valbenazine | 172 | 74,272 | 1,266 | 2,859,850 | 1,438 | 5.24 | 4.47 | 6.15 | 5.23 | 4.47 | 6.14 |
| phentermine topiramate | 75 | 74,369 | 564 | 2,860,552 | 639 | 5.14 | 4.05 | 6.54 | 5.14 | 4.04 | 6.53 |
| burosumab twza | 28 | 74,416 | 216 | 2,860,900 | 244 | 5.06 | 3.42 | 7.48 | 5.06 | 3.42 | 7.48 |
| tasimelteon | 10 | 74,434 | 80 | 2,861,036 | 90 | 5.01 | 2.64 | 9.54 | 5.01 | 2.64 | 9.54 |
| lurasidone | 283 | 74,161 | 2,189 | 2,858,927 | 2,472 | 4.99 | 4.41 | 5.65 | 4.98 | 4.40 | 5.63 |
| relugolix | 84 | 74,360 | 657 | 2,860,459 | 741 | 4.94 | 3.94 | 6.20 | 4.94 | 3.94 | 6.19 |
| naltrexone | 348 | 74,096 | 2,827 | 2,858,289 | 3,175 | 4.75 | 4.25 | 5.32 | 4.74 | 4.24 | 5.29 |
| abaloparatide | 184 | 74,260 | 1,496 | 2,859,620 | 1,680 | 4.75 | 4.07 | 5.53 | 4.74 | 4.07 | 5.52 |
| avapritinib | 222 | 74,222 | 1,812 | 2,859,304 | 2,034 | 4.73 | 4.11 | 5.44 | 4.72 | 4.11 | 5.42 |
| elagolix | 82 | 74,362 | 671 | 2,860,445 | 753 | 4.73 | 3.76 | 5.94 | 4.72 | 3.76 | 5.93 |
| olanzapine samidorphan l | 49 | 74,395 | 403 | 2,860,713 | 452 | 4.72 | 3.51 | 6.34 | 4.71 | 3.51 | 6.33 |
| estrogens | 47 | 74,397 | 388 | 2,860,728 | 435 | 4.70 | 3.48 | 6.35 | 4.70 | 3.48 | 6.35 |
| rucaparib | 117 | 74,327 | 964 | 2,860,152 | 1,081 | 4.69 | 3.87 | 5.68 | 4.68 | 3.87 | 5.67 |
| pregabalin | 580 | 73,864 | 4,801 | 2,856,315 | 5,381 | 4.68 | 4.29 | 5.10 | 4.65 | 4.26 | 5.06 |
| cetirizine pseudoephedrine | 13 | 74,431 | 111 | 2,861,005 | 124 | 4.65 | 2.65 | 8.19 | 4.65 | 2.65 | 8.19 |
| corticotropin | 134 | 74,310 | 1,119 | 2,859,997 | 1,253 | 4.62 | 3.87 | 5.53 | 4.62 | 3.86 | 5.52 |
| levothyroxine | 378 | 74,066 | 3,226 | 2,857,890 | 3,604 | 4.53 | 4.07 | 5.04 | 4.51 | 4.05 | 5.01 |
| omalizumab | 744 | 73,700 | 6,375 | 2,854,741 | 7,119 | 4.52 | 4.19 | 4.88 | 4.49 | 4.16 | 4.84 |
| mometasone furoate olopatadine | 6 | 74,438 | 55 | 2,861,061 | 61 | 4.50 | 2.00 | 10.14 | 4.50 | 2.00 | 10.14 |
| carbidopa levodopa | 166 | 74,278 | 1,432 | 2,859,684 | 1,598 | 4.47 | 3.81 | 5.25 | 4.47 | 3.81 | 5.24 |
| mifepristone | 35 | 74,409 | 316 | 2,860,800 | 351 | 4.31 | 3.05 | 6.10 | 4.31 | 3.05 | 6.10 |
| amantadine | 57 | 74,387 | 519 | 2,860,597 | 576 | 4.26 | 3.24 | 5.59 | 4.25 | 3.24 | 5.59 |
| amphetamine aspartate amphetamine dextroamphetamine saccharate dextroamphetamine | 125 | 74,319 | 1,183 | 2,859,933 | 1,308 | 4.08 | 3.39 | 4.91 | 4.08 | 3.39 | 4.90 |
| sofosbuvir | 16 | 74,428 | 157 | 2,860,959 | 173 | 4.03 | 2.42 | 6.69 | 4.03 | 2.42 | 6.69 |
| desvenlafaxine | 63 | 74,381 | 606 | 2,860,510 | 669 | 4.03 | 3.11 | 5.21 | 4.02 | 3.11 | 5.21 |
| estradiol | 118 | 74,326 | 1,141 | 2,859,975 | 1,259 | 3.99 | 3.31 | 4.83 | 3.99 | 3.30 | 4.82 |
| sofosbuvir velpatasvir | 261 | 74,183 | 2,592 | 2,858,524 | 2,853 | 3.89 | 3.42 | 4.41 | 3.88 | 3.41 | 4.40 |
| doxylamine | 18 | 74,426 | 186 | 2,860,930 | 204 | 3.81 | 2.36 | 6.15 | 3.81 | 2.36 | 6.15 |
| fezolinetant | 14 | 74,430 | 146 | 2,860,970 | 160 | 3.80 | 2.22 | 6.53 | 3.80 | 2.22 | 6.52 |
| elacestrant | 57 | 74,387 | 591 | 2,860,525 | 648 | 3.74 | 2.85 | 4.90 | 3.74 | 2.85 | 4.90 |
| bazedoxifene estrogens | 7 | 74,437 | 78 | 2,861,038 | 85 | 3.67 | 1.74 | 7.77 | 3.67 | 1.74 | 7.77 |
| deutetrabenazine | 92 | 74,352 | 969 | 2,860,147 | 1,061 | 3.67 | 2.96 | 4.54 | 3.67 | 2.96 | 4.54 |
| estradiol progesterone | 9 | 74,435 | 102 | 2,861,014 | 111 | 3.56 | 1.83 | 6.93 | 3.56 | 1.83 | 6.92 |
| clonazepam | 145 | 74,299 | 1,631 | 2,859,485 | 1,776 | 3.43 | 2.90 | 4.07 | 3.43 | 2.89 | 4.06 |
| cariprazine | 74 | 74,370 | 836 | 2,860,280 | 910 | 3.43 | 2.70 | 4.34 | 3.42 | 2.70 | 4.34 |
| elexacaftor ivacaftor tezacaftor | 283 | 74,161 | 3,215 | 2,857,901 | 3,498 | 3.40 | 3.01 | 3.84 | 3.39 | 3.00 | 3.83 |
| dalfampridine | 225 | 74,219 | 2,566 | 2,858,550 | 2,791 | 3.38 | 2.95 | 3.88 | 3.38 | 2.95 | 3.87 |
| ruxolitinib | 207 | 74,237 | 2,418 | 2,858,698 | 2,625 | 3.30 | 2.87 | 3.81 | 3.30 | 2.86 | 3.80 |
| ciprofloxacin | 302 | 74,142 | 3,541 | 2,857,575 | 3,843 | 3.29 | 2.93 | 3.70 | 3.28 | 2.92 | 3.69 |
| osilodrostat | 12 | 74,432 | 148 | 2,860,968 | 160 | 3.24 | 1.82 | 5.76 | 3.24 | 1.82 | 5.76 |
| nortriptyline | 13 | 74,431 | 165 | 2,860,951 | 178 | 3.14 | 1.80 | 5.46 | 3.13 | 1.80 | 5.46 |
| hydroxyprogesterone caproate | 69 | 74,375 | 854 | 2,860,262 | 923 | 3.13 | 2.45 | 3.99 | 3.13 | 2.45 | 3.99 |
| naproxen pseudoephedrine | 9 | 74,435 | 117 | 2,860,999 | 126 | 3.11 | 1.60 | 6.02 | 3.11 | 1.60 | 6.02 |
| thyroid porcine | 22 | 74,422 | 278 | 2,860,838 | 300 | 3.11 | 2.02 | 4.77 | 3.10 | 2.02 | 4.77 |
| bupropion dextromethorphan | 25 | 74,419 | 316 | 2,860,800 | 341 | 3.10 | 2.07 | 4.64 | 3.10 | 2.07 | 4.64 |
| hydrocodone | 29 | 74,415 | 367 | 2,860,749 | 396 | 3.09 | 2.12 | 4.49 | 3.09 | 2.12 | 4.49 |
| diphenhydramine ibuprofen | 7 | 74,437 | 93 | 2,861,023 | 100 | 3.08 | 1.47 | 6.49 | 3.08 | 1.47 | 6.49 |
| varenicline | 105 | 74,339 | 1,319 | 2,859,797 | 1,424 | 3.08 | 2.52 | 3.75 | 3.07 | 2.52 | 3.75 |
| lenalidomide | 1,053 | 73,391 | 13,321 | 2,847,795 | 14,374 | 3.07 | 2.88 | 3.27 | 3.04 | 2.86 | 3.23 |
| efgartigimod alfa fcab | 23 | 74,421 | 294 | 2,860,822 | 317 | 3.07 | 2.02 | 4.67 | 3.07 | 2.01 | 4.67 |
| solriamfetol | 12 | 74,432 | 157 | 2,860,959 | 169 | 3.05 | 1.71 | 5.43 | 3.05 | 1.71 | 5.43 |
| ocrelizumab | 543 | 73,901 | 6,921 | 2,854,195 | 7,464 | 3.03 | 2.78 | 3.31 | 3.02 | 2.77 | 3.29 |
| amoxicillin omeprazole rifabutin | 6 | 74,438 | 82 | 2,861,034 | 88 | 3.03 | 1.36 | 6.73 | 3.03 | 1.36 | 6.73 |
| progesterone | 21 | 74,423 | 274 | 2,860,842 | 295 | 3.01 | 1.94 | 4.67 | 3.01 | 1.94 | 4.67 |
| burosumab | 9 | 74,435 | 121 | 2,860,995 | 130 | 3.01 | 1.55 | 5.82 | 3.01 | 1.55 | 5.82 |
| ofatumumab | 278 | 74,166 | 3,577 | 2,857,539 | 3,855 | 3.00 | 2.65 | 3.39 | 2.99 | 2.65 | 3.38 |
| teriflunomide | 309 | 74,135 | 4,003 | 2,857,113 | 4,312 | 2.98 | 2.65 | 3.35 | 2.97 | 2.65 | 3.33 |
| phentermine | 8 | 74,436 | 110 | 2,861,006 | 118 | 2.96 | 1.47 | 5.94 | 2.96 | 1.47 | 5.94 |
| mogamulizumab | 7 | 74,437 | 97 | 2,861,019 | 104 | 2.96 | 1.41 | 6.21 | 2.96 | 1.41 | 6.21 |
| methylphenidate | 183 | 74,261 | 2,441 | 2,858,675 | 2,624 | 2.89 | 2.49 | 3.36 | 2.89 | 2.49 | 3.36 |
| opicapone | 15 | 74,429 | 208 | 2,860,908 | 223 | 2.86 | 1.71 | 4.79 | 2.86 | 1.71 | 4.79 |
| bupropion | 138 | 74,306 | 1,877 | 2,859,239 | 2,015 | 2.84 | 2.39 | 3.37 | 2.84 | 2.39 | 3.37 |
| duloxetine | 160 | 74,284 | 2,184 | 2,858,932 | 2,344 | 2.83 | 2.41 | 3.32 | 2.82 | 2.41 | 3.31 |
| ziprasidone | 11 | 74,433 | 156 | 2,860,960 | 167 | 2.82 | 1.55 | 5.14 | 2.82 | 1.55 | 5.14 |
| cannabidiol | 106 | 74,338 | 1,456 | 2,859,660 | 1,562 | 2.81 | 2.31 | 3.42 | 2.81 | 2.31 | 3.42 |
| amphetamine aspartate amphetamine aspartate amphetamine dextroamphetamine saccharate dextroamphetamine | 10 | 74,434 | 144 | 2,860,972 | 154 | 2.79 | 1.49 | 5.23 | 2.79 | 1.49 | 5.22 |
| acetaminophen hydrocodone | 80 | 74,364 | 1,110 | 2,860,006 | 1,190 | 2.79 | 2.22 | 3.50 | 2.79 | 2.22 | 3.49 |
| apremilast | 410 | 74,034 | 5,706 | 2,855,410 | 6,116 | 2.77 | 2.51 | 3.07 | 2.76 | 2.50 | 3.06 |
| ledipasvir sofosbuvir | 70 | 74,374 | 978 | 2,860,138 | 1,048 | 2.77 | 2.18 | 3.53 | 2.77 | 2.17 | 3.53 |
| roflumilast | 35 | 74,409 | 494 | 2,860,622 | 529 | 2.76 | 1.96 | 3.88 | 2.76 | 1.96 | 3.88 |
| givosiran | 10 | 74,434 | 146 | 2,860,970 | 156 | 2.75 | 1.47 | 5.15 | 2.75 | 1.47 | 5.15 |
| liothyronine | 13 | 74,431 | 189 | 2,860,927 | 202 | 2.74 | 1.58 | 4.76 | 2.74 | 1.58 | 4.76 |
| doconexent icosapent phosphatidyl serine | 6 | 74,438 | 91 | 2,861,025 | 97 | 2.73 | 1.23 | 6.05 | 2.73 | 1.23 | 6.05 |
| sacubitril valsartan | 574 | 73,870 | 8,168 | 2,852,948 | 8,742 | 2.72 | 2.50 | 2.96 | 2.70 | 2.48 | 2.94 |
| ciclesonide | 36 | 74,408 | 519 | 2,860,597 | 555 | 2.70 | 1.93 | 3.78 | 2.70 | 1.93 | 3.78 |
| alemtuzumab | 175 | 74,269 | 2,557 | 2,858,559 | 2,732 | 2.64 | 2.27 | 3.08 | 2.64 | 2.26 | 3.07 |
| dichlorphenamide | 39 | 74,405 | 576 | 2,860,540 | 615 | 2.63 | 1.91 | 3.64 | 2.63 | 1.91 | 3.63 |
| selexipag | 257 | 74,187 | 3,782 | 2,857,334 | 4,039 | 2.62 | 2.31 | 2.98 | 2.62 | 2.31 | 2.97 |
| umbralisib | 6 | 74,438 | 95 | 2,861,021 | 101 | 2.62 | 1.18 | 5.79 | 2.62 | 1.18 | 5.79 |
| nirmatrelvir ritonavir | 181 | 74,263 | 2,676 | 2,858,440 | 2,857 | 2.61 | 2.25 | 3.03 | 2.61 | 2.24 | 3.03 |
| setmelanotide | 5 | 74,439 | 81 | 2,861,035 | 86 | 2.59 | 1.09 | 6.15 | 2.59 | 1.09 | 6.15 |
| dextromethorphan guaifenesin | 48 | 74,396 | 720 | 2,860,396 | 768 | 2.59 | 1.93 | 3.46 | 2.59 | 1.93 | 3.46 |
| adalimumab aaty | 9 | 74,435 | 141 | 2,860,975 | 150 | 2.58 | 1.34 | 4.98 | 2.58 | 1.34 | 4.98 |
| levofloxacin | 191 | 74,253 | 2,867 | 2,858,249 | 3,058 | 2.57 | 2.22 | 2.98 | 2.57 | 2.22 | 2.97 |
| mitapivat | 10 | 74,434 | 157 | 2,860,959 | 167 | 2.56 | 1.37 | 4.79 | 2.56 | 1.37 | 4.79 |
| atovaquone proguanil | 7 | 74,437 | 112 | 2,861,004 | 119 | 2.56 | 1.22 | 5.37 | 2.56 | 1.22 | 5.37 |
| selegiline | 7 | 74,437 | 112 | 2,861,004 | 119 | 2.56 | 1.22 | 5.37 | 2.56 | 1.22 | 5.37 |
| pirfenidone | 219 | 74,225 | 3,319 | 2,857,797 | 3,538 | 2.55 | 2.22 | 2.92 | 2.54 | 2.22 | 2.91 |
| nirogacestat | 10 | 74,434 | 159 | 2,860,957 | 169 | 2.53 | 1.36 | 4.72 | 2.53 | 1.36 | 4.72 |
| tivozanib | 6 | 74,438 | 99 | 2,861,017 | 105 | 2.51 | 1.14 | 5.55 | 2.51 | 1.14 | 5.55 |
| abacavir dolutegravir lamivudine | 33 | 74,411 | 514 | 2,860,602 | 547 | 2.50 | 1.76 | 3.55 | 2.50 | 1.76 | 3.55 |
| tofacitinib | 584 | 73,860 | 9,067 | 2,852,049 | 9,651 | 2.49 | 2.29 | 2.71 | 2.48 | 2.28 | 2.69 |
| fenfluramine | 41 | 74,403 | 641 | 2,860,475 | 682 | 2.49 | 1.82 | 3.40 | 2.49 | 1.82 | 3.40 |
| vamorolone | 5 | 74,439 | 85 | 2,861,031 | 90 | 2.47 | 1.04 | 5.86 | 2.47 | 1.04 | 5.86 |
| fexofenadine | 40 | 74,404 | 634 | 2,860,482 | 674 | 2.45 | 1.79 | 3.37 | 2.45 | 1.79 | 3.37 |
| colestipol | 5 | 74,439 | 86 | 2,861,030 | 91 | 2.44 | 1.03 | 5.79 | 2.44 | 1.03 | 5.79 |
| deflazacort | 16 | 74,428 | 260 | 2,860,856 | 276 | 2.43 | 1.48 | 4.00 | 2.43 | 1.48 | 4.00 |
| lemborexant | 8 | 74,436 | 134 | 2,860,982 | 142 | 2.43 | 1.21 | 4.86 | 2.43 | 1.21 | 4.86 |
| atomoxetine | 25 | 74,419 | 404 | 2,860,712 | 429 | 2.42 | 1.62 | 3.62 | 2.42 | 1.62 | 3.61 |
| escitalopram | 148 | 74,296 | 2,358 | 2,858,758 | 2,506 | 2.42 | 2.05 | 2.86 | 2.42 | 2.05 | 2.86 |
| amphetamine | 11 | 74,433 | 182 | 2,860,934 | 193 | 2.42 | 1.33 | 4.40 | 2.42 | 1.33 | 4.39 |
| atogepant | 42 | 74,402 | 675 | 2,860,441 | 717 | 2.42 | 1.77 | 3.30 | 2.42 | 1.77 | 3.30 |
| treprostinil | 261 | 74,183 | 4,167 | 2,856,949 | 4,428 | 2.42 | 2.13 | 2.74 | 2.41 | 2.13 | 2.73 |
| palbociclib | 361 | 74,083 | 5,786 | 2,855,330 | 6,147 | 2.41 | 2.16 | 2.68 | 2.40 | 2.16 | 2.67 |
| clarithromycin | 73 | 74,371 | 1,177 | 2,859,939 | 1,250 | 2.40 | 1.90 | 3.04 | 2.40 | 1.90 | 3.04 |
| leflunomide | 63 | 74,381 | 1,022 | 2,860,094 | 1,085 | 2.39 | 1.85 | 3.08 | 2.39 | 1.85 | 3.08 |
| cetirizine | 126 | 74,318 | 2,044 | 2,859,072 | 2,170 | 2.38 | 1.99 | 2.85 | 2.38 | 1.99 | 2.85 |
| herbals mitragynine | 7 | 74,437 | 122 | 2,860,994 | 129 | 2.35 | 1.13 | 4.92 | 2.35 | 1.13 | 4.92 |
| pacritinib | 27 | 74,417 | 449 | 2,860,667 | 476 | 2.35 | 1.60 | 3.46 | 2.35 | 1.60 | 3.46 |
| tirzepatide | 114 | 74,330 | 1,881 | 2,859,235 | 1,995 | 2.34 | 1.94 | 2.83 | 2.34 | 1.94 | 2.82 |
| galcanezumab gnlm | 81 | 74,363 | 1,343 | 2,859,773 | 1,424 | 2.33 | 1.87 | 2.92 | 2.33 | 1.86 | 2.92 |
| gabapentin | 163 | 74,281 | 2,701 | 2,858,415 | 2,864 | 2.33 | 1.99 | 2.73 | 2.33 | 1.99 | 2.72 |
| pyrimethamine | 10 | 74,434 | 173 | 2,860,943 | 183 | 2.33 | 1.25 | 4.34 | 2.33 | 1.25 | 4.34 |
| oxycodone | 377 | 74,067 | 6,259 | 2,854,857 | 6,636 | 2.32 | 2.09 | 2.58 | 2.32 | 2.09 | 2.57 |
| aripiprazole | 192 | 74,252 | 3,210 | 2,857,906 | 3,402 | 2.31 | 2.00 | 2.67 | 2.30 | 1.99 | 2.66 |
| chlorhexidine | 12 | 74,432 | 208 | 2,860,908 | 220 | 2.30 | 1.30 | 4.08 | 2.30 | 1.30 | 4.08 |
| enzalutamide | 178 | 74,266 | 2,987 | 2,858,129 | 3,165 | 2.30 | 1.98 | 2.67 | 2.30 | 1.97 | 2.67 |
| alendronate | 97 | 74,347 | 1,634 | 2,859,482 | 1,731 | 2.29 | 1.87 | 2.81 | 2.29 | 1.87 | 2.81 |
| tesamorelin | 18 | 74,426 | 310 | 2,860,806 | 328 | 2.29 | 1.43 | 3.66 | 2.29 | 1.43 | 3.66 |
| trospium chloride xanomeline | 6 | 74,438 | 109 | 2,861,007 | 115 | 2.28 | 1.03 | 5.03 | 2.28 | 1.03 | 5.03 |
| citalopram | 91 | 74,353 | 1,548 | 2,859,568 | 1,639 | 2.27 | 1.84 | 2.81 | 2.27 | 1.84 | 2.80 |
| sertraline | 228 | 74,216 | 3,873 | 2,857,243 | 4,101 | 2.27 | 1.99 | 2.60 | 2.27 | 1.98 | 2.59 |
| nebivolol | 25 | 74,419 | 435 | 2,860,681 | 460 | 2.25 | 1.51 | 3.36 | 2.25 | 1.51 | 3.35 |
| triamcinolone acetonide | 18 | 74,426 | 318 | 2,860,798 | 336 | 2.23 | 1.40 | 3.57 | 2.23 | 1.40 | 3.57 |
| belimumab | 132 | 74,312 | 2,304 | 2,858,812 | 2,436 | 2.21 | 1.86 | 2.64 | 2.21 | 1.86 | 2.63 |
| pexidartinib | 21 | 74,423 | 376 | 2,860,740 | 397 | 2.20 | 1.42 | 3.39 | 2.19 | 1.42 | 3.39 |
| patisiran | 34 | 74,410 | 604 | 2,860,512 | 638 | 2.19 | 1.56 | 3.09 | 2.19 | 1.56 | 3.09 |
| doxepin | 9 | 74,435 | 166 | 2,860,950 | 175 | 2.19 | 1.14 | 4.22 | 2.19 | 1.14 | 4.22 |
| maralixibat chloride | 9 | 74,435 | 166 | 2,860,950 | 175 | 2.19 | 1.14 | 4.22 | 2.19 | 1.14 | 4.22 |
| stiripentol | 14 | 74,430 | 254 | 2,860,862 | 268 | 2.19 | 1.29 | 3.72 | 2.19 | 1.29 | 3.72 |
| fingolimod | 431 | 74,013 | 7,657 | 2,853,459 | 8,088 | 2.17 | 1.97 | 2.39 | 2.17 | 1.97 | 2.39 |
| efavirenz | 9 | 74,435 | 168 | 2,860,948 | 177 | 2.17 | 1.13 | 4.17 | 2.17 | 1.13 | 4.17 |
| nicotine | 55 | 74,389 | 985 | 2,860,131 | 1,040 | 2.17 | 1.65 | 2.84 | 2.16 | 1.65 | 2.84 |
| venlafaxine | 157 | 74,287 | 2,837 | 2,858,279 | 2,994 | 2.14 | 1.82 | 2.51 | 2.13 | 1.82 | 2.50 |
| lisdexamfetamine dimesylate | 58 | 74,386 | 1,053 | 2,860,063 | 1,111 | 2.14 | 1.64 | 2.78 | 2.13 | 1.64 | 2.78 |
| water | 12 | 74,432 | 225 | 2,860,891 | 237 | 2.13 | 1.21 | 3.77 | 2.13 | 1.21 | 3.77 |
| oseltamivir | 54 | 74,390 | 988 | 2,860,128 | 1,042 | 2.12 | 1.61 | 2.78 | 2.12 | 1.61 | 2.78 |
| guaifenesin | 26 | 74,418 | 481 | 2,860,635 | 507 | 2.12 | 1.43 | 3.13 | 2.12 | 1.43 | 3.13 |
| vortioxetine | 73 | 74,371 | 1,342 | 2,859,774 | 1,415 | 2.11 | 1.66 | 2.66 | 2.10 | 1.66 | 2.66 |
| vibegron | 20 | 74,424 | 374 | 2,860,742 | 394 | 2.10 | 1.35 | 3.28 | 2.10 | 1.35 | 3.28 |
| ofloxacin | 9 | 74,435 | 174 | 2,860,942 | 183 | 2.09 | 1.09 | 4.02 | 2.09 | 1.09 | 4.02 |
| budesonide | 186 | 74,258 | 3,433 | 2,857,683 | 3,619 | 2.09 | 1.80 | 2.42 | 2.09 | 1.80 | 2.42 |
| hydromorphone | 52 | 74,392 | 966 | 2,860,150 | 1,018 | 2.09 | 1.58 | 2.76 | 2.09 | 1.58 | 2.76 |
| teprotumumab trbw | 37 | 74,407 | 693 | 2,860,423 | 730 | 2.08 | 1.50 | 2.89 | 2.08 | 1.50 | 2.89 |
| ripretinib | 54 | 74,390 | 1,012 | 2,860,104 | 1,066 | 2.07 | 1.58 | 2.72 | 2.07 | 1.58 | 2.72 |
| amphetamine dextroamphetamine | 6 | 74,438 | 122 | 2,860,994 | 128 | 2.04 | 0.93 | 4.49 | 2.04 | 0.93 | 4.49 |
| voclosporin | 57 | 74,387 | 1,092 | 2,860,024 | 1,149 | 2.02 | 1.55 | 2.64 | 2.02 | 1.55 | 2.64 |
| tazemetostat | 19 | 74,425 | 370 | 2,860,746 | 389 | 2.02 | 1.28 | 3.19 | 2.02 | 1.28 | 3.19 |
| dexmethylphenidate | 8 | 74,436 | 161 | 2,860,955 | 169 | 2.02 | 1.01 | 4.03 | 2.02 | 1.01 | 4.03 |
| lasmiditan | 5 | 74,439 | 105 | 2,861,011 | 110 | 2.00 | 0.85 | 4.72 | 2.00 | 0.85 | 4.72 |
| anastrozole | 64 | 74,380 | 1,247 | 2,859,869 | 1,311 | 1.99 | 1.55 | 2.55 | 1.99 | 1.55 | 2.55 |
| oxybate | 27 | 74,417 | 534 | 2,860,582 | 561 | 1.98 | 1.35 | 2.90 | 1.98 | 1.35 | 2.90 |
| asenapine | 5 | 74,439 | 107 | 2,861,009 | 112 | 1.97 | 0.83 | 4.63 | 1.97 | 0.83 | 4.63 |
| metoclopramide | 17 | 74,427 | 342 | 2,860,774 | 359 | 1.96 | 1.21 | 3.18 | 1.96 | 1.21 | 3.17 |
| modafinil | 6 | 74,438 | 127 | 2,860,989 | 133 | 1.96 | 0.89 | 4.31 | 1.96 | 0.89 | 4.31 |
| telotristat ethyl | 21 | 74,423 | 424 | 2,860,692 | 445 | 1.95 | 1.26 | 3.00 | 1.95 | 1.26 | 3.00 |
| buprenorphine | 99 | 74,345 | 1,980 | 2,859,136 | 2,079 | 1.93 | 1.58 | 2.36 | 1.93 | 1.58 | 2.36 |
| propranolol | 60 | 74,384 | 1,211 | 2,859,905 | 1,271 | 1.92 | 1.48 | 2.49 | 1.92 | 1.48 | 2.48 |
| rosuvastatin | 164 | 74,280 | 3,300 | 2,857,816 | 3,464 | 1.92 | 1.64 | 2.24 | 1.92 | 1.64 | 2.24 |
| doravirine lamivudine tenofovir disoproxil | 5 | 74,439 | 110 | 2,861,006 | 115 | 1.91 | 0.81 | 4.50 | 1.91 | 0.81 | 4.50 |
| loratadine | 96 | 74,348 | 1,940 | 2,859,176 | 2,036 | 1.91 | 1.56 | 2.35 | 1.91 | 1.56 | 2.34 |
| erenumab aooe | 92 | 74,352 | 1,880 | 2,859,236 | 1,972 | 1.89 | 1.54 | 2.33 | 1.89 | 1.53 | 2.33 |
| eslicarbazepine | 34 | 74,410 | 704 | 2,860,412 | 738 | 1.88 | 1.34 | 2.65 | 1.88 | 1.34 | 2.65 |
| bismuth subcitrate metronidazole tetracycline | 7 | 74,437 | 153 | 2,860,963 | 160 | 1.88 | 0.90 | 3.91 | 1.88 | 0.90 | 3.91 |
| tamoxifen | 30 | 74,414 | 633 | 2,860,483 | 663 | 1.85 | 1.29 | 2.66 | 1.85 | 1.29 | 2.66 |
| bremelanotide | 5 | 74,439 | 114 | 2,861,002 | 119 | 1.85 | 0.78 | 4.34 | 1.85 | 0.78 | 4.34 |
| paliperidone palmitate | 237 | 74,207 | 4,988 | 2,856,128 | 5,225 | 1.83 | 1.61 | 2.09 | 1.83 | 1.61 | 2.08 |
| nintedanib | 214 | 74,230 | 4,509 | 2,856,607 | 4,723 | 1.83 | 1.60 | 2.10 | 1.83 | 1.59 | 2.10 |
| acetaminophen oxycodone | 43 | 74,401 | 914 | 2,860,202 | 957 | 1.83 | 1.35 | 2.48 | 1.83 | 1.35 | 2.48 |
| fluvoxamine | 8 | 74,436 | 179 | 2,860,937 | 187 | 1.82 | 0.91 | 3.62 | 1.82 | 0.91 | 3.62 |
| brexpiprazole | 31 | 74,413 | 668 | 2,860,448 | 699 | 1.81 | 1.27 | 2.59 | 1.81 | 1.27 | 2.59 |
| ombitasvir paritaprevir ritonavir | 7 | 74,437 | 159 | 2,860,957 | 166 | 1.81 | 0.87 | 3.76 | 1.81 | 0.87 | 3.76 |
| quetiapine | 240 | 74,204 | 5,147 | 2,855,969 | 5,387 | 1.80 | 1.58 | 2.05 | 1.80 | 1.58 | 2.04 |
| sucralfate | 7 | 74,437 | 160 | 2,860,956 | 167 | 1.80 | 0.86 | 3.74 | 1.80 | 0.86 | 3.73 |
| octreotide | 198 | 74,246 | 4,267 | 2,856,849 | 4,465 | 1.79 | 1.55 | 2.06 | 1.79 | 1.55 | 2.06 |
| sparsentan | 30 | 74,414 | 656 | 2,860,460 | 686 | 1.79 | 1.24 | 2.57 | 1.79 | 1.24 | 2.57 |
| cyclobenzaprine | 10 | 74,434 | 228 | 2,860,888 | 238 | 1.77 | 0.95 | 3.28 | 1.77 | 0.95 | 3.28 |
| adalimumab | 1,305 | 73,139 | 28,672 | 2,832,444 | 29,977 | 1.76 | 1.67 | 1.86 | 1.75 | 1.66 | 1.85 |
| ethinyl estradiol norethindrone | 16 | 74,428 | 360 | 2,860,756 | 376 | 1.76 | 1.07 | 2.88 | 1.76 | 1.07 | 2.88 |
| budesonide formoterol dihydrate | 163 | 74,281 | 3,608 | 2,857,508 | 3,771 | 1.74 | 1.49 | 2.04 | 1.74 | 1.49 | 2.04 |
| rimegepant | 33 | 74,411 | 740 | 2,860,376 | 773 | 1.74 | 1.23 | 2.46 | 1.74 | 1.23 | 2.46 |
| macitentan | 317 | 74,127 | 7,054 | 2,854,062 | 7,371 | 1.73 | 1.55 | 1.94 | 1.73 | 1.55 | 1.94 |
| drospirenone | 15 | 74,429 | 344 | 2,860,772 | 359 | 1.73 | 1.04 | 2.88 | 1.73 | 1.04 | 2.88 |
| paroxetine | 63 | 74,381 | 1,413 | 2,859,703 | 1,476 | 1.73 | 1.34 | 2.22 | 1.73 | 1.34 | 2.22 |
| buspirone | 10 | 74,434 | 234 | 2,860,882 | 244 | 1.72 | 0.93 | 3.19 | 1.72 | 0.93 | 3.19 |
| triazolam | 6 | 74,438 | 146 | 2,860,970 | 152 | 1.71 | 0.78 | 3.74 | 1.71 | 0.78 | 3.74 |
| adalimumab afzb | 31 | 74,413 | 710 | 2,860,406 | 741 | 1.70 | 1.19 | 2.44 | 1.70 | 1.19 | 2.43 |
| amifampridine | 42 | 74,402 | 963 | 2,860,153 | 1,005 | 1.70 | 1.25 | 2.31 | 1.70 | 1.25 | 2.30 |
| amitriptyline | 21 | 74,423 | 488 | 2,860,628 | 509 | 1.69 | 1.10 | 2.61 | 1.69 | 1.10 | 2.61 |
| sofosbuvir velpatasvir voxilaprevir | 12 | 74,432 | 284 | 2,860,832 | 296 | 1.69 | 0.96 | 2.98 | 1.69 | 0.96 | 2.98 |
| pomalidomide | 275 | 74,169 | 6,311 | 2,854,805 | 6,586 | 1.68 | 1.49 | 1.90 | 1.68 | 1.49 | 1.89 |
| mepolizumab | 123 | 74,321 | 2,835 | 2,858,281 | 2,958 | 1.68 | 1.40 | 2.01 | 1.67 | 1.40 | 2.00 |
| semaglutide | 273 | 74,171 | 6,311 | 2,854,805 | 6,584 | 1.67 | 1.48 | 1.88 | 1.67 | 1.48 | 1.88 |
| esketamine | 26 | 74,418 | 614 | 2,860,502 | 640 | 1.66 | 1.12 | 2.45 | 1.66 | 1.12 | 2.44 |
| fremanezumab vfrm | 41 | 74,403 | 967 | 2,860,149 | 1,008 | 1.65 | 1.21 | 2.25 | 1.65 | 1.21 | 2.25 |
| inotersen | 30 | 74,414 | 711 | 2,860,405 | 741 | 1.65 | 1.15 | 2.37 | 1.65 | 1.15 | 2.37 |
| secukinumab | 510 | 73,934 | 12,077 | 2,849,039 | 12,587 | 1.63 | 1.49 | 1.78 | 1.62 | 1.49 | 1.77 |
| lorcaserin | 5 | 74,439 | 131 | 2,860,985 | 136 | 1.61 | 0.68 | 3.77 | 1.61 | 0.68 | 3.77 |
| mirtazapine | 82 | 74,362 | 1,976 | 2,859,140 | 2,058 | 1.60 | 1.29 | 2.00 | 1.60 | 1.29 | 2.00 |
| ribociclib | 208 | 74,236 | 5,014 | 2,856,102 | 5,222 | 1.60 | 1.39 | 1.84 | 1.60 | 1.39 | 1.84 |
| procarbazine | 9 | 74,435 | 228 | 2,860,888 | 237 | 1.60 | 0.84 | 3.06 | 1.60 | 0.84 | 3.06 |
| dextroamphetamine | 9 | 74,435 | 229 | 2,860,887 | 238 | 1.59 | 0.83 | 3.04 | 1.59 | 0.83 | 3.04 |
| inclisiran | 51 | 74,393 | 1,248 | 2,859,868 | 1,299 | 1.59 | 1.20 | 2.10 | 1.59 | 1.20 | 2.09 |
| cobicistat elvitegravir emtricitabine tenofovir | 10 | 74,434 | 256 | 2,860,860 | 266 | 1.57 | 0.85 | 2.92 | 1.57 | 0.85 | 2.92 |
| brexanolone | 5 | 74,439 | 134 | 2,860,982 | 139 | 1.57 | 0.67 | 3.69 | 1.57 | 0.67 | 3.69 |
| leuprolide | 275 | 74,169 | 6,793 | 2,854,323 | 7,068 | 1.56 | 1.38 | 1.76 | 1.56 | 1.38 | 1.76 |
| dextromethorphan | 19 | 74,425 | 480 | 2,860,636 | 499 | 1.56 | 0.99 | 2.45 | 1.56 | 0.99 | 2.45 |
| estradiol norethindrone | 27 | 74,417 | 683 | 2,860,433 | 710 | 1.55 | 1.06 | 2.26 | 1.55 | 1.06 | 2.26 |
| moxifloxacin | 45 | 74,399 | 1,134 | 2,859,982 | 1,179 | 1.54 | 1.15 | 2.07 | 1.54 | 1.15 | 2.07 |
| fluticasone propionate | 50 | 74,394 | 1,260 | 2,859,856 | 1,310 | 1.54 | 1.16 | 2.04 | 1.54 | 1.16 | 2.04 |
| trazodone | 15 | 74,429 | 388 | 2,860,728 | 403 | 1.53 | 0.92 | 2.55 | 1.53 | 0.92 | 2.55 |
| risedronate | 16 | 74,428 | 415 | 2,860,701 | 431 | 1.53 | 0.93 | 2.50 | 1.53 | 0.93 | 2.50 |
| erenumab | 7 | 74,437 | 189 | 2,860,927 | 196 | 1.52 | 0.73 | 3.16 | 1.52 | 0.73 | 3.16 |
| diroximel | 21 | 74,423 | 543 | 2,860,573 | 564 | 1.52 | 0.99 | 2.34 | 1.52 | 0.99 | 2.34 |
| tolterodine | 6 | 74,438 | 164 | 2,860,952 | 170 | 1.52 | 0.69 | 3.33 | 1.52 | 0.69 | 3.33 |
| ozanimod | 89 | 74,355 | 2,272 | 2,858,844 | 2,361 | 1.51 | 1.23 | 1.87 | 1.51 | 1.23 | 1.87 |
| icatibant | 52 | 74,392 | 1,335 | 2,859,781 | 1,387 | 1.51 | 1.15 | 1.99 | 1.51 | 1.15 | 1.99 |
| neratinib | 10 | 74,434 | 267 | 2,860,849 | 277 | 1.51 | 0.81 | 2.80 | 1.51 | 0.81 | 2.79 |
| paliperidone | 45 | 74,399 | 1,174 | 2,859,942 | 1,219 | 1.49 | 1.11 | 2.00 | 1.49 | 1.11 | 2.00 |
| pramipexole | 8 | 74,436 | 219 | 2,860,897 | 227 | 1.49 | 0.75 | 2.95 | 1.49 | 0.75 | 2.95 |
| brivaracetam | 33 | 74,411 | 865 | 2,860,251 | 898 | 1.49 | 1.05 | 2.10 | 1.49 | 1.05 | 2.10 |
| diclofenac | 7 | 74,437 | 194 | 2,860,922 | 201 | 1.48 | 0.71 | 3.07 | 1.48 | 0.71 | 3.07 |
| clofazimine | 17 | 74,427 | 456 | 2,860,660 | 473 | 1.47 | 0.91 | 2.38 | 1.47 | 0.91 | 2.37 |
| aclidinium bromide | 17 | 74,427 | 457 | 2,860,659 | 474 | 1.47 | 0.91 | 2.37 | 1.47 | 0.91 | 2.37 |
| famotidine | 21 | 74,423 | 562 | 2,860,554 | 583 | 1.47 | 0.95 | 2.26 | 1.47 | 0.95 | 2.26 |
| dronabinol | 5 | 74,439 | 145 | 2,860,971 | 150 | 1.45 | 0.62 | 3.40 | 1.45 | 0.62 | 3.40 |
| morphine | 62 | 74,382 | 1,656 | 2,859,460 | 1,718 | 1.45 | 1.13 | 1.87 | 1.45 | 1.13 | 1.87 |
| caplacizumab yhdp | 24 | 74,420 | 650 | 2,860,466 | 674 | 1.45 | 0.97 | 2.17 | 1.45 | 0.97 | 2.17 |
| dimethyl | 153 | 74,291 | 4,079 | 2,857,037 | 4,232 | 1.45 | 1.23 | 1.70 | 1.45 | 1.23 | 1.70 |
| guanfacine | 19 | 74,425 | 518 | 2,860,598 | 537 | 1.45 | 0.92 | 2.27 | 1.45 | 0.92 | 2.27 |
| adalimumab fkjp | 12 | 74,432 | 332 | 2,860,784 | 344 | 1.44 | 0.82 | 2.54 | 1.44 | 0.82 | 2.54 |
| taliglucerase alfa | 5 | 74,439 | 146 | 2,860,970 | 151 | 1.44 | 0.62 | 3.38 | 1.44 | 0.62 | 3.38 |
| selinexor | 56 | 74,388 | 1,506 | 2,859,610 | 1,562 | 1.44 | 1.11 | 1.88 | 1.44 | 1.11 | 1.88 |
| rituximab abbs | 16 | 74,428 | 440 | 2,860,676 | 456 | 1.44 | 0.88 | 2.35 | 1.44 | 0.88 | 2.35 |
| levonorgestrel | 19 | 74,425 | 521 | 2,860,595 | 540 | 1.44 | 0.91 | 2.26 | 1.44 | 0.91 | 2.26 |
| vonoprazan | 14 | 74,430 | 390 | 2,860,726 | 404 | 1.43 | 0.84 | 2.41 | 1.43 | 0.84 | 2.41 |
| epoprostenol | 69 | 74,375 | 1,881 | 2,859,235 | 1,950 | 1.42 | 1.12 | 1.80 | 1.42 | 1.12 | 1.80 |
| fluoxetine | 62 | 74,382 | 1,694 | 2,859,422 | 1,756 | 1.42 | 1.10 | 1.83 | 1.42 | 1.10 | 1.82 |
| ublituximab | 8 | 74,436 | 230 | 2,860,886 | 238 | 1.42 | 0.71 | 2.81 | 1.42 | 0.71 | 2.81 |
| cefdinir | 7 | 74,437 | 203 | 2,860,913 | 210 | 1.42 | 0.68 | 2.94 | 1.42 | 0.68 | 2.94 |
| maribavir | 11 | 74,433 | 313 | 2,860,803 | 324 | 1.41 | 0.78 | 2.54 | 1.41 | 0.78 | 2.54 |
| pegcetacoplan | 6 | 74,438 | 177 | 2,860,939 | 183 | 1.41 | 0.64 | 3.08 | 1.41 | 0.64 | 3.08 |
| adalimumab adaz | 59 | 74,385 | 1,630 | 2,859,486 | 1,689 | 1.40 | 1.08 | 1.82 | 1.40 | 1.08 | 1.82 |
| obeticholic acid | 47 | 74,397 | 1,302 | 2,859,814 | 1,349 | 1.40 | 1.05 | 1.87 | 1.40 | 1.05 | 1.87 |
| fluticasone furoate umeclidinium bromide vilanterol trifenatate | 50 | 74,394 | 1,391 | 2,859,725 | 1,441 | 1.40 | 1.05 | 1.85 | 1.39 | 1.05 | 1.85 |
| fentanyl | 90 | 74,354 | 2,541 | 2,858,575 | 2,631 | 1.37 | 1.11 | 1.69 | 1.37 | 1.11 | 1.69 |
| aripiprazole lauroxil | 38 | 74,406 | 1,081 | 2,860,035 | 1,119 | 1.37 | 0.99 | 1.89 | 1.37 | 0.99 | 1.89 |
| alendronic acid | 14 | 74,430 | 409 | 2,860,707 | 423 | 1.36 | 0.81 | 2.30 | 1.36 | 0.81 | 2.30 |
| topiramate | 80 | 74,364 | 2,281 | 2,858,835 | 2,361 | 1.36 | 1.09 | 1.69 | 1.36 | 1.09 | 1.69 |
| budesonide formoterol | 27 | 74,417 | 780 | 2,860,336 | 807 | 1.35 | 0.93 | 1.98 | 1.35 | 0.93 | 1.98 |
| pitavastatin | 5 | 74,439 | 156 | 2,860,960 | 161 | 1.35 | 0.58 | 3.16 | 1.35 | 0.58 | 3.16 |
| asfotase alfa | 16 | 74,428 | 470 | 2,860,646 | 486 | 1.35 | 0.82 | 2.20 | 1.35 | 0.82 | 2.20 |
| metformin vildagliptin | 26 | 74,418 | 757 | 2,860,359 | 783 | 1.34 | 0.91 | 1.98 | 1.34 | 0.91 | 1.98 |
| glycopyrrolate | 23 | 74,421 | 675 | 2,860,441 | 698 | 1.34 | 0.89 | 2.02 | 1.34 | 0.89 | 2.02 |
| nitazoxanide | 5 | 74,439 | 158 | 2,860,958 | 163 | 1.33 | 0.57 | 3.12 | 1.33 | 0.57 | 3.12 |
| dolutegravir lamivudine | 10 | 74,434 | 303 | 2,860,813 | 313 | 1.33 | 0.72 | 2.46 | 1.33 | 0.72 | 2.46 |
| glecaprevir pibrentasvir | 98 | 74,346 | 2,862 | 2,858,254 | 2,960 | 1.32 | 1.08 | 1.62 | 1.32 | 1.08 | 1.62 |
| darolutamide | 17 | 74,427 | 514 | 2,860,602 | 531 | 1.31 | 0.81 | 2.11 | 1.31 | 0.81 | 2.10 |
| prednisolone | 78 | 74,366 | 2,308 | 2,858,808 | 2,386 | 1.31 | 1.04 | 1.64 | 1.31 | 1.04 | 1.64 |
| fluticasone furoate vilanterol trifenatate | 42 | 74,402 | 1,255 | 2,859,861 | 1,297 | 1.30 | 0.96 | 1.77 | 1.30 | 0.96 | 1.77 |
| bempedoic acid | 10 | 74,434 | 310 | 2,860,806 | 320 | 1.30 | 0.70 | 2.40 | 1.30 | 0.70 | 2.40 |
| nitrofurantoin | 5 | 74,439 | 163 | 2,860,953 | 168 | 1.29 | 0.55 | 3.02 | 1.29 | 0.55 | 3.02 |
| avacopan | 34 | 74,410 | 1,027 | 2,860,089 | 1,061 | 1.29 | 0.92 | 1.81 | 1.29 | 0.92 | 1.81 |
| vigabatrin | 46 | 74,398 | 1,386 | 2,859,730 | 1,432 | 1.29 | 0.96 | 1.73 | 1.29 | 0.96 | 1.73 |
| exemestane | 27 | 74,417 | 820 | 2,860,296 | 847 | 1.29 | 0.88 | 1.88 | 1.29 | 0.88 | 1.88 |
| dolutegravir rilpivirine | 6 | 74,438 | 194 | 2,860,922 | 200 | 1.28 | 0.59 | 2.81 | 1.28 | 0.59 | 2.81 |
| eptinezumab jjmr | 61 | 74,383 | 1,853 | 2,859,263 | 1,914 | 1.28 | 0.99 | 1.64 | 1.28 | 0.99 | 1.64 |
| oxybutynin | 6 | 74,438 | 196 | 2,860,920 | 202 | 1.27 | 0.58 | 2.78 | 1.27 | 0.58 | 2.78 |
| tapentadol | 17 | 74,427 | 529 | 2,860,587 | 546 | 1.27 | 0.79 | 2.05 | 1.27 | 0.79 | 2.04 |
| alprazolam | 79 | 74,365 | 2,419 | 2,858,697 | 2,498 | 1.26 | 1.01 | 1.58 | 1.26 | 1.01 | 1.58 |
| azelastine fluticasone propionate | 5 | 74,439 | 167 | 2,860,949 | 172 | 1.26 | 0.54 | 2.95 | 1.26 | 0.54 | 2.95 |
| emtricitabine rilpivirine tenofovir alafenamide | 9 | 74,435 | 290 | 2,860,826 | 299 | 1.26 | 0.66 | 2.40 | 1.26 | 0.66 | 2.40 |
| rifaximin | 66 | 74,378 | 2,036 | 2,859,080 | 2,102 | 1.26 | 0.98 | 1.60 | 1.25 | 0.98 | 1.60 |
| prucalopride | 5 | 74,439 | 168 | 2,860,948 | 173 | 1.25 | 0.54 | 2.93 | 1.25 | 0.54 | 2.93 |
| tocilizumab | 244 | 74,200 | 7,499 | 2,853,617 | 7,743 | 1.25 | 1.10 | 1.42 | 1.25 | 1.10 | 1.42 |
| mirabegron | 39 | 74,405 | 1,213 | 2,859,903 | 1,252 | 1.25 | 0.91 | 1.72 | 1.25 | 0.91 | 1.72 |
| lorazepam | 50 | 74,394 | 1,556 | 2,859,560 | 1,606 | 1.25 | 0.94 | 1.65 | 1.25 | 0.94 | 1.65 |
| norethindrone | 5 | 74,439 | 169 | 2,860,947 | 174 | 1.25 | 0.53 | 2.92 | 1.25 | 0.53 | 2.92 |
| ambrisentan | 93 | 74,351 | 2,893 | 2,858,223 | 2,986 | 1.24 | 1.01 | 1.53 | 1.24 | 1.01 | 1.53 |
| etanercept szzs | 37 | 74,407 | 1,161 | 2,859,955 | 1,198 | 1.24 | 0.90 | 1.72 | 1.24 | 0.90 | 1.72 |
| divalproex | 32 | 74,412 | 1,013 | 2,860,103 | 1,045 | 1.23 | 0.87 | 1.75 | 1.23 | 0.87 | 1.75 |
| albuterol | 60 | 74,384 | 1,888 | 2,859,228 | 1,948 | 1.23 | 0.95 | 1.59 | 1.23 | 0.95 | 1.59 |
| pravastatin | 15 | 74,429 | 485 | 2,860,631 | 500 | 1.23 | 0.74 | 2.03 | 1.23 | 0.74 | 2.03 |
| ropinirole | 5 | 74,439 | 172 | 2,860,944 | 177 | 1.23 | 0.52 | 2.86 | 1.23 | 0.52 | 2.86 |
| prednisone | 79 | 74,365 | 2,494 | 2,858,622 | 2,573 | 1.23 | 0.98 | 1.53 | 1.22 | 0.98 | 1.53 |
| rituximab | 293 | 74,151 | 9,253 | 2,851,863 | 9,546 | 1.22 | 1.09 | 1.37 | 1.22 | 1.09 | 1.37 |
| voriconazole | 57 | 74,387 | 1,815 | 2,859,301 | 1,872 | 1.22 | 0.94 | 1.58 | 1.22 | 0.94 | 1.58 |
| ponatinib | 64 | 74,380 | 2,040 | 2,859,076 | 2,104 | 1.22 | 0.95 | 1.56 | 1.21 | 0.95 | 1.56 |
| infliximab | 454 | 73,990 | 14,441 | 2,846,675 | 14,895 | 1.21 | 1.10 | 1.33 | 1.21 | 1.10 | 1.33 |
| siponimod | 79 | 74,365 | 2,524 | 2,858,592 | 2,603 | 1.21 | 0.97 | 1.51 | 1.21 | 0.97 | 1.51 |
| abemaciclib | 91 | 74,353 | 2,912 | 2,858,204 | 3,003 | 1.21 | 0.98 | 1.49 | 1.21 | 0.98 | 1.49 |
| beclomethasone dipropionate | 9 | 74,435 | 303 | 2,860,813 | 312 | 1.20 | 0.63 | 2.29 | 1.20 | 0.63 | 2.29 |
| acalabrutinib | 54 | 74,390 | 1,743 | 2,859,373 | 1,797 | 1.20 | 0.92 | 1.57 | 1.20 | 0.92 | 1.57 |
| abiraterone | 119 | 74,325 | 3,841 | 2,857,275 | 3,960 | 1.20 | 1.00 | 1.43 | 1.20 | 1.00 | 1.43 |
| fruquintinib | 15 | 74,429 | 498 | 2,860,618 | 513 | 1.20 | 0.72 | 1.98 | 1.19 | 0.72 | 1.98 |
| bictegravir emtricitabine tenofovir alafenamide | 55 | 74,389 | 1,786 | 2,859,330 | 1,841 | 1.19 | 0.91 | 1.56 | 1.19 | 0.91 | 1.56 |
| clobazam | 17 | 74,427 | 564 | 2,860,552 | 581 | 1.19 | 0.74 | 1.92 | 1.19 | 0.74 | 1.92 |
| amikacin | 52 | 74,392 | 1,694 | 2,859,422 | 1,746 | 1.19 | 0.90 | 1.57 | 1.19 | 0.90 | 1.57 |
| alpha 1 proteinase inhibitor human | 50 | 74,394 | 1,630 | 2,859,486 | 1,680 | 1.19 | 0.90 | 1.58 | 1.19 | 0.90 | 1.57 |
| alirocumab | 64 | 74,380 | 2,085 | 2,859,031 | 2,149 | 1.19 | 0.93 | 1.52 | 1.19 | 0.93 | 1.52 |
| ixazomib | 137 | 74,307 | 4,467 | 2,856,649 | 4,604 | 1.18 | 1.00 | 1.40 | 1.18 | 1.00 | 1.40 |
| rituximab pvvr | 35 | 74,409 | 1,162 | 2,859,954 | 1,197 | 1.17 | 0.84 | 1.64 | 1.17 | 0.84 | 1.64 |
| testosterone undecanoate | 6 | 74,438 | 213 | 2,860,903 | 219 | 1.17 | 0.54 | 2.55 | 1.17 | 0.54 | 2.55 |
| tamsulosin | 39 | 74,405 | 1,300 | 2,859,816 | 1,339 | 1.17 | 0.85 | 1.60 | 1.17 | 0.85 | 1.60 |
| interferon beta 1a | 151 | 74,293 | 4,993 | 2,856,123 | 5,144 | 1.17 | 0.99 | 1.37 | 1.17 | 0.99 | 1.37 |
| adalimumab ryvk | 5 | 74,439 | 181 | 2,860,935 | 186 | 1.16 | 0.50 | 2.72 | 1.16 | 0.50 | 2.72 |
| ivosidenib | 7 | 74,437 | 248 | 2,860,868 | 255 | 1.16 | 0.56 | 2.40 | 1.16 | 0.56 | 2.40 |
| rizatriptan benzoate | 6 | 74,438 | 215 | 2,860,901 | 221 | 1.16 | 0.53 | 2.53 | 1.16 | 0.53 | 2.53 |
| methylprednisolone | 150 | 74,294 | 5,008 | 2,856,108 | 5,158 | 1.16 | 0.98 | 1.36 | 1.15 | 0.98 | 1.36 |
| dolutegravir | 19 | 74,425 | 649 | 2,860,467 | 668 | 1.15 | 0.74 | 1.81 | 1.15 | 0.74 | 1.81 |
| sipuleucel t | 5 | 74,439 | 183 | 2,860,933 | 188 | 1.15 | 0.49 | 2.69 | 1.15 | 0.49 | 2.69 |
| sarilumab | 86 | 74,358 | 2,889 | 2,858,227 | 2,975 | 1.15 | 0.93 | 1.43 | 1.15 | 0.93 | 1.42 |
| desmopressin | 13 | 74,431 | 456 | 2,860,660 | 469 | 1.14 | 0.66 | 1.95 | 1.14 | 0.66 | 1.95 |
| simvastatin | 45 | 74,399 | 1,540 | 2,859,576 | 1,585 | 1.14 | 0.85 | 1.52 | 1.14 | 0.85 | 1.52 |
| losartan | 60 | 74,384 | 2,050 | 2,859,066 | 2,110 | 1.13 | 0.88 | 1.46 | 1.13 | 0.88 | 1.46 |
| epirubicin | 30 | 74,414 | 1,034 | 2,860,082 | 1,064 | 1.13 | 0.79 | 1.62 | 1.13 | 0.79 | 1.62 |
| ticagrelor | 96 | 74,348 | 3,273 | 2,857,843 | 3,369 | 1.13 | 0.93 | 1.39 | 1.13 | 0.93 | 1.39 |
| zanubrutinib | 9 | 74,435 | 323 | 2,860,793 | 332 | 1.13 | 0.59 | 2.15 | 1.13 | 0.59 | 2.15 |
| bamlanivimab | 19 | 74,425 | 665 | 2,860,451 | 684 | 1.13 | 0.72 | 1.77 | 1.13 | 0.72 | 1.77 |
| dutasteride | 10 | 74,434 | 358 | 2,860,758 | 368 | 1.13 | 0.61 | 2.08 | 1.13 | 0.61 | 2.08 |
| tizanidine | 7 | 74,437 | 256 | 2,860,860 | 263 | 1.12 | 0.54 | 2.32 | 1.12 | 0.54 | 2.32 |
| anifrolumab fnia | 9 | 74,435 | 325 | 2,860,791 | 334 | 1.12 | 0.59 | 2.14 | 1.12 | 0.59 | 2.14 |
| adalimumab isopropyl alcohol | 24 | 74,420 | 839 | 2,860,277 | 863 | 1.12 | 0.75 | 1.68 | 1.12 | 0.75 | 1.68 |
| ravulizumab cwvz | 23 | 74,421 | 805 | 2,860,311 | 828 | 1.12 | 0.74 | 1.69 | 1.12 | 0.74 | 1.69 |
| polyethylene glycol 3350 | 64 | 74,380 | 2,211 | 2,858,905 | 2,275 | 1.12 | 0.88 | 1.44 | 1.12 | 0.88 | 1.44 |
| vandetanib | 13 | 74,431 | 463 | 2,860,653 | 476 | 1.12 | 0.65 | 1.92 | 1.12 | 0.65 | 1.92 |
| abacavir lamivudine | 6 | 74,438 | 223 | 2,860,893 | 229 | 1.12 | 0.51 | 2.44 | 1.12 | 0.51 | 2.44 |
| risperidone | 162 | 74,282 | 5,596 | 2,855,520 | 5,758 | 1.12 | 0.95 | 1.30 | 1.12 | 0.95 | 1.30 |
| eltrombopag olamine | 90 | 74,354 | 3,127 | 2,857,989 | 3,217 | 1.11 | 0.90 | 1.37 | 1.11 | 0.90 | 1.37 |
| dexlansoprazole | 18 | 74,426 | 642 | 2,860,474 | 660 | 1.11 | 0.70 | 1.76 | 1.11 | 0.70 | 1.76 |
| isotretinoin | 33 | 74,411 | 1,165 | 2,859,951 | 1,198 | 1.10 | 0.78 | 1.56 | 1.10 | 0.78 | 1.56 |
| fluticasone | 8 | 74,436 | 297 | 2,860,819 | 305 | 1.10 | 0.56 | 2.17 | 1.10 | 0.56 | 2.17 |
| onabotulinumtoxina | 53 | 74,391 | 1,876 | 2,859,240 | 1,929 | 1.10 | 0.83 | 1.44 | 1.10 | 0.84 | 1.44 |
| fluticasone furoate | 12 | 74,432 | 438 | 2,860,678 | 450 | 1.10 | 0.62 | 1.92 | 1.10 | 0.62 | 1.92 |
| ibrutinib | 294 | 74,150 | 10,359 | 2,850,757 | 10,653 | 1.09 | 0.97 | 1.23 | 1.09 | 0.97 | 1.23 |
| lanreotide | 68 | 74,376 | 2,420 | 2,858,696 | 2,488 | 1.09 | 0.86 | 1.38 | 1.09 | 0.86 | 1.38 |
| letrozole | 57 | 74,387 | 2,042 | 2,859,074 | 2,099 | 1.08 | 0.83 | 1.41 | 1.08 | 0.83 | 1.41 |
| bosentan | 73 | 74,371 | 2,614 | 2,858,502 | 2,687 | 1.08 | 0.86 | 1.36 | 1.08 | 0.86 | 1.36 |
| budesonide formoterol glycopyrronium | 26 | 74,418 | 952 | 2,860,164 | 978 | 1.07 | 0.73 | 1.57 | 1.07 | 0.73 | 1.57 |
| carvedilol | 16 | 74,428 | 595 | 2,860,521 | 611 | 1.06 | 0.65 | 1.74 | 1.06 | 0.65 | 1.74 |
| umeclidinium bromide | 11 | 74,433 | 415 | 2,860,701 | 426 | 1.06 | 0.59 | 1.91 | 1.06 | 0.59 | 1.91 |
| methotrexate | 181 | 74,263 | 6,590 | 2,854,526 | 6,771 | 1.06 | 0.91 | 1.23 | 1.06 | 0.91 | 1.23 |
| mavacamten | 23 | 74,421 | 853 | 2,860,263 | 876 | 1.06 | 0.70 | 1.59 | 1.06 | 0.70 | 1.59 |
| cedazuridine decitabine | 10 | 74,434 | 382 | 2,860,734 | 392 | 1.06 | 0.57 | 1.95 | 1.06 | 0.57 | 1.95 |
| elagolix estradiol norethindrone | 6 | 74,438 | 238 | 2,860,878 | 244 | 1.05 | 0.48 | 2.28 | 1.05 | 0.48 | 2.28 |
| cholestyramine | 8 | 74,436 | 312 | 2,860,804 | 320 | 1.05 | 0.53 | 2.07 | 1.05 | 0.53 | 2.07 |
| amlodipine besylate hydrochlorothiazide valsartan | 13 | 74,431 | 496 | 2,860,620 | 509 | 1.05 | 0.61 | 1.79 | 1.04 | 0.61 | 1.79 |
| emtricitabine tenofovir alafenamide | 8 | 74,436 | 313 | 2,860,803 | 321 | 1.04 | 0.53 | 2.06 | 1.04 | 0.53 | 2.06 |
| formoterol glycopyrrolate | 12 | 74,432 | 461 | 2,860,655 | 473 | 1.04 | 0.59 | 1.83 | 1.04 | 0.59 | 1.83 |
| anakinra | 49 | 74,395 | 1,830 | 2,859,286 | 1,879 | 1.04 | 0.78 | 1.38 | 1.04 | 0.78 | 1.38 |
| abatacept | 258 | 74,186 | 9,613 | 2,851,503 | 9,871 | 1.03 | 0.91 | 1.17 | 1.03 | 0.91 | 1.17 |
| tramadol | 61 | 74,383 | 2,288 | 2,858,828 | 2,349 | 1.03 | 0.80 | 1.33 | 1.03 | 0.80 | 1.33 |
| apalutamide | 87 | 74,357 | 3,264 | 2,857,852 | 3,351 | 1.03 | 0.83 | 1.27 | 1.03 | 0.83 | 1.27 |
| risankizumab rzaa | 216 | 74,228 | 8,092 | 2,853,024 | 8,308 | 1.03 | 0.90 | 1.18 | 1.03 | 0.90 | 1.18 |
| lamotrigine | 70 | 74,374 | 2,635 | 2,858,481 | 2,705 | 1.03 | 0.81 | 1.30 | 1.03 | 0.81 | 1.30 |
| acyclovir | 35 | 74,409 | 1,334 | 2,859,782 | 1,369 | 1.02 | 0.73 | 1.43 | 1.02 | 0.73 | 1.43 |
| apomorphine | 25 | 74,419 | 962 | 2,860,154 | 987 | 1.02 | 0.69 | 1.51 | 1.02 | 0.69 | 1.51 |
| rotigotine | 43 | 74,401 | 1,648 | 2,859,468 | 1,691 | 1.01 | 0.75 | 1.37 | 1.01 | 0.75 | 1.37 |
| vismodegib | 37 | 74,407 | 1,424 | 2,859,692 | 1,461 | 1.01 | 0.73 | 1.40 | 1.01 | 0.73 | 1.40 |
| acetaminophen tramadol | 12 | 74,432 | 475 | 2,860,641 | 487 | 1.01 | 0.58 | 1.77 | 1.01 | 0.58 | 1.77 |
| cabozantinib s | 132 | 74,312 | 5,072 | 2,856,044 | 5,204 | 1.00 | 0.84 | 1.19 | 1.00 | 0.84 | 1.19 |
| estradiol levonorgestrel | 7 | 74,437 | 287 | 2,860,829 | 294 | 1.00 | 0.49 | 2.07 | 1.00 | 0.49 | 2.07 |
| botulinum toxin type a | 6 | 74,438 | 249 | 2,860,867 | 255 | 1.00 | 0.46 | 2.18 | 1.00 | 0.46 | 2.18 |
| levocetirizine dihydrochloride | 8 | 74,436 | 327 | 2,860,789 | 335 | 1.00 | 0.50 | 1.97 | 1.00 | 0.50 | 1.97 |
| hydrochlorothiazide losartan | 12 | 74,432 | 483 | 2,860,633 | 495 | 0.99 | 0.57 | 1.74 | 0.99 | 0.57 | 1.74 |
| miglustat | 5 | 74,439 | 213 | 2,860,903 | 218 | 0.99 | 0.42 | 2.31 | 0.99 | 0.42 | 2.31 |
| lithium | 8 | 74,436 | 330 | 2,860,786 | 338 | 0.99 | 0.50 | 1.95 | 0.99 | 0.50 | 1.95 |
| vedolizumab | 335 | 74,109 | 13,076 | 2,848,040 | 13,411 | 0.99 | 0.88 | 1.10 | 0.99 | 0.88 | 1.10 |
| regorafenib | 47 | 74,397 | 1,852 | 2,859,264 | 1,899 | 0.99 | 0.74 | 1.31 | 0.99 | 0.74 | 1.31 |
| idursulfase | 34 | 74,410 | 1,347 | 2,859,769 | 1,381 | 0.98 | 0.70 | 1.38 | 0.98 | 0.70 | 1.38 |
| candesartan cilexetil | 20 | 74,424 | 804 | 2,860,312 | 824 | 0.98 | 0.63 | 1.52 | 0.98 | 0.63 | 1.52 |
| nilotinib | 91 | 74,353 | 3,602 | 2,857,514 | 3,693 | 0.98 | 0.79 | 1.20 | 0.98 | 0.79 | 1.20 |
| ezetimibe simvastatin | 5 | 74,439 | 217 | 2,860,899 | 222 | 0.97 | 0.42 | 2.27 | 0.97 | 0.42 | 2.27 |
| rilonacept | 6 | 74,438 | 257 | 2,860,859 | 263 | 0.97 | 0.45 | 2.11 | 0.97 | 0.45 | 2.11 |
| cladribine | 49 | 74,395 | 1,961 | 2,859,155 | 2,010 | 0.97 | 0.73 | 1.29 | 0.97 | 0.73 | 1.29 |
| pegvaliase pqpz | 25 | 74,419 | 1,010 | 2,860,106 | 1,035 | 0.97 | 0.65 | 1.44 | 0.97 | 0.65 | 1.44 |
| triptorelin | 12 | 74,432 | 495 | 2,860,621 | 507 | 0.97 | 0.55 | 1.70 | 0.97 | 0.55 | 1.70 |
| azelastine | 11 | 74,433 | 456 | 2,860,660 | 467 | 0.97 | 0.54 | 1.74 | 0.97 | 0.54 | 1.74 |
| cabotegravir | 11 | 74,433 | 456 | 2,860,660 | 467 | 0.97 | 0.54 | 1.74 | 0.97 | 0.54 | 1.74 |
| migalastat | 8 | 74,436 | 338 | 2,860,778 | 346 | 0.97 | 0.49 | 1.91 | 0.97 | 0.49 | 1.91 |
| fluticasone propionate salmeterol xinafoate | 41 | 74,403 | 1,655 | 2,859,461 | 1,696 | 0.96 | 0.71 | 1.31 | 0.96 | 0.71 | 1.31 |
| etonogestrel | 8 | 74,436 | 340 | 2,860,776 | 348 | 0.96 | 0.49 | 1.89 | 0.96 | 0.49 | 1.89 |
| rasagiline | 11 | 74,433 | 461 | 2,860,655 | 472 | 0.96 | 0.53 | 1.72 | 0.96 | 0.53 | 1.72 |
| donepezil | 25 | 74,419 | 1,026 | 2,860,090 | 1,051 | 0.95 | 0.64 | 1.41 | 0.95 | 0.64 | 1.41 |
| lecanemab irmb | 15 | 74,429 | 625 | 2,860,491 | 640 | 0.95 | 0.58 | 1.58 | 0.95 | 0.58 | 1.58 |
| evolocumab | 77 | 74,367 | 3,135 | 2,857,981 | 3,212 | 0.95 | 0.76 | 1.19 | 0.95 | 0.76 | 1.19 |
| peginterferon alfa 2a | 11 | 74,433 | 466 | 2,860,650 | 477 | 0.95 | 0.53 | 1.70 | 0.95 | 0.53 | 1.70 |
| upadacitinib | 260 | 74,184 | 10,590 | 2,850,526 | 10,850 | 0.95 | 0.84 | 1.07 | 0.95 | 0.84 | 1.07 |
| bosutinib | 38 | 74,406 | 1,567 | 2,859,549 | 1,605 | 0.94 | 0.69 | 1.30 | 0.94 | 0.69 | 1.30 |
| buprenorphine naloxone | 7 | 74,437 | 305 | 2,860,811 | 312 | 0.94 | 0.46 | 1.95 | 0.94 | 0.46 | 1.95 |
| human immunoglobulin g | 262 | 74,182 | 10,755 | 2,850,361 | 11,017 | 0.94 | 0.83 | 1.06 | 0.94 | 0.83 | 1.06 |
| sotalol | 5 | 74,439 | 225 | 2,860,891 | 230 | 0.94 | 0.40 | 2.18 | 0.94 | 0.40 | 2.18 |
| ipratropium bromide | 11 | 74,433 | 472 | 2,860,644 | 483 | 0.94 | 0.52 | 1.68 | 0.94 | 0.52 | 1.68 |
| dulaglutide | 75 | 74,369 | 3,111 | 2,858,005 | 3,186 | 0.93 | 0.74 | 1.17 | 0.93 | 0.74 | 1.17 |
| ribavirin | 15 | 74,429 | 640 | 2,860,476 | 655 | 0.93 | 0.56 | 1.54 | 0.93 | 0.56 | 1.54 |
| hydrochlorothiazide valsartan | 18 | 74,426 | 766 | 2,860,350 | 784 | 0.93 | 0.58 | 1.47 | 0.93 | 0.58 | 1.47 |
| hydroxyzine | 8 | 74,436 | 352 | 2,860,764 | 360 | 0.93 | 0.47 | 1.83 | 0.93 | 0.47 | 1.83 |
| satralizumab mwge | 10 | 74,434 | 438 | 2,860,678 | 448 | 0.92 | 0.50 | 1.70 | 0.92 | 0.50 | 1.70 |
| tafamidis | 28 | 74,416 | 1,193 | 2,859,923 | 1,221 | 0.92 | 0.63 | 1.33 | 0.92 | 0.63 | 1.33 |
| telmisartan | 12 | 74,432 | 528 | 2,860,588 | 540 | 0.91 | 0.52 | 1.59 | 0.91 | 0.52 | 1.59 |
| rivastigmine | 28 | 74,416 | 1,205 | 2,859,911 | 1,233 | 0.91 | 0.63 | 1.32 | 0.91 | 0.63 | 1.32 |
| estradiol norethindrone relugolix | 7 | 74,437 | 318 | 2,860,798 | 325 | 0.90 | 0.44 | 1.87 | 0.91 | 0.44 | 1.87 |
| ubrogepant | 9 | 74,435 | 404 | 2,860,712 | 413 | 0.90 | 0.47 | 1.72 | 0.90 | 0.47 | 1.72 |
| emtricitabine tenofovir | 12 | 74,432 | 532 | 2,860,584 | 544 | 0.90 | 0.51 | 1.58 | 0.90 | 0.51 | 1.58 |
| ibandronate | 15 | 74,429 | 661 | 2,860,455 | 676 | 0.90 | 0.54 | 1.49 | 0.90 | 0.54 | 1.49 |
| tucatinib | 9 | 74,435 | 405 | 2,860,711 | 414 | 0.90 | 0.47 | 1.71 | 0.90 | 0.47 | 1.71 |
| voxelotor | 63 | 74,381 | 2,718 | 2,858,398 | 2,781 | 0.90 | 0.70 | 1.15 | 0.90 | 0.70 | 1.15 |
| clonidine | 18 | 74,426 | 792 | 2,860,324 | 810 | 0.90 | 0.57 | 1.42 | 0.90 | 0.57 | 1.42 |
| levetiracetam | 93 | 74,351 | 4,021 | 2,857,095 | 4,114 | 0.89 | 0.73 | 1.10 | 0.89 | 0.73 | 1.10 |
| olanzapine | 69 | 74,375 | 2,994 | 2,858,122 | 3,063 | 0.89 | 0.70 | 1.13 | 0.89 | 0.70 | 1.13 |
| bimekizumab | 10 | 74,434 | 452 | 2,860,664 | 462 | 0.89 | 0.48 | 1.64 | 0.89 | 0.48 | 1.64 |
| arformoterol | 5 | 74,439 | 237 | 2,860,879 | 242 | 0.89 | 0.38 | 2.07 | 0.89 | 0.38 | 2.07 |
| tramadol | 7 | 74,437 | 324 | 2,860,792 | 331 | 0.89 | 0.43 | 1.83 | 0.89 | 0.43 | 1.83 |
| metoprolol | 48 | 74,396 | 2,117 | 2,858,999 | 2,165 | 0.88 | 0.66 | 1.17 | 0.88 | 0.66 | 1.17 |
| alpelisib | 41 | 74,403 | 1,825 | 2,859,291 | 1,866 | 0.87 | 0.64 | 1.19 | 0.87 | 0.64 | 1.19 |
| apixaban | 192 | 74,252 | 8,531 | 2,852,585 | 8,723 | 0.87 | 0.75 | 1.00 | 0.87 | 0.75 | 1.00 |
| lisinopril | 52 | 74,392 | 2,330 | 2,858,786 | 2,382 | 0.87 | 0.66 | 1.14 | 0.87 | 0.66 | 1.14 |
| valsartan | 40 | 74,404 | 1,804 | 2,859,312 | 1,844 | 0.86 | 0.63 | 1.18 | 0.86 | 0.63 | 1.18 |
| eplerenone | 6 | 74,438 | 291 | 2,860,825 | 297 | 0.86 | 0.39 | 1.86 | 0.86 | 0.39 | 1.86 |
| omadacycline | 10 | 74,434 | 475 | 2,860,641 | 485 | 0.85 | 0.46 | 1.56 | 0.85 | 0.46 | 1.56 |
| amlodipine besylate | 85 | 74,359 | 3,885 | 2,857,231 | 3,970 | 0.85 | 0.68 | 1.05 | 0.85 | 0.68 | 1.05 |
| methadone | 9 | 74,435 | 432 | 2,860,684 | 441 | 0.84 | 0.44 | 1.61 | 0.84 | 0.44 | 1.61 |
| natalizumab | 57 | 74,387 | 2,617 | 2,858,499 | 2,674 | 0.84 | 0.65 | 1.10 | 0.84 | 0.65 | 1.10 |
| zonisamide | 6 | 74,438 | 296 | 2,860,820 | 302 | 0.84 | 0.39 | 1.83 | 0.84 | 0.39 | 1.83 |
| celecoxib | 37 | 74,407 | 1,713 | 2,859,403 | 1,750 | 0.84 | 0.61 | 1.16 | 0.84 | 0.61 | 1.16 |
| bebtelovimab | 6 | 74,438 | 297 | 2,860,819 | 303 | 0.84 | 0.39 | 1.83 | 0.84 | 0.39 | 1.83 |
| etanercept | 175 | 74,269 | 8,040 | 2,853,076 | 8,215 | 0.84 | 0.72 | 0.97 | 0.84 | 0.72 | 0.97 |
| doxycycline hyclate | 10 | 74,434 | 481 | 2,860,635 | 491 | 0.84 | 0.45 | 1.54 | 0.84 | 0.45 | 1.54 |
| cabotegravir rilpivirine | 9 | 74,435 | 437 | 2,860,679 | 446 | 0.83 | 0.44 | 1.59 | 0.83 | 0.44 | 1.59 |
| elbasvir grazoprevir | 6 | 74,438 | 299 | 2,860,817 | 305 | 0.83 | 0.38 | 1.81 | 0.83 | 0.38 | 1.81 |
| hydrochlorothiazide lisinopril | 7 | 74,437 | 346 | 2,860,770 | 353 | 0.83 | 0.40 | 1.71 | 0.83 | 0.40 | 1.71 |
| raltegravir | 13 | 74,431 | 625 | 2,860,491 | 638 | 0.83 | 0.48 | 1.42 | 0.83 | 0.48 | 1.42 |
| lorlatinib | 34 | 74,410 | 1,598 | 2,859,518 | 1,632 | 0.83 | 0.59 | 1.16 | 0.83 | 0.59 | 1.16 |
| pancrelipase amylase pancrelipase lipase pancrelipase protease | 59 | 74,385 | 2,762 | 2,858,354 | 2,821 | 0.83 | 0.64 | 1.07 | 0.83 | 0.64 | 1.07 |
| fesoterodine | 9 | 74,435 | 442 | 2,860,674 | 451 | 0.83 | 0.43 | 1.57 | 0.83 | 0.43 | 1.57 |
| mitotane | 7 | 74,437 | 352 | 2,860,764 | 359 | 0.82 | 0.40 | 1.69 | 0.82 | 0.40 | 1.69 |
| benralizumab | 77 | 74,367 | 3,687 | 2,857,429 | 3,764 | 0.81 | 0.64 | 1.01 | 0.81 | 0.65 | 1.01 |
| lacosamide | 43 | 74,401 | 2,094 | 2,859,022 | 2,137 | 0.80 | 0.59 | 1.08 | 0.80 | 0.59 | 1.08 |
| hydrochlorothiazide olmesartan medoxomil | 7 | 74,437 | 363 | 2,860,753 | 370 | 0.79 | 0.38 | 1.63 | 0.79 | 0.38 | 1.63 |
| icosapent ethyl | 16 | 74,428 | 808 | 2,860,308 | 824 | 0.78 | 0.48 | 1.28 | 0.78 | 0.48 | 1.28 |
| metronidazole | 24 | 74,420 | 1,201 | 2,859,915 | 1,225 | 0.78 | 0.53 | 1.17 | 0.78 | 0.53 | 1.17 |
| diazepam | 27 | 74,417 | 1,349 | 2,859,767 | 1,376 | 0.78 | 0.54 | 1.14 | 0.78 | 0.54 | 1.14 |
| linaclotide | 22 | 74,422 | 1,107 | 2,860,009 | 1,129 | 0.78 | 0.51 | 1.19 | 0.78 | 0.51 | 1.19 |
| atorvastatin | 103 | 74,341 | 5,099 | 2,856,017 | 5,202 | 0.78 | 0.64 | 0.95 | 0.78 | 0.64 | 0.95 |
| omeprazole | 101 | 74,343 | 5,009 | 2,856,107 | 5,110 | 0.78 | 0.64 | 0.95 | 0.78 | 0.64 | 0.95 |
| umeclidinium bromide vilanterol trifenatate | 17 | 74,427 | 865 | 2,860,251 | 882 | 0.78 | 0.48 | 1.25 | 0.78 | 0.48 | 1.25 |
| teduglutide | 73 | 74,371 | 3,640 | 2,857,476 | 3,713 | 0.78 | 0.62 | 0.98 | 0.78 | 0.62 | 0.98 |
| certolizumab pegol | 184 | 74,260 | 9,142 | 2,851,974 | 9,326 | 0.78 | 0.67 | 0.90 | 0.78 | 0.67 | 0.90 |
| laronidase | 20 | 74,424 | 1,016 | 2,860,100 | 1,036 | 0.78 | 0.50 | 1.20 | 0.78 | 0.50 | 1.20 |
| sulfasalazine | 13 | 74,431 | 671 | 2,860,445 | 684 | 0.77 | 0.45 | 1.32 | 0.77 | 0.45 | 1.32 |
| terbinafine | 12 | 74,432 | 623 | 2,860,493 | 635 | 0.77 | 0.44 | 1.35 | 0.77 | 0.44 | 1.35 |
| memantine | 8 | 74,436 | 424 | 2,860,692 | 432 | 0.77 | 0.39 | 1.52 | 0.77 | 0.39 | 1.52 |
| mometasone furoate | 11 | 74,433 | 575 | 2,860,541 | 586 | 0.77 | 0.43 | 1.38 | 0.77 | 0.43 | 1.38 |
| pegloticase | 10 | 74,434 | 525 | 2,860,591 | 535 | 0.77 | 0.42 | 1.41 | 0.77 | 0.42 | 1.41 |
| fostamatinib | 27 | 74,417 | 1,387 | 2,859,729 | 1,414 | 0.76 | 0.52 | 1.11 | 0.76 | 0.52 | 1.11 |
| teriparatide | 55 | 74,389 | 2,805 | 2,858,311 | 2,860 | 0.76 | 0.58 | 0.99 | 0.76 | 0.58 | 0.99 |
| mobocertinib | 5 | 74,439 | 278 | 2,860,838 | 283 | 0.76 | 0.33 | 1.77 | 0.76 | 0.33 | 1.77 |
| rilpivirine | 16 | 74,428 | 835 | 2,860,281 | 851 | 0.76 | 0.47 | 1.24 | 0.76 | 0.47 | 1.24 |
| sunitinib | 50 | 74,394 | 2,557 | 2,858,559 | 2,607 | 0.76 | 0.57 | 1.00 | 0.76 | 0.57 | 1.00 |
| sildenafil | 66 | 74,378 | 3,368 | 2,857,748 | 3,434 | 0.76 | 0.59 | 0.97 | 0.76 | 0.60 | 0.97 |
| somatropin | 123 | 74,321 | 6,289 | 2,854,827 | 6,412 | 0.75 | 0.63 | 0.90 | 0.75 | 0.63 | 0.90 |
| sulfamethoxazole trimethoprim | 23 | 74,421 | 1,201 | 2,859,915 | 1,224 | 0.75 | 0.50 | 1.13 | 0.75 | 0.50 | 1.13 |
| efavirenz emtricitabine tenofovir disoproxil | 51 | 74,393 | 2,639 | 2,858,477 | 2,690 | 0.75 | 0.57 | 0.99 | 0.75 | 0.57 | 0.99 |
| haloperidol | 18 | 74,426 | 952 | 2,860,164 | 970 | 0.75 | 0.47 | 1.18 | 0.75 | 0.47 | 1.18 |
| atenolol | 16 | 74,428 | 850 | 2,860,266 | 866 | 0.75 | 0.46 | 1.21 | 0.75 | 0.46 | 1.21 |
| budesonide formoterol glycopyrrolate | 15 | 74,429 | 799 | 2,860,317 | 814 | 0.75 | 0.45 | 1.23 | 0.75 | 0.45 | 1.23 |
| paricalcitol | 13 | 74,431 | 701 | 2,860,415 | 714 | 0.74 | 0.43 | 1.27 | 0.74 | 0.43 | 1.27 |
| avatrombopag | 11 | 74,433 | 599 | 2,860,517 | 610 | 0.74 | 0.41 | 1.32 | 0.74 | 0.41 | 1.32 |
| cobicistat darunavir emtricitabine tenofovir alafenamide | 9 | 74,435 | 495 | 2,860,621 | 504 | 0.74 | 0.39 | 1.40 | 0.74 | 0.39 | 1.40 |
| degarelix | 8 | 74,436 | 444 | 2,860,672 | 452 | 0.73 | 0.37 | 1.45 | 0.73 | 0.37 | 1.45 |
| pralsetinib | 13 | 74,431 | 713 | 2,860,403 | 726 | 0.73 | 0.42 | 1.25 | 0.73 | 0.42 | 1.25 |
| testosterone | 10 | 74,434 | 556 | 2,860,560 | 566 | 0.73 | 0.39 | 1.34 | 0.73 | 0.39 | 1.34 |
| human immunoglobulin g hyaluronidase human recombinant | 22 | 74,422 | 1,199 | 2,859,917 | 1,221 | 0.72 | 0.47 | 1.09 | 0.72 | 0.48 | 1.09 |
| brigatinib | 22 | 74,422 | 1,209 | 2,859,907 | 1,231 | 0.71 | 0.47 | 1.08 | 0.71 | 0.47 | 1.08 |
| dapagliflozin propanediol | 42 | 74,402 | 2,291 | 2,858,825 | 2,333 | 0.71 | 0.53 | 0.97 | 0.71 | 0.53 | 0.97 |
| cobicistat elvitegravir emtricitabine tenofovir disoproxil | 14 | 74,430 | 785 | 2,860,331 | 799 | 0.71 | 0.42 | 1.19 | 0.71 | 0.42 | 1.19 |
| drospirenone ethinyl estradiol | 20 | 74,424 | 1,111 | 2,860,005 | 1,131 | 0.71 | 0.46 | 1.10 | 0.71 | 0.46 | 1.10 |
| phenytoin | 15 | 74,429 | 844 | 2,860,272 | 859 | 0.71 | 0.43 | 1.17 | 0.71 | 0.43 | 1.17 |
| minocycline | 5 | 74,439 | 300 | 2,860,816 | 305 | 0.70 | 0.30 | 1.63 | 0.70 | 0.30 | 1.63 |
| lapatinib ditosylate | 8 | 74,436 | 464 | 2,860,652 | 472 | 0.70 | 0.36 | 1.39 | 0.70 | 0.36 | 1.39 |
| tolvaptan | 41 | 74,403 | 2,267 | 2,858,849 | 2,308 | 0.70 | 0.52 | 0.96 | 0.70 | 0.52 | 0.96 |
| sapropterin dihydrochloride | 16 | 74,428 | 906 | 2,860,210 | 922 | 0.70 | 0.43 | 1.14 | 0.70 | 0.43 | 1.14 |
| dexamethasone | 50 | 74,394 | 2,777 | 2,858,339 | 2,827 | 0.70 | 0.53 | 0.92 | 0.70 | 0.53 | 0.92 |
| zolpidem | 20 | 74,424 | 1,129 | 2,859,987 | 1,149 | 0.70 | 0.45 | 1.08 | 0.70 | 0.45 | 1.08 |
| abrocitinib | 15 | 74,429 | 854 | 2,860,262 | 869 | 0.70 | 0.42 | 1.15 | 0.70 | 0.42 | 1.15 |
| insulin degludec | 27 | 74,417 | 1,516 | 2,859,600 | 1,543 | 0.70 | 0.48 | 1.02 | 0.70 | 0.48 | 1.02 |
| droxidopa | 34 | 74,410 | 1,904 | 2,859,212 | 1,938 | 0.70 | 0.50 | 0.97 | 0.70 | 0.50 | 0.97 |
| baclofen | 10 | 74,434 | 580 | 2,860,536 | 590 | 0.70 | 0.38 | 1.28 | 0.70 | 0.38 | 1.28 |
| tiotropium bromide | 21 | 74,423 | 1,189 | 2,859,927 | 1,210 | 0.69 | 0.45 | 1.06 | 0.69 | 0.45 | 1.06 |
| capecitabine | 99 | 74,345 | 5,521 | 2,855,595 | 5,620 | 0.69 | 0.57 | 0.84 | 0.69 | 0.57 | 0.84 |
| dasatinib | 69 | 74,375 | 3,859 | 2,857,257 | 3,928 | 0.69 | 0.55 | 0.88 | 0.69 | 0.55 | 0.88 |
| parathyroid hormone | 12 | 74,432 | 694 | 2,860,422 | 706 | 0.69 | 0.40 | 1.21 | 0.69 | 0.40 | 1.21 |
| azithromycin | 34 | 74,410 | 1,920 | 2,859,196 | 1,954 | 0.69 | 0.49 | 0.97 | 0.69 | 0.49 | 0.97 |
| belumosudil | 13 | 74,431 | 756 | 2,860,360 | 769 | 0.69 | 0.40 | 1.17 | 0.69 | 0.40 | 1.17 |
| scopolamine | 6 | 74,438 | 364 | 2,860,752 | 370 | 0.69 | 0.32 | 1.49 | 0.69 | 0.32 | 1.49 |
| liraglutide | 50 | 74,394 | 2,841 | 2,858,275 | 2,891 | 0.68 | 0.52 | 0.90 | 0.68 | 0.52 | 0.90 |
| olodaterol tiotropium bromide | 7 | 74,437 | 423 | 2,860,693 | 430 | 0.68 | 0.33 | 1.40 | 0.68 | 0.33 | 1.40 |
| exenatide | 42 | 74,402 | 2,401 | 2,858,715 | 2,443 | 0.68 | 0.50 | 0.92 | 0.68 | 0.50 | 0.92 |
| goserelin | 14 | 74,430 | 820 | 2,860,296 | 834 | 0.68 | 0.40 | 1.14 | 0.68 | 0.40 | 1.14 |
| tetrabenazine | 6 | 74,438 | 369 | 2,860,747 | 375 | 0.68 | 0.31 | 1.47 | 0.68 | 0.31 | 1.47 |
| ascorbic acid polyethylene glycol 3350 chloride ascorbate chloride | 9 | 74,435 | 540 | 2,860,576 | 549 | 0.68 | 0.36 | 1.28 | 0.68 | 0.36 | 1.28 |
| diltiazem | 17 | 74,427 | 996 | 2,860,120 | 1,013 | 0.67 | 0.42 | 1.08 | 0.67 | 0.42 | 1.08 |
| ranolazine | 10 | 74,434 | 606 | 2,860,510 | 616 | 0.67 | 0.36 | 1.22 | 0.67 | 0.36 | 1.22 |
| axitinib | 43 | 74,401 | 2,518 | 2,858,598 | 2,561 | 0.66 | 0.49 | 0.90 | 0.66 | 0.49 | 0.90 |
| hydroxychloroquine | 19 | 74,425 | 1,129 | 2,859,987 | 1,148 | 0.66 | 0.42 | 1.04 | 0.66 | 0.42 | 1.04 |
| abobotulinumtoxina | 9 | 74,435 | 551 | 2,860,565 | 560 | 0.66 | 0.35 | 1.26 | 0.66 | 0.35 | 1.26 |
| elotuzumab | 6 | 74,438 | 379 | 2,860,737 | 385 | 0.66 | 0.30 | 1.43 | 0.66 | 0.30 | 1.43 |
| peginterferon beta 1a | 19 | 74,425 | 1,148 | 2,859,968 | 1,167 | 0.65 | 0.42 | 1.02 | 0.65 | 0.42 | 1.02 |
| teduglutide water | 5 | 74,439 | 324 | 2,860,792 | 329 | 0.65 | 0.28 | 1.51 | 0.65 | 0.28 | 1.51 |
| lenvatinib | 147 | 74,297 | 8,696 | 2,852,420 | 8,843 | 0.65 | 0.55 | 0.77 | 0.65 | 0.55 | 0.77 |
| cobicistat elvitegravir emtricitabine tenofovir alafenamide | 6 | 74,438 | 384 | 2,860,732 | 390 | 0.65 | 0.30 | 1.41 | 0.65 | 0.30 | 1.41 |
| glatiramer | 64 | 74,380 | 3,821 | 2,857,295 | 3,885 | 0.65 | 0.51 | 0.83 | 0.65 | 0.51 | 0.83 |
| medroxyprogesterone | 11 | 74,433 | 693 | 2,860,423 | 704 | 0.64 | 0.36 | 1.14 | 0.64 | 0.36 | 1.14 |
| doxycycline | 21 | 74,423 | 1,303 | 2,859,813 | 1,324 | 0.63 | 0.41 | 0.97 | 0.63 | 0.41 | 0.97 |
| candesartan | 9 | 74,435 | 577 | 2,860,539 | 586 | 0.63 | 0.33 | 1.20 | 0.63 | 0.33 | 1.20 |
| molnupiravir | 28 | 74,416 | 1,735 | 2,859,381 | 1,763 | 0.63 | 0.44 | 0.91 | 0.63 | 0.44 | 0.91 |
| haloperidol decanoate | 5 | 74,439 | 336 | 2,860,780 | 341 | 0.63 | 0.27 | 1.46 | 0.63 | 0.27 | 1.46 |
| cilastatin imipenem | 20 | 74,424 | 1,254 | 2,859,862 | 1,274 | 0.63 | 0.41 | 0.97 | 0.63 | 0.41 | 0.97 |
| diphenhydramine | 10 | 74,434 | 643 | 2,860,473 | 653 | 0.63 | 0.34 | 1.15 | 0.63 | 0.34 | 1.15 |
| meloxicam | 6 | 74,438 | 398 | 2,860,718 | 404 | 0.63 | 0.29 | 1.36 | 0.63 | 0.29 | 1.36 |
| pantoprazole | 55 | 74,389 | 3,409 | 2,857,707 | 3,464 | 0.63 | 0.48 | 0.82 | 0.63 | 0.48 | 0.82 |
| bicalutamide | 6 | 74,438 | 399 | 2,860,717 | 405 | 0.63 | 0.29 | 1.36 | 0.63 | 0.29 | 1.36 |
| everolimus | 61 | 74,383 | 3,818 | 2,857,298 | 3,879 | 0.62 | 0.48 | 0.80 | 0.62 | 0.48 | 0.80 |
| ivacaftor tezacaftor | 20 | 74,424 | 1,276 | 2,859,840 | 1,296 | 0.62 | 0.40 | 0.95 | 0.62 | 0.40 | 0.95 |
| dornase alfa | 14 | 74,430 | 904 | 2,860,212 | 918 | 0.62 | 0.37 | 1.04 | 0.62 | 0.37 | 1.04 |
| capmatinib | 10 | 74,434 | 655 | 2,860,461 | 665 | 0.62 | 0.33 | 1.13 | 0.62 | 0.33 | 1.13 |
| tezepelumab ekko | 10 | 74,434 | 656 | 2,860,460 | 666 | 0.61 | 0.33 | 1.13 | 0.61 | 0.33 | 1.13 |
| velaglucerase alfa | 11 | 74,433 | 719 | 2,860,397 | 730 | 0.61 | 0.34 | 1.10 | 0.61 | 0.34 | 1.10 |
| ritonavir | 7 | 74,437 | 469 | 2,860,647 | 476 | 0.61 | 0.30 | 1.26 | 0.61 | 0.30 | 1.26 |
| albuterol ipratropium bromide | 6 | 74,438 | 407 | 2,860,709 | 413 | 0.61 | 0.28 | 1.33 | 0.61 | 0.28 | 1.33 |
| hydrocortisone | 13 | 74,431 | 846 | 2,860,270 | 859 | 0.61 | 0.36 | 1.05 | 0.61 | 0.36 | 1.05 |
| romosozumab aqqg | 23 | 74,421 | 1,482 | 2,859,634 | 1,505 | 0.61 | 0.41 | 0.92 | 0.61 | 0.41 | 0.92 |
| insulin glargine | 52 | 74,392 | 3,312 | 2,857,804 | 3,364 | 0.61 | 0.46 | 0.80 | 0.61 | 0.46 | 0.80 |
| galsulfase | 13 | 74,431 | 857 | 2,860,259 | 870 | 0.60 | 0.35 | 1.04 | 0.61 | 0.35 | 1.04 |
| emtricitabine tenofovir disoproxil | 68 | 74,376 | 4,415 | 2,856,701 | 4,483 | 0.60 | 0.47 | 0.76 | 0.60 | 0.47 | 0.76 |
| desloratadine | 5 | 74,439 | 357 | 2,860,759 | 362 | 0.59 | 0.25 | 1.37 | 0.59 | 0.25 | 1.37 |
| diclofenac | 37 | 74,407 | 2,445 | 2,858,671 | 2,482 | 0.59 | 0.43 | 0.81 | 0.59 | 0.43 | 0.81 |
| eltrombopag | 15 | 74,429 | 1,014 | 2,860,102 | 1,029 | 0.59 | 0.36 | 0.97 | 0.59 | 0.36 | 0.97 |
| denosumab | 70 | 74,374 | 4,619 | 2,856,497 | 4,689 | 0.59 | 0.46 | 0.74 | 0.59 | 0.46 | 0.74 |
| naproxen | 33 | 74,411 | 2,198 | 2,858,918 | 2,231 | 0.59 | 0.42 | 0.82 | 0.59 | 0.42 | 0.82 |
| riociguat | 25 | 74,419 | 1,683 | 2,859,433 | 1,708 | 0.58 | 0.39 | 0.86 | 0.58 | 0.39 | 0.86 |
| alectinib | 43 | 74,401 | 2,873 | 2,858,243 | 2,916 | 0.58 | 0.43 | 0.78 | 0.58 | 0.43 | 0.78 |
| metformin sitagliptin | 21 | 74,423 | 1,423 | 2,859,693 | 1,444 | 0.58 | 0.38 | 0.89 | 0.58 | 0.38 | 0.89 |
| olaparib | 53 | 74,391 | 3,560 | 2,857,556 | 3,613 | 0.58 | 0.44 | 0.76 | 0.58 | 0.44 | 0.76 |
| amiodarone | 30 | 74,414 | 2,031 | 2,859,085 | 2,061 | 0.58 | 0.40 | 0.82 | 0.58 | 0.40 | 0.82 |
| riluzole | 6 | 74,438 | 435 | 2,860,681 | 441 | 0.57 | 0.26 | 1.24 | 0.57 | 0.26 | 1.24 |
| hyaluronidase zzxf pertuzumab trastuzumab | 8 | 74,436 | 572 | 2,860,544 | 580 | 0.57 | 0.29 | 1.12 | 0.57 | 0.29 | 1.12 |
| oxcarbazepine | 13 | 74,431 | 918 | 2,860,198 | 931 | 0.56 | 0.33 | 0.97 | 0.56 | 0.33 | 0.97 |
| human c1 esterase inhibitor | 37 | 74,407 | 2,560 | 2,858,556 | 2,597 | 0.56 | 0.41 | 0.78 | 0.56 | 0.41 | 0.78 |
| amoxicillin clavulanate | 16 | 74,428 | 1,127 | 2,859,989 | 1,143 | 0.56 | 0.35 | 0.91 | 0.56 | 0.35 | 0.91 |
| metreleptin | 5 | 74,439 | 379 | 2,860,737 | 384 | 0.56 | 0.24 | 1.29 | 0.56 | 0.24 | 1.29 |
| brodalumab | 15 | 74,429 | 1,069 | 2,860,047 | 1,084 | 0.56 | 0.34 | 0.92 | 0.56 | 0.34 | 0.92 |
| ivacaftor | 14 | 74,430 | 1,012 | 2,860,104 | 1,026 | 0.55 | 0.33 | 0.92 | 0.55 | 0.33 | 0.92 |
| entecavir | 8 | 74,436 | 595 | 2,860,521 | 603 | 0.55 | 0.28 | 1.08 | 0.55 | 0.28 | 1.08 |
| sitagliptin | 36 | 74,408 | 2,572 | 2,858,544 | 2,608 | 0.55 | 0.39 | 0.76 | 0.55 | 0.39 | 0.76 |
| ezetimibe | 18 | 74,426 | 1,315 | 2,859,801 | 1,333 | 0.54 | 0.34 | 0.86 | 0.54 | 0.34 | 0.86 |
| zoledronic acid | 57 | 74,387 | 4,090 | 2,857,026 | 4,147 | 0.54 | 0.42 | 0.70 | 0.54 | 0.42 | 0.70 |
| perampanel | 13 | 74,431 | 965 | 2,860,151 | 978 | 0.54 | 0.31 | 0.92 | 0.54 | 0.31 | 0.92 |
| fedratinib | 10 | 74,434 | 757 | 2,860,359 | 767 | 0.53 | 0.29 | 0.98 | 0.53 | 0.29 | 0.98 |
| foscarbidopa foslevodopa | 6 | 74,438 | 469 | 2,860,647 | 475 | 0.53 | 0.25 | 1.15 | 0.53 | 0.25 | 1.15 |
| gadoterate meglumine | 15 | 74,429 | 1,128 | 2,859,988 | 1,143 | 0.53 | 0.32 | 0.87 | 0.53 | 0.32 | 0.87 |
| lamivudine | 5 | 74,439 | 400 | 2,860,716 | 405 | 0.53 | 0.23 | 1.22 | 0.53 | 0.23 | 1.22 |
| interferon beta 1b | 12 | 74,432 | 910 | 2,860,206 | 922 | 0.53 | 0.30 | 0.92 | 0.53 | 0.30 | 0.92 |
| glutamine | 13 | 74,431 | 984 | 2,860,132 | 997 | 0.53 | 0.31 | 0.90 | 0.53 | 0.31 | 0.90 |
| venetoclax | 142 | 74,302 | 10,376 | 2,850,740 | 10,518 | 0.53 | 0.45 | 0.62 | 0.53 | 0.45 | 0.62 |
| solifenacin | 7 | 74,437 | 547 | 2,860,569 | 554 | 0.53 | 0.26 | 1.08 | 0.53 | 0.26 | 1.08 |
| pegvisomant | 10 | 74,434 | 772 | 2,860,344 | 782 | 0.52 | 0.28 | 0.96 | 0.52 | 0.28 | 0.96 |
| crizanlizumab tmca | 6 | 74,438 | 478 | 2,860,638 | 484 | 0.52 | 0.24 | 1.13 | 0.52 | 0.24 | 1.13 |
| encorafenib | 30 | 74,414 | 2,246 | 2,858,870 | 2,276 | 0.52 | 0.36 | 0.75 | 0.52 | 0.37 | 0.75 |
| aprepitant | 6 | 74,438 | 479 | 2,860,637 | 485 | 0.52 | 0.24 | 1.13 | 0.52 | 0.24 | 1.13 |
| casirivimab imdevimab | 9 | 74,435 | 703 | 2,860,413 | 712 | 0.52 | 0.27 | 0.98 | 0.52 | 0.27 | 0.98 |
| bedaquiline | 17 | 74,427 | 1,299 | 2,859,817 | 1,316 | 0.52 | 0.32 | 0.83 | 0.52 | 0.32 | 0.83 |
| insulin human | 26 | 74,418 | 2,002 | 2,859,114 | 2,028 | 0.51 | 0.35 | 0.75 | 0.51 | 0.35 | 0.75 |
| incobotulinumtoxina | 5 | 74,439 | 416 | 2,860,700 | 421 | 0.51 | 0.22 | 1.18 | 0.51 | 0.22 | 1.18 |
| canakinumab | 27 | 74,417 | 2,086 | 2,859,030 | 2,113 | 0.51 | 0.35 | 0.74 | 0.51 | 0.35 | 0.74 |
| thalidomide | 25 | 74,419 | 1,941 | 2,859,175 | 1,966 | 0.50 | 0.34 | 0.75 | 0.50 | 0.34 | 0.75 |
| lanadelumab | 12 | 74,432 | 953 | 2,860,163 | 965 | 0.50 | 0.29 | 0.88 | 0.50 | 0.29 | 0.88 |
| tadalafil | 34 | 74,410 | 2,652 | 2,858,464 | 2,686 | 0.50 | 0.36 | 0.70 | 0.50 | 0.36 | 0.70 |
| idelalisib | 8 | 74,436 | 657 | 2,860,459 | 665 | 0.50 | 0.25 | 0.98 | 0.50 | 0.25 | 0.98 |
| eculizumab | 27 | 74,417 | 2,128 | 2,858,988 | 2,155 | 0.50 | 0.34 | 0.72 | 0.50 | 0.34 | 0.72 |
| ondansetron | 15 | 74,429 | 1,201 | 2,859,915 | 1,216 | 0.50 | 0.30 | 0.82 | 0.50 | 0.30 | 0.82 |
| insulin lispro | 33 | 74,411 | 2,616 | 2,858,500 | 2,649 | 0.49 | 0.35 | 0.69 | 0.49 | 0.35 | 0.69 |
| bimekizumab bkzx | 6 | 74,438 | 509 | 2,860,607 | 515 | 0.49 | 0.23 | 1.06 | 0.49 | 0.23 | 1.06 |
| tenofovir alafenamide | 11 | 74,433 | 902 | 2,860,214 | 913 | 0.49 | 0.27 | 0.88 | 0.49 | 0.27 | 0.88 |
| fluconazole | 16 | 74,428 | 1,295 | 2,859,821 | 1,311 | 0.49 | 0.30 | 0.80 | 0.49 | 0.30 | 0.80 |
| tenofovir disoproxil | 46 | 74,398 | 3,677 | 2,857,439 | 3,723 | 0.49 | 0.36 | 0.65 | 0.49 | 0.36 | 0.65 |
| trastuzumab | 56 | 74,388 | 4,490 | 2,856,626 | 4,546 | 0.48 | 0.37 | 0.63 | 0.48 | 0.37 | 0.63 |
| imatinib | 47 | 74,397 | 3,826 | 2,857,290 | 3,873 | 0.48 | 0.36 | 0.63 | 0.48 | 0.36 | 0.64 |
| hydrochlorothiazide irbesartan | 5 | 74,439 | 445 | 2,860,671 | 450 | 0.47 | 0.20 | 1.10 | 0.47 | 0.20 | 1.10 |
| bortezomib | 27 | 74,417 | 2,227 | 2,858,889 | 2,254 | 0.47 | 0.33 | 0.69 | 0.47 | 0.33 | 0.69 |
| pazopanib | 32 | 74,412 | 2,639 | 2,858,477 | 2,671 | 0.47 | 0.33 | 0.67 | 0.47 | 0.33 | 0.67 |
| enasidenib | 19 | 74,425 | 1,594 | 2,859,522 | 1,613 | 0.47 | 0.30 | 0.73 | 0.47 | 0.30 | 0.73 |
| triptorelin pamoate | 7 | 74,437 | 613 | 2,860,503 | 620 | 0.47 | 0.23 | 0.97 | 0.47 | 0.23 | 0.97 |
| ranitidine | 27 | 74,417 | 2,249 | 2,858,867 | 2,276 | 0.47 | 0.32 | 0.68 | 0.47 | 0.32 | 0.68 |
| selumetinib | 5 | 74,439 | 451 | 2,860,665 | 456 | 0.47 | 0.20 | 1.09 | 0.47 | 0.20 | 1.09 |
| dapagliflozin | 38 | 74,406 | 3,165 | 2,857,951 | 3,203 | 0.47 | 0.34 | 0.64 | 0.47 | 0.34 | 0.64 |
| fulvestrant | 29 | 74,415 | 2,426 | 2,858,690 | 2,455 | 0.47 | 0.32 | 0.67 | 0.47 | 0.33 | 0.67 |
| infliximab abda | 8 | 74,436 | 699 | 2,860,417 | 707 | 0.47 | 0.24 | 0.92 | 0.47 | 0.24 | 0.92 |
| bisoprolol | 17 | 74,427 | 1,442 | 2,859,674 | 1,459 | 0.47 | 0.29 | 0.75 | 0.47 | 0.29 | 0.75 |
| osimertinib | 63 | 74,381 | 5,249 | 2,855,867 | 5,312 | 0.46 | 0.36 | 0.59 | 0.46 | 0.36 | 0.60 |
| asciminib | 6 | 74,438 | 541 | 2,860,575 | 547 | 0.46 | 0.21 | 1.00 | 0.46 | 0.21 | 1.00 |
| mycophenolate mofetil | 28 | 74,416 | 2,398 | 2,858,718 | 2,426 | 0.46 | 0.32 | 0.66 | 0.46 | 0.32 | 0.66 |
| entrectinib | 7 | 74,437 | 631 | 2,860,485 | 638 | 0.46 | 0.22 | 0.94 | 0.46 | 0.22 | 0.94 |
| palivizumab | 19 | 74,425 | 1,644 | 2,859,472 | 1,663 | 0.46 | 0.29 | 0.71 | 0.46 | 0.29 | 0.71 |
| pembrolizumab | 83 | 74,361 | 7,058 | 2,854,058 | 7,141 | 0.45 | 0.37 | 0.56 | 0.45 | 0.37 | 0.56 |
| irbesartan | 12 | 74,432 | 1,066 | 2,860,050 | 1,078 | 0.45 | 0.26 | 0.79 | 0.45 | 0.26 | 0.79 |
| dapagliflozin propanediol metformin | 9 | 74,435 | 811 | 2,860,305 | 820 | 0.45 | 0.24 | 0.85 | 0.45 | 0.24 | 0.85 |
| sorafenib | 15 | 74,429 | 1,338 | 2,859,778 | 1,353 | 0.44 | 0.27 | 0.73 | 0.45 | 0.27 | 0.73 |
| erythropoietin | 5 | 74,439 | 482 | 2,860,634 | 487 | 0.44 | 0.19 | 1.02 | 0.44 | 0.19 | 1.02 |
| eliglustat | 6 | 74,438 | 578 | 2,860,538 | 584 | 0.43 | 0.20 | 0.94 | 0.43 | 0.20 | 0.94 |
| edaravone | 6 | 74,438 | 579 | 2,860,537 | 585 | 0.43 | 0.20 | 0.93 | 0.43 | 0.20 | 0.93 |
| darunavir ethanolate | 6 | 74,438 | 580 | 2,860,536 | 586 | 0.43 | 0.20 | 0.93 | 0.43 | 0.20 | 0.93 |
| filgrastim | 11 | 74,433 | 1,031 | 2,860,085 | 1,042 | 0.43 | 0.24 | 0.77 | 0.43 | 0.24 | 0.77 |
| metformin | 60 | 74,384 | 5,446 | 2,855,670 | 5,506 | 0.43 | 0.33 | 0.55 | 0.43 | 0.33 | 0.55 |
| clindamycin | 15 | 74,429 | 1,401 | 2,859,715 | 1,416 | 0.42 | 0.26 | 0.70 | 0.43 | 0.26 | 0.70 |
| trametinib dimethyl sulfoxide | 19 | 74,425 | 1,764 | 2,859,352 | 1,783 | 0.42 | 0.27 | 0.66 | 0.42 | 0.27 | 0.66 |
| cyclophosphamide | 32 | 74,412 | 2,948 | 2,858,168 | 2,980 | 0.42 | 0.30 | 0.60 | 0.42 | 0.30 | 0.60 |
| insulin detemir | 14 | 74,430 | 1,316 | 2,859,800 | 1,330 | 0.42 | 0.25 | 0.71 | 0.42 | 0.25 | 0.71 |
| pertuzumab | 17 | 74,427 | 1,591 | 2,859,525 | 1,608 | 0.42 | 0.26 | 0.68 | 0.42 | 0.26 | 0.68 |
| ramipril | 20 | 74,424 | 1,872 | 2,859,244 | 1,892 | 0.42 | 0.27 | 0.65 | 0.42 | 0.27 | 0.65 |
| deucravacitinib | 8 | 74,436 | 777 | 2,860,339 | 785 | 0.42 | 0.21 | 0.83 | 0.42 | 0.21 | 0.83 |
| esomeprazole | 70 | 74,374 | 6,447 | 2,854,669 | 6,517 | 0.42 | 0.33 | 0.53 | 0.42 | 0.33 | 0.53 |
| nivolumab | 117 | 74,327 | 10,764 | 2,850,352 | 10,881 | 0.42 | 0.35 | 0.50 | 0.42 | 0.35 | 0.50 |
| ustekinumab | 166 | 74,278 | 15,260 | 2,845,856 | 15,426 | 0.42 | 0.36 | 0.49 | 0.42 | 0.36 | 0.49 |
| ado trastuzumab emtansine | 10 | 74,434 | 976 | 2,860,140 | 986 | 0.41 | 0.22 | 0.76 | 0.41 | 0.22 | 0.76 |
| tacrolimus | 49 | 74,395 | 4,602 | 2,856,514 | 4,651 | 0.41 | 0.31 | 0.55 | 0.41 | 0.31 | 0.55 |
| rabeprazole | 7 | 74,437 | 702 | 2,860,414 | 709 | 0.41 | 0.20 | 0.84 | 0.41 | 0.20 | 0.84 |
| binimetinib | 8 | 74,436 | 797 | 2,860,319 | 805 | 0.41 | 0.21 | 0.81 | 0.41 | 0.21 | 0.81 |
| acetaminophen | 55 | 74,389 | 5,226 | 2,855,890 | 5,281 | 0.41 | 0.31 | 0.53 | 0.41 | 0.31 | 0.53 |
| cyclosporine | 26 | 74,418 | 2,522 | 2,858,594 | 2,548 | 0.40 | 0.28 | 0.59 | 0.40 | 0.28 | 0.59 |
| onasemnogene abeparvovec xioi | 10 | 74,434 | 1,006 | 2,860,110 | 1,016 | 0.40 | 0.22 | 0.74 | 0.40 | 0.22 | 0.74 |
| baloxavir marboxil | 5 | 74,439 | 537 | 2,860,579 | 542 | 0.39 | 0.17 | 0.91 | 0.39 | 0.17 | 0.91 |
| carbamazepine | 19 | 74,425 | 1,908 | 2,859,208 | 1,927 | 0.39 | 0.25 | 0.61 | 0.39 | 0.25 | 0.61 |
| amlodipine besylate valsartan | 5 | 74,439 | 538 | 2,860,578 | 543 | 0.39 | 0.17 | 0.91 | 0.39 | 0.17 | 0.91 |
| enalapril | 5 | 74,439 | 540 | 2,860,576 | 545 | 0.39 | 0.17 | 0.91 | 0.39 | 0.17 | 0.91 |
| tobramycin | 11 | 74,433 | 1,144 | 2,859,972 | 1,155 | 0.39 | 0.22 | 0.69 | 0.39 | 0.22 | 0.69 |
| deferasirox | 14 | 74,430 | 1,450 | 2,859,666 | 1,464 | 0.38 | 0.23 | 0.64 | 0.38 | 0.23 | 0.64 |
| tralokinumab ldrm | 8 | 74,436 | 858 | 2,860,258 | 866 | 0.38 | 0.19 | 0.75 | 0.38 | 0.19 | 0.75 |
| valacyclovir | 12 | 74,432 | 1,269 | 2,859,847 | 1,281 | 0.38 | 0.22 | 0.66 | 0.38 | 0.22 | 0.66 |
| mesalamine | 13 | 74,431 | 1,378 | 2,859,738 | 1,391 | 0.38 | 0.22 | 0.64 | 0.38 | 0.22 | 0.64 |
| ertapenem | 7 | 74,437 | 769 | 2,860,347 | 776 | 0.37 | 0.18 | 0.77 | 0.37 | 0.18 | 0.77 |
| spironolactone | 12 | 74,432 | 1,284 | 2,859,832 | 1,296 | 0.37 | 0.21 | 0.65 | 0.37 | 0.21 | 0.65 |
| olmesartan medoxomil | 8 | 74,436 | 875 | 2,860,241 | 883 | 0.37 | 0.19 | 0.73 | 0.37 | 0.19 | 0.73 |
| cephalexin | 5 | 74,439 | 571 | 2,860,545 | 576 | 0.37 | 0.16 | 0.86 | 0.37 | 0.16 | 0.86 |
| dabrafenib | 30 | 74,414 | 3,192 | 2,857,924 | 3,222 | 0.37 | 0.26 | 0.52 | 0.37 | 0.26 | 0.52 |
| ibuprofen | 51 | 74,393 | 5,408 | 2,855,708 | 5,459 | 0.37 | 0.28 | 0.48 | 0.37 | 0.28 | 0.48 |
| valproate | 13 | 74,431 | 1,441 | 2,859,675 | 1,454 | 0.36 | 0.21 | 0.61 | 0.36 | 0.21 | 0.62 |
| daratumumab | 37 | 74,407 | 4,003 | 2,857,113 | 4,040 | 0.36 | 0.26 | 0.50 | 0.36 | 0.26 | 0.50 |
| crizotinib | 17 | 74,427 | 1,872 | 2,859,244 | 1,889 | 0.36 | 0.22 | 0.57 | 0.36 | 0.22 | 0.58 |
| rivaroxaban | 147 | 74,297 | 15,790 | 2,845,326 | 15,937 | 0.36 | 0.30 | 0.42 | 0.36 | 0.31 | 0.42 |
| itraconazole | 5 | 74,439 | 591 | 2,860,525 | 596 | 0.36 | 0.15 | 0.83 | 0.36 | 0.15 | 0.83 |
| cilgavimab tixagevimab | 6 | 74,438 | 701 | 2,860,415 | 707 | 0.36 | 0.16 | 0.77 | 0.36 | 0.16 | 0.77 |
| docetaxel | 31 | 74,413 | 3,443 | 2,857,673 | 3,474 | 0.35 | 0.25 | 0.50 | 0.35 | 0.25 | 0.50 |
| emtricitabine rilpivirine tenofovir disoproxil | 7 | 74,437 | 832 | 2,860,284 | 839 | 0.35 | 0.17 | 0.71 | 0.35 | 0.17 | 0.71 |
| sirolimus | 11 | 74,433 | 1,276 | 2,859,840 | 1,287 | 0.35 | 0.19 | 0.62 | 0.35 | 0.19 | 0.62 |
| elosulfase alfa | 10 | 74,434 | 1,174 | 2,859,942 | 1,184 | 0.34 | 0.19 | 0.63 | 0.34 | 0.19 | 0.63 |
| bevacizumab | 43 | 74,401 | 4,877 | 2,856,239 | 4,920 | 0.34 | 0.25 | 0.46 | 0.34 | 0.25 | 0.46 |
| brentuximab vedotin | 17 | 74,427 | 1,972 | 2,859,144 | 1,989 | 0.34 | 0.21 | 0.55 | 0.34 | 0.21 | 0.55 |
| romiplostim | 6 | 74,438 | 746 | 2,860,370 | 752 | 0.33 | 0.15 | 0.72 | 0.33 | 0.15 | 0.72 |
| sacituzumab govitecan hziy | 6 | 74,438 | 748 | 2,860,368 | 754 | 0.33 | 0.15 | 0.72 | 0.33 | 0.15 | 0.72 |
| lansoprazole | 21 | 74,423 | 2,480 | 2,858,636 | 2,501 | 0.33 | 0.22 | 0.51 | 0.33 | 0.22 | 0.51 |
| ivacaftor lumacaftor | 11 | 74,433 | 1,334 | 2,859,782 | 1,345 | 0.33 | 0.19 | 0.59 | 0.33 | 0.19 | 0.59 |
| dofetilide | 6 | 74,438 | 754 | 2,860,362 | 760 | 0.33 | 0.15 | 0.72 | 0.33 | 0.15 | 0.72 |
| insulin aspart | 30 | 74,414 | 3,595 | 2,857,521 | 3,625 | 0.33 | 0.23 | 0.47 | 0.33 | 0.23 | 0.47 |
| tipiracil trifluridine | 22 | 74,422 | 2,676 | 2,858,440 | 2,698 | 0.32 | 0.21 | 0.49 | 0.32 | 0.21 | 0.49 |
| chloride | 18 | 74,426 | 2,212 | 2,858,904 | 2,230 | 0.32 | 0.20 | 0.51 | 0.32 | 0.20 | 0.51 |
| golimumab | 111 | 74,333 | 13,435 | 2,847,681 | 13,546 | 0.32 | 0.26 | 0.38 | 0.32 | 0.26 | 0.38 |
| darbepoetin alfa | 10 | 74,434 | 1,274 | 2,859,842 | 1,284 | 0.32 | 0.17 | 0.58 | 0.32 | 0.17 | 0.58 |
| sumatriptan | 6 | 74,438 | 813 | 2,860,303 | 819 | 0.31 | 0.14 | 0.66 | 0.31 | 0.14 | 0.66 |
| panitumumab | 10 | 74,434 | 1,331 | 2,859,785 | 1,341 | 0.30 | 0.17 | 0.56 | 0.30 | 0.17 | 0.56 |
| mycophenolate mofetil mycophenolate mofetil | 5 | 74,439 | 708 | 2,860,408 | 713 | 0.30 | 0.13 | 0.69 | 0.30 | 0.13 | 0.69 |
| tafamidis meglumine | 8 | 74,436 | 1,096 | 2,860,020 | 1,104 | 0.30 | 0.15 | 0.58 | 0.30 | 0.15 | 0.59 |
| clozapine | 64 | 74,380 | 8,377 | 2,852,739 | 8,441 | 0.30 | 0.23 | 0.38 | 0.30 | 0.23 | 0.38 |
| hydrochlorothiazide | 7 | 74,437 | 980 | 2,860,136 | 987 | 0.29 | 0.14 | 0.60 | 0.29 | 0.14 | 0.60 |
| ethinyl estradiol levonorgestrel | 5 | 74,439 | 726 | 2,860,390 | 731 | 0.29 | 0.13 | 0.67 | 0.29 | 0.13 | 0.67 |
| fenofibrate | 5 | 74,439 | 738 | 2,860,378 | 743 | 0.29 | 0.12 | 0.66 | 0.29 | 0.12 | 0.66 |
| midostaurin | 5 | 74,439 | 740 | 2,860,376 | 745 | 0.29 | 0.12 | 0.66 | 0.29 | 0.12 | 0.66 |
| erlotinib | 9 | 74,435 | 1,302 | 2,859,814 | 1,311 | 0.28 | 0.15 | 0.53 | 0.28 | 0.15 | 0.53 |
| agalsidase beta | 12 | 74,432 | 1,738 | 2,859,378 | 1,750 | 0.28 | 0.16 | 0.48 | 0.28 | 0.16 | 0.48 |
| amoxicillin | 18 | 74,426 | 2,577 | 2,858,539 | 2,595 | 0.28 | 0.17 | 0.44 | 0.28 | 0.17 | 0.44 |
| ferric carboxymaltose | 12 | 74,432 | 1,756 | 2,859,360 | 1,768 | 0.27 | 0.16 | 0.48 | 0.27 | 0.16 | 0.48 |
| deferiprone | 6 | 74,438 | 928 | 2,860,188 | 934 | 0.27 | 0.12 | 0.58 | 0.27 | 0.12 | 0.58 |
| ixekizumab | 26 | 74,418 | 3,815 | 2,857,301 | 3,841 | 0.27 | 0.18 | 0.39 | 0.27 | 0.18 | 0.39 |
| axicabtagene ciloleucel | 18 | 74,426 | 2,669 | 2,858,447 | 2,687 | 0.27 | 0.17 | 0.42 | 0.27 | 0.17 | 0.42 |
| risdiplam | 6 | 74,438 | 946 | 2,860,170 | 952 | 0.26 | 0.12 | 0.57 | 0.26 | 0.12 | 0.57 |
| rifampin | 6 | 74,438 | 981 | 2,860,135 | 987 | 0.25 | 0.12 | 0.55 | 0.25 | 0.12 | 0.55 |
| warfarin | 9 | 74,435 | 1,473 | 2,859,643 | 1,482 | 0.25 | 0.13 | 0.47 | 0.25 | 0.13 | 0.47 |
| furosemide | 17 | 74,427 | 2,730 | 2,858,386 | 2,747 | 0.25 | 0.15 | 0.39 | 0.25 | 0.15 | 0.39 |
| loperamide | 8 | 74,436 | 1,332 | 2,859,784 | 1,340 | 0.25 | 0.12 | 0.48 | 0.25 | 0.12 | 0.48 |
| pegfilgrastim | 10 | 74,434 | 1,647 | 2,859,469 | 1,657 | 0.24 | 0.13 | 0.45 | 0.24 | 0.13 | 0.45 |
| patiromer | 8 | 74,436 | 1,386 | 2,859,730 | 1,394 | 0.24 | 0.12 | 0.46 | 0.24 | 0.12 | 0.46 |
| linezolid | 13 | 74,431 | 2,229 | 2,858,887 | 2,242 | 0.23 | 0.14 | 0.40 | 0.23 | 0.14 | 0.40 |
| guselkumab | 25 | 74,419 | 4,280 | 2,856,836 | 4,305 | 0.23 | 0.15 | 0.34 | 0.23 | 0.16 | 0.34 |
| durvalumab | 21 | 74,423 | 3,620 | 2,857,496 | 3,641 | 0.23 | 0.15 | 0.35 | 0.23 | 0.15 | 0.35 |
| alglucosidase alfa | 5 | 74,439 | 950 | 2,860,166 | 955 | 0.22 | 0.10 | 0.51 | 0.22 | 0.10 | 0.51 |
| cabozantinib | 6 | 74,438 | 1,141 | 2,859,975 | 1,147 | 0.22 | 0.10 | 0.47 | 0.22 | 0.10 | 0.47 |
| gemcitabine | 11 | 74,433 | 2,051 | 2,859,065 | 2,062 | 0.22 | 0.12 | 0.38 | 0.22 | 0.12 | 0.38 |
| aztreonam lysine | 9 | 74,435 | 1,701 | 2,859,415 | 1,710 | 0.21 | 0.11 | 0.41 | 0.21 | 0.11 | 0.41 |
| carboplatin | 21 | 74,423 | 3,851 | 2,857,265 | 3,872 | 0.21 | 0.14 | 0.33 | 0.21 | 0.14 | 0.33 |
| fluorouracil | 9 | 74,435 | 1,703 | 2,859,413 | 1,712 | 0.21 | 0.11 | 0.41 | 0.21 | 0.11 | 0.41 |
| mycophenolic acid | 6 | 74,438 | 1,169 | 2,859,947 | 1,175 | 0.21 | 0.10 | 0.46 | 0.21 | 0.10 | 0.46 |
| atezolizumab | 32 | 74,412 | 6,006 | 2,855,110 | 6,038 | 0.21 | 0.15 | 0.29 | 0.21 | 0.15 | 0.29 |
| iron sucrose | 5 | 74,439 | 1,018 | 2,860,098 | 1,023 | 0.21 | 0.09 | 0.48 | 0.21 | 0.09 | 0.48 |
| gilteritinib | 6 | 74,438 | 1,255 | 2,859,861 | 1,261 | 0.20 | 0.09 | 0.43 | 0.20 | 0.09 | 0.43 |
| emicizumab kxwh | 8 | 74,436 | 1,672 | 2,859,444 | 1,680 | 0.20 | 0.10 | 0.38 | 0.20 | 0.10 | 0.38 |
| clopidogrel bisulfate | 15 | 74,429 | 3,049 | 2,858,067 | 3,064 | 0.20 | 0.12 | 0.32 | 0.20 | 0.12 | 0.32 |
| irinotecan | 9 | 74,435 | 1,909 | 2,859,207 | 1,918 | 0.19 | 0.10 | 0.36 | 0.19 | 0.10 | 0.36 |
| paclitaxel | 26 | 74,418 | 5,433 | 2,855,683 | 5,459 | 0.19 | 0.13 | 0.27 | 0.19 | 0.13 | 0.27 |
| fam trastuzumab deruxtecan nxki | 7 | 74,437 | 1,546 | 2,859,570 | 1,553 | 0.19 | 0.09 | 0.38 | 0.19 | 0.09 | 0.38 |
| ipilimumab | 13 | 74,431 | 2,791 | 2,858,325 | 2,804 | 0.19 | 0.11 | 0.32 | 0.19 | 0.11 | 0.32 |
| tisagenlecleucel | 8 | 74,436 | 1,779 | 2,859,337 | 1,787 | 0.18 | 0.09 | 0.36 | 0.18 | 0.09 | 0.36 |
| empagliflozin | 20 | 74,424 | 4,357 | 2,856,759 | 4,377 | 0.18 | 0.12 | 0.28 | 0.18 | 0.12 | 0.28 |
| canagliflozin | 11 | 74,433 | 2,487 | 2,858,629 | 2,498 | 0.18 | 0.10 | 0.32 | 0.18 | 0.10 | 0.32 |
| obinutuzumab | 13 | 74,431 | 2,924 | 2,858,192 | 2,937 | 0.18 | 0.10 | 0.30 | 0.18 | 0.10 | 0.30 |
| pentosan polysulfate | 5 | 74,439 | 1,207 | 2,859,909 | 1,212 | 0.17 | 0.08 | 0.40 | 0.18 | 0.08 | 0.40 |
| gefitinib | 5 | 74,439 | 1,412 | 2,859,704 | 1,417 | 0.15 | 0.06 | 0.35 | 0.15 | 0.06 | 0.35 |
| doxorubicin | 8 | 74,436 | 2,370 | 2,858,746 | 2,378 | 0.14 | 0.07 | 0.27 | 0.14 | 0.07 | 0.27 |
| baricitinib | 9 | 74,435 | 2,652 | 2,858,464 | 2,661 | 0.14 | 0.07 | 0.26 | 0.14 | 0.07 | 0.26 |
| temozolomide | 8 | 74,436 | 2,584 | 2,858,532 | 2,592 | 0.13 | 0.06 | 0.25 | 0.13 | 0.06 | 0.25 |
| vancomycin | 8 | 74,436 | 2,617 | 2,858,499 | 2,625 | 0.12 | 0.06 | 0.24 | 0.12 | 0.06 | 0.24 |
| oxaliplatin | 11 | 74,433 | 4,135 | 2,856,981 | 4,146 | 0.11 | 0.06 | 0.19 | 0.11 | 0.06 | 0.19 |
| dabigatran etexilate | 10 | 74,434 | 4,002 | 2,857,114 | 4,012 | 0.10 | 0.05 | 0.18 | 0.10 | 0.06 | 0.18 |
| azacitidine | 6 | 74,438 | 3,300 | 2,857,816 | 3,306 | 0.08 | 0.04 | 0.16 | 0.08 | 0.04 | 0.16 |

Notes: PS analysis. a=insomnia reports with drug; b=insomnia reports without drug; c=non-insomnia reports with drug; d=non-insomnia reports without drug. Continuity correction (0.5) applied to all cells for effect estimation. Inclusion thresholds: a≥5, (a+c)≥50.

## Table S3. Full disproportionality results for insomnia (ANY analysis; PS+SS suspect drugs).

| **Parent systemic drug** | **a** | **b** | **c** | **d** | **PS/SS exposed reports (a+c)** | **ROR** | **ROR_low** | **ROR_high** | **PRR** | **PRR_low** | **PRR_high** |
| --- | --- | --- | --- | --- | --- | --- | --- | --- | --- | --- | --- |
| flumethasone | 48 | 74,396 | 11 | 2,861,105 | 59 | 162.19 | 85.27 | 308.48 | 162.09 | 85.23 | 308.26 |
| isosorbide dinitrate | 180 | 74,264 | 117 | 2,860,999 | 297 | 59.18 | 46.91 | 74.67 | 59.04 | 46.80 | 74.47 |
| glucosamine | 49 | 74,395 | 38 | 2,861,078 | 87 | 49.45 | 32.45 | 75.35 | 49.41 | 32.43 | 75.29 |
| chlorhexidine | 109 | 74,335 | 105 | 2,861,011 | 214 | 39.95 | 30.57 | 52.20 | 39.89 | 30.53 | 52.11 |
| meglumine | 59 | 74,385 | 58 | 2,861,058 | 117 | 39.12 | 27.27 | 56.13 | 39.09 | 27.25 | 56.07 |
| picosulfate | 41 | 74,403 | 59 | 2,861,057 | 100 | 26.82 | 18.04 | 39.87 | 26.81 | 18.03 | 39.84 |
| acetaminophen caffeine codeine | 24 | 74,420 | 37 | 2,861,079 | 61 | 25.12 | 15.09 | 41.79 | 25.11 | 15.09 | 41.78 |
| chlorhexidine | 385 | 74,059 | 599 | 2,860,517 | 984 | 24.84 | 21.85 | 28.23 | 24.71 | 21.75 | 28.08 |
| chlorhexidine isopropyl alcohol | 112 | 74,332 | 193 | 2,860,923 | 305 | 22.38 | 17.73 | 28.23 | 22.34 | 17.71 | 28.19 |
| gold thiomalate | 301 | 74,143 | 578 | 2,860,538 | 879 | 20.11 | 17.49 | 23.12 | 20.03 | 17.43 | 23.02 |
| neomycin | 28 | 74,416 | 56 | 2,861,060 | 84 | 19.39 | 12.36 | 30.43 | 19.39 | 12.36 | 30.41 |
| azd 1222 | 175 | 74,269 | 356 | 2,860,760 | 531 | 18.96 | 15.82 | 22.72 | 18.92 | 15.79 | 22.66 |
| phthalylsulfathiazole | 469 | 73,975 | 973 | 2,860,143 | 1,442 | 18.65 | 16.70 | 20.82 | 18.54 | 16.61 | 20.69 |
| isosorbide mononitrate | 190 | 74,254 | 402 | 2,860,714 | 592 | 18.23 | 15.34 | 21.67 | 18.19 | 15.31 | 21.61 |
| hydrocortisone butyrate | 37 | 74,407 | 80 | 2,861,036 | 117 | 17.91 | 12.16 | 26.39 | 17.90 | 12.15 | 26.38 |
| flumethasone pivalate | 194 | 74,250 | 429 | 2,860,687 | 623 | 17.45 | 14.73 | 20.67 | 17.40 | 14.69 | 20.61 |
| cetirizine pseudoephedrine | 191 | 74,253 | 439 | 2,860,677 | 630 | 16.79 | 14.16 | 19.89 | 16.75 | 14.13 | 19.84 |
| cortisone | 325 | 74,119 | 825 | 2,860,291 | 1,150 | 15.22 | 13.38 | 17.30 | 15.15 | 13.33 | 17.22 |
| isopropyl alcohol | 26 | 74,418 | 68 | 2,861,048 | 94 | 14.87 | 9.50 | 23.29 | 14.87 | 9.50 | 23.28 |
| diclofenac diethylamine | 28 | 74,416 | 77 | 2,861,039 | 105 | 14.14 | 9.20 | 21.72 | 14.13 | 9.20 | 21.71 |
| sulfamethoxazole | 68 | 74,376 | 188 | 2,860,928 | 256 | 13.98 | 10.60 | 18.43 | 13.97 | 10.59 | 18.41 |
| tyrothricin | 17 | 74,427 | 48 | 2,861,068 | 65 | 13.87 | 8.03 | 23.96 | 13.87 | 8.03 | 23.95 |
| oxybate | 84 | 74,360 | 252 | 2,860,864 | 336 | 12.88 | 10.06 | 16.47 | 12.86 | 10.05 | 16.45 |
| istradefylline | 55 | 74,389 | 168 | 2,860,948 | 223 | 12.67 | 9.35 | 17.16 | 12.66 | 9.35 | 17.14 |
| pentazocine | 12 | 74,432 | 39 | 2,861,077 | 51 | 12.16 | 6.44 | 22.98 | 12.16 | 6.44 | 22.97 |
| diclofenac | 135 | 74,309 | 432 | 2,860,684 | 567 | 12.06 | 9.94 | 14.63 | 12.04 | 9.93 | 14.60 |
| alendronic acid | 337 | 74,107 | 1,104 | 2,860,012 | 1,441 | 11.79 | 10.44 | 13.32 | 11.74 | 10.40 | 13.26 |
| niraparib | 607 | 73,837 | 2,038 | 2,859,078 | 2,645 | 11.54 | 10.54 | 12.64 | 11.45 | 10.46 | 12.54 |
| nitrofurazone | 17 | 74,427 | 58 | 2,861,058 | 75 | 11.50 | 6.74 | 19.62 | 11.50 | 6.74 | 19.61 |
| desoximetasone | 607 | 73,837 | 2,075 | 2,859,041 | 2,682 | 11.33 | 10.35 | 12.41 | 11.25 | 10.28 | 12.31 |
| fosaprepitant | 30 | 74,414 | 107 | 2,861,009 | 137 | 10.91 | 7.30 | 16.31 | 10.90 | 7.29 | 16.30 |
| daridorexant | 12 | 74,432 | 44 | 2,861,072 | 56 | 10.80 | 5.77 | 20.22 | 10.80 | 5.76 | 20.22 |
| conestat alfa | 15 | 74,429 | 56 | 2,861,060 | 71 | 10.55 | 6.01 | 18.50 | 10.54 | 6.01 | 18.49 |
| trofinetide | 93 | 74,351 | 341 | 2,860,775 | 434 | 10.53 | 8.38 | 13.24 | 10.52 | 8.37 | 13.23 |
| thymol | 15 | 74,429 | 58 | 2,861,058 | 73 | 10.18 | 5.82 | 17.83 | 10.18 | 5.82 | 17.82 |
| viloxazine | 27 | 74,417 | 105 | 2,861,011 | 132 | 10.02 | 6.59 | 15.25 | 10.02 | 6.58 | 15.24 |
| pramipexole dihydrochloride | 43 | 74,401 | 174 | 2,860,942 | 217 | 9.59 | 6.88 | 13.36 | 9.58 | 6.87 | 13.35 |
| donepezil | 12 | 74,432 | 50 | 2,861,066 | 62 | 9.51 | 5.12 | 17.67 | 9.51 | 5.12 | 17.67 |
| loratadine pseudoephedrine | 129 | 74,315 | 532 | 2,860,584 | 661 | 9.36 | 7.72 | 11.34 | 9.35 | 7.71 | 11.32 |
| flibanserin | 112 | 74,332 | 470 | 2,860,646 | 582 | 9.20 | 7.49 | 11.30 | 9.19 | 7.48 | 11.29 |
| diphenhydramine naproxen | 29 | 74,415 | 124 | 2,860,992 | 153 | 9.11 | 6.10 | 13.61 | 9.11 | 6.10 | 13.60 |
| hydrocortisone | 33 | 74,411 | 151 | 2,860,965 | 184 | 8.50 | 5.85 | 12.36 | 8.50 | 5.85 | 12.35 |
| levoketoconazole | 14 | 74,430 | 66 | 2,861,050 | 80 | 8.38 | 4.75 | 14.79 | 8.38 | 4.75 | 14.79 |
| bisoprolol hydrochlorothiazide | 28 | 74,416 | 131 | 2,860,985 | 159 | 8.33 | 5.56 | 12.49 | 8.33 | 5.56 | 12.49 |
| dietary supplement herbals | 27 | 74,417 | 130 | 2,860,986 | 157 | 8.10 | 5.37 | 12.22 | 8.10 | 5.37 | 12.22 |
| acetaminophen oxycodone terephthalate | 39 | 74,405 | 187 | 2,860,929 | 226 | 8.10 | 5.75 | 11.42 | 8.10 | 5.75 | 11.41 |
| leflunomide | 1,059 | 73,385 | 5,208 | 2,855,908 | 6,267 | 7.92 | 7.41 | 8.46 | 7.82 | 7.32 | 8.35 |
| guaifenesin pseudoephedrine | 25 | 74,419 | 126 | 2,860,990 | 151 | 7.75 | 5.06 | 11.86 | 7.75 | 5.06 | 11.85 |
| hyaluronidase | 8 | 74,436 | 43 | 2,861,073 | 51 | 7.51 | 3.60 | 15.66 | 7.51 | 3.60 | 15.66 |
| pimavanserin | 605 | 73,839 | 3,159 | 2,857,957 | 3,764 | 7.42 | 6.80 | 8.09 | 7.37 | 6.75 | 8.03 |
| ropeginterferon alfa 2b njft | 34 | 74,410 | 182 | 2,860,934 | 216 | 7.27 | 5.05 | 10.46 | 7.27 | 5.05 | 10.45 |
| pseudoephedrine | 133 | 74,311 | 714 | 2,860,402 | 847 | 7.19 | 5.98 | 8.65 | 7.18 | 5.97 | 8.64 |
| pramipexole dihydrochloride | 10 | 74,434 | 56 | 2,861,060 | 66 | 7.14 | 3.70 | 13.80 | 7.14 | 3.70 | 13.80 |
| alendronate | 686 | 73,758 | 3,733 | 2,857,383 | 4,419 | 7.12 | 6.56 | 7.73 | 7.07 | 6.52 | 7.66 |
| benzyl alcohol etanercept | 116 | 74,328 | 651 | 2,860,465 | 767 | 6.88 | 5.65 | 8.38 | 6.87 | 5.64 | 8.37 |
| cetirizine | 657 | 73,787 | 3,713 | 2,857,403 | 4,370 | 6.86 | 6.31 | 7.45 | 6.80 | 6.27 | 7.39 |
| etanercept szzs | 440 | 74,004 | 2,491 | 2,858,625 | 2,931 | 6.83 | 6.17 | 7.56 | 6.79 | 6.14 | 7.52 |
| hyaluronidase human recombinant | 23 | 74,421 | 134 | 2,860,982 | 157 | 6.72 | 4.33 | 10.41 | 6.71 | 4.33 | 10.41 |
| folic acid | 479 | 73,965 | 2,857 | 2,858,259 | 3,336 | 6.48 | 5.89 | 7.14 | 6.45 | 5.86 | 7.10 |
| sulfasalazine | 660 | 73,784 | 3,982 | 2,857,134 | 4,642 | 6.42 | 5.91 | 6.98 | 6.37 | 5.87 | 6.92 |
| omalizumab | 1,457 | 72,987 | 8,905 | 2,852,211 | 10,362 | 6.40 | 6.05 | 6.76 | 6.29 | 5.95 | 6.64 |
| alendronate cholecalciferol | 60 | 74,384 | 367 | 2,860,749 | 427 | 6.33 | 4.82 | 8.31 | 6.33 | 4.82 | 8.30 |
| bupropion naltrexone | 164 | 74,280 | 1,003 | 2,860,113 | 1,167 | 6.31 | 5.35 | 7.44 | 6.30 | 5.34 | 7.43 |
| ziprasidone | 13 | 74,431 | 82 | 2,861,034 | 95 | 6.29 | 3.54 | 11.18 | 6.29 | 3.54 | 11.18 |
| aprepitant | 225 | 74,219 | 1,405 | 2,859,711 | 1,630 | 6.18 | 5.37 | 7.12 | 6.17 | 5.36 | 7.10 |
| caffeine | 37 | 74,407 | 239 | 2,860,877 | 276 | 6.02 | 4.27 | 8.49 | 6.02 | 4.27 | 8.49 |
| estrogens medroxyprogesterone | 37 | 74,407 | 239 | 2,860,877 | 276 | 6.02 | 4.27 | 8.49 | 6.02 | 4.27 | 8.49 |
| dexmethylphenidate serdexmethylphenidate chloride | 16 | 74,428 | 105 | 2,861,011 | 121 | 6.01 | 3.58 | 10.10 | 6.01 | 3.58 | 10.10 |
| acetaminophen caffeine dihydrocodeine | 14 | 74,430 | 93 | 2,861,023 | 107 | 5.96 | 3.43 | 10.37 | 5.96 | 3.43 | 10.36 |
| dupilumab | 1,547 | 72,897 | 10,447 | 2,850,669 | 11,994 | 5.79 | 5.49 | 6.11 | 5.69 | 5.40 | 6.00 |
| tasimelteon | 12 | 74,432 | 83 | 2,861,033 | 95 | 5.75 | 3.18 | 10.43 | 5.75 | 3.18 | 10.42 |
| pramipexole | 53 | 74,391 | 372 | 2,860,744 | 425 | 5.52 | 4.15 | 7.36 | 5.52 | 4.14 | 7.35 |
| fexofenadine pseudoephedrine | 10 | 74,434 | 73 | 2,861,043 | 83 | 5.49 | 2.88 | 10.48 | 5.49 | 2.88 | 10.48 |
| levothyroxine liothyronine | 46 | 74,398 | 330 | 2,860,786 | 376 | 5.41 | 3.98 | 7.35 | 5.41 | 3.98 | 7.35 |
| hydroxychloroquine | 795 | 73,649 | 5,709 | 2,855,407 | 6,504 | 5.40 | 5.01 | 5.82 | 5.35 | 4.97 | 5.77 |
| oxymorphone | 11 | 74,433 | 84 | 2,861,032 | 95 | 5.23 | 2.83 | 9.69 | 5.23 | 2.82 | 9.68 |
| suvorexant | 43 | 74,401 | 321 | 2,860,795 | 364 | 5.20 | 3.79 | 7.14 | 5.20 | 3.79 | 7.14 |
| phentermine topiramate | 76 | 74,368 | 567 | 2,860,549 | 643 | 5.19 | 4.08 | 6.58 | 5.18 | 4.08 | 6.58 |
| almotriptan | 7 | 74,437 | 56 | 2,861,060 | 63 | 5.10 | 2.38 | 10.93 | 5.10 | 2.38 | 10.93 |
| adalimumab isopropyl alcohol | 189 | 74,255 | 1,440 | 2,859,676 | 1,629 | 5.07 | 4.35 | 5.90 | 5.06 | 4.35 | 5.88 |
| amantadine | 123 | 74,321 | 940 | 2,860,176 | 1,063 | 5.05 | 4.19 | 6.10 | 5.05 | 4.18 | 6.09 |
| valbenazine | 175 | 74,269 | 1,364 | 2,859,752 | 1,539 | 4.95 | 4.23 | 5.80 | 4.94 | 4.22 | 5.78 |
| celecoxib | 425 | 74,019 | 3,341 | 2,857,775 | 3,766 | 4.92 | 4.44 | 5.44 | 4.89 | 4.43 | 5.41 |
| lumateperone | 79 | 74,365 | 623 | 2,860,493 | 702 | 4.90 | 3.88 | 6.19 | 4.90 | 3.88 | 6.19 |
| cyanocobalamin | 61 | 74,383 | 486 | 2,860,630 | 547 | 4.86 | 3.73 | 6.34 | 4.86 | 3.73 | 6.33 |
| fenoterol ipratropium bromide | 6 | 74,438 | 51 | 2,861,065 | 57 | 4.85 | 2.15 | 10.97 | 4.85 | 2.15 | 10.97 |
| burosumab twza | 28 | 74,416 | 226 | 2,860,890 | 254 | 4.84 | 3.28 | 7.14 | 4.84 | 3.28 | 7.14 |
| corticotropin | 150 | 74,294 | 1,204 | 2,859,912 | 1,354 | 4.81 | 4.06 | 5.70 | 4.80 | 4.05 | 5.69 |
| cenobamate | 52 | 74,392 | 421 | 2,860,695 | 473 | 4.79 | 3.59 | 6.38 | 4.79 | 3.59 | 6.38 |
| amlodipine | 9 | 74,435 | 76 | 2,861,040 | 85 | 4.77 | 2.43 | 9.37 | 4.77 | 2.43 | 9.37 |
| ramelteon | 7 | 74,437 | 60 | 2,861,056 | 67 | 4.76 | 2.23 | 10.18 | 4.76 | 2.23 | 10.17 |
| relugolix | 136 | 74,308 | 1,109 | 2,860,007 | 1,245 | 4.74 | 3.96 | 5.66 | 4.73 | 3.96 | 5.65 |
| milnacipran | 16 | 74,428 | 134 | 2,860,982 | 150 | 4.72 | 2.83 | 7.86 | 4.71 | 2.83 | 7.86 |
| finasteride | 191 | 74,253 | 1,564 | 2,859,552 | 1,755 | 4.71 | 4.06 | 5.48 | 4.70 | 4.05 | 5.46 |
| estrogens | 69 | 74,375 | 569 | 2,860,547 | 638 | 4.69 | 3.66 | 6.02 | 4.69 | 3.66 | 6.02 |
| avapritinib | 233 | 74,211 | 1,921 | 2,859,195 | 2,154 | 4.68 | 4.09 | 5.36 | 4.67 | 4.08 | 5.35 |
| eszopiclone | 39 | 74,405 | 329 | 2,860,787 | 368 | 4.61 | 3.31 | 6.41 | 4.61 | 3.31 | 6.41 |
| phenylephrine | 43 | 74,401 | 364 | 2,860,752 | 407 | 4.59 | 3.35 | 6.28 | 4.59 | 3.35 | 6.28 |
| althiazide | 5 | 74,439 | 46 | 2,861,070 | 51 | 4.55 | 1.88 | 11.00 | 4.55 | 1.88 | 11.00 |
| olanzapine samidorphan l | 49 | 74,395 | 419 | 2,860,697 | 468 | 4.54 | 3.38 | 6.09 | 4.53 | 3.38 | 6.09 |
| naltrexone | 362 | 74,082 | 3,082 | 2,858,034 | 3,444 | 4.54 | 4.07 | 5.06 | 4.52 | 4.05 | 5.04 |
| lurasidone | 304 | 74,140 | 2,599 | 2,858,517 | 2,903 | 4.52 | 4.01 | 5.09 | 4.50 | 4.00 | 5.07 |
| abaloparatide | 191 | 74,253 | 1,662 | 2,859,454 | 1,853 | 4.44 | 3.82 | 5.15 | 4.43 | 3.81 | 5.14 |
| mometasone furoate olopatadine | 6 | 74,438 | 56 | 2,861,060 | 62 | 4.42 | 1.96 | 9.96 | 4.42 | 1.96 | 9.96 |
| vilazodone | 40 | 74,404 | 353 | 2,860,763 | 393 | 4.41 | 3.18 | 6.10 | 4.40 | 3.18 | 6.09 |
| fosaprepitant dimeglumine | 16 | 74,428 | 144 | 2,860,972 | 160 | 4.39 | 2.64 | 7.30 | 4.39 | 2.64 | 7.30 |
| hydrocortisone | 317 | 74,127 | 2,830 | 2,858,286 | 3,147 | 4.33 | 3.85 | 4.86 | 4.31 | 3.84 | 4.84 |
| formoterol mometasone furoate | 20 | 74,424 | 182 | 2,860,934 | 202 | 4.32 | 2.74 | 6.82 | 4.32 | 2.73 | 6.81 |
| montelukast | 403 | 74,041 | 3,610 | 2,857,506 | 4,013 | 4.31 | 3.89 | 4.78 | 4.30 | 3.88 | 4.76 |
| apremilast | 730 | 73,714 | 6,770 | 2,854,346 | 7,500 | 4.18 | 3.87 | 4.51 | 4.15 | 3.84 | 4.47 |
| gadopentetate dimeglumine | 5 | 74,439 | 51 | 2,861,065 | 56 | 4.10 | 1.70 | 9.89 | 4.10 | 1.70 | 9.89 |
| rucaparib | 126 | 74,318 | 1,188 | 2,859,928 | 1,314 | 4.10 | 3.41 | 4.92 | 4.09 | 3.41 | 4.91 |
| hyaluronidase human recombinant rituximab | 23 | 74,421 | 224 | 2,860,892 | 247 | 4.02 | 2.63 | 6.16 | 4.02 | 2.63 | 6.15 |
| pregabalin | 868 | 73,576 | 8,444 | 2,852,672 | 9,312 | 3.99 | 3.72 | 4.28 | 3.95 | 3.69 | 4.24 |
| tofacitinib | 1,302 | 73,142 | 12,978 | 2,848,138 | 14,280 | 3.91 | 3.69 | 4.14 | 3.86 | 3.65 | 4.08 |
| carbidopa levodopa | 191 | 74,253 | 1,887 | 2,859,229 | 2,078 | 3.91 | 3.37 | 4.53 | 3.90 | 3.36 | 4.52 |
| bilastine | 19 | 74,425 | 192 | 2,860,924 | 211 | 3.89 | 2.44 | 6.20 | 3.89 | 2.44 | 6.20 |
| mefenamic acid | 6 | 74,438 | 64 | 2,861,052 | 70 | 3.87 | 1.73 | 8.68 | 3.87 | 1.73 | 8.68 |
| diclofenac | 371 | 74,073 | 3,767 | 2,857,349 | 4,138 | 3.80 | 3.42 | 4.23 | 3.79 | 3.41 | 4.21 |
| infliximab dyyb | 116 | 74,328 | 1,186 | 2,859,930 | 1,302 | 3.78 | 3.12 | 4.57 | 3.77 | 3.12 | 4.56 |
| bazedoxifene estrogens | 8 | 74,436 | 86 | 2,861,030 | 94 | 3.78 | 1.87 | 7.64 | 3.78 | 1.87 | 7.64 |
| sofosbuvir velpatasvir | 265 | 74,179 | 2,712 | 2,858,404 | 2,977 | 3.77 | 3.32 | 4.28 | 3.76 | 3.32 | 4.27 |
| deutetrabenazine | 97 | 74,347 | 997 | 2,860,119 | 1,094 | 3.76 | 3.05 | 4.63 | 3.76 | 3.05 | 4.62 |
| felodipine | 23 | 74,421 | 240 | 2,860,876 | 263 | 3.76 | 2.46 | 5.74 | 3.76 | 2.46 | 5.74 |
| amphetamine aspartate amphetamine dextroamphetamine saccharate dextroamphetamine | 132 | 74,312 | 1,358 | 2,859,758 | 1,490 | 3.75 | 3.14 | 4.49 | 3.75 | 3.14 | 4.48 |
| fezolinetant | 14 | 74,430 | 148 | 2,860,968 | 162 | 3.75 | 2.19 | 6.44 | 3.75 | 2.19 | 6.43 |
| tocilizumab | 1,281 | 73,163 | 13,417 | 2,847,699 | 14,698 | 3.72 | 3.51 | 3.94 | 3.67 | 3.47 | 3.89 |
| beclomethasone | 6 | 74,438 | 67 | 2,861,049 | 73 | 3.70 | 1.65 | 8.28 | 3.70 | 1.65 | 8.28 |
| certolizumab pegol | 1,223 | 73,221 | 12,936 | 2,848,180 | 14,159 | 3.68 | 3.47 | 3.90 | 3.63 | 3.43 | 3.85 |
| acetaminophen oxycodone | 345 | 74,099 | 3,629 | 2,857,487 | 3,974 | 3.67 | 3.29 | 4.10 | 3.66 | 3.28 | 4.08 |
| elacestrant | 57 | 74,387 | 606 | 2,860,510 | 663 | 3.65 | 2.78 | 4.78 | 3.64 | 2.78 | 4.77 |
| tiotropium | 9 | 74,435 | 100 | 2,861,016 | 109 | 3.63 | 1.87 | 7.07 | 3.63 | 1.87 | 7.07 |
| perindopril arginine | 7 | 74,437 | 79 | 2,861,037 | 86 | 3.63 | 1.71 | 7.67 | 3.63 | 1.71 | 7.67 |
| clopidogrel besilate | 5 | 74,439 | 58 | 2,861,058 | 63 | 3.61 | 1.51 | 8.66 | 3.61 | 1.51 | 8.66 |
| desvenlafaxine | 77 | 74,367 | 835 | 2,860,281 | 912 | 3.57 | 2.83 | 4.50 | 3.56 | 2.83 | 4.50 |
| elagolix | 85 | 74,359 | 923 | 2,860,193 | 1,008 | 3.56 | 2.85 | 4.44 | 3.56 | 2.85 | 4.44 |
| estradiol progesterone | 9 | 74,435 | 104 | 2,861,012 | 113 | 3.49 | 1.80 | 6.79 | 3.49 | 1.80 | 6.79 |
| etanercept | 1,224 | 73,220 | 13,857 | 2,847,259 | 15,081 | 3.44 | 3.24 | 3.64 | 3.40 | 3.20 | 3.60 |
| cariprazine | 81 | 74,363 | 915 | 2,860,201 | 996 | 3.42 | 2.73 | 4.30 | 3.42 | 2.73 | 4.29 |
| varicella zoster virus strain oka merck live antigen | 17 | 74,427 | 199 | 2,860,917 | 216 | 3.37 | 2.07 | 5.50 | 3.37 | 2.07 | 5.50 |
| ocrelizumab | 659 | 73,785 | 7,569 | 2,853,547 | 8,228 | 3.37 | 3.11 | 3.65 | 3.35 | 3.09 | 3.62 |
| mifepristone | 76 | 74,368 | 881 | 2,860,235 | 957 | 3.34 | 2.64 | 4.22 | 3.34 | 2.64 | 4.21 |
| phenelzine | 7 | 74,437 | 86 | 2,861,030 | 93 | 3.33 | 1.58 | 7.03 | 3.33 | 1.58 | 7.03 |
| daclatasvir | 9 | 74,435 | 110 | 2,861,006 | 119 | 3.30 | 1.70 | 6.41 | 3.30 | 1.70 | 6.41 |
| alemtuzumab | 276 | 74,168 | 3,230 | 2,857,886 | 3,506 | 3.30 | 2.92 | 3.73 | 3.29 | 2.91 | 3.72 |
| abatacept | 1,221 | 73,223 | 14,437 | 2,846,679 | 15,658 | 3.29 | 3.10 | 3.49 | 3.25 | 3.07 | 3.45 |
| elexacaftor ivacaftor tezacaftor | 283 | 74,161 | 3,322 | 2,857,794 | 3,605 | 3.29 | 2.91 | 3.71 | 3.28 | 2.91 | 3.70 |
| estradiol | 126 | 74,318 | 1,483 | 2,859,633 | 1,609 | 3.28 | 2.74 | 3.93 | 3.28 | 2.73 | 3.93 |
| simvastatin | 266 | 74,178 | 3,147 | 2,857,969 | 3,413 | 3.26 | 2.88 | 3.70 | 3.25 | 2.87 | 3.69 |
| estazolam | 5 | 74,439 | 65 | 2,861,051 | 70 | 3.23 | 1.35 | 7.70 | 3.23 | 1.35 | 7.70 |
| dalfampridine | 250 | 74,194 | 3,015 | 2,858,101 | 3,265 | 3.20 | 2.81 | 3.64 | 3.19 | 2.81 | 3.63 |
| mepolizumab | 495 | 73,949 | 6,021 | 2,855,095 | 6,516 | 3.18 | 2.90 | 3.48 | 3.16 | 2.89 | 3.46 |
| opicapone | 19 | 74,425 | 236 | 2,860,880 | 255 | 3.17 | 2.00 | 5.03 | 3.17 | 2.00 | 5.03 |
| candesartan | 175 | 74,269 | 2,141 | 2,858,975 | 2,316 | 3.15 | 2.70 | 3.68 | 3.15 | 2.70 | 3.67 |
| hydroxyprogesterone caproate | 69 | 74,375 | 856 | 2,860,260 | 925 | 3.12 | 2.44 | 3.99 | 3.12 | 2.44 | 3.98 |
| ciclesonide | 108 | 74,336 | 1,338 | 2,859,778 | 1,446 | 3.12 | 2.56 | 3.79 | 3.12 | 2.56 | 3.79 |
| etizolam | 9 | 74,435 | 117 | 2,860,999 | 126 | 3.11 | 1.60 | 6.02 | 3.11 | 1.60 | 6.02 |
| thyroid porcine | 25 | 74,419 | 317 | 2,860,799 | 342 | 3.09 | 2.06 | 4.62 | 3.09 | 2.06 | 4.62 |
| naproxen pseudoephedrine | 9 | 74,435 | 118 | 2,860,998 | 127 | 3.08 | 1.59 | 5.97 | 3.08 | 1.59 | 5.97 |
| bupropion dextromethorphan | 25 | 74,419 | 318 | 2,860,798 | 343 | 3.08 | 2.06 | 4.61 | 3.08 | 2.06 | 4.61 |
| efgartigimod alfa fcab | 24 | 74,420 | 309 | 2,860,807 | 333 | 3.04 | 2.02 | 4.59 | 3.04 | 2.02 | 4.59 |
| blonanserin | 5 | 74,439 | 69 | 2,861,047 | 74 | 3.04 | 1.28 | 7.25 | 3.04 | 1.28 | 7.25 |
| amoxicillin omeprazole rifabutin | 6 | 74,438 | 82 | 2,861,034 | 88 | 3.03 | 1.36 | 6.73 | 3.03 | 1.36 | 6.73 |
| sofosbuvir | 24 | 74,420 | 311 | 2,860,805 | 335 | 3.02 | 2.00 | 4.56 | 3.02 | 2.00 | 4.56 |
| solriamfetol | 12 | 74,432 | 159 | 2,860,957 | 171 | 3.01 | 1.69 | 5.36 | 3.01 | 1.69 | 5.36 |
| teriflunomide | 338 | 74,106 | 4,386 | 2,856,730 | 4,724 | 2.97 | 2.66 | 3.32 | 2.97 | 2.66 | 3.31 |
| fenfluramine | 57 | 74,387 | 743 | 2,860,373 | 800 | 2.97 | 2.27 | 3.89 | 2.97 | 2.27 | 3.89 |
| levothyroxine | 445 | 73,999 | 5,811 | 2,855,305 | 6,256 | 2.96 | 2.69 | 3.26 | 2.95 | 2.68 | 3.24 |
| liothyronine | 20 | 74,424 | 266 | 2,860,850 | 286 | 2.96 | 1.89 | 4.63 | 2.96 | 1.89 | 4.63 |
| varenicline | 114 | 74,330 | 1,495 | 2,859,621 | 1,609 | 2.95 | 2.44 | 3.56 | 2.94 | 2.43 | 3.56 |
| ofatumumab | 301 | 74,143 | 3,946 | 2,857,170 | 4,247 | 2.94 | 2.62 | 3.31 | 2.94 | 2.61 | 3.30 |
| cefpodoxime | 6 | 74,438 | 85 | 2,861,031 | 91 | 2.92 | 1.32 | 6.49 | 2.92 | 1.32 | 6.49 |
| acetaminophen hydrocodone | 300 | 74,144 | 3,973 | 2,857,143 | 4,273 | 2.91 | 2.59 | 3.28 | 2.91 | 2.59 | 3.27 |
| pirfenidone | 273 | 74,171 | 3,624 | 2,857,492 | 3,897 | 2.91 | 2.57 | 3.29 | 2.90 | 2.57 | 3.28 |
| hydrocodone | 79 | 74,365 | 1,055 | 2,860,061 | 1,134 | 2.90 | 2.31 | 3.64 | 2.89 | 2.30 | 3.64 |
| burosumab | 10 | 74,434 | 143 | 2,860,973 | 153 | 2.81 | 1.50 | 5.26 | 2.81 | 1.50 | 5.26 |
| temazepam | 25 | 74,419 | 349 | 2,860,767 | 374 | 2.80 | 1.88 | 4.19 | 2.80 | 1.88 | 4.19 |
| osilodrostat | 13 | 74,431 | 185 | 2,860,931 | 198 | 2.80 | 1.61 | 4.86 | 2.80 | 1.61 | 4.86 |
| methylphenidate | 218 | 74,226 | 3,024 | 2,858,092 | 3,242 | 2.78 | 2.42 | 3.19 | 2.78 | 2.42 | 3.18 |
| hydromorphone | 273 | 74,171 | 3,808 | 2,857,308 | 4,081 | 2.77 | 2.45 | 3.13 | 2.76 | 2.44 | 3.12 |
| diphenhydramine ibuprofen | 7 | 74,437 | 105 | 2,861,011 | 112 | 2.73 | 1.30 | 5.73 | 2.73 | 1.30 | 5.73 |
| doconexent icosapent phosphatidyl serine | 6 | 74,438 | 91 | 2,861,025 | 97 | 2.73 | 1.23 | 6.05 | 2.73 | 1.23 | 6.05 |
| cannabidiol | 113 | 74,331 | 1,634 | 2,859,482 | 1,747 | 2.67 | 2.21 | 3.23 | 2.67 | 2.21 | 3.23 |
| doxycycline hyclate | 8 | 74,436 | 122 | 2,860,994 | 130 | 2.67 | 1.33 | 5.35 | 2.67 | 1.33 | 5.34 |
| baclofen | 99 | 74,345 | 1,437 | 2,859,679 | 1,536 | 2.66 | 2.17 | 3.26 | 2.66 | 2.17 | 3.26 |
| dichlorphenamide | 40 | 74,404 | 589 | 2,860,527 | 629 | 2.64 | 1.92 | 3.63 | 2.64 | 1.92 | 3.63 |
| lemborexant | 12 | 74,432 | 182 | 2,860,934 | 194 | 2.63 | 1.48 | 4.67 | 2.63 | 1.48 | 4.67 |
| elasomeran | 22 | 74,422 | 330 | 2,860,786 | 352 | 2.62 | 1.71 | 4.01 | 2.62 | 1.71 | 4.01 |
| doxycycline hyclate | 42 | 74,402 | 625 | 2,860,491 | 667 | 2.61 | 1.91 | 3.56 | 2.61 | 1.91 | 3.56 |
| givosiran | 10 | 74,434 | 155 | 2,860,961 | 165 | 2.60 | 1.39 | 4.85 | 2.60 | 1.39 | 4.85 |
| setmelanotide | 5 | 74,439 | 81 | 2,861,035 | 86 | 2.59 | 1.09 | 6.15 | 2.59 | 1.09 | 6.15 |
| lactulose | 38 | 74,406 | 571 | 2,860,545 | 609 | 2.59 | 1.87 | 3.59 | 2.59 | 1.87 | 3.59 |
| ledipasvir sofosbuvir | 70 | 74,374 | 1,049 | 2,860,067 | 1,119 | 2.58 | 2.03 | 3.29 | 2.58 | 2.03 | 3.29 |
| budesonide formoterol | 106 | 74,338 | 1,591 | 2,859,525 | 1,697 | 2.57 | 2.12 | 3.13 | 2.57 | 2.11 | 3.13 |
| prednisone | 962 | 73,482 | 14,550 | 2,846,566 | 15,512 | 2.56 | 2.40 | 2.74 | 2.54 | 2.38 | 2.71 |
| mogamulizumab | 7 | 74,437 | 112 | 2,861,004 | 119 | 2.56 | 1.22 | 5.37 | 2.56 | 1.22 | 5.37 |
| covid 19 vaccine nos | 27 | 74,417 | 414 | 2,860,702 | 441 | 2.55 | 1.73 | 3.75 | 2.55 | 1.73 | 3.75 |
| roflumilast | 45 | 74,399 | 686 | 2,860,430 | 731 | 2.55 | 1.89 | 3.44 | 2.55 | 1.89 | 3.44 |
| adalimumab | 2,640 | 71,804 | 40,894 | 2,820,222 | 43,534 | 2.54 | 2.44 | 2.64 | 2.48 | 2.39 | 2.58 |
| dicyclomine | 8 | 74,436 | 129 | 2,860,987 | 137 | 2.52 | 1.26 | 5.05 | 2.52 | 1.26 | 5.05 |
| golimumab | 1,158 | 73,286 | 17,828 | 2,843,288 | 18,986 | 2.52 | 2.37 | 2.68 | 2.50 | 2.35 | 2.65 |
| buprenorphine | 219 | 74,225 | 3,357 | 2,857,759 | 3,576 | 2.52 | 2.20 | 2.89 | 2.51 | 2.19 | 2.88 |
| methotrexate | 1,232 | 73,212 | 19,011 | 2,842,105 | 20,243 | 2.52 | 2.37 | 2.67 | 2.49 | 2.35 | 2.64 |
| mitapivat | 10 | 74,434 | 160 | 2,860,956 | 170 | 2.51 | 1.35 | 4.69 | 2.51 | 1.35 | 4.69 |
| sacubitril valsartan | 634 | 73,810 | 9,750 | 2,851,366 | 10,384 | 2.51 | 2.32 | 2.72 | 2.50 | 2.31 | 2.71 |
| nirogacestat | 12 | 74,432 | 191 | 2,860,925 | 203 | 2.51 | 1.42 | 4.45 | 2.51 | 1.42 | 4.45 |
| ciprofloxacin | 343 | 74,101 | 5,291 | 2,855,825 | 5,634 | 2.50 | 2.24 | 2.79 | 2.49 | 2.24 | 2.78 |
| nirmatrelvir ritonavir | 183 | 74,261 | 2,832 | 2,858,284 | 3,015 | 2.49 | 2.15 | 2.90 | 2.49 | 2.14 | 2.89 |
| selegiline | 9 | 74,435 | 146 | 2,860,970 | 155 | 2.49 | 1.29 | 4.80 | 2.49 | 1.29 | 4.80 |
| triamcinolone acetonide | 26 | 74,418 | 410 | 2,860,706 | 436 | 2.48 | 1.68 | 3.68 | 2.48 | 1.68 | 3.67 |
| umeclidinium bromide | 42 | 74,402 | 658 | 2,860,458 | 700 | 2.48 | 1.82 | 3.38 | 2.48 | 1.82 | 3.38 |
| modafinil | 11 | 74,433 | 178 | 2,860,938 | 189 | 2.48 | 1.36 | 4.50 | 2.48 | 1.36 | 4.49 |
| dextromethorphan guaifenesin | 48 | 74,396 | 759 | 2,860,357 | 807 | 2.46 | 1.84 | 3.28 | 2.45 | 1.84 | 3.28 |
| vamorolone | 5 | 74,439 | 86 | 2,861,030 | 91 | 2.44 | 1.03 | 5.79 | 2.44 | 1.03 | 5.79 |
| hydrochlorothiazide | 208 | 74,236 | 3,300 | 2,857,816 | 3,508 | 2.43 | 2.11 | 2.80 | 2.43 | 2.11 | 2.79 |
| bisoprolol | 39 | 74,405 | 629 | 2,860,487 | 668 | 2.41 | 1.75 | 3.33 | 2.41 | 1.75 | 3.33 |
| fluticasone furoate vilanterol trifenatate | 117 | 74,327 | 1,884 | 2,859,232 | 2,001 | 2.40 | 1.99 | 2.89 | 2.40 | 1.99 | 2.89 |
| selexipag | 365 | 74,079 | 5,886 | 2,855,230 | 6,251 | 2.39 | 2.15 | 2.66 | 2.39 | 2.15 | 2.65 |
| rituximab | 1,481 | 72,963 | 24,275 | 2,836,841 | 25,756 | 2.37 | 2.25 | 2.50 | 2.35 | 2.23 | 2.47 |
| ruxolitinib | 266 | 74,178 | 4,337 | 2,856,779 | 4,603 | 2.37 | 2.09 | 2.68 | 2.36 | 2.09 | 2.67 |
| atogepant | 44 | 74,400 | 726 | 2,860,390 | 770 | 2.35 | 1.74 | 3.19 | 2.35 | 1.74 | 3.19 |
| ascorbic acid | 21 | 74,423 | 351 | 2,860,765 | 372 | 2.35 | 1.52 | 3.63 | 2.35 | 1.52 | 3.63 |
| levodopa | 6 | 74,438 | 106 | 2,861,010 | 112 | 2.35 | 1.06 | 5.18 | 2.35 | 1.06 | 5.18 |
| galcanezumab gnlm | 84 | 74,360 | 1,386 | 2,859,730 | 1,470 | 2.34 | 1.88 | 2.92 | 2.34 | 1.88 | 2.92 |
| pacritinib | 27 | 74,417 | 451 | 2,860,665 | 478 | 2.34 | 1.59 | 3.44 | 2.34 | 1.59 | 3.44 |
| brotizolam | 5 | 74,439 | 90 | 2,861,026 | 95 | 2.34 | 0.99 | 5.52 | 2.34 | 0.99 | 5.52 |
| infliximab | 1,366 | 73,078 | 22,775 | 2,838,341 | 24,141 | 2.33 | 2.21 | 2.46 | 2.31 | 2.18 | 2.43 |
| secukinumab | 901 | 73,543 | 15,013 | 2,846,103 | 15,914 | 2.32 | 2.17 | 2.49 | 2.31 | 2.16 | 2.47 |
| oxycodone | 547 | 73,897 | 9,135 | 2,851,981 | 9,682 | 2.31 | 2.12 | 2.52 | 2.30 | 2.11 | 2.51 |
| amphetamine aspartate amphetamine aspartate amphetamine dextroamphetamine saccharate dextroamphetamine | 10 | 74,434 | 174 | 2,860,942 | 184 | 2.31 | 1.24 | 4.31 | 2.31 | 1.24 | 4.31 |
| tivozanib | 6 | 74,438 | 108 | 2,861,008 | 114 | 2.30 | 1.04 | 5.08 | 2.30 | 1.04 | 5.08 |
| enzalutamide | 276 | 74,168 | 4,638 | 2,856,478 | 4,914 | 2.30 | 2.03 | 2.59 | 2.29 | 2.03 | 2.59 |
| stiripentol | 19 | 74,425 | 326 | 2,860,790 | 345 | 2.30 | 1.45 | 3.63 | 2.30 | 1.45 | 3.62 |
| phentermine | 8 | 74,436 | 142 | 2,860,974 | 150 | 2.29 | 1.15 | 4.58 | 2.29 | 1.15 | 4.58 |
| tirzepatide | 122 | 74,322 | 2,062 | 2,859,054 | 2,184 | 2.28 | 1.90 | 2.74 | 2.28 | 1.90 | 2.74 |
| trospium chloride xanomeline | 6 | 74,438 | 109 | 2,861,007 | 115 | 2.28 | 1.03 | 5.03 | 2.28 | 1.03 | 5.03 |
| octreotide | 332 | 74,112 | 5,623 | 2,855,493 | 5,955 | 2.28 | 2.04 | 2.55 | 2.27 | 2.03 | 2.54 |
| oxybate | 34 | 74,410 | 583 | 2,860,533 | 617 | 2.27 | 1.61 | 3.20 | 2.27 | 1.61 | 3.20 |
| tesamorelin | 18 | 74,426 | 314 | 2,860,802 | 332 | 2.26 | 1.41 | 3.61 | 2.26 | 1.41 | 3.61 |
| atovaquone proguanil | 7 | 74,437 | 127 | 2,860,989 | 134 | 2.26 | 1.08 | 4.72 | 2.26 | 1.08 | 4.72 |
| brexpiprazole | 50 | 74,394 | 862 | 2,860,254 | 912 | 2.25 | 1.69 | 2.99 | 2.25 | 1.69 | 2.99 |
| umbralisib | 8 | 74,436 | 145 | 2,860,971 | 153 | 2.25 | 1.12 | 4.48 | 2.25 | 1.12 | 4.48 |
| colestipol | 5 | 74,439 | 94 | 2,861,022 | 99 | 2.24 | 0.95 | 5.28 | 2.24 | 0.95 | 5.28 |
| lisdexamfetamine | 5 | 74,439 | 95 | 2,861,021 | 100 | 2.21 | 0.94 | 5.23 | 2.21 | 0.94 | 5.23 |
| abacavir dolutegravir lamivudine | 37 | 74,407 | 653 | 2,860,463 | 690 | 2.21 | 1.59 | 3.07 | 2.21 | 1.59 | 3.06 |
| escitalopram | 248 | 74,196 | 4,340 | 2,856,776 | 4,588 | 2.20 | 1.94 | 2.51 | 2.20 | 1.94 | 2.50 |
| lenalidomide | 1,156 | 73,288 | 20,417 | 2,840,699 | 21,573 | 2.20 | 2.07 | 2.33 | 2.18 | 2.05 | 2.31 |
| fingolimod | 463 | 73,981 | 8,173 | 2,852,943 | 8,636 | 2.19 | 1.99 | 2.40 | 2.18 | 1.99 | 2.39 |
| patisiran | 36 | 74,408 | 642 | 2,860,474 | 678 | 2.18 | 1.56 | 3.05 | 2.18 | 1.56 | 3.05 |
| tamsulosin | 130 | 74,314 | 2,301 | 2,858,815 | 2,431 | 2.18 | 1.83 | 2.60 | 2.18 | 1.83 | 2.60 |
| maralixibat chloride | 9 | 74,435 | 167 | 2,860,949 | 176 | 2.18 | 1.13 | 4.19 | 2.18 | 1.13 | 4.19 |
| pyrimethamine | 16 | 74,428 | 291 | 2,860,825 | 307 | 2.18 | 1.32 | 3.57 | 2.18 | 1.32 | 3.57 |
| belimumab | 143 | 74,301 | 2,546 | 2,858,570 | 2,689 | 2.17 | 1.83 | 2.57 | 2.17 | 1.83 | 2.56 |
| bremelanotide | 6 | 74,438 | 115 | 2,861,001 | 121 | 2.16 | 0.98 | 4.77 | 2.16 | 0.98 | 4.77 |
| morphine | 345 | 74,099 | 6,176 | 2,854,940 | 6,521 | 2.16 | 1.93 | 2.40 | 2.15 | 1.93 | 2.40 |
| pexidartinib | 21 | 74,423 | 387 | 2,860,729 | 408 | 2.13 | 1.38 | 3.29 | 2.13 | 1.38 | 3.29 |
| fexofenadine | 41 | 74,403 | 749 | 2,860,367 | 790 | 2.13 | 1.56 | 2.91 | 2.13 | 1.56 | 2.91 |
| palbociclib | 467 | 73,977 | 8,508 | 2,852,608 | 8,975 | 2.12 | 1.93 | 2.33 | 2.11 | 1.92 | 2.32 |
| teprotumumab trbw | 38 | 74,406 | 703 | 2,860,413 | 741 | 2.10 | 1.52 | 2.91 | 2.10 | 1.52 | 2.91 |
| rizatriptan benzoate | 13 | 74,431 | 247 | 2,860,869 | 260 | 2.10 | 1.21 | 3.63 | 2.10 | 1.21 | 3.63 |
| phenprocoumon | 9 | 74,435 | 174 | 2,860,942 | 183 | 2.09 | 1.09 | 4.02 | 2.09 | 1.09 | 4.02 |
| ripretinib | 57 | 74,387 | 1,059 | 2,860,057 | 1,116 | 2.09 | 1.60 | 2.72 | 2.09 | 1.60 | 2.72 |
| deflazacort | 17 | 74,427 | 323 | 2,860,793 | 340 | 2.08 | 1.29 | 3.36 | 2.08 | 1.29 | 3.36 |
| aripiprazole | 291 | 74,153 | 5,399 | 2,855,717 | 5,690 | 2.08 | 1.85 | 2.34 | 2.07 | 1.84 | 2.33 |
| dexmethylphenidate | 10 | 74,434 | 194 | 2,860,922 | 204 | 2.07 | 1.12 | 3.86 | 2.07 | 1.12 | 3.86 |
| treprostinil | 413 | 74,031 | 7,694 | 2,853,422 | 8,107 | 2.07 | 1.88 | 2.29 | 2.07 | 1.87 | 2.28 |
| influenza virus vaccine | 5 | 74,439 | 102 | 2,861,014 | 107 | 2.06 | 0.87 | 4.86 | 2.06 | 0.87 | 4.86 |
| rabeprazole | 86 | 74,358 | 1,613 | 2,859,503 | 1,699 | 2.06 | 1.66 | 2.56 | 2.06 | 1.66 | 2.56 |
| erenumab aooe | 115 | 74,329 | 2,168 | 2,858,948 | 2,283 | 2.05 | 1.70 | 2.47 | 2.05 | 1.70 | 2.47 |
| vibegron | 21 | 74,423 | 403 | 2,860,713 | 424 | 2.05 | 1.33 | 3.16 | 2.05 | 1.33 | 3.16 |
| budesonide formoterol dihydrate | 286 | 74,158 | 5,393 | 2,855,723 | 5,679 | 2.05 | 1.82 | 2.30 | 2.04 | 1.81 | 2.30 |
| budesonide | 282 | 74,162 | 5,376 | 2,855,740 | 5,658 | 2.02 | 1.79 | 2.28 | 2.02 | 1.79 | 2.28 |
| dasabuvir | 12 | 74,432 | 238 | 2,860,878 | 250 | 2.01 | 1.14 | 3.56 | 2.01 | 1.14 | 3.56 |
| adalimumab aaty | 9 | 74,435 | 181 | 2,860,935 | 190 | 2.01 | 1.05 | 3.86 | 2.01 | 1.05 | 3.86 |
| nicotine | 62 | 74,382 | 1,198 | 2,859,918 | 1,260 | 2.01 | 1.55 | 2.59 | 2.00 | 1.55 | 2.58 |
| guaifenesin | 28 | 74,416 | 551 | 2,860,565 | 579 | 1.99 | 1.36 | 2.89 | 1.99 | 1.36 | 2.89 |
| duloxetine | 214 | 74,230 | 4,157 | 2,856,959 | 4,371 | 1.99 | 1.73 | 2.28 | 1.98 | 1.73 | 2.27 |
| dapagliflozin | 213 | 74,231 | 4,149 | 2,856,967 | 4,362 | 1.98 | 1.73 | 2.27 | 1.98 | 1.72 | 2.27 |
| tazemetostat | 19 | 74,425 | 379 | 2,860,737 | 398 | 1.98 | 1.25 | 3.11 | 1.97 | 1.25 | 3.11 |
| telotristat ethyl | 24 | 74,420 | 480 | 2,860,636 | 504 | 1.96 | 1.31 | 2.94 | 1.96 | 1.31 | 2.94 |
| mirabegron | 77 | 74,367 | 1,536 | 2,859,580 | 1,613 | 1.94 | 1.54 | 2.44 | 1.94 | 1.54 | 2.44 |
| lasmiditan | 5 | 74,439 | 109 | 2,861,007 | 114 | 1.93 | 0.82 | 4.55 | 1.93 | 0.82 | 4.55 |
| doxepin | 19 | 74,425 | 389 | 2,860,727 | 408 | 1.92 | 1.22 | 3.03 | 1.92 | 1.22 | 3.03 |
| voclosporin | 58 | 74,386 | 1,181 | 2,859,935 | 1,239 | 1.90 | 1.46 | 2.48 | 1.90 | 1.46 | 2.47 |
| lisdexamfetamine dimesylate | 70 | 74,374 | 1,429 | 2,859,687 | 1,499 | 1.90 | 1.49 | 2.41 | 1.90 | 1.49 | 2.41 |
| ergocalciferol | 13 | 74,431 | 274 | 2,860,842 | 287 | 1.89 | 1.09 | 3.26 | 1.89 | 1.09 | 3.26 |
| vortioxetine | 85 | 74,359 | 1,748 | 2,859,368 | 1,833 | 1.88 | 1.51 | 2.34 | 1.88 | 1.51 | 2.33 |
| doxylamine | 19 | 74,425 | 402 | 2,860,714 | 421 | 1.86 | 1.18 | 2.93 | 1.86 | 1.18 | 2.93 |
| diltiazem | 100 | 74,344 | 2,076 | 2,859,040 | 2,176 | 1.86 | 1.52 | 2.27 | 1.86 | 1.52 | 2.27 |
| oxycodone | 8 | 74,436 | 178 | 2,860,938 | 186 | 1.83 | 0.92 | 3.64 | 1.83 | 0.92 | 3.64 |
| paliperidone palmitate | 264 | 74,180 | 5,572 | 2,855,544 | 5,836 | 1.83 | 1.61 | 2.07 | 1.82 | 1.61 | 2.06 |
| sennosides | 7 | 74,437 | 158 | 2,860,958 | 165 | 1.82 | 0.87 | 3.78 | 1.82 | 0.87 | 3.78 |
| donepezil | 62 | 74,382 | 1,325 | 2,859,791 | 1,387 | 1.81 | 1.41 | 2.34 | 1.81 | 1.41 | 2.34 |
| nintedanib | 251 | 74,193 | 5,345 | 2,855,771 | 5,596 | 1.81 | 1.60 | 2.06 | 1.81 | 1.59 | 2.05 |
| citalopram | 129 | 74,315 | 2,760 | 2,858,356 | 2,889 | 1.80 | 1.51 | 2.15 | 1.80 | 1.51 | 2.15 |
| loratadine | 112 | 74,332 | 2,401 | 2,858,715 | 2,513 | 1.80 | 1.49 | 2.18 | 1.80 | 1.49 | 2.17 |
| macitentan | 422 | 74,022 | 9,056 | 2,852,060 | 9,478 | 1.80 | 1.63 | 1.98 | 1.79 | 1.63 | 1.98 |
| semaglutide | 341 | 74,103 | 7,358 | 2,853,758 | 7,699 | 1.79 | 1.60 | 1.99 | 1.78 | 1.60 | 1.99 |
| atomoxetine | 25 | 74,419 | 551 | 2,860,565 | 576 | 1.78 | 1.19 | 2.64 | 1.78 | 1.19 | 2.64 |
| inotersen | 34 | 74,410 | 746 | 2,860,370 | 780 | 1.78 | 1.26 | 2.50 | 1.78 | 1.26 | 2.50 |
| erenumab | 10 | 74,434 | 227 | 2,860,889 | 237 | 1.77 | 0.96 | 3.29 | 1.77 | 0.96 | 3.29 |
| sertraline | 298 | 74,146 | 6,479 | 2,854,637 | 6,777 | 1.77 | 1.58 | 1.99 | 1.77 | 1.58 | 1.99 |
| dimethyl | 234 | 74,210 | 5,116 | 2,856,000 | 5,350 | 1.76 | 1.55 | 2.01 | 1.76 | 1.55 | 2.01 |
| sparsentan | 30 | 74,414 | 665 | 2,860,451 | 695 | 1.76 | 1.23 | 2.53 | 1.76 | 1.23 | 2.53 |
| rosuvastatin | 300 | 74,144 | 6,595 | 2,854,521 | 6,895 | 1.75 | 1.56 | 1.97 | 1.75 | 1.56 | 1.97 |
| brexanolone | 6 | 74,438 | 142 | 2,860,974 | 148 | 1.75 | 0.80 | 3.85 | 1.75 | 0.80 | 3.85 |
| oseltamivir | 56 | 74,388 | 1,244 | 2,859,872 | 1,300 | 1.75 | 1.34 | 2.28 | 1.74 | 1.34 | 2.28 |
| ipratropium | 6 | 74,438 | 143 | 2,860,973 | 149 | 1.74 | 0.79 | 3.82 | 1.74 | 0.79 | 3.82 |
| anastrozole | 91 | 74,353 | 2,031 | 2,859,085 | 2,122 | 1.73 | 1.40 | 2.14 | 1.73 | 1.40 | 2.13 |
| mirtazapine | 184 | 74,260 | 4,134 | 2,856,982 | 4,318 | 1.72 | 1.48 | 1.99 | 1.72 | 1.48 | 1.99 |
| dimenhydrinate | 10 | 74,434 | 235 | 2,860,881 | 245 | 1.71 | 0.92 | 3.18 | 1.71 | 0.92 | 3.18 |
| metronidazole | 17 | 74,427 | 393 | 2,860,723 | 410 | 1.71 | 1.06 | 2.76 | 1.71 | 1.06 | 2.76 |
| rituximab abbs | 31 | 74,413 | 709 | 2,860,407 | 740 | 1.71 | 1.19 | 2.44 | 1.71 | 1.19 | 2.44 |
| obeticholic acid | 66 | 74,378 | 1,498 | 2,859,618 | 1,564 | 1.71 | 1.33 | 2.18 | 1.71 | 1.33 | 2.18 |
| eslicarbazepine | 37 | 74,407 | 846 | 2,860,270 | 883 | 1.70 | 1.23 | 2.36 | 1.70 | 1.23 | 2.36 |
| drospirenone | 16 | 74,428 | 372 | 2,860,744 | 388 | 1.70 | 1.04 | 2.79 | 1.70 | 1.04 | 2.79 |
| interferon beta 1a | 245 | 74,199 | 5,571 | 2,855,545 | 5,816 | 1.70 | 1.49 | 1.93 | 1.69 | 1.49 | 1.92 |
| clonazepam | 202 | 74,242 | 4,611 | 2,856,505 | 4,813 | 1.69 | 1.47 | 1.95 | 1.69 | 1.47 | 1.94 |
| lamotrigine | 216 | 74,228 | 4,966 | 2,856,150 | 5,182 | 1.68 | 1.46 | 1.92 | 1.68 | 1.46 | 1.92 |
| herbals mitragynine | 7 | 74,437 | 172 | 2,860,944 | 179 | 1.67 | 0.80 | 3.47 | 1.67 | 0.80 | 3.47 |
| doravirine lamivudine tenofovir disoproxil | 5 | 74,439 | 126 | 2,860,990 | 131 | 1.67 | 0.71 | 3.92 | 1.67 | 0.71 | 3.92 |
| amifampridine | 47 | 74,397 | 1,094 | 2,860,022 | 1,141 | 1.67 | 1.25 | 2.23 | 1.67 | 1.25 | 2.23 |
| bupropion | 152 | 74,292 | 3,534 | 2,857,582 | 3,686 | 1.66 | 1.41 | 1.95 | 1.66 | 1.41 | 1.95 |
| aclidinium bromide | 28 | 74,416 | 661 | 2,860,455 | 689 | 1.66 | 1.14 | 2.41 | 1.66 | 1.14 | 2.41 |
| ustekinumab | 768 | 73,676 | 17,911 | 2,843,205 | 18,679 | 1.66 | 1.54 | 1.78 | 1.65 | 1.53 | 1.77 |
| levofloxacin | 218 | 74,226 | 5,101 | 2,856,015 | 5,319 | 1.65 | 1.44 | 1.89 | 1.65 | 1.44 | 1.88 |
| adalimumab adaz | 81 | 74,363 | 1,913 | 2,859,203 | 1,994 | 1.64 | 1.31 | 2.04 | 1.64 | 1.31 | 2.04 |
| estradiol norethindrone | 30 | 74,414 | 720 | 2,860,396 | 750 | 1.63 | 1.13 | 2.34 | 1.63 | 1.13 | 2.34 |
| sofosbuvir velpatasvir voxilaprevir | 12 | 74,432 | 295 | 2,860,821 | 307 | 1.63 | 0.92 | 2.86 | 1.63 | 0.92 | 2.86 |
| brivaracetam | 47 | 74,397 | 1,123 | 2,859,993 | 1,170 | 1.63 | 1.22 | 2.17 | 1.62 | 1.22 | 2.17 |
| ethinyl estradiol norethindrone | 16 | 74,428 | 391 | 2,860,725 | 407 | 1.62 | 0.99 | 2.65 | 1.62 | 0.99 | 2.65 |
| glycopyrrolate | 37 | 74,407 | 890 | 2,860,226 | 927 | 1.62 | 1.17 | 2.24 | 1.62 | 1.17 | 2.24 |
| adalimumab afzb | 31 | 74,413 | 750 | 2,860,366 | 781 | 1.61 | 1.13 | 2.30 | 1.61 | 1.13 | 2.30 |
| ribociclib | 294 | 74,150 | 7,032 | 2,854,084 | 7,326 | 1.61 | 1.43 | 1.81 | 1.61 | 1.43 | 1.81 |
| fremanezumab vfrm | 41 | 74,403 | 990 | 2,860,126 | 1,031 | 1.61 | 1.18 | 2.20 | 1.61 | 1.18 | 2.20 |
| lorcaserin | 5 | 74,439 | 131 | 2,860,985 | 136 | 1.61 | 0.68 | 3.77 | 1.61 | 0.68 | 3.77 |
| taliglucerase alfa | 6 | 74,438 | 155 | 2,860,961 | 161 | 1.61 | 0.73 | 3.52 | 1.61 | 0.73 | 3.52 |
| ombitasvir paritaprevir ritonavir | 11 | 74,433 | 278 | 2,860,838 | 289 | 1.59 | 0.88 | 2.86 | 1.59 | 0.88 | 2.86 |
| leuprolide | 307 | 74,137 | 7,492 | 2,853,624 | 7,799 | 1.58 | 1.41 | 1.77 | 1.58 | 1.41 | 1.77 |
| minocycline | 21 | 74,423 | 525 | 2,860,591 | 546 | 1.57 | 1.02 | 2.42 | 1.57 | 1.02 | 2.42 |
| cyproterone | 7 | 74,437 | 183 | 2,860,933 | 190 | 1.57 | 0.76 | 3.26 | 1.57 | 0.76 | 3.26 |
| esketamine | 27 | 74,417 | 676 | 2,860,440 | 703 | 1.56 | 1.07 | 2.29 | 1.56 | 1.07 | 2.29 |
| rimegepant | 35 | 74,409 | 883 | 2,860,233 | 918 | 1.54 | 1.10 | 2.16 | 1.54 | 1.10 | 2.16 |
| venlafaxine | 207 | 74,237 | 5,183 | 2,855,933 | 5,390 | 1.54 | 1.34 | 1.77 | 1.54 | 1.34 | 1.77 |
| azelastine fluticasone propionate | 7 | 74,437 | 187 | 2,860,929 | 194 | 1.54 | 0.74 | 3.19 | 1.54 | 0.74 | 3.19 |
| cx 024414 | 5 | 74,439 | 137 | 2,860,979 | 142 | 1.54 | 0.66 | 3.61 | 1.54 | 0.66 | 3.60 |
| insulin degludec | 90 | 74,354 | 2,270 | 2,858,846 | 2,360 | 1.53 | 1.24 | 1.89 | 1.53 | 1.24 | 1.89 |
| gabapentin | 218 | 74,226 | 5,490 | 2,855,626 | 5,708 | 1.53 | 1.34 | 1.75 | 1.53 | 1.34 | 1.75 |
| carisoprodol | 6 | 74,438 | 163 | 2,860,953 | 169 | 1.53 | 0.70 | 3.35 | 1.53 | 0.70 | 3.35 |
| clarithromycin | 80 | 74,364 | 2,027 | 2,859,089 | 2,107 | 1.53 | 1.22 | 1.91 | 1.53 | 1.22 | 1.91 |
| perindopril erbumine | 5 | 74,439 | 138 | 2,860,978 | 143 | 1.53 | 0.65 | 3.58 | 1.53 | 0.65 | 3.58 |
| inclisiran | 53 | 74,391 | 1,356 | 2,859,760 | 1,409 | 1.52 | 1.15 | 1.99 | 1.52 | 1.15 | 1.99 |
| diroximel | 27 | 74,417 | 697 | 2,860,419 | 724 | 1.52 | 1.04 | 2.22 | 1.52 | 1.04 | 2.22 |
| paliperidone | 84 | 74,360 | 2,149 | 2,858,967 | 2,233 | 1.51 | 1.22 | 1.88 | 1.51 | 1.22 | 1.88 |
| ofloxacin | 19 | 74,425 | 496 | 2,860,620 | 515 | 1.51 | 0.96 | 2.37 | 1.51 | 0.96 | 2.37 |
| ribavirin | 43 | 74,401 | 1,108 | 2,860,008 | 1,151 | 1.51 | 1.11 | 2.04 | 1.51 | 1.11 | 2.04 |
| siponimod | 105 | 74,339 | 2,696 | 2,858,420 | 2,801 | 1.50 | 1.24 | 1.83 | 1.50 | 1.24 | 1.83 |
| fluticasone propionate salmeterol xinafoate | 91 | 74,353 | 2,340 | 2,858,776 | 2,431 | 1.50 | 1.22 | 1.85 | 1.50 | 1.22 | 1.85 |
| moxifloxacin | 85 | 74,359 | 2,196 | 2,858,920 | 2,281 | 1.50 | 1.21 | 1.86 | 1.50 | 1.21 | 1.86 |
| dolutegravir lamivudine | 13 | 74,431 | 347 | 2,860,769 | 360 | 1.49 | 0.87 | 2.57 | 1.49 | 0.87 | 2.57 |
| ozanimod | 89 | 74,355 | 2,305 | 2,858,811 | 2,394 | 1.49 | 1.21 | 1.84 | 1.49 | 1.21 | 1.84 |
| azathioprine | 75 | 74,369 | 1,953 | 2,859,163 | 2,028 | 1.49 | 1.18 | 1.87 | 1.49 | 1.18 | 1.87 |
| rosuvastatin | 6 | 74,438 | 168 | 2,860,948 | 174 | 1.48 | 0.68 | 3.25 | 1.48 | 0.68 | 3.25 |
| aminosalicylic acid | 9 | 74,435 | 247 | 2,860,869 | 256 | 1.48 | 0.77 | 2.82 | 1.48 | 0.77 | 2.82 |
| caplacizumab yhdp | 25 | 74,419 | 671 | 2,860,445 | 696 | 1.46 | 0.98 | 2.17 | 1.46 | 0.98 | 2.17 |
| tiotropium bromide | 71 | 74,373 | 1,888 | 2,859,228 | 1,959 | 1.46 | 1.15 | 1.84 | 1.46 | 1.15 | 1.84 |
| epoprostenol | 99 | 74,345 | 2,630 | 2,858,486 | 2,729 | 1.45 | 1.19 | 1.78 | 1.45 | 1.19 | 1.78 |
| beclomethasone dipropionate | 19 | 74,425 | 515 | 2,860,601 | 534 | 1.45 | 0.92 | 2.29 | 1.45 | 0.93 | 2.28 |
| candesartan cilexetil | 65 | 74,379 | 1,740 | 2,859,376 | 1,805 | 1.45 | 1.13 | 1.85 | 1.45 | 1.13 | 1.85 |
| progesterone | 28 | 74,416 | 757 | 2,860,359 | 785 | 1.45 | 0.99 | 2.10 | 1.45 | 0.99 | 2.10 |
| cimetidine | 8 | 74,436 | 226 | 2,860,890 | 234 | 1.44 | 0.73 | 2.86 | 1.44 | 0.73 | 2.86 |
| quetiapine | 384 | 74,060 | 10,273 | 2,850,843 | 10,657 | 1.44 | 1.30 | 1.60 | 1.44 | 1.30 | 1.59 |
| ublituximab | 9 | 74,435 | 253 | 2,860,863 | 262 | 1.44 | 0.75 | 2.75 | 1.44 | 0.75 | 2.75 |
| selinexor | 61 | 74,383 | 1,652 | 2,859,464 | 1,713 | 1.43 | 1.11 | 1.85 | 1.43 | 1.11 | 1.84 |
| fluticasone propionate | 58 | 74,386 | 1,574 | 2,859,542 | 1,632 | 1.43 | 1.10 | 1.85 | 1.43 | 1.10 | 1.85 |
| amphetamine dextroamphetamine | 6 | 74,438 | 175 | 2,860,941 | 181 | 1.42 | 0.65 | 3.11 | 1.42 | 0.65 | 3.11 |
| albuterol | 138 | 74,306 | 3,748 | 2,857,368 | 3,886 | 1.42 | 1.20 | 1.68 | 1.42 | 1.20 | 1.68 |
| emtricitabine rilpivirine tenofovir alafenamide | 12 | 74,432 | 338 | 2,860,778 | 350 | 1.42 | 0.81 | 2.50 | 1.42 | 0.81 | 2.50 |
| tamoxifen | 59 | 74,385 | 1,616 | 2,859,500 | 1,675 | 1.41 | 1.09 | 1.83 | 1.41 | 1.09 | 1.83 |
| risedronate | 29 | 74,415 | 801 | 2,860,315 | 830 | 1.41 | 0.98 | 2.04 | 1.41 | 0.98 | 2.04 |
| cobicistat elvitegravir emtricitabine tenofovir | 10 | 74,434 | 286 | 2,860,830 | 296 | 1.41 | 0.76 | 2.61 | 1.41 | 0.76 | 2.61 |
| ziprasidone | 13 | 74,431 | 370 | 2,860,746 | 383 | 1.40 | 0.81 | 2.41 | 1.40 | 0.81 | 2.41 |
| nevirapine | 9 | 74,435 | 261 | 2,860,855 | 270 | 1.40 | 0.73 | 2.67 | 1.40 | 0.73 | 2.67 |
| guanfacine | 23 | 74,421 | 647 | 2,860,469 | 670 | 1.39 | 0.92 | 2.11 | 1.39 | 0.92 | 2.10 |
| ambrisentan | 134 | 74,310 | 3,733 | 2,857,383 | 3,867 | 1.39 | 1.17 | 1.65 | 1.38 | 1.17 | 1.64 |
| maribavir | 12 | 74,432 | 347 | 2,860,769 | 359 | 1.38 | 0.79 | 2.43 | 1.38 | 0.79 | 2.43 |
| fluticasone furoate umeclidinium bromide vilanterol trifenatate | 58 | 74,386 | 1,636 | 2,859,480 | 1,694 | 1.37 | 1.06 | 1.78 | 1.37 | 1.06 | 1.78 |
| pomalidomide | 300 | 74,144 | 8,438 | 2,852,678 | 8,738 | 1.37 | 1.22 | 1.54 | 1.37 | 1.22 | 1.54 |
| prucalopride | 7 | 74,437 | 210 | 2,860,906 | 217 | 1.37 | 0.66 | 2.84 | 1.37 | 0.66 | 2.84 |
| ipratropium bromide | 29 | 74,415 | 832 | 2,860,284 | 861 | 1.36 | 0.94 | 1.97 | 1.36 | 0.94 | 1.97 |
| abacavir lamivudine | 13 | 74,431 | 381 | 2,860,735 | 394 | 1.36 | 0.79 | 2.34 | 1.36 | 0.79 | 2.34 |
| nebivolol | 41 | 74,403 | 1,180 | 2,859,936 | 1,221 | 1.35 | 0.99 | 1.84 | 1.35 | 0.99 | 1.84 |
| asfotase alfa | 17 | 74,427 | 498 | 2,860,618 | 515 | 1.35 | 0.84 | 2.17 | 1.35 | 0.84 | 2.17 |
| cholecalciferol | 58 | 74,386 | 1,668 | 2,859,448 | 1,726 | 1.35 | 1.04 | 1.75 | 1.35 | 1.04 | 1.75 |
| pegcetacoplan | 6 | 74,438 | 185 | 2,860,931 | 191 | 1.35 | 0.62 | 2.94 | 1.35 | 0.62 | 2.94 |
| idursulfase | 49 | 74,395 | 1,414 | 2,859,702 | 1,463 | 1.35 | 1.01 | 1.79 | 1.34 | 1.01 | 1.79 |
| aripiprazole lauroxil | 38 | 74,406 | 1,101 | 2,860,015 | 1,139 | 1.34 | 0.97 | 1.85 | 1.34 | 0.97 | 1.85 |
| peginterferon alfa 2a | 22 | 74,422 | 644 | 2,860,472 | 666 | 1.34 | 0.88 | 2.04 | 1.34 | 0.88 | 2.04 |
| adalimumab fkjp | 13 | 74,431 | 389 | 2,860,727 | 402 | 1.33 | 0.77 | 2.29 | 1.33 | 0.77 | 2.29 |
| emtricitabine | 7 | 74,437 | 216 | 2,860,900 | 223 | 1.33 | 0.64 | 2.76 | 1.33 | 0.64 | 2.76 |
| levonorgestrel | 19 | 74,425 | 564 | 2,860,552 | 583 | 1.33 | 0.85 | 2.09 | 1.33 | 0.85 | 2.09 |
| diphenhydramine | 88 | 74,356 | 2,566 | 2,858,550 | 2,654 | 1.33 | 1.07 | 1.64 | 1.33 | 1.07 | 1.64 |
| losartan | 115 | 74,329 | 3,355 | 2,857,761 | 3,470 | 1.32 | 1.10 | 1.59 | 1.32 | 1.10 | 1.59 |
| glecaprevir pibrentasvir | 99 | 74,345 | 2,904 | 2,858,212 | 3,003 | 1.32 | 1.08 | 1.61 | 1.32 | 1.08 | 1.61 |
| avacopan | 36 | 74,408 | 1,072 | 2,860,044 | 1,108 | 1.31 | 0.94 | 1.82 | 1.31 | 0.94 | 1.82 |
| tramadol | 200 | 74,244 | 5,914 | 2,855,202 | 6,114 | 1.30 | 1.13 | 1.50 | 1.30 | 1.13 | 1.50 |
| lorazepam | 148 | 74,296 | 4,389 | 2,856,727 | 4,537 | 1.30 | 1.10 | 1.53 | 1.30 | 1.10 | 1.53 |
| vigabatrin | 50 | 74,394 | 1,495 | 2,859,621 | 1,545 | 1.30 | 0.98 | 1.72 | 1.30 | 0.98 | 1.72 |
| rivastigmine | 45 | 74,399 | 1,351 | 2,859,765 | 1,396 | 1.29 | 0.96 | 1.74 | 1.29 | 0.96 | 1.74 |
| chlorpromazine | 19 | 74,425 | 579 | 2,860,537 | 598 | 1.29 | 0.82 | 2.03 | 1.29 | 0.82 | 2.03 |
| cyclosporine | 162 | 74,282 | 4,841 | 2,856,275 | 5,003 | 1.29 | 1.10 | 1.51 | 1.29 | 1.10 | 1.51 |
| dolutegravir rilpivirine | 7 | 74,437 | 223 | 2,860,893 | 230 | 1.29 | 0.62 | 2.67 | 1.29 | 0.62 | 2.67 |
| diamorphine | 12 | 74,432 | 373 | 2,860,743 | 385 | 1.29 | 0.73 | 2.26 | 1.29 | 0.73 | 2.26 |
| icatibant | 65 | 74,379 | 1,969 | 2,859,147 | 2,034 | 1.28 | 1.00 | 1.64 | 1.28 | 1.00 | 1.63 |
| nitrofurantoin | 6 | 74,438 | 195 | 2,860,921 | 201 | 1.28 | 0.58 | 2.79 | 1.28 | 0.58 | 2.79 |
| darolutamide | 18 | 74,426 | 557 | 2,860,559 | 575 | 1.28 | 0.80 | 2.03 | 1.28 | 0.80 | 2.03 |
| eptinezumab jjmr | 61 | 74,383 | 1,859 | 2,859,257 | 1,920 | 1.27 | 0.99 | 1.64 | 1.27 | 0.99 | 1.64 |
| levocetirizine | 5 | 74,439 | 168 | 2,860,948 | 173 | 1.25 | 0.54 | 2.93 | 1.25 | 0.54 | 2.93 |
| ixazomib | 190 | 74,254 | 5,848 | 2,855,268 | 6,038 | 1.25 | 1.08 | 1.45 | 1.25 | 1.08 | 1.45 |
| abemaciclib | 112 | 74,332 | 3,463 | 2,857,653 | 3,575 | 1.25 | 1.03 | 1.51 | 1.25 | 1.03 | 1.51 |
| meloxicam | 24 | 74,420 | 754 | 2,860,362 | 778 | 1.25 | 0.83 | 1.87 | 1.25 | 0.83 | 1.87 |
| bismuth subcitrate metronidazole tetracycline | 7 | 74,437 | 231 | 2,860,885 | 238 | 1.25 | 0.60 | 2.58 | 1.25 | 0.60 | 2.58 |
| exemestane | 50 | 74,394 | 1,562 | 2,859,554 | 1,612 | 1.24 | 0.94 | 1.64 | 1.24 | 0.94 | 1.64 |
| levalbuterol | 5 | 74,439 | 170 | 2,860,946 | 175 | 1.24 | 0.53 | 2.90 | 1.24 | 0.53 | 2.90 |
| dextroamphetamine | 9 | 74,435 | 295 | 2,860,821 | 304 | 1.24 | 0.65 | 2.36 | 1.24 | 0.65 | 2.36 |
| nitazoxanide | 5 | 74,439 | 171 | 2,860,945 | 176 | 1.23 | 0.53 | 2.88 | 1.23 | 0.53 | 2.88 |
| triamcinolone | 5 | 74,439 | 171 | 2,860,945 | 176 | 1.23 | 0.53 | 2.88 | 1.23 | 0.53 | 2.88 |
| alirocumab | 71 | 74,373 | 2,240 | 2,858,876 | 2,311 | 1.23 | 0.97 | 1.55 | 1.23 | 0.97 | 1.55 |
| rituximab pvvr | 40 | 74,404 | 1,274 | 2,859,842 | 1,314 | 1.22 | 0.89 | 1.67 | 1.22 | 0.89 | 1.67 |
| dextromethorphan | 21 | 74,423 | 678 | 2,860,438 | 699 | 1.22 | 0.79 | 1.87 | 1.22 | 0.79 | 1.87 |
| water | 16 | 74,428 | 521 | 2,860,595 | 537 | 1.22 | 0.74 | 1.99 | 1.22 | 0.74 | 1.98 |
| apomorphine | 35 | 74,409 | 1,123 | 2,859,993 | 1,158 | 1.21 | 0.87 | 1.70 | 1.21 | 0.87 | 1.70 |
| metformin vildagliptin | 32 | 74,412 | 1,031 | 2,860,085 | 1,063 | 1.21 | 0.85 | 1.72 | 1.21 | 0.85 | 1.72 |
| ubrogepant | 14 | 74,430 | 462 | 2,860,654 | 476 | 1.20 | 0.71 | 2.03 | 1.20 | 0.71 | 2.03 |
| alpha 1 proteinase inhibitor human | 52 | 74,392 | 1,676 | 2,859,440 | 1,728 | 1.20 | 0.91 | 1.58 | 1.20 | 0.91 | 1.58 |
| letrozole | 147 | 74,297 | 4,726 | 2,856,390 | 4,873 | 1.20 | 1.02 | 1.41 | 1.20 | 1.02 | 1.41 |
| acalabrutinib | 58 | 74,386 | 1,877 | 2,859,239 | 1,935 | 1.20 | 0.92 | 1.55 | 1.20 | 0.92 | 1.55 |
| trazodone | 63 | 74,381 | 2,038 | 2,859,078 | 2,101 | 1.20 | 0.93 | 1.54 | 1.20 | 0.93 | 1.54 |
| rifaximin | 66 | 74,378 | 2,141 | 2,858,975 | 2,207 | 1.19 | 0.94 | 1.52 | 1.19 | 0.94 | 1.52 |
| formoterol glycopyrrolate | 16 | 74,428 | 534 | 2,860,582 | 550 | 1.19 | 0.73 | 1.94 | 1.19 | 0.73 | 1.94 |
| bictegravir emtricitabine tenofovir alafenamide | 61 | 74,383 | 1,997 | 2,859,119 | 2,058 | 1.18 | 0.92 | 1.53 | 1.18 | 0.92 | 1.52 |
| divalproex | 45 | 74,399 | 1,478 | 2,859,638 | 1,523 | 1.18 | 0.88 | 1.59 | 1.18 | 0.88 | 1.59 |
| lanadelumab flyo | 19 | 74,425 | 634 | 2,860,482 | 653 | 1.18 | 0.75 | 1.85 | 1.18 | 0.75 | 1.85 |
| fentanyl | 149 | 74,295 | 4,874 | 2,856,242 | 5,023 | 1.18 | 1.00 | 1.39 | 1.18 | 1.00 | 1.39 |
| paroxetine | 83 | 74,361 | 2,722 | 2,858,394 | 2,805 | 1.18 | 0.95 | 1.47 | 1.18 | 0.95 | 1.47 |
| sucralfate | 7 | 74,437 | 245 | 2,860,871 | 252 | 1.17 | 0.57 | 2.43 | 1.17 | 0.57 | 2.43 |
| fruquintinib | 15 | 74,429 | 508 | 2,860,608 | 523 | 1.17 | 0.71 | 1.94 | 1.17 | 0.71 | 1.94 |
| eltrombopag olamine | 107 | 74,337 | 3,554 | 2,857,562 | 3,661 | 1.16 | 0.96 | 1.41 | 1.16 | 0.96 | 1.41 |
| vedolizumab | 443 | 74,001 | 14,685 | 2,846,431 | 15,128 | 1.16 | 1.06 | 1.28 | 1.16 | 1.06 | 1.28 |
| tolterodine | 7 | 74,437 | 249 | 2,860,867 | 256 | 1.16 | 0.56 | 2.39 | 1.16 | 0.56 | 2.39 |
| norethindrone | 6 | 74,438 | 216 | 2,860,900 | 222 | 1.15 | 0.53 | 2.52 | 1.15 | 0.53 | 2.52 |
| apalutamide | 103 | 74,341 | 3,456 | 2,857,660 | 3,559 | 1.15 | 0.95 | 1.40 | 1.15 | 0.95 | 1.40 |
| sarilumab | 92 | 74,352 | 3,091 | 2,858,025 | 3,183 | 1.15 | 0.94 | 1.41 | 1.15 | 0.94 | 1.41 |
| topiramate | 98 | 74,346 | 3,307 | 2,857,809 | 3,405 | 1.14 | 0.94 | 1.40 | 1.14 | 0.94 | 1.40 |
| amphetamine | 12 | 74,432 | 423 | 2,860,693 | 435 | 1.13 | 0.65 | 1.99 | 1.13 | 0.65 | 1.99 |
| levocetirizine dihydrochloride | 12 | 74,432 | 423 | 2,860,693 | 435 | 1.13 | 0.65 | 1.99 | 1.13 | 0.65 | 1.99 |
| risperidone | 238 | 74,206 | 8,085 | 2,853,031 | 8,323 | 1.13 | 1.00 | 1.29 | 1.13 | 1.00 | 1.29 |
| adalimumab ryvk | 5 | 74,439 | 186 | 2,860,930 | 191 | 1.13 | 0.49 | 2.65 | 1.13 | 0.49 | 2.65 |
| sipuleucel t | 5 | 74,439 | 186 | 2,860,930 | 191 | 1.13 | 0.49 | 2.65 | 1.13 | 0.49 | 2.65 |
| vandetanib | 14 | 74,430 | 493 | 2,860,623 | 507 | 1.13 | 0.67 | 1.90 | 1.13 | 0.67 | 1.90 |
| valsartan | 88 | 74,356 | 3,018 | 2,858,098 | 3,106 | 1.13 | 0.91 | 1.39 | 1.13 | 0.91 | 1.39 |
| amlodipine besylate hydrochlorothiazide valsartan | 17 | 74,427 | 598 | 2,860,518 | 615 | 1.12 | 0.70 | 1.81 | 1.12 | 0.70 | 1.81 |
| cefdinir | 7 | 74,437 | 256 | 2,860,860 | 263 | 1.12 | 0.54 | 2.32 | 1.12 | 0.54 | 2.32 |
| elbasvir grazoprevir | 9 | 74,435 | 326 | 2,860,790 | 335 | 1.12 | 0.59 | 2.13 | 1.12 | 0.59 | 2.13 |
| neratinib | 12 | 74,432 | 430 | 2,860,686 | 442 | 1.12 | 0.64 | 1.96 | 1.12 | 0.64 | 1.96 |
| buspirone | 19 | 74,425 | 673 | 2,860,443 | 692 | 1.11 | 0.71 | 1.75 | 1.11 | 0.71 | 1.75 |
| bosentan | 90 | 74,354 | 3,130 | 2,857,986 | 3,220 | 1.11 | 0.90 | 1.37 | 1.11 | 0.90 | 1.37 |
| buprenorphine naloxone | 12 | 74,432 | 433 | 2,860,683 | 445 | 1.11 | 0.63 | 1.94 | 1.11 | 0.63 | 1.94 |
| oxybutynin | 7 | 74,437 | 260 | 2,860,856 | 267 | 1.11 | 0.54 | 2.29 | 1.11 | 0.54 | 2.29 |
| isotretinoin | 37 | 74,407 | 1,303 | 2,859,813 | 1,340 | 1.11 | 0.80 | 1.53 | 1.11 | 0.80 | 1.53 |
| ponatinib | 65 | 74,379 | 2,285 | 2,858,831 | 2,350 | 1.10 | 0.86 | 1.41 | 1.10 | 0.86 | 1.41 |
| zanubrutinib | 12 | 74,432 | 436 | 2,860,680 | 448 | 1.10 | 0.63 | 1.93 | 1.10 | 0.63 | 1.93 |
| ticagrelor | 119 | 74,325 | 4,173 | 2,856,943 | 4,292 | 1.10 | 0.92 | 1.32 | 1.10 | 0.92 | 1.32 |
| anifrolumab fnia | 9 | 74,435 | 332 | 2,860,784 | 341 | 1.10 | 0.58 | 2.09 | 1.10 | 0.58 | 2.09 |
| lanreotide | 74 | 74,370 | 2,628 | 2,858,488 | 2,702 | 1.09 | 0.87 | 1.37 | 1.09 | 0.87 | 1.37 |
| mometasone furoate | 22 | 74,422 | 795 | 2,860,321 | 817 | 1.09 | 0.71 | 1.65 | 1.09 | 0.71 | 1.65 |
| naproxen | 96 | 74,348 | 3,418 | 2,857,698 | 3,514 | 1.09 | 0.89 | 1.33 | 1.08 | 0.89 | 1.33 |
| human c1 esterase inhibitor | 79 | 74,365 | 2,828 | 2,858,288 | 2,907 | 1.08 | 0.86 | 1.35 | 1.08 | 0.86 | 1.35 |
| bempedoic acid | 11 | 74,433 | 410 | 2,860,706 | 421 | 1.08 | 0.60 | 1.93 | 1.08 | 0.60 | 1.93 |
| indomethacin | 8 | 74,436 | 303 | 2,860,813 | 311 | 1.08 | 0.54 | 2.13 | 1.08 | 0.54 | 2.13 |
| ezetimibe | 63 | 74,381 | 2,274 | 2,858,842 | 2,337 | 1.07 | 0.84 | 1.38 | 1.07 | 0.84 | 1.38 |
| lisinopril | 123 | 74,321 | 4,428 | 2,856,688 | 4,551 | 1.07 | 0.90 | 1.28 | 1.07 | 0.90 | 1.28 |
| emtricitabine tenofovir alafenamide | 14 | 74,430 | 520 | 2,860,596 | 534 | 1.07 | 0.64 | 1.80 | 1.07 | 0.64 | 1.80 |
| vismodegib | 40 | 74,404 | 1,459 | 2,859,657 | 1,499 | 1.07 | 0.78 | 1.46 | 1.07 | 0.78 | 1.46 |
| testosterone undecanoate | 6 | 74,438 | 235 | 2,860,881 | 241 | 1.06 | 0.49 | 2.31 | 1.06 | 0.49 | 2.31 |
| dronabinol | 5 | 74,439 | 199 | 2,860,917 | 204 | 1.06 | 0.45 | 2.47 | 1.06 | 0.45 | 2.47 |
| letrozole ribociclib | 5 | 74,439 | 199 | 2,860,917 | 204 | 1.06 | 0.45 | 2.47 | 1.06 | 0.45 | 2.47 |
| domperidone | 11 | 74,433 | 418 | 2,860,698 | 429 | 1.06 | 0.59 | 1.90 | 1.06 | 0.59 | 1.90 |
| propranolol | 67 | 74,377 | 2,459 | 2,858,657 | 2,526 | 1.05 | 0.83 | 1.34 | 1.05 | 0.83 | 1.34 |
| pyridostigmine bromide | 5 | 74,439 | 200 | 2,860,916 | 205 | 1.05 | 0.45 | 2.46 | 1.05 | 0.45 | 2.46 |
| ravulizumab cwvz | 25 | 74,419 | 930 | 2,860,186 | 955 | 1.05 | 0.71 | 1.56 | 1.05 | 0.71 | 1.56 |
| polyethylene glycol 3350 | 65 | 74,379 | 2,391 | 2,858,725 | 2,456 | 1.05 | 0.82 | 1.35 | 1.05 | 0.82 | 1.35 |
| rotigotine | 48 | 74,396 | 1,777 | 2,859,339 | 1,825 | 1.05 | 0.79 | 1.39 | 1.05 | 0.79 | 1.39 |
| abiraterone | 127 | 74,317 | 4,676 | 2,856,440 | 4,803 | 1.05 | 0.88 | 1.25 | 1.05 | 0.88 | 1.25 |
| mavacamten | 24 | 74,420 | 900 | 2,860,216 | 924 | 1.05 | 0.70 | 1.56 | 1.05 | 0.70 | 1.56 |
| cabozantinib s | 164 | 74,280 | 6,058 | 2,855,058 | 6,222 | 1.04 | 0.89 | 1.22 | 1.04 | 0.89 | 1.22 |
| vonoprazan | 14 | 74,430 | 535 | 2,860,581 | 549 | 1.04 | 0.62 | 1.75 | 1.04 | 0.62 | 1.75 |
| methylprednisolone | 351 | 74,093 | 13,046 | 2,848,070 | 13,397 | 1.04 | 0.93 | 1.15 | 1.04 | 0.93 | 1.15 |
| cedazuridine decitabine | 11 | 74,433 | 427 | 2,860,689 | 438 | 1.03 | 0.58 | 1.86 | 1.03 | 0.58 | 1.86 |
| dolutegravir | 36 | 74,408 | 1,362 | 2,859,754 | 1,398 | 1.03 | 0.74 | 1.43 | 1.03 | 0.74 | 1.43 |
| minoxidil | 5 | 74,439 | 205 | 2,860,911 | 210 | 1.03 | 0.44 | 2.40 | 1.03 | 0.44 | 2.40 |
| naloxegol | 8 | 74,436 | 319 | 2,860,797 | 327 | 1.02 | 0.52 | 2.02 | 1.02 | 0.52 | 2.02 |
| elagolix estradiol norethindrone | 6 | 74,438 | 244 | 2,860,872 | 250 | 1.02 | 0.47 | 2.23 | 1.02 | 0.47 | 2.23 |
| onabotulinumtoxina | 55 | 74,389 | 2,090 | 2,859,026 | 2,145 | 1.02 | 0.78 | 1.33 | 1.02 | 0.78 | 1.33 |
| tapentadol | 19 | 74,425 | 734 | 2,860,382 | 753 | 1.02 | 0.65 | 1.60 | 1.02 | 0.65 | 1.60 |
| risankizumab rzaa | 223 | 74,221 | 8,436 | 2,852,680 | 8,659 | 1.02 | 0.89 | 1.16 | 1.02 | 0.89 | 1.16 |
| nortriptyline | 15 | 74,429 | 585 | 2,860,531 | 600 | 1.02 | 0.61 | 1.68 | 1.02 | 0.61 | 1.68 |
| asenapine | 6 | 74,438 | 246 | 2,860,870 | 252 | 1.01 | 0.47 | 2.21 | 1.01 | 0.47 | 2.21 |
| nilotinib | 104 | 74,340 | 3,980 | 2,857,136 | 4,084 | 1.01 | 0.83 | 1.23 | 1.01 | 0.83 | 1.23 |
| ibandronate | 20 | 74,424 | 784 | 2,860,332 | 804 | 1.00 | 0.65 | 1.56 | 1.00 | 0.65 | 1.56 |
| regorafenib | 54 | 74,390 | 2,088 | 2,859,028 | 2,142 | 1.00 | 0.77 | 1.31 | 1.00 | 0.77 | 1.31 |
| haloperidol decanoate | 13 | 74,431 | 517 | 2,860,599 | 530 | 1.00 | 0.58 | 1.72 | 1.00 | 0.58 | 1.72 |
| cholestyramine | 10 | 74,434 | 402 | 2,860,714 | 412 | 1.00 | 0.54 | 1.85 | 1.00 | 0.54 | 1.85 |
| triazolam | 7 | 74,437 | 287 | 2,860,829 | 294 | 1.00 | 0.49 | 2.07 | 1.00 | 0.49 | 2.07 |
| tizanidine | 18 | 74,426 | 711 | 2,860,405 | 729 | 1.00 | 0.63 | 1.59 | 1.00 | 0.63 | 1.59 |
| tramadol | 17 | 74,427 | 678 | 2,860,438 | 695 | 0.99 | 0.62 | 1.59 | 0.99 | 0.62 | 1.59 |
| estradiol levonorgestrel | 7 | 74,437 | 292 | 2,860,824 | 299 | 0.99 | 0.48 | 2.03 | 0.99 | 0.48 | 2.03 |
| acyclovir | 62 | 74,382 | 2,437 | 2,858,679 | 2,499 | 0.99 | 0.77 | 1.27 | 0.99 | 0.77 | 1.27 |
| lubiprostone | 6 | 74,438 | 254 | 2,860,862 | 260 | 0.98 | 0.45 | 2.14 | 0.98 | 0.45 | 2.14 |
| ibrutinib | 308 | 74,136 | 12,204 | 2,848,912 | 12,512 | 0.97 | 0.87 | 1.09 | 0.97 | 0.87 | 1.09 |
| azelastine | 12 | 74,432 | 495 | 2,860,621 | 507 | 0.97 | 0.55 | 1.70 | 0.97 | 0.55 | 1.70 |
| emtricitabine tenofovir | 16 | 74,428 | 654 | 2,860,462 | 670 | 0.97 | 0.59 | 1.58 | 0.97 | 0.59 | 1.58 |
| natalizumab | 80 | 74,364 | 3,196 | 2,857,920 | 3,276 | 0.97 | 0.78 | 1.21 | 0.97 | 0.78 | 1.21 |
| cladribine | 54 | 74,390 | 2,171 | 2,858,945 | 2,225 | 0.96 | 0.74 | 1.26 | 0.96 | 0.74 | 1.26 |
| rilonacept | 6 | 74,438 | 259 | 2,860,857 | 265 | 0.96 | 0.44 | 2.10 | 0.96 | 0.44 | 2.10 |
| migalastat | 8 | 74,436 | 339 | 2,860,777 | 347 | 0.96 | 0.49 | 1.90 | 0.96 | 0.49 | 1.90 |
| budesonide formoterol glycopyrronium | 26 | 74,418 | 1,062 | 2,860,054 | 1,088 | 0.96 | 0.65 | 1.41 | 0.96 | 0.65 | 1.41 |
| cabotegravir rilpivirine | 11 | 74,433 | 461 | 2,860,655 | 472 | 0.96 | 0.53 | 1.72 | 0.96 | 0.53 | 1.72 |
| fluvoxamine | 9 | 74,435 | 381 | 2,860,735 | 390 | 0.96 | 0.50 | 1.82 | 0.96 | 0.50 | 1.82 |
| ropinirole | 8 | 74,436 | 343 | 2,860,773 | 351 | 0.95 | 0.48 | 1.88 | 0.95 | 0.48 | 1.88 |
| pitavastatin | 7 | 74,437 | 303 | 2,860,813 | 310 | 0.95 | 0.46 | 1.96 | 0.95 | 0.46 | 1.96 |
| budesonide formoterol glycopyrrolate | 22 | 74,422 | 910 | 2,860,206 | 932 | 0.95 | 0.63 | 1.44 | 0.95 | 0.63 | 1.44 |
| valacyclovir | 70 | 74,374 | 2,861 | 2,858,255 | 2,931 | 0.95 | 0.75 | 1.20 | 0.95 | 0.75 | 1.20 |
| pegvaliase pqpz | 25 | 74,419 | 1,035 | 2,860,081 | 1,060 | 0.95 | 0.64 | 1.40 | 0.95 | 0.64 | 1.40 |
| lecanemab irmb | 15 | 74,429 | 629 | 2,860,487 | 644 | 0.95 | 0.57 | 1.57 | 0.95 | 0.57 | 1.57 |
| ramipril | 133 | 74,311 | 5,433 | 2,855,683 | 5,566 | 0.94 | 0.80 | 1.12 | 0.94 | 0.80 | 1.12 |
| ivosidenib | 7 | 74,437 | 306 | 2,860,810 | 313 | 0.94 | 0.46 | 1.94 | 0.94 | 0.46 | 1.94 |
| benralizumab | 96 | 74,348 | 3,944 | 2,857,172 | 4,040 | 0.94 | 0.77 | 1.15 | 0.94 | 0.77 | 1.15 |
| propafenone | 5 | 74,439 | 225 | 2,860,891 | 230 | 0.94 | 0.40 | 2.18 | 0.94 | 0.40 | 2.18 |
| hydrochlorothiazide lisinopril | 12 | 74,432 | 516 | 2,860,600 | 528 | 0.93 | 0.53 | 1.63 | 0.93 | 0.53 | 1.63 |
| voriconazole | 66 | 74,378 | 2,762 | 2,858,354 | 2,828 | 0.93 | 0.73 | 1.18 | 0.93 | 0.73 | 1.18 |
| evolocumab | 82 | 74,362 | 3,434 | 2,857,682 | 3,516 | 0.92 | 0.74 | 1.15 | 0.92 | 0.74 | 1.15 |
| bosutinib | 45 | 74,399 | 1,906 | 2,859,210 | 1,951 | 0.92 | 0.68 | 1.23 | 0.92 | 0.68 | 1.23 |
| dulaglutide | 88 | 74,356 | 3,720 | 2,857,396 | 3,808 | 0.91 | 0.74 | 1.13 | 0.91 | 0.74 | 1.13 |
| gadoteridol | 6 | 74,438 | 273 | 2,860,843 | 279 | 0.91 | 0.42 | 1.99 | 0.91 | 0.42 | 1.99 |
| amisulpride | 8 | 74,436 | 358 | 2,860,758 | 366 | 0.91 | 0.46 | 1.80 | 0.91 | 0.46 | 1.80 |
| upadacitinib | 270 | 74,174 | 11,476 | 2,849,640 | 11,746 | 0.91 | 0.80 | 1.02 | 0.91 | 0.80 | 1.02 |
| human immunoglobulin g hyaluronidase human recombinant | 31 | 74,413 | 1,339 | 2,859,777 | 1,370 | 0.90 | 0.63 | 1.29 | 0.90 | 0.63 | 1.29 |
| estradiol norethindrone relugolix | 7 | 74,437 | 320 | 2,860,796 | 327 | 0.90 | 0.44 | 1.86 | 0.90 | 0.44 | 1.86 |
| bamlanivimab | 21 | 74,423 | 919 | 2,860,197 | 940 | 0.90 | 0.59 | 1.38 | 0.90 | 0.59 | 1.38 |
| desmopressin | 14 | 74,430 | 620 | 2,860,496 | 634 | 0.90 | 0.53 | 1.51 | 0.90 | 0.53 | 1.51 |
| tolvaptan | 59 | 74,385 | 2,550 | 2,858,566 | 2,609 | 0.90 | 0.69 | 1.16 | 0.90 | 0.69 | 1.16 |
| acetaminophen codeine | 28 | 74,416 | 1,226 | 2,859,890 | 1,254 | 0.89 | 0.62 | 1.29 | 0.89 | 0.62 | 1.29 |
| teduglutide | 88 | 74,356 | 3,821 | 2,857,295 | 3,909 | 0.89 | 0.72 | 1.10 | 0.89 | 0.72 | 1.10 |
| lacosamide | 73 | 74,371 | 3,177 | 2,857,939 | 3,250 | 0.89 | 0.71 | 1.12 | 0.89 | 0.71 | 1.12 |
| satralizumab mwge | 10 | 74,434 | 455 | 2,860,661 | 465 | 0.89 | 0.48 | 1.63 | 0.89 | 0.48 | 1.63 |
| botulinum toxin type a | 13 | 74,431 | 587 | 2,860,529 | 600 | 0.88 | 0.51 | 1.51 | 0.88 | 0.51 | 1.51 |
| amlodipine besylate | 196 | 74,248 | 8,549 | 2,852,567 | 8,745 | 0.88 | 0.77 | 1.02 | 0.88 | 0.77 | 1.02 |
| herbals | 9 | 74,435 | 413 | 2,860,703 | 422 | 0.88 | 0.46 | 1.68 | 0.88 | 0.46 | 1.68 |
| rilpivirine | 28 | 74,416 | 1,249 | 2,859,867 | 1,277 | 0.88 | 0.60 | 1.27 | 0.88 | 0.60 | 1.27 |
| miglustat | 5 | 74,439 | 241 | 2,860,875 | 246 | 0.88 | 0.38 | 2.04 | 0.88 | 0.38 | 2.04 |
| clofazimine | 32 | 74,412 | 1,429 | 2,859,687 | 1,461 | 0.87 | 0.62 | 1.24 | 0.87 | 0.62 | 1.24 |
| fluoxetine | 73 | 74,371 | 3,245 | 2,857,871 | 3,318 | 0.87 | 0.69 | 1.10 | 0.87 | 0.69 | 1.10 |
| efavirenz | 12 | 74,432 | 552 | 2,860,564 | 564 | 0.87 | 0.50 | 1.52 | 0.87 | 0.50 | 1.52 |
| voxelotor | 63 | 74,381 | 2,810 | 2,858,306 | 2,873 | 0.87 | 0.68 | 1.11 | 0.87 | 0.68 | 1.11 |
| famotidine | 23 | 74,421 | 1,040 | 2,860,076 | 1,063 | 0.87 | 0.58 | 1.31 | 0.87 | 0.58 | 1.31 |
| bimekizumab | 10 | 74,434 | 467 | 2,860,649 | 477 | 0.86 | 0.47 | 1.59 | 0.86 | 0.47 | 1.59 |
| fluticasone furoate | 13 | 74,431 | 601 | 2,860,515 | 614 | 0.86 | 0.50 | 1.48 | 0.86 | 0.50 | 1.48 |
| omadacycline | 12 | 74,432 | 559 | 2,860,557 | 571 | 0.86 | 0.49 | 1.50 | 0.86 | 0.49 | 1.50 |
| alprazolam | 128 | 74,316 | 5,769 | 2,855,347 | 5,897 | 0.86 | 0.72 | 1.02 | 0.86 | 0.72 | 1.02 |
| fluticasone | 10 | 74,434 | 473 | 2,860,643 | 483 | 0.85 | 0.46 | 1.57 | 0.85 | 0.46 | 1.57 |
| fentanyl | 9 | 74,435 | 429 | 2,860,687 | 438 | 0.85 | 0.45 | 1.62 | 0.85 | 0.45 | 1.62 |
| eculizumab | 59 | 74,385 | 2,690 | 2,858,426 | 2,749 | 0.85 | 0.66 | 1.10 | 0.85 | 0.66 | 1.10 |
| clobazam | 26 | 74,418 | 1,198 | 2,859,918 | 1,224 | 0.85 | 0.58 | 1.25 | 0.85 | 0.58 | 1.25 |
| amikacin | 61 | 74,383 | 2,798 | 2,858,318 | 2,859 | 0.84 | 0.66 | 1.09 | 0.84 | 0.66 | 1.09 |
| tafamidis | 31 | 74,413 | 1,435 | 2,859,681 | 1,466 | 0.84 | 0.59 | 1.20 | 0.84 | 0.59 | 1.20 |
| etonogestrel | 8 | 74,436 | 388 | 2,860,728 | 396 | 0.84 | 0.43 | 1.66 | 0.84 | 0.43 | 1.66 |
| ivacaftor | 41 | 74,403 | 1,899 | 2,859,217 | 1,940 | 0.84 | 0.62 | 1.14 | 0.84 | 0.62 | 1.14 |
| bebtelovimab | 6 | 74,438 | 298 | 2,860,818 | 304 | 0.84 | 0.38 | 1.82 | 0.84 | 0.38 | 1.82 |
| anakinra | 54 | 74,390 | 2,514 | 2,858,602 | 2,568 | 0.83 | 0.64 | 1.09 | 0.83 | 0.64 | 1.09 |
| alpelisib | 45 | 74,399 | 2,099 | 2,859,017 | 2,144 | 0.83 | 0.62 | 1.12 | 0.83 | 0.62 | 1.12 |
| lorlatinib | 38 | 74,406 | 1,780 | 2,859,336 | 1,818 | 0.83 | 0.60 | 1.14 | 0.83 | 0.60 | 1.14 |
| parathyroid hormone | 17 | 74,427 | 814 | 2,860,302 | 831 | 0.83 | 0.51 | 1.33 | 0.83 | 0.51 | 1.33 |
| pantoprazole | 207 | 74,237 | 9,680 | 2,851,436 | 9,887 | 0.82 | 0.72 | 0.94 | 0.82 | 0.72 | 0.95 |
| prednisolone | 181 | 74,263 | 8,476 | 2,852,640 | 8,657 | 0.82 | 0.71 | 0.95 | 0.82 | 0.71 | 0.95 |
| sunitinib | 61 | 74,383 | 2,876 | 2,858,240 | 2,937 | 0.82 | 0.64 | 1.06 | 0.82 | 0.64 | 1.06 |
| carbidopa entacapone levodopa | 6 | 74,438 | 304 | 2,860,812 | 310 | 0.82 | 0.38 | 1.78 | 0.82 | 0.38 | 1.78 |
| apixaban | 249 | 74,195 | 11,886 | 2,849,230 | 12,135 | 0.81 | 0.71 | 0.91 | 0.81 | 0.71 | 0.91 |
| vildagliptin | 9 | 74,435 | 454 | 2,860,662 | 463 | 0.80 | 0.42 | 1.53 | 0.80 | 0.42 | 1.53 |
| pegloticase | 11 | 74,433 | 551 | 2,860,565 | 562 | 0.80 | 0.45 | 1.44 | 0.80 | 0.45 | 1.44 |
| formoterol dihydrate mometasone furoate | 5 | 74,439 | 264 | 2,860,852 | 269 | 0.80 | 0.34 | 1.86 | 0.80 | 0.34 | 1.86 |
| medroxyprogesterone | 16 | 74,428 | 796 | 2,860,320 | 812 | 0.80 | 0.49 | 1.30 | 0.80 | 0.49 | 1.30 |
| pancrelipase amylase pancrelipase lipase pancrelipase protease | 60 | 74,384 | 2,927 | 2,858,189 | 2,987 | 0.79 | 0.62 | 1.02 | 0.79 | 0.62 | 1.02 |
| somatropin | 137 | 74,307 | 6,652 | 2,854,464 | 6,789 | 0.79 | 0.67 | 0.94 | 0.79 | 0.67 | 0.94 |
| acetaminophen | 274 | 74,170 | 13,294 | 2,847,822 | 13,568 | 0.79 | 0.70 | 0.89 | 0.79 | 0.70 | 0.89 |
| imipenem | 6 | 74,438 | 315 | 2,860,801 | 321 | 0.79 | 0.36 | 1.72 | 0.79 | 0.36 | 1.72 |
| teriparatide | 62 | 74,382 | 3,039 | 2,858,077 | 3,101 | 0.79 | 0.61 | 1.02 | 0.79 | 0.62 | 1.02 |
| rasagiline | 11 | 74,433 | 561 | 2,860,555 | 572 | 0.79 | 0.44 | 1.41 | 0.79 | 0.44 | 1.41 |
| human immunoglobulin g | 294 | 74,150 | 14,377 | 2,846,739 | 14,671 | 0.79 | 0.70 | 0.88 | 0.79 | 0.70 | 0.88 |
| umeclidinium bromide vilanterol trifenatate | 20 | 74,424 | 1,006 | 2,860,110 | 1,026 | 0.78 | 0.51 | 1.21 | 0.78 | 0.51 | 1.21 |
| atenolol | 38 | 74,406 | 1,890 | 2,859,226 | 1,928 | 0.78 | 0.57 | 1.08 | 0.78 | 0.57 | 1.08 |
| dutasteride | 11 | 74,433 | 565 | 2,860,551 | 576 | 0.78 | 0.44 | 1.40 | 0.78 | 0.44 | 1.40 |
| zonisamide | 10 | 74,434 | 516 | 2,860,600 | 526 | 0.78 | 0.42 | 1.44 | 0.78 | 0.42 | 1.44 |
| hydrochlorothiazide losartan | 13 | 74,431 | 669 | 2,860,447 | 682 | 0.77 | 0.45 | 1.33 | 0.77 | 0.45 | 1.33 |
| brigatinib | 25 | 74,419 | 1,266 | 2,859,850 | 1,291 | 0.77 | 0.52 | 1.15 | 0.77 | 0.52 | 1.15 |
| metoprolol | 101 | 74,343 | 5,044 | 2,856,072 | 5,145 | 0.77 | 0.64 | 0.94 | 0.77 | 0.64 | 0.94 |
| arformoterol | 5 | 74,439 | 273 | 2,860,843 | 278 | 0.77 | 0.33 | 1.80 | 0.77 | 0.33 | 1.80 |
| perindopril | 25 | 74,419 | 1,275 | 2,859,841 | 1,300 | 0.77 | 0.52 | 1.14 | 0.77 | 0.52 | 1.14 |
| laronidase | 20 | 74,424 | 1,025 | 2,860,091 | 1,045 | 0.77 | 0.50 | 1.19 | 0.77 | 0.50 | 1.19 |
| dapagliflozin propanediol | 50 | 74,394 | 2,537 | 2,858,579 | 2,587 | 0.76 | 0.58 | 1.01 | 0.76 | 0.58 | 1.01 |
| icosapent ethyl | 16 | 74,428 | 832 | 2,860,284 | 848 | 0.76 | 0.47 | 1.24 | 0.76 | 0.47 | 1.24 |
| droxidopa | 39 | 74,405 | 1,999 | 2,859,117 | 2,038 | 0.76 | 0.55 | 1.04 | 0.76 | 0.55 | 1.04 |
| fostamatinib | 27 | 74,417 | 1,404 | 2,859,712 | 1,431 | 0.75 | 0.52 | 1.10 | 0.75 | 0.52 | 1.10 |
| glycopyrronium indacaterol | 5 | 74,439 | 281 | 2,860,835 | 286 | 0.75 | 0.32 | 1.75 | 0.75 | 0.32 | 1.75 |
| levetiracetam | 120 | 74,324 | 6,168 | 2,854,948 | 6,288 | 0.75 | 0.63 | 0.90 | 0.75 | 0.63 | 0.90 |
| lenvatinib | 208 | 74,236 | 10,701 | 2,850,415 | 10,909 | 0.75 | 0.65 | 0.86 | 0.75 | 0.65 | 0.86 |
| cabotegravir | 23 | 74,421 | 1,209 | 2,859,907 | 1,232 | 0.75 | 0.50 | 1.12 | 0.75 | 0.50 | 1.12 |
| glatiramer | 77 | 74,367 | 3,989 | 2,857,127 | 4,066 | 0.75 | 0.60 | 0.93 | 0.75 | 0.60 | 0.93 |
| carvedilol | 32 | 74,412 | 1,674 | 2,859,442 | 1,706 | 0.75 | 0.53 | 1.06 | 0.75 | 0.53 | 1.06 |
| riociguat | 59 | 74,385 | 3,075 | 2,858,041 | 3,134 | 0.74 | 0.58 | 0.96 | 0.74 | 0.58 | 0.96 |
| benserazide levodopa | 8 | 74,436 | 440 | 2,860,676 | 448 | 0.74 | 0.38 | 1.46 | 0.74 | 0.38 | 1.46 |
| insulin aspart | 94 | 74,350 | 4,915 | 2,856,201 | 5,009 | 0.74 | 0.60 | 0.91 | 0.74 | 0.60 | 0.91 |
| fesoterodine | 12 | 74,432 | 653 | 2,860,463 | 665 | 0.74 | 0.42 | 1.29 | 0.74 | 0.42 | 1.29 |
| betamethasone | 9 | 74,435 | 497 | 2,860,619 | 506 | 0.73 | 0.39 | 1.39 | 0.73 | 0.39 | 1.39 |
| triptorelin | 13 | 74,431 | 712 | 2,860,404 | 725 | 0.73 | 0.42 | 1.25 | 0.73 | 0.43 | 1.25 |
| canakinumab | 46 | 74,398 | 2,454 | 2,858,662 | 2,500 | 0.73 | 0.54 | 0.97 | 0.73 | 0.54 | 0.97 |
| procarbazine | 9 | 74,435 | 502 | 2,860,614 | 511 | 0.73 | 0.38 | 1.38 | 0.73 | 0.38 | 1.38 |
| cannabis sativa subsp indica top | 11 | 74,433 | 608 | 2,860,508 | 619 | 0.73 | 0.41 | 1.30 | 0.73 | 0.41 | 1.30 |
| atazanavir | 8 | 74,436 | 450 | 2,860,666 | 458 | 0.73 | 0.37 | 1.43 | 0.73 | 0.37 | 1.43 |
| linaclotide | 23 | 74,421 | 1,251 | 2,859,865 | 1,274 | 0.72 | 0.48 | 1.09 | 0.72 | 0.48 | 1.09 |
| epirubicin | 51 | 74,393 | 2,744 | 2,858,372 | 2,795 | 0.72 | 0.55 | 0.95 | 0.72 | 0.55 | 0.95 |
| avatrombopag | 11 | 74,433 | 613 | 2,860,503 | 624 | 0.72 | 0.40 | 1.29 | 0.72 | 0.40 | 1.29 |
| pralsetinib | 13 | 74,431 | 721 | 2,860,395 | 734 | 0.72 | 0.42 | 1.23 | 0.72 | 0.42 | 1.23 |
| cobicistat elvitegravir emtricitabine tenofovir alafenamide | 8 | 74,436 | 456 | 2,860,660 | 464 | 0.72 | 0.36 | 1.41 | 0.72 | 0.36 | 1.41 |
| mitotane | 7 | 74,437 | 403 | 2,860,713 | 410 | 0.71 | 0.35 | 1.47 | 0.71 | 0.35 | 1.47 |
| paricalcitol | 13 | 74,431 | 726 | 2,860,390 | 739 | 0.71 | 0.42 | 1.22 | 0.71 | 0.42 | 1.22 |
| mobocertinib | 5 | 74,439 | 296 | 2,860,820 | 301 | 0.71 | 0.31 | 1.66 | 0.71 | 0.31 | 1.66 |
| sildenafil | 75 | 74,369 | 4,080 | 2,857,036 | 4,155 | 0.71 | 0.57 | 0.89 | 0.71 | 0.57 | 0.89 |
| telmisartan | 17 | 74,427 | 949 | 2,860,167 | 966 | 0.71 | 0.44 | 1.14 | 0.71 | 0.44 | 1.14 |
| ezetimibe simvastatin | 7 | 74,437 | 408 | 2,860,708 | 415 | 0.71 | 0.34 | 1.45 | 0.71 | 0.34 | 1.45 |
| abrocitinib | 16 | 74,428 | 899 | 2,860,217 | 915 | 0.70 | 0.43 | 1.15 | 0.70 | 0.43 | 1.15 |
| ibuprofen | 149 | 74,295 | 8,193 | 2,852,923 | 8,342 | 0.70 | 0.60 | 0.82 | 0.70 | 0.60 | 0.82 |
| cyclobenzaprine | 15 | 74,429 | 851 | 2,860,265 | 866 | 0.70 | 0.42 | 1.16 | 0.70 | 0.42 | 1.16 |
| bicalutamide | 13 | 74,431 | 744 | 2,860,372 | 757 | 0.70 | 0.41 | 1.19 | 0.70 | 0.41 | 1.19 |
| candesartan cilexetil hydrochlorothiazide | 8 | 74,436 | 471 | 2,860,645 | 479 | 0.69 | 0.35 | 1.37 | 0.69 | 0.35 | 1.37 |
| infliximab abda | 14 | 74,430 | 806 | 2,860,310 | 820 | 0.69 | 0.41 | 1.16 | 0.69 | 0.41 | 1.16 |
| hydrochlorothiazide valsartan | 20 | 74,424 | 1,142 | 2,859,974 | 1,162 | 0.69 | 0.45 | 1.07 | 0.69 | 0.45 | 1.07 |
| methadone | 25 | 74,419 | 1,421 | 2,859,695 | 1,446 | 0.69 | 0.47 | 1.02 | 0.69 | 0.47 | 1.02 |
| bisoprolol | 86 | 74,358 | 4,875 | 2,856,241 | 4,961 | 0.68 | 0.55 | 0.84 | 0.68 | 0.55 | 0.84 |
| acetaminophen tramadol | 18 | 74,426 | 1,048 | 2,860,068 | 1,066 | 0.68 | 0.43 | 1.07 | 0.68 | 0.43 | 1.07 |
| lansoprazole | 110 | 74,334 | 6,268 | 2,854,848 | 6,378 | 0.68 | 0.56 | 0.82 | 0.68 | 0.56 | 0.82 |
| perampanel | 21 | 74,423 | 1,220 | 2,859,896 | 1,241 | 0.68 | 0.44 | 1.04 | 0.68 | 0.44 | 1.04 |
| belumosudil | 13 | 74,431 | 770 | 2,860,346 | 783 | 0.67 | 0.39 | 1.15 | 0.67 | 0.39 | 1.15 |
| cobicistat darunavir emtricitabine tenofovir alafenamide | 9 | 74,435 | 542 | 2,860,574 | 551 | 0.67 | 0.35 | 1.28 | 0.67 | 0.35 | 1.28 |
| tucatinib | 15 | 74,429 | 886 | 2,860,230 | 901 | 0.67 | 0.41 | 1.11 | 0.67 | 0.41 | 1.11 |
| tozinameran | 26 | 74,418 | 1,520 | 2,859,596 | 1,546 | 0.67 | 0.46 | 0.98 | 0.67 | 0.46 | 0.98 |
| peginterferon beta 1a | 22 | 74,422 | 1,295 | 2,859,821 | 1,317 | 0.67 | 0.44 | 1.01 | 0.67 | 0.44 | 1.01 |
| raltegravir | 25 | 74,419 | 1,470 | 2,859,646 | 1,495 | 0.67 | 0.45 | 0.99 | 0.67 | 0.45 | 0.99 |
| dasatinib | 72 | 74,372 | 4,187 | 2,856,929 | 4,259 | 0.67 | 0.53 | 0.84 | 0.67 | 0.53 | 0.84 |
| exenatide | 46 | 74,398 | 2,688 | 2,858,428 | 2,734 | 0.66 | 0.50 | 0.89 | 0.66 | 0.50 | 0.89 |
| drospirenone ethinyl estradiol | 20 | 74,424 | 1,189 | 2,859,927 | 1,209 | 0.66 | 0.43 | 1.02 | 0.66 | 0.43 | 1.02 |
| amitriptyline | 36 | 74,408 | 2,124 | 2,858,992 | 2,160 | 0.66 | 0.48 | 0.92 | 0.66 | 0.48 | 0.92 |
| zoledronic acid | 89 | 74,355 | 5,218 | 2,855,898 | 5,307 | 0.66 | 0.53 | 0.81 | 0.66 | 0.53 | 0.81 |
| degarelix | 10 | 74,434 | 615 | 2,860,501 | 625 | 0.66 | 0.36 | 1.21 | 0.66 | 0.36 | 1.21 |
| pertuzumab | 92 | 74,352 | 5,437 | 2,855,679 | 5,529 | 0.65 | 0.53 | 0.80 | 0.65 | 0.53 | 0.80 |
| olanzapine | 92 | 74,352 | 5,439 | 2,855,677 | 5,531 | 0.65 | 0.53 | 0.80 | 0.65 | 0.53 | 0.80 |
| abobotulinumtoxina | 9 | 74,435 | 564 | 2,860,552 | 573 | 0.65 | 0.34 | 1.23 | 0.65 | 0.34 | 1.23 |
| sapropterin dihydrochloride | 16 | 74,428 | 980 | 2,860,136 | 996 | 0.65 | 0.40 | 1.05 | 0.65 | 0.40 | 1.05 |
| terbinafine | 12 | 74,432 | 747 | 2,860,369 | 759 | 0.64 | 0.37 | 1.12 | 0.64 | 0.37 | 1.12 |
| zolpidem | 37 | 74,407 | 2,249 | 2,858,867 | 2,286 | 0.64 | 0.46 | 0.88 | 0.64 | 0.46 | 0.88 |
| liraglutide | 54 | 74,390 | 3,274 | 2,857,842 | 3,328 | 0.64 | 0.49 | 0.84 | 0.64 | 0.49 | 0.84 |
| azithromycin | 50 | 74,394 | 3,045 | 2,858,071 | 3,095 | 0.64 | 0.48 | 0.84 | 0.64 | 0.48 | 0.84 |
| albuterol ipratropium bromide | 8 | 74,436 | 513 | 2,860,603 | 521 | 0.64 | 0.32 | 1.25 | 0.64 | 0.32 | 1.25 |
| capmatinib | 11 | 74,433 | 695 | 2,860,421 | 706 | 0.64 | 0.35 | 1.14 | 0.64 | 0.35 | 1.14 |
| ascorbic acid polyethylene glycol 3350 chloride ascorbate chloride | 9 | 74,435 | 575 | 2,860,541 | 584 | 0.63 | 0.33 | 1.20 | 0.63 | 0.33 | 1.20 |
| tetrabenazine | 6 | 74,438 | 395 | 2,860,721 | 401 | 0.63 | 0.29 | 1.37 | 0.63 | 0.29 | 1.37 |
| atorvastatin | 160 | 74,284 | 9,801 | 2,851,315 | 9,961 | 0.63 | 0.54 | 0.73 | 0.63 | 0.54 | 0.74 |
| velaglucerase alfa | 12 | 74,432 | 764 | 2,860,352 | 776 | 0.63 | 0.36 | 1.10 | 0.63 | 0.36 | 1.10 |
| romosozumab aqqg | 25 | 74,419 | 1,560 | 2,859,556 | 1,585 | 0.63 | 0.42 | 0.93 | 0.63 | 0.42 | 0.93 |
| ivacaftor tezacaftor | 21 | 74,423 | 1,323 | 2,859,793 | 1,344 | 0.62 | 0.41 | 0.96 | 0.62 | 0.41 | 0.96 |
| hydroxyurea | 16 | 74,428 | 1,016 | 2,860,100 | 1,032 | 0.62 | 0.38 | 1.01 | 0.62 | 0.38 | 1.01 |
| molnupiravir | 28 | 74,416 | 1,756 | 2,859,360 | 1,784 | 0.62 | 0.43 | 0.90 | 0.62 | 0.43 | 0.90 |
| ursodiol | 7 | 74,437 | 462 | 2,860,654 | 469 | 0.62 | 0.30 | 1.28 | 0.62 | 0.30 | 1.28 |
| cycloserine | 5 | 74,439 | 339 | 2,860,777 | 344 | 0.62 | 0.27 | 1.45 | 0.62 | 0.27 | 1.45 |
| doxycycline | 32 | 74,412 | 2,011 | 2,859,105 | 2,043 | 0.62 | 0.44 | 0.88 | 0.62 | 0.44 | 0.88 |
| colchicine | 16 | 74,428 | 1,022 | 2,860,094 | 1,038 | 0.62 | 0.38 | 1.01 | 0.62 | 0.38 | 1.01 |
| eltrombopag | 19 | 74,425 | 1,210 | 2,859,906 | 1,229 | 0.62 | 0.40 | 0.97 | 0.62 | 0.40 | 0.97 |
| emtricitabine tenofovir disoproxil | 148 | 74,296 | 9,220 | 2,851,896 | 9,368 | 0.62 | 0.53 | 0.73 | 0.62 | 0.53 | 0.73 |
| axitinib | 49 | 74,395 | 3,082 | 2,858,034 | 3,131 | 0.62 | 0.47 | 0.82 | 0.62 | 0.47 | 0.82 |
| hydrochlorothiazide irbesartan | 11 | 74,433 | 716 | 2,860,400 | 727 | 0.62 | 0.34 | 1.10 | 0.62 | 0.34 | 1.10 |
| testosterone | 11 | 74,433 | 716 | 2,860,400 | 727 | 0.62 | 0.34 | 1.10 | 0.62 | 0.34 | 1.10 |
| hyaluronidase zzxf pertuzumab trastuzumab | 10 | 74,434 | 655 | 2,860,461 | 665 | 0.62 | 0.33 | 1.13 | 0.62 | 0.33 | 1.13 |
| ado trastuzumab emtansine | 36 | 74,408 | 2,282 | 2,858,834 | 2,318 | 0.61 | 0.44 | 0.85 | 0.61 | 0.44 | 0.85 |
| idelalisib | 12 | 74,432 | 788 | 2,860,328 | 800 | 0.61 | 0.35 | 1.07 | 0.61 | 0.35 | 1.07 |
| diazepam | 69 | 74,375 | 4,409 | 2,856,707 | 4,478 | 0.61 | 0.48 | 0.77 | 0.61 | 0.48 | 0.77 |
| omeprazole | 215 | 74,229 | 13,690 | 2,847,426 | 13,905 | 0.60 | 0.53 | 0.69 | 0.60 | 0.53 | 0.69 |
| lutetium lu 177 vipivotide tetraxetan | 6 | 74,438 | 414 | 2,860,702 | 420 | 0.60 | 0.28 | 1.31 | 0.60 | 0.28 | 1.31 |
| galsulfase | 13 | 74,431 | 861 | 2,860,255 | 874 | 0.60 | 0.35 | 1.03 | 0.60 | 0.35 | 1.03 |
| denosumab | 95 | 74,349 | 6,124 | 2,854,992 | 6,219 | 0.60 | 0.49 | 0.73 | 0.60 | 0.49 | 0.73 |
| metronidazole | 42 | 74,402 | 2,730 | 2,858,386 | 2,772 | 0.60 | 0.44 | 0.81 | 0.60 | 0.44 | 0.81 |
| olodaterol tiotropium bromide | 7 | 74,437 | 483 | 2,860,633 | 490 | 0.60 | 0.29 | 1.23 | 0.60 | 0.29 | 1.23 |
| interferon beta 1b | 15 | 74,429 | 1,004 | 2,860,112 | 1,019 | 0.59 | 0.36 | 0.98 | 0.59 | 0.36 | 0.98 |
| teduglutide water | 5 | 74,439 | 358 | 2,860,758 | 363 | 0.59 | 0.25 | 1.37 | 0.59 | 0.25 | 1.37 |
| tezepelumab ekko | 10 | 74,434 | 684 | 2,860,432 | 694 | 0.59 | 0.32 | 1.08 | 0.59 | 0.32 | 1.08 |
| trastuzumab | 135 | 74,309 | 8,824 | 2,852,292 | 8,959 | 0.59 | 0.50 | 0.70 | 0.59 | 0.50 | 0.70 |
| everolimus | 78 | 74,366 | 5,164 | 2,855,952 | 5,242 | 0.58 | 0.47 | 0.73 | 0.58 | 0.47 | 0.73 |
| foscarbidopa foslevodopa | 7 | 74,437 | 495 | 2,860,621 | 502 | 0.58 | 0.28 | 1.20 | 0.58 | 0.28 | 1.20 |
| capecitabine | 141 | 74,303 | 9,377 | 2,851,739 | 9,518 | 0.58 | 0.49 | 0.68 | 0.58 | 0.49 | 0.68 |
| mesalamine | 31 | 74,413 | 2,092 | 2,859,024 | 2,123 | 0.58 | 0.41 | 0.82 | 0.58 | 0.41 | 0.82 |
| insulin glargine | 80 | 74,364 | 5,351 | 2,855,765 | 5,431 | 0.58 | 0.46 | 0.72 | 0.58 | 0.46 | 0.72 |
| alectinib | 44 | 74,400 | 2,990 | 2,858,126 | 3,034 | 0.57 | 0.43 | 0.77 | 0.57 | 0.43 | 0.77 |
| olaparib | 60 | 74,384 | 4,074 | 2,857,042 | 4,134 | 0.57 | 0.44 | 0.74 | 0.57 | 0.44 | 0.74 |
| imatinib | 64 | 74,380 | 4,345 | 2,856,771 | 4,409 | 0.57 | 0.45 | 0.73 | 0.57 | 0.45 | 0.73 |
| elotuzumab | 10 | 74,434 | 710 | 2,860,406 | 720 | 0.57 | 0.31 | 1.04 | 0.57 | 0.31 | 1.04 |
| lithium | 25 | 74,419 | 1,727 | 2,859,389 | 1,752 | 0.57 | 0.38 | 0.84 | 0.57 | 0.38 | 0.84 |
| ranolazine | 10 | 74,434 | 712 | 2,860,404 | 722 | 0.57 | 0.31 | 1.04 | 0.57 | 0.31 | 1.04 |
| pegvisomant | 14 | 74,430 | 984 | 2,860,132 | 998 | 0.57 | 0.34 | 0.95 | 0.57 | 0.34 | 0.95 |
| phenytoin | 21 | 74,423 | 1,466 | 2,859,650 | 1,487 | 0.56 | 0.37 | 0.86 | 0.56 | 0.37 | 0.86 |
| oxcarbazepine | 20 | 74,424 | 1,408 | 2,859,708 | 1,428 | 0.56 | 0.36 | 0.86 | 0.56 | 0.36 | 0.87 |
| ranitidine | 44 | 74,400 | 3,060 | 2,858,056 | 3,104 | 0.56 | 0.42 | 0.75 | 0.56 | 0.42 | 0.75 |
| theophylline | 5 | 74,439 | 378 | 2,860,738 | 383 | 0.56 | 0.24 | 1.30 | 0.56 | 0.24 | 1.30 |
| hydrochlorothiazide olmesartan medoxomil | 9 | 74,435 | 657 | 2,860,459 | 666 | 0.56 | 0.29 | 1.05 | 0.56 | 0.29 | 1.05 |
| tenofovir alafenamide | 13 | 74,431 | 941 | 2,860,175 | 954 | 0.55 | 0.32 | 0.94 | 0.55 | 0.32 | 0.94 |
| riluzole | 6 | 74,438 | 454 | 2,860,662 | 460 | 0.55 | 0.25 | 1.19 | 0.55 | 0.25 | 1.19 |
| pravastatin | 19 | 74,425 | 1,373 | 2,859,743 | 1,392 | 0.55 | 0.35 | 0.85 | 0.55 | 0.35 | 0.85 |
| scopolamine | 6 | 74,438 | 463 | 2,860,653 | 469 | 0.54 | 0.25 | 1.17 | 0.54 | 0.25 | 1.17 |
| ketamine | 25 | 74,419 | 1,834 | 2,859,282 | 1,859 | 0.53 | 0.36 | 0.79 | 0.53 | 0.36 | 0.79 |
| tadalafil | 49 | 74,395 | 3,576 | 2,857,540 | 3,625 | 0.53 | 0.40 | 0.70 | 0.53 | 0.40 | 0.70 |
| efavirenz emtricitabine tenofovir disoproxil | 81 | 74,363 | 5,899 | 2,855,217 | 5,980 | 0.53 | 0.43 | 0.66 | 0.53 | 0.43 | 0.66 |
| clonidine | 20 | 74,424 | 1,485 | 2,859,631 | 1,505 | 0.53 | 0.34 | 0.82 | 0.53 | 0.34 | 0.82 |
| amlodipine besylate valsartan | 10 | 74,434 | 767 | 2,860,349 | 777 | 0.53 | 0.29 | 0.97 | 0.53 | 0.29 | 0.97 |
| glutamine | 13 | 74,431 | 989 | 2,860,127 | 1,002 | 0.52 | 0.31 | 0.90 | 0.52 | 0.31 | 0.90 |
| fedratinib | 10 | 74,434 | 770 | 2,860,346 | 780 | 0.52 | 0.28 | 0.96 | 0.52 | 0.28 | 0.96 |
| brodalumab | 15 | 74,429 | 1,142 | 2,859,974 | 1,157 | 0.52 | 0.32 | 0.86 | 0.52 | 0.32 | 0.86 |
| metreleptin | 5 | 74,439 | 406 | 2,860,710 | 411 | 0.52 | 0.22 | 1.21 | 0.52 | 0.22 | 1.21 |
| memantine | 8 | 74,436 | 628 | 2,860,488 | 636 | 0.52 | 0.26 | 1.02 | 0.52 | 0.26 | 1.02 |
| esomeprazole naproxen | 11 | 74,433 | 851 | 2,860,265 | 862 | 0.52 | 0.29 | 0.93 | 0.52 | 0.29 | 0.93 |
| cocaine | 9 | 74,435 | 703 | 2,860,413 | 712 | 0.52 | 0.27 | 0.98 | 0.52 | 0.27 | 0.98 |
| tislelizumab | 6 | 74,438 | 481 | 2,860,635 | 487 | 0.52 | 0.24 | 1.12 | 0.52 | 0.24 | 1.12 |
| asciminib | 8 | 74,436 | 640 | 2,860,476 | 648 | 0.51 | 0.26 | 1.00 | 0.51 | 0.26 | 1.00 |
| lapatinib ditosylate | 8 | 74,436 | 643 | 2,860,473 | 651 | 0.51 | 0.26 | 1.00 | 0.51 | 0.26 | 1.00 |
| crizanlizumab tmca | 6 | 74,438 | 495 | 2,860,621 | 501 | 0.50 | 0.23 | 1.09 | 0.50 | 0.23 | 1.09 |
| dornase alfa | 14 | 74,430 | 1,110 | 2,860,006 | 1,124 | 0.50 | 0.30 | 0.84 | 0.50 | 0.30 | 0.84 |
| encorafenib | 42 | 74,402 | 3,255 | 2,857,861 | 3,297 | 0.50 | 0.37 | 0.68 | 0.50 | 0.37 | 0.68 |
| cilastatin imipenem | 22 | 74,422 | 1,725 | 2,859,391 | 1,747 | 0.50 | 0.33 | 0.76 | 0.50 | 0.33 | 0.76 |
| bedaquiline | 23 | 74,421 | 1,806 | 2,859,310 | 1,829 | 0.50 | 0.33 | 0.75 | 0.50 | 0.33 | 0.75 |
| carbamazepine | 40 | 74,404 | 3,113 | 2,858,003 | 3,153 | 0.50 | 0.37 | 0.68 | 0.50 | 0.37 | 0.68 |
| dexlansoprazole | 24 | 74,420 | 1,888 | 2,859,228 | 1,912 | 0.50 | 0.33 | 0.74 | 0.50 | 0.33 | 0.74 |
| insulin human | 34 | 74,410 | 2,661 | 2,858,455 | 2,695 | 0.50 | 0.36 | 0.70 | 0.50 | 0.36 | 0.70 |
| casirivimab imdevimab | 9 | 74,435 | 735 | 2,860,381 | 744 | 0.50 | 0.26 | 0.94 | 0.50 | 0.26 | 0.94 |
| mycophenolate mofetil | 55 | 74,389 | 4,305 | 2,856,811 | 4,360 | 0.50 | 0.38 | 0.65 | 0.50 | 0.38 | 0.65 |
| gadoterate meglumine | 15 | 74,429 | 1,204 | 2,859,912 | 1,219 | 0.49 | 0.30 | 0.82 | 0.49 | 0.30 | 0.82 |
| lanadelumab | 14 | 74,430 | 1,127 | 2,859,989 | 1,141 | 0.49 | 0.29 | 0.83 | 0.49 | 0.29 | 0.83 |
| enalapril | 16 | 74,428 | 1,288 | 2,859,828 | 1,304 | 0.49 | 0.30 | 0.80 | 0.49 | 0.30 | 0.80 |
| haloperidol | 30 | 74,414 | 2,392 | 2,858,724 | 2,422 | 0.49 | 0.34 | 0.70 | 0.49 | 0.34 | 0.70 |
| cefuroxime axetil | 5 | 74,439 | 433 | 2,860,683 | 438 | 0.49 | 0.21 | 1.13 | 0.49 | 0.21 | 1.13 |
| spironolactone | 48 | 74,396 | 3,823 | 2,857,293 | 3,871 | 0.49 | 0.37 | 0.65 | 0.49 | 0.37 | 0.65 |
| insulin lispro | 48 | 74,396 | 3,835 | 2,857,281 | 3,883 | 0.49 | 0.37 | 0.64 | 0.49 | 0.37 | 0.65 |
| zopiclone | 22 | 74,422 | 1,782 | 2,859,334 | 1,804 | 0.48 | 0.32 | 0.74 | 0.49 | 0.32 | 0.74 |
| bimekizumab bkzx | 6 | 74,438 | 515 | 2,860,601 | 521 | 0.48 | 0.22 | 1.05 | 0.48 | 0.22 | 1.05 |
| fenofibrate | 15 | 74,429 | 1,231 | 2,859,885 | 1,246 | 0.48 | 0.29 | 0.80 | 0.48 | 0.29 | 0.80 |
| hydroxyzine | 23 | 74,421 | 1,869 | 2,859,247 | 1,892 | 0.48 | 0.32 | 0.73 | 0.48 | 0.32 | 0.73 |
| fulvestrant | 49 | 74,395 | 3,948 | 2,857,168 | 3,997 | 0.48 | 0.36 | 0.64 | 0.48 | 0.36 | 0.64 |
| metoclopramide | 20 | 74,424 | 1,646 | 2,859,470 | 1,666 | 0.48 | 0.31 | 0.74 | 0.48 | 0.31 | 0.74 |
| triptorelin pamoate | 8 | 74,436 | 684 | 2,860,432 | 692 | 0.48 | 0.24 | 0.94 | 0.48 | 0.24 | 0.94 |
| torsemide | 19 | 74,425 | 1,570 | 2,859,546 | 1,589 | 0.48 | 0.31 | 0.75 | 0.48 | 0.31 | 0.75 |
| entecavir | 8 | 74,436 | 685 | 2,860,431 | 693 | 0.48 | 0.24 | 0.94 | 0.48 | 0.24 | 0.94 |
| incobotulinumtoxina | 5 | 74,439 | 445 | 2,860,671 | 450 | 0.47 | 0.20 | 1.10 | 0.47 | 0.20 | 1.10 |
| metformin sitagliptin | 24 | 74,420 | 1,995 | 2,859,121 | 2,019 | 0.47 | 0.32 | 0.70 | 0.47 | 0.32 | 0.70 |
| fluconazole | 29 | 74,415 | 2,404 | 2,858,712 | 2,433 | 0.47 | 0.33 | 0.68 | 0.47 | 0.33 | 0.68 |
| enasidenib | 20 | 74,424 | 1,673 | 2,859,443 | 1,693 | 0.47 | 0.30 | 0.73 | 0.47 | 0.30 | 0.73 |
| palivizumab | 20 | 74,424 | 1,685 | 2,859,431 | 1,705 | 0.47 | 0.30 | 0.72 | 0.47 | 0.30 | 0.72 |
| dapagliflozin propanediol metformin | 11 | 74,433 | 949 | 2,860,167 | 960 | 0.47 | 0.26 | 0.83 | 0.47 | 0.26 | 0.83 |
| sitagliptin | 46 | 74,398 | 3,837 | 2,857,279 | 3,883 | 0.47 | 0.35 | 0.62 | 0.47 | 0.35 | 0.62 |
| goserelin | 17 | 74,427 | 1,446 | 2,859,670 | 1,463 | 0.46 | 0.29 | 0.74 | 0.46 | 0.29 | 0.74 |
| venetoclax | 159 | 74,285 | 13,174 | 2,847,942 | 13,333 | 0.46 | 0.40 | 0.54 | 0.47 | 0.40 | 0.54 |
| dexamethasone | 188 | 74,256 | 15,587 | 2,845,529 | 15,775 | 0.46 | 0.40 | 0.54 | 0.46 | 0.40 | 0.54 |
| ritonavir | 15 | 74,429 | 1,288 | 2,859,828 | 1,303 | 0.46 | 0.28 | 0.76 | 0.46 | 0.28 | 0.76 |
| sacituzumab govitecan | 6 | 74,438 | 541 | 2,860,575 | 547 | 0.46 | 0.21 | 1.00 | 0.46 | 0.21 | 1.00 |
| pazopanib | 34 | 74,410 | 2,892 | 2,858,224 | 2,926 | 0.46 | 0.33 | 0.64 | 0.46 | 0.33 | 0.64 |
| atorvastatin ezetimibe | 5 | 74,439 | 463 | 2,860,653 | 468 | 0.46 | 0.20 | 1.06 | 0.46 | 0.20 | 1.06 |
| osimertinib | 69 | 74,375 | 5,891 | 2,855,225 | 5,960 | 0.45 | 0.36 | 0.57 | 0.45 | 0.36 | 0.57 |
| baloxavir marboxil | 6 | 74,438 | 559 | 2,860,557 | 565 | 0.45 | 0.21 | 0.97 | 0.45 | 0.21 | 0.97 |
| entrectinib | 7 | 74,437 | 647 | 2,860,469 | 654 | 0.45 | 0.22 | 0.91 | 0.45 | 0.22 | 0.91 |
| selumetinib | 5 | 74,439 | 476 | 2,860,640 | 481 | 0.44 | 0.19 | 1.03 | 0.44 | 0.19 | 1.03 |
| trametinib dimethyl sulfoxide | 45 | 74,399 | 3,963 | 2,857,153 | 4,008 | 0.44 | 0.33 | 0.59 | 0.44 | 0.33 | 0.59 |
| darunavir ethanolate | 11 | 74,433 | 1,002 | 2,860,114 | 1,013 | 0.44 | 0.25 | 0.79 | 0.44 | 0.25 | 0.79 |
| eplerenone | 7 | 74,437 | 654 | 2,860,462 | 661 | 0.44 | 0.21 | 0.90 | 0.44 | 0.21 | 0.90 |
| lamivudine | 10 | 74,434 | 918 | 2,860,198 | 928 | 0.44 | 0.24 | 0.81 | 0.44 | 0.24 | 0.81 |
| metformin | 119 | 74,325 | 10,465 | 2,850,651 | 10,584 | 0.44 | 0.37 | 0.52 | 0.44 | 0.37 | 0.53 |
| loperamide | 24 | 74,420 | 2,154 | 2,858,962 | 2,178 | 0.44 | 0.29 | 0.65 | 0.44 | 0.29 | 0.65 |
| solifenacin | 9 | 74,435 | 838 | 2,860,278 | 847 | 0.44 | 0.23 | 0.83 | 0.44 | 0.23 | 0.83 |
| nitroglycerin | 9 | 74,435 | 839 | 2,860,277 | 848 | 0.43 | 0.23 | 0.82 | 0.43 | 0.23 | 0.82 |
| sulfamethoxazole trimethoprim | 64 | 74,380 | 5,696 | 2,855,420 | 5,760 | 0.43 | 0.34 | 0.56 | 0.44 | 0.34 | 0.56 |
| esomeprazole | 131 | 74,313 | 11,874 | 2,849,242 | 12,005 | 0.42 | 0.36 | 0.50 | 0.43 | 0.36 | 0.51 |
| sumatriptan | 11 | 74,433 | 1,042 | 2,860,074 | 1,053 | 0.42 | 0.24 | 0.76 | 0.42 | 0.24 | 0.76 |
| dofetilide | 9 | 74,435 | 870 | 2,860,246 | 879 | 0.42 | 0.22 | 0.79 | 0.42 | 0.22 | 0.79 |
| sotalol | 5 | 74,439 | 504 | 2,860,612 | 509 | 0.42 | 0.18 | 0.97 | 0.42 | 0.18 | 0.97 |
| tipiracil trifluridine | 32 | 74,412 | 2,985 | 2,858,131 | 3,017 | 0.42 | 0.30 | 0.59 | 0.42 | 0.30 | 0.59 |
| eliglustat | 6 | 74,438 | 599 | 2,860,517 | 605 | 0.42 | 0.19 | 0.90 | 0.42 | 0.19 | 0.90 |
| follitropin | 5 | 74,439 | 509 | 2,860,607 | 514 | 0.41 | 0.18 | 0.96 | 0.41 | 0.18 | 0.96 |
| deucravacitinib | 8 | 74,436 | 787 | 2,860,329 | 795 | 0.41 | 0.21 | 0.82 | 0.41 | 0.21 | 0.82 |
| nivolumab | 171 | 74,273 | 15,853 | 2,845,263 | 16,024 | 0.41 | 0.36 | 0.48 | 0.42 | 0.36 | 0.48 |
| sorafenib | 16 | 74,428 | 1,534 | 2,859,582 | 1,550 | 0.41 | 0.25 | 0.67 | 0.41 | 0.25 | 0.67 |
| chloride | 65 | 74,379 | 6,097 | 2,855,019 | 6,162 | 0.41 | 0.32 | 0.53 | 0.41 | 0.32 | 0.53 |
| gentamicin | 10 | 74,434 | 978 | 2,860,138 | 988 | 0.41 | 0.22 | 0.76 | 0.41 | 0.22 | 0.76 |
| insulin detemir | 16 | 74,428 | 1,563 | 2,859,553 | 1,579 | 0.41 | 0.25 | 0.66 | 0.41 | 0.25 | 0.66 |
| fludarabine | 25 | 74,419 | 2,417 | 2,858,699 | 2,442 | 0.41 | 0.27 | 0.60 | 0.41 | 0.27 | 0.60 |
| tenofovir disoproxil | 53 | 74,391 | 5,094 | 2,856,022 | 5,147 | 0.40 | 0.31 | 0.53 | 0.40 | 0.31 | 0.53 |
| dabrafenib | 49 | 74,395 | 4,725 | 2,856,391 | 4,774 | 0.40 | 0.30 | 0.53 | 0.40 | 0.30 | 0.53 |
| binimetinib | 27 | 74,417 | 2,638 | 2,858,478 | 2,665 | 0.40 | 0.27 | 0.58 | 0.40 | 0.28 | 0.58 |
| clomipramine | 6 | 74,438 | 627 | 2,860,489 | 633 | 0.40 | 0.18 | 0.86 | 0.40 | 0.18 | 0.86 |
| irbesartan | 23 | 74,421 | 2,270 | 2,858,846 | 2,293 | 0.40 | 0.26 | 0.60 | 0.40 | 0.26 | 0.60 |
| onasemnogene abeparvovec xioi | 10 | 74,434 | 1,024 | 2,860,092 | 1,034 | 0.39 | 0.21 | 0.72 | 0.39 | 0.21 | 0.72 |
| ferrous | 7 | 74,437 | 734 | 2,860,382 | 741 | 0.39 | 0.19 | 0.81 | 0.39 | 0.19 | 0.81 |
| clindamycin | 23 | 74,421 | 2,316 | 2,858,800 | 2,339 | 0.39 | 0.26 | 0.59 | 0.39 | 0.26 | 0.59 |
| thalidomide | 26 | 74,418 | 2,626 | 2,858,490 | 2,652 | 0.39 | 0.26 | 0.57 | 0.39 | 0.26 | 0.57 |
| cobicistat elvitegravir emtricitabine tenofovir disoproxil | 34 | 74,410 | 3,429 | 2,857,687 | 3,463 | 0.39 | 0.28 | 0.54 | 0.39 | 0.28 | 0.54 |
| ipilimumab | 72 | 74,372 | 7,222 | 2,853,894 | 7,294 | 0.39 | 0.31 | 0.49 | 0.39 | 0.31 | 0.49 |
| ethambutol | 8 | 74,436 | 849 | 2,860,267 | 857 | 0.38 | 0.20 | 0.76 | 0.38 | 0.20 | 0.76 |
| nifedipine | 9 | 74,435 | 956 | 2,860,160 | 965 | 0.38 | 0.20 | 0.72 | 0.38 | 0.20 | 0.72 |
| hydralazine | 5 | 74,439 | 555 | 2,860,561 | 560 | 0.38 | 0.16 | 0.88 | 0.38 | 0.16 | 0.88 |
| tobramycin | 15 | 74,429 | 1,578 | 2,859,538 | 1,593 | 0.38 | 0.23 | 0.62 | 0.38 | 0.23 | 0.62 |
| deferasirox | 16 | 74,428 | 1,680 | 2,859,436 | 1,696 | 0.38 | 0.23 | 0.61 | 0.38 | 0.23 | 0.61 |
| bevacizumab | 99 | 74,345 | 10,119 | 2,850,997 | 10,218 | 0.38 | 0.31 | 0.46 | 0.38 | 0.31 | 0.46 |
| codeine | 8 | 74,436 | 872 | 2,860,244 | 880 | 0.37 | 0.19 | 0.74 | 0.37 | 0.19 | 0.74 |
| edaravone | 6 | 74,438 | 667 | 2,860,449 | 673 | 0.37 | 0.17 | 0.81 | 0.37 | 0.17 | 0.81 |
| olmesartan medoxomil | 12 | 74,432 | 1,290 | 2,859,826 | 1,302 | 0.37 | 0.21 | 0.65 | 0.37 | 0.21 | 0.65 |
| tralokinumab ldrm | 8 | 74,436 | 888 | 2,860,228 | 896 | 0.37 | 0.19 | 0.72 | 0.37 | 0.19 | 0.72 |
| pyrazinamide | 6 | 74,438 | 683 | 2,860,433 | 689 | 0.37 | 0.17 | 0.79 | 0.37 | 0.17 | 0.79 |
| itraconazole | 7 | 74,437 | 789 | 2,860,327 | 796 | 0.37 | 0.18 | 0.75 | 0.37 | 0.18 | 0.75 |
| leucovorin | 5 | 74,439 | 580 | 2,860,536 | 585 | 0.36 | 0.16 | 0.84 | 0.36 | 0.16 | 0.84 |
| daratumumab | 71 | 74,373 | 7,535 | 2,853,581 | 7,606 | 0.36 | 0.29 | 0.46 | 0.36 | 0.29 | 0.46 |
| crizotinib | 19 | 74,425 | 2,066 | 2,859,050 | 2,085 | 0.36 | 0.23 | 0.57 | 0.36 | 0.23 | 0.57 |
| amiodarone | 37 | 74,407 | 3,975 | 2,857,141 | 4,012 | 0.36 | 0.26 | 0.50 | 0.36 | 0.26 | 0.50 |
| bortezomib | 53 | 74,391 | 5,767 | 2,855,349 | 5,820 | 0.36 | 0.27 | 0.47 | 0.36 | 0.27 | 0.47 |
| tremelimumab | 9 | 74,435 | 1,025 | 2,860,091 | 1,034 | 0.36 | 0.19 | 0.67 | 0.36 | 0.19 | 0.67 |
| erythropoietin | 7 | 74,437 | 821 | 2,860,295 | 828 | 0.35 | 0.17 | 0.72 | 0.35 | 0.17 | 0.72 |
| emtricitabine rilpivirine tenofovir disoproxil | 26 | 74,418 | 2,944 | 2,858,172 | 2,970 | 0.35 | 0.24 | 0.51 | 0.35 | 0.24 | 0.51 |
| elosulfase alfa | 10 | 74,434 | 1,175 | 2,859,941 | 1,185 | 0.34 | 0.19 | 0.63 | 0.34 | 0.19 | 0.63 |
| delamanid | 5 | 74,439 | 622 | 2,860,494 | 627 | 0.34 | 0.15 | 0.79 | 0.34 | 0.15 | 0.79 |
| ertapenem | 9 | 74,435 | 1,088 | 2,860,028 | 1,097 | 0.34 | 0.18 | 0.64 | 0.34 | 0.18 | 0.64 |
| ivabradine | 6 | 74,438 | 753 | 2,860,363 | 759 | 0.33 | 0.15 | 0.72 | 0.33 | 0.15 | 0.72 |
| cilgavimab tixagevimab | 6 | 74,438 | 756 | 2,860,360 | 762 | 0.33 | 0.15 | 0.71 | 0.33 | 0.15 | 0.71 |
| agalsidase beta | 15 | 74,429 | 1,805 | 2,859,311 | 1,820 | 0.33 | 0.20 | 0.54 | 0.33 | 0.20 | 0.54 |
| pembrolizumab | 118 | 74,326 | 14,015 | 2,847,101 | 14,133 | 0.32 | 0.27 | 0.39 | 0.32 | 0.27 | 0.39 |
| ivacaftor lumacaftor | 11 | 74,433 | 1,375 | 2,859,741 | 1,386 | 0.32 | 0.18 | 0.57 | 0.32 | 0.18 | 0.57 |
| cyclophosphamide | 139 | 74,305 | 16,665 | 2,844,451 | 16,804 | 0.32 | 0.27 | 0.38 | 0.32 | 0.27 | 0.38 |
| docetaxel | 7 | 74,437 | 900 | 2,860,216 | 907 | 0.32 | 0.16 | 0.66 | 0.32 | 0.16 | 0.66 |
| mycophenolate mofetil mycophenolate mofetil | 10 | 74,434 | 1,265 | 2,859,851 | 1,275 | 0.32 | 0.17 | 0.59 | 0.32 | 0.17 | 0.59 |
| afatinib | 6 | 74,438 | 783 | 2,860,333 | 789 | 0.32 | 0.15 | 0.69 | 0.32 | 0.15 | 0.69 |
| sacituzumab govitecan hziy | 6 | 74,438 | 784 | 2,860,332 | 790 | 0.32 | 0.15 | 0.69 | 0.32 | 0.15 | 0.69 |
| docetaxel | 60 | 74,384 | 7,306 | 2,853,810 | 7,366 | 0.32 | 0.25 | 0.41 | 0.32 | 0.25 | 0.41 |
| tafamidis meglumine | 10 | 74,434 | 1,274 | 2,859,842 | 1,284 | 0.32 | 0.17 | 0.58 | 0.32 | 0.17 | 0.58 |
| midostaurin | 7 | 74,437 | 918 | 2,860,198 | 925 | 0.31 | 0.15 | 0.64 | 0.31 | 0.15 | 0.64 |
| tacrolimus | 63 | 74,381 | 7,793 | 2,853,323 | 7,856 | 0.31 | 0.24 | 0.40 | 0.31 | 0.24 | 0.40 |
| brentuximab vedotin | 19 | 74,425 | 2,409 | 2,858,707 | 2,428 | 0.31 | 0.20 | 0.49 | 0.31 | 0.20 | 0.49 |
| desloratadine | 6 | 74,438 | 804 | 2,860,312 | 810 | 0.31 | 0.14 | 0.67 | 0.31 | 0.14 | 0.67 |
| ondansetron | 28 | 74,416 | 3,527 | 2,857,589 | 3,555 | 0.31 | 0.21 | 0.45 | 0.31 | 0.21 | 0.45 |
| doxazosin | 6 | 74,438 | 805 | 2,860,311 | 811 | 0.31 | 0.14 | 0.67 | 0.31 | 0.14 | 0.67 |
| clozapine | 87 | 74,357 | 10,958 | 2,850,158 | 11,045 | 0.31 | 0.25 | 0.38 | 0.31 | 0.25 | 0.38 |
| paclitaxel | 106 | 74,338 | 13,347 | 2,847,769 | 13,453 | 0.31 | 0.25 | 0.37 | 0.31 | 0.25 | 0.37 |
| rivaroxaban | 152 | 74,292 | 19,099 | 2,842,017 | 19,251 | 0.31 | 0.26 | 0.36 | 0.31 | 0.26 | 0.36 |
| valproate | 22 | 74,422 | 2,879 | 2,858,237 | 2,901 | 0.30 | 0.20 | 0.45 | 0.30 | 0.20 | 0.45 |
| vemurafenib | 10 | 74,434 | 1,346 | 2,859,770 | 1,356 | 0.30 | 0.16 | 0.55 | 0.30 | 0.16 | 0.55 |
| romiplostim | 6 | 74,438 | 858 | 2,860,258 | 864 | 0.29 | 0.13 | 0.63 | 0.29 | 0.13 | 0.63 |
| amoxicillin clavulanate | 21 | 74,423 | 2,849 | 2,858,267 | 2,870 | 0.29 | 0.19 | 0.44 | 0.29 | 0.19 | 0.44 |
| lidocaine | 12 | 74,432 | 1,668 | 2,859,448 | 1,680 | 0.29 | 0.16 | 0.50 | 0.29 | 0.17 | 0.50 |
| ferric carboxymaltose | 14 | 74,430 | 1,967 | 2,859,149 | 1,981 | 0.28 | 0.17 | 0.47 | 0.28 | 0.17 | 0.47 |
| darbepoetin alfa | 11 | 74,433 | 1,561 | 2,859,555 | 1,572 | 0.28 | 0.16 | 0.51 | 0.28 | 0.16 | 0.51 |
| tigecycline | 6 | 74,438 | 884 | 2,860,232 | 890 | 0.28 | 0.13 | 0.61 | 0.28 | 0.13 | 0.61 |
| daratumumab hyaluronidase fihj | 5 | 74,439 | 751 | 2,860,365 | 756 | 0.28 | 0.12 | 0.65 | 0.28 | 0.12 | 0.65 |
| valproic acid | 11 | 74,433 | 1,689 | 2,859,427 | 1,700 | 0.26 | 0.15 | 0.47 | 0.26 | 0.15 | 0.47 |
| deferiprone | 6 | 74,438 | 955 | 2,860,161 | 961 | 0.26 | 0.12 | 0.57 | 0.26 | 0.12 | 0.57 |
| ixekizumab | 27 | 74,417 | 4,040 | 2,857,076 | 4,067 | 0.26 | 0.18 | 0.38 | 0.26 | 0.18 | 0.38 |
| filgrastim | 12 | 74,432 | 1,858 | 2,859,258 | 1,870 | 0.26 | 0.15 | 0.45 | 0.26 | 0.15 | 0.45 |
| risdiplam | 6 | 74,438 | 967 | 2,860,149 | 973 | 0.26 | 0.12 | 0.56 | 0.26 | 0.12 | 0.56 |
| sirolimus | 11 | 74,433 | 1,717 | 2,859,399 | 1,728 | 0.26 | 0.14 | 0.46 | 0.26 | 0.14 | 0.46 |
| alglucosidase alfa | 6 | 74,438 | 971 | 2,860,145 | 977 | 0.26 | 0.12 | 0.56 | 0.26 | 0.12 | 0.56 |
| bromazepam | 7 | 74,437 | 1,129 | 2,859,987 | 1,136 | 0.26 | 0.12 | 0.52 | 0.26 | 0.12 | 0.52 |
| cephalexin | 5 | 74,439 | 829 | 2,860,287 | 834 | 0.25 | 0.11 | 0.59 | 0.25 | 0.11 | 0.59 |
| patiromer | 9 | 74,435 | 1,445 | 2,859,671 | 1,454 | 0.25 | 0.13 | 0.48 | 0.25 | 0.13 | 0.48 |
| axicabtagene ciloleucel | 18 | 74,426 | 2,832 | 2,858,284 | 2,850 | 0.25 | 0.16 | 0.40 | 0.25 | 0.16 | 0.40 |
| mycophenolate | 6 | 74,438 | 1,003 | 2,860,113 | 1,009 | 0.25 | 0.12 | 0.54 | 0.25 | 0.12 | 0.54 |
| erlotinib | 9 | 74,435 | 1,469 | 2,859,647 | 1,478 | 0.25 | 0.13 | 0.47 | 0.25 | 0.13 | 0.47 |
| durvalumab | 29 | 74,415 | 4,565 | 2,856,551 | 4,594 | 0.25 | 0.17 | 0.36 | 0.25 | 0.17 | 0.36 |
| amoxicillin | 29 | 74,415 | 4,646 | 2,856,470 | 4,675 | 0.24 | 0.17 | 0.35 | 0.24 | 0.17 | 0.35 |
| linezolid | 27 | 74,417 | 4,334 | 2,856,782 | 4,361 | 0.24 | 0.17 | 0.35 | 0.24 | 0.17 | 0.35 |
| fluorouracil | 51 | 74,393 | 8,121 | 2,852,995 | 8,172 | 0.24 | 0.18 | 0.32 | 0.24 | 0.19 | 0.32 |
| daunorubicin | 7 | 74,437 | 1,215 | 2,859,901 | 1,222 | 0.24 | 0.12 | 0.49 | 0.24 | 0.12 | 0.49 |
| furosemide | 54 | 74,390 | 8,833 | 2,852,283 | 8,887 | 0.24 | 0.18 | 0.31 | 0.24 | 0.18 | 0.31 |
| ethinyl estradiol levonorgestrel | 5 | 74,439 | 900 | 2,860,216 | 905 | 0.23 | 0.10 | 0.54 | 0.23 | 0.10 | 0.54 |
| posaconazole | 9 | 74,435 | 1,574 | 2,859,542 | 1,583 | 0.23 | 0.12 | 0.44 | 0.23 | 0.12 | 0.44 |
| atezolizumab | 56 | 74,388 | 9,412 | 2,851,704 | 9,468 | 0.23 | 0.18 | 0.30 | 0.23 | 0.18 | 0.30 |
| canagliflozin | 16 | 74,428 | 2,756 | 2,858,360 | 2,772 | 0.23 | 0.14 | 0.37 | 0.23 | 0.14 | 0.37 |
| gadobutrol | 5 | 74,439 | 923 | 2,860,193 | 928 | 0.23 | 0.10 | 0.53 | 0.23 | 0.10 | 0.53 |
| cyamemazine | 6 | 74,438 | 1,096 | 2,860,020 | 1,102 | 0.23 | 0.11 | 0.49 | 0.23 | 0.11 | 0.49 |
| irinotecan | 27 | 74,417 | 4,698 | 2,856,418 | 4,725 | 0.22 | 0.15 | 0.33 | 0.22 | 0.15 | 0.33 |
| obinutuzumab | 28 | 74,416 | 4,872 | 2,856,244 | 4,900 | 0.22 | 0.16 | 0.32 | 0.22 | 0.16 | 0.32 |
| guselkumab | 25 | 74,419 | 4,394 | 2,856,722 | 4,419 | 0.22 | 0.15 | 0.33 | 0.22 | 0.15 | 0.33 |
| rifampin | 11 | 74,433 | 2,032 | 2,859,084 | 2,043 | 0.22 | 0.12 | 0.39 | 0.22 | 0.12 | 0.39 |
| panitumumab | 10 | 74,434 | 1,894 | 2,859,222 | 1,904 | 0.21 | 0.12 | 0.39 | 0.21 | 0.12 | 0.39 |
| linagliptin | 5 | 74,439 | 995 | 2,860,121 | 1,000 | 0.21 | 0.09 | 0.49 | 0.21 | 0.09 | 0.49 |
| carboplatin | 66 | 74,378 | 12,048 | 2,849,068 | 12,114 | 0.21 | 0.17 | 0.27 | 0.21 | 0.17 | 0.27 |
| warfarin | 16 | 74,428 | 3,034 | 2,858,082 | 3,050 | 0.21 | 0.13 | 0.34 | 0.21 | 0.13 | 0.34 |
| pentosan polysulfate | 6 | 74,438 | 1,212 | 2,859,904 | 1,218 | 0.21 | 0.10 | 0.45 | 0.21 | 0.10 | 0.45 |
| unspecified ingredient | 17 | 74,427 | 3,326 | 2,857,790 | 3,343 | 0.20 | 0.13 | 0.32 | 0.20 | 0.13 | 0.32 |
| aztreonam lysine | 9 | 74,435 | 1,810 | 2,859,306 | 1,819 | 0.20 | 0.11 | 0.38 | 0.20 | 0.11 | 0.38 |
| gefitinib | 8 | 74,436 | 1,624 | 2,859,492 | 1,632 | 0.20 | 0.10 | 0.39 | 0.20 | 0.10 | 0.39 |
| pegfilgrastim | 12 | 74,432 | 2,416 | 2,858,700 | 2,428 | 0.20 | 0.11 | 0.35 | 0.20 | 0.11 | 0.35 |
| leucovorin | 18 | 74,426 | 3,578 | 2,857,538 | 3,596 | 0.20 | 0.13 | 0.31 | 0.20 | 0.13 | 0.31 |
| cabozantinib | 8 | 74,436 | 1,651 | 2,859,465 | 1,659 | 0.20 | 0.10 | 0.39 | 0.20 | 0.10 | 0.39 |
| emicizumab kxwh | 8 | 74,436 | 1,706 | 2,859,410 | 1,714 | 0.19 | 0.10 | 0.38 | 0.19 | 0.10 | 0.38 |
| cobimetinib | 6 | 74,438 | 1,318 | 2,859,798 | 1,324 | 0.19 | 0.09 | 0.41 | 0.19 | 0.09 | 0.41 |
| clopidogrel bisulfate | 25 | 74,419 | 5,412 | 2,855,704 | 5,437 | 0.18 | 0.12 | 0.27 | 0.18 | 0.12 | 0.27 |
| baricitinib | 13 | 74,431 | 2,883 | 2,858,233 | 2,896 | 0.18 | 0.11 | 0.31 | 0.18 | 0.11 | 0.31 |
| empagliflozin | 23 | 74,421 | 5,082 | 2,856,034 | 5,105 | 0.18 | 0.12 | 0.27 | 0.18 | 0.12 | 0.27 |
| doxorubicin | 38 | 74,406 | 8,456 | 2,852,660 | 8,494 | 0.17 | 0.13 | 0.24 | 0.17 | 0.13 | 0.24 |
| verapamil | 6 | 74,438 | 1,454 | 2,859,662 | 1,460 | 0.17 | 0.08 | 0.37 | 0.17 | 0.08 | 0.37 |
| midazolam | 10 | 74,434 | 2,362 | 2,858,754 | 2,372 | 0.17 | 0.09 | 0.31 | 0.17 | 0.09 | 0.31 |
| gilteritinib | 6 | 74,438 | 1,473 | 2,859,643 | 1,479 | 0.17 | 0.08 | 0.37 | 0.17 | 0.08 | 0.37 |
| fam trastuzumab deruxtecan nxki | 7 | 74,437 | 1,743 | 2,859,373 | 1,750 | 0.17 | 0.08 | 0.34 | 0.17 | 0.08 | 0.34 |
| tisagenlecleucel | 8 | 74,436 | 2,030 | 2,859,086 | 2,038 | 0.16 | 0.08 | 0.32 | 0.16 | 0.08 | 0.32 |
| valganciclovir | 5 | 74,439 | 1,326 | 2,859,790 | 1,331 | 0.16 | 0.07 | 0.37 | 0.16 | 0.07 | 0.37 |
| isatuximab | 6 | 74,438 | 1,579 | 2,859,537 | 1,585 | 0.16 | 0.07 | 0.34 | 0.16 | 0.07 | 0.34 |
| mycophenolic acid | 7 | 74,437 | 1,824 | 2,859,292 | 1,831 | 0.16 | 0.08 | 0.32 | 0.16 | 0.08 | 0.32 |
| cisplatin | 20 | 74,424 | 5,045 | 2,856,071 | 5,065 | 0.16 | 0.10 | 0.24 | 0.16 | 0.10 | 0.24 |
| aspirin | 34 | 74,410 | 8,535 | 2,852,581 | 8,569 | 0.15 | 0.11 | 0.22 | 0.16 | 0.11 | 0.22 |
| aspirin dl lysine | 9 | 74,435 | 2,358 | 2,858,758 | 2,367 | 0.15 | 0.08 | 0.29 | 0.15 | 0.08 | 0.29 |
| gemcitabine | 19 | 74,425 | 4,859 | 2,856,257 | 4,878 | 0.15 | 0.10 | 0.24 | 0.15 | 0.10 | 0.24 |
| cefazolin | 5 | 74,439 | 1,414 | 2,859,702 | 1,419 | 0.15 | 0.06 | 0.35 | 0.15 | 0.06 | 0.35 |
| edoxaban | 7 | 74,437 | 1,995 | 2,859,121 | 2,002 | 0.14 | 0.07 | 0.30 | 0.14 | 0.07 | 0.30 |
| etoposide | 21 | 74,423 | 5,817 | 2,855,299 | 5,838 | 0.14 | 0.09 | 0.22 | 0.14 | 0.09 | 0.22 |
| daptomycin | 6 | 74,438 | 1,790 | 2,859,326 | 1,796 | 0.14 | 0.06 | 0.30 | 0.14 | 0.06 | 0.30 |
| epinephrine | 5 | 74,439 | 1,529 | 2,859,587 | 1,534 | 0.14 | 0.06 | 0.32 | 0.14 | 0.06 | 0.32 |
| temozolomide | 11 | 74,433 | 3,222 | 2,857,894 | 3,233 | 0.14 | 0.08 | 0.24 | 0.14 | 0.08 | 0.24 |
| vincristine | 25 | 74,419 | 7,291 | 2,853,825 | 7,316 | 0.13 | 0.09 | 0.20 | 0.13 | 0.09 | 0.20 |
| iron sucrose | 6 | 74,438 | 1,875 | 2,859,241 | 1,881 | 0.13 | 0.06 | 0.29 | 0.13 | 0.06 | 0.29 |
| meropenem | 8 | 74,436 | 2,684 | 2,858,432 | 2,692 | 0.12 | 0.06 | 0.24 | 0.12 | 0.06 | 0.24 |
| oxaliplatin | 28 | 74,416 | 9,147 | 2,851,969 | 9,175 | 0.12 | 0.08 | 0.17 | 0.12 | 0.08 | 0.17 |
| alcohol | 7 | 74,437 | 2,436 | 2,858,680 | 2,443 | 0.12 | 0.06 | 0.24 | 0.12 | 0.06 | 0.24 |
| allopurinol | 9 | 74,435 | 3,168 | 2,857,948 | 3,177 | 0.12 | 0.06 | 0.22 | 0.12 | 0.06 | 0.22 |
| azacitidine | 13 | 74,431 | 4,593 | 2,856,523 | 4,606 | 0.11 | 0.07 | 0.19 | 0.11 | 0.07 | 0.19 |
| dabigatran etexilate | 11 | 74,433 | 4,378 | 2,856,738 | 4,389 | 0.10 | 0.06 | 0.18 | 0.10 | 0.06 | 0.18 |
| carfilzomib | 7 | 74,437 | 3,257 | 2,857,859 | 3,264 | 0.09 | 0.04 | 0.18 | 0.09 | 0.04 | 0.18 |
| vancomycin | 10 | 74,434 | 5,055 | 2,856,061 | 5,065 | 0.08 | 0.04 | 0.15 | 0.08 | 0.04 | 0.15 |
| bendamustine | 6 | 74,438 | 3,876 | 2,857,240 | 3,882 | 0.06 | 0.03 | 0.14 | 0.06 | 0.03 | 0.14 |
| cytarabine | 8 | 74,436 | 5,262 | 2,855,854 | 5,270 | 0.06 | 0.03 | 0.12 | 0.06 | 0.03 | 0.12 |
| enoxaparin | 6 | 74,438 | 4,841 | 2,856,275 | 4,847 | 0.05 | 0.02 | 0.11 | 0.05 | 0.02 | 0.11 |

Notes: ANY analysis includes primary-suspect (PS) and secondary-suspect (SS) drugs. Other definitions identical to PS analysis.

## Table S4a. Full sex heterogeneity results for insomnia disproportionality (PS analysis).

| **Parent systemic drug** | **a_F** | **b_F** | **c_F** | **d_F** | **a_M** | **b_M** | **c_M** | **d_M** | **ROR_F** | **ROR_M** | **logROR_diff** | **se_diff** | **z_diff** | **p_diff** | **p_diff_FDR** | **BD_p** | **BD_FDR** | **MH_OR** | **MH_p** | **sex_specific** |
| --- | --- | --- | --- | --- | --- | --- | --- | --- | --- | --- | --- | --- | --- | --- | --- | --- | --- | --- | --- | --- |
| oxybate | 49 | 42,898 | 109 | 1,396,374 | 11 | 23,949 | 58 | 1,151,640 | 14.71 | 9.45 | 0.443 | 0.365 | 1.211 | 0.226 | 0.226 | 0.201 | 0.201 | 13.16 | <0.001 | false |
| loratadine pseudoephedrine | 94 | 42,853 | 275 | 1,396,208 | 24 | 23,936 | 194 | 1,151,504 | 11.18 | 6.06 | 0.612 | 0.245 | 2.494 | 0.013 |  | 0.011 |  | 9.45 | <0.001 | false |
| trofinetide | 88 | 42,859 | 265 | 1,396,218 | 0 | 0 | 0 | 0 | 10.86 | 1.00 | 2.385 | 2.831 | 0.842 | 0.400 |  |  |  |  |  | true |
| niraparib | 364 | 42,583 | 1,134 | 1,395,349 | 0 | 0 | 0 | 0 | 10.53 | 1.00 | 2.354 | 2.829 | 0.832 | 0.405 |  |  |  |  |  | true |
| diphenhydramine naproxen | 17 | 42,930 | 57 | 1,396,426 | 0 | 0 | 0 | 0 | 9.90 | 1.00 | 2.293 | 2.842 | 0.807 | 0.420 |  |  |  |  |  | true |
| viloxazine | 15 | 42,932 | 52 | 1,396,431 | 12 | 23,948 | 49 | 1,151,649 | 9.60 | 12.14 | -0.235 | 0.429 | -0.547 | 0.584 |  | 0.601 |  | 10.32 | <0.001 | false |
| ropeginterferon alfa 2b njft | 23 | 42,924 | 91 | 1,396,392 | 8 | 23,952 | 72 | 1,151,626 | 8.36 | 5.64 | 0.394 | 0.430 | 0.915 | 0.360 |  | 0.325 |  | 7.21 | <0.001 | false |
| flibanserin | 99 | 42,848 | 422 | 1,396,061 | 0 | 0 | 0 | 0 | 7.67 | 1.00 | 2.038 | 2.831 | 0.720 | 0.472 |  |  |  |  |  | true |
| pseudoephedrine | 44 | 42,903 | 210 | 1,396,273 | 24 | 23,936 | 143 | 1,151,555 | 6.88 | 8.21 | -0.177 | 0.274 | -0.647 | 0.518 |  | 0.540 |  | 7.22 | <0.001 | false |
| suvorexant | 26 | 42,921 | 125 | 1,396,358 | 15 | 23,945 | 85 | 1,151,613 | 6.87 | 8.72 | -0.238 | 0.349 | -0.683 | 0.495 |  | 0.521 |  | 7.31 | <0.001 | false |
| eszopiclone | 21 | 42,926 | 107 | 1,396,376 | 10 | 23,950 | 68 | 1,151,630 | 6.51 | 7.37 | -0.125 | 0.407 | -0.307 | 0.759 |  | 0.805 |  | 6.59 | <0.001 | false |
| levoketoconazole | 8 | 42,939 | 44 | 1,396,439 | 0 | 0 | 0 | 0 | 6.21 | 1.00 | 1.826 | 2.853 | 0.640 | 0.522 |  |  |  |  |  | true |
| dupilumab | 869 | 42,078 | 4,652 | 1,391,831 | 605 | 23,355 | 4,244 | 1,147,454 | 6.18 | 7.01 | -0.126 | 0.058 | -2.179 | 0.029 |  | 0.030 |  | 6.49 | <0.001 | false |
| guaifenesin pseudoephedrine | 13 | 42,934 | 71 | 1,396,412 | 12 | 23,948 | 51 | 1,151,647 | 6.14 | 11.67 | -0.642 | 0.433 | -1.483 | 0.138 |  | 0.140 |  | 7.72 | <0.001 | false |
| pimavanserin | 241 | 42,706 | 1,354 | 1,395,129 | 327 | 23,633 | 1,524 | 1,150,174 | 5.82 | 10.45 | -0.585 | 0.093 | -6.288 | <0.001 |  | <0.001 |  | 7.82 | <0.001 | false |
| estrogens medroxyprogesterone | 31 | 42,916 | 188 | 1,396,295 | 0 | 0 | 0 | 0 | 5.44 | 1.00 | 1.693 | 2.835 | 0.597 | 0.550 |  |  |  |  |  | true |
| levothyroxine liothyronine | 39 | 42,908 | 238 | 1,396,245 | 0 | 0 | 0 | 0 | 5.39 | 1.00 | 1.684 | 2.834 | 0.594 | 0.552 |  |  |  |  |  | true |
| valbenazine | 98 | 42,849 | 598 | 1,395,885 | 48 | 23,912 | 511 | 1,151,187 | 5.36 | 4.56 | 0.161 | 0.186 | 0.866 | 0.386 |  | 0.373 |  | 5.04 | <0.001 | false |
| rucaparib | 113 | 42,834 | 732 | 1,395,751 | 0 | 0 | 0 | 0 | 5.05 | 1.00 | 1.619 | 2.830 | 0.572 | 0.567 |  |  |  |  |  | true |
| avapritinib | 130 | 42,817 | 861 | 1,395,622 | 92 | 23,868 | 903 | 1,150,795 | 4.94 | 4.94 | 0.000 | 0.144 | 0.002 | 0.999 |  | 0.990 |  | 4.92 | <0.001 | false |
| atovaquone proguanil | 6 | 42,941 | 44 | 1,396,439 | 0 | 0 | 0 | 0 | 4.75 | 1.00 | 1.558 | 2.859 | 0.545 | 0.586 |  |  |  |  |  | true |
| montelukast | 178 | 42,769 | 1,226 | 1,395,257 | 130 | 23,830 | 880 | 1,150,818 | 4.75 | 7.16 | -0.410 | 0.124 | -3.320 | <0.001 |  | <0.001 |  | 5.52 | <0.001 | false |
| pregabalin | 386 | 42,561 | 2,666 | 1,393,817 | 179 | 23,781 | 1,943 | 1,149,755 | 4.75 | 4.47 | 0.061 | 0.095 | 0.640 | 0.522 |  | 0.513 |  | 4.65 | <0.001 | false |
| lumateperone | 45 | 42,902 | 318 | 1,396,165 | 30 | 23,930 | 194 | 1,151,504 | 4.65 | 7.55 | -0.484 | 0.251 | -1.928 | 0.054 |  | 0.056 |  | 5.44 | <0.001 | false |
| abaloparatide | 171 | 42,776 | 1,209 | 1,395,274 | 9 | 23,951 | 216 | 1,151,482 | 4.63 | 2.11 | 0.785 | 0.341 | 2.299 | 0.022 |  | 0.014 |  | 4.33 | <0.001 | false |
| bupropion naltrexone | 87 | 42,860 | 639 | 1,395,844 | 12 | 23,948 | 117 | 1,151,581 | 4.46 | 5.12 | -0.138 | 0.319 | -0.433 | 0.665 |  | 0.742 |  | 4.49 | <0.001 | false |
| estrogens | 47 | 42,900 | 348 | 1,396,135 | 0 | 0 | 0 | 0 | 4.44 | 1.00 | 1.490 | 2.833 | 0.526 | 0.599 |  |  |  |  |  | true |
| carbidopa levodopa | 93 | 42,854 | 709 | 1,395,774 | 73 | 23,887 | 663 | 1,151,035 | 4.29 | 5.34 | -0.218 | 0.165 | -1.320 | 0.187 |  | 0.190 |  | 4.67 | <0.001 | false |
| vilazodone | 24 | 42,923 | 189 | 1,396,294 | 12 | 23,948 | 80 | 1,151,618 | 4.21 | 7.47 | -0.574 | 0.372 | -1.542 | 0.123 |  | 0.136 |  | 4.82 | <0.001 | false |
| elagolix | 77 | 42,870 | 609 | 1,395,874 | 0 | 0 | 0 | 0 | 4.14 | 1.00 | 1.421 | 2.831 | 0.502 | 0.616 |  |  |  |  |  | true |
| omalizumab | 504 | 42,443 | 4,047 | 1,392,436 | 239 | 23,721 | 2,150 | 1,149,548 | 4.09 | 5.40 | -0.277 | 0.083 | -3.332 | <0.001 |  | <0.001 |  | 4.43 | <0.001 | false |
| corticotropin | 88 | 42,859 | 708 | 1,395,775 | 46 | 23,914 | 402 | 1,151,296 | 4.07 | 5.56 | -0.313 | 0.192 | -1.631 | 0.103 |  | 0.108 |  | 4.46 | <0.001 | false |
| amphetamine aspartate amphetamine dextroamphetamine saccharate dextroamphetamine | 79 | 42,868 | 639 | 1,395,844 | 32 | 23,928 | 475 | 1,151,223 | 4.05 | 3.29 | 0.208 | 0.217 | 0.958 | 0.338 |  | 0.320 |  | 3.76 | <0.001 | false |
| levothyroxine | 291 | 42,656 | 2,362 | 1,394,121 | 42 | 23,918 | 679 | 1,151,019 | 4.03 | 3.01 | 0.293 | 0.170 | 1.720 | 0.085 |  | 0.076 |  | 3.85 | <0.001 | false |
| ofloxacin | 8 | 42,939 | 69 | 1,396,414 | 0 | 0 | 0 | 0 | 3.98 | 1.00 | 1.381 | 2.852 | 0.484 | 0.628 |  |  |  |  |  | true |
| phentermine topiramate | 56 | 42,891 | 466 | 1,396,017 | 17 | 23,943 | 85 | 1,151,613 | 3.94 | 9.84 | -0.915 | 0.298 | -3.072 | 0.002 |  | 0.002 |  | 4.54 | <0.001 | false |
| lurasidone | 136 | 42,811 | 1,153 | 1,395,330 | 90 | 23,870 | 688 | 1,151,010 | 3.86 | 6.34 | -0.497 | 0.144 | -3.447 | <0.001 |  | <0.001 |  | 4.56 | <0.001 | false |
| desvenlafaxine | 50 | 42,897 | 430 | 1,396,053 | 10 | 23,950 | 126 | 1,151,572 | 3.82 | 3.99 | -0.044 | 0.354 | -0.125 | 0.900 |  | 0.981 |  | 3.79 | <0.001 | false |
| naltrexone | 144 | 42,803 | 1,248 | 1,395,235 | 200 | 23,760 | 1,415 | 1,150,283 | 3.77 | 6.86 | -0.598 | 0.116 | -5.146 | <0.001 |  | <0.001 |  | 5.10 | <0.001 | false |
| amantadine | 31 | 42,916 | 273 | 1,396,210 | 23 | 23,937 | 233 | 1,151,465 | 3.75 | 4.84 | -0.256 | 0.287 | -0.893 | 0.372 |  | 0.384 |  | 4.08 | <0.001 | false |
| mifepristone | 23 | 42,924 | 208 | 1,396,275 | 5 | 23,955 | 55 | 1,151,643 | 3.67 | 4.76 | -0.262 | 0.497 | -0.527 | 0.598 |  | 0.705 |  | 3.72 | <0.001 | false |
| milnacipran | 8 | 42,939 | 75 | 1,396,408 | 0 | 0 | 0 | 0 | 3.66 | 1.00 | 1.298 | 2.851 | 0.455 | 0.649 |  |  |  |  |  | true |
| elacestrant | 55 | 42,892 | 494 | 1,395,989 | 0 | 0 | 0 | 0 | 3.65 | 1.00 | 1.295 | 2.832 | 0.457 | 0.647 |  |  |  |  |  | true |
| cetirizine pseudoephedrine | 7 | 42,940 | 67 | 1,396,416 | 0 | 0 | 0 | 0 | 3.61 | 1.00 | 1.285 | 2.855 | 0.450 | 0.653 |  |  |  |  |  | true |
| estradiol | 107 | 42,840 | 971 | 1,395,512 | 0 | 0 | 0 | 0 | 3.60 | 1.00 | 1.282 | 2.830 | 0.453 | 0.651 |  |  |  |  |  | true |
| dalfampridine | 170 | 42,777 | 1,554 | 1,394,929 | 55 | 23,905 | 945 | 1,150,753 | 3.58 | 2.83 | 0.236 | 0.160 | 1.472 | 0.141 |  | 0.132 |  | 3.34 | <0.001 | false |
| clonazepam | 96 | 42,851 | 882 | 1,395,601 | 46 | 23,914 | 698 | 1,151,000 | 3.56 | 3.20 | 0.106 | 0.186 | 0.569 | 0.569 |  | 0.551 |  | 3.41 | <0.001 | false |
| olanzapine samidorphan l | 21 | 42,926 | 198 | 1,396,285 | 19 | 23,941 | 160 | 1,151,538 | 3.52 | 5.84 | -0.506 | 0.330 | -1.532 | 0.126 |  | 0.128 |  | 4.25 | <0.001 | false |
| diphenhydramine ibuprofen | 5 | 42,942 | 51 | 1,396,432 | 0 | 0 | 0 | 0 | 3.47 | 1.00 | 1.245 | 2.864 | 0.435 | 0.664 |  |  |  |  |  | true |
| sofosbuvir velpatasvir | 122 | 42,825 | 1,164 | 1,395,319 | 133 | 23,827 | 1,377 | 1,150,321 | 3.43 | 4.68 | -0.311 | 0.132 | -2.366 | 0.018 |  | 0.018 |  | 3.97 | <0.001 | false |
| deutetrabenazine | 59 | 42,888 | 587 | 1,395,896 | 31 | 23,929 | 362 | 1,151,336 | 3.30 | 4.18 | -0.238 | 0.230 | -1.032 | 0.302 |  | 0.318 |  | 3.52 | <0.001 | false |
| cariprazine | 46 | 42,901 | 460 | 1,396,023 | 22 | 23,938 | 318 | 1,151,380 | 3.29 | 3.40 | -0.034 | 0.267 | -0.125 | 0.900 |  | 0.934 |  | 3.28 | <0.001 | false |
| fezolinetant | 14 | 42,933 | 144 | 1,396,339 | 0 | 0 | 0 | 0 | 3.26 | 1.00 | 1.183 | 2.842 | 0.416 | 0.677 |  |  |  |  |  | true |
| patisiran | 9 | 42,938 | 95 | 1,396,388 | 8 | 23,952 | 150 | 1,151,548 | 3.24 | 2.72 | 0.175 | 0.490 | 0.357 | 0.721 |  | 0.715 |  | 2.81 | <0.001 | false |
| bazedoxifene estrogens | 7 | 42,940 | 75 | 1,396,408 | 0 | 0 | 0 | 0 | 3.23 | 1.00 | 1.173 | 2.854 | 0.411 | 0.681 |  |  |  |  |  | true |
| ciprofloxacin | 175 | 42,772 | 1,777 | 1,394,706 | 119 | 23,841 | 1,634 | 1,150,064 | 3.22 | 3.53 | -0.091 | 0.124 | -0.737 | 0.461 |  | 0.468 |  | 3.33 | <0.001 | false |
| ocrelizumab | 414 | 42,533 | 4,229 | 1,392,254 | 126 | 23,834 | 2,500 | 1,149,198 | 3.21 | 2.44 | 0.274 | 0.105 | 2.609 | 0.009 |  | 0.008 |  | 2.98 | <0.001 | false |
| phentermine | 7 | 42,940 | 77 | 1,396,406 | 0 | 0 | 0 | 0 | 3.15 | 1.00 | 1.146 | 2.854 | 0.402 | 0.688 |  |  |  |  |  | true |
| estradiol progesterone | 9 | 42,938 | 98 | 1,396,385 | 0 | 0 | 0 | 0 | 3.14 | 1.00 | 1.143 | 2.849 | 0.401 | 0.688 |  |  |  |  |  | true |
| ciclesonide | 30 | 42,917 | 316 | 1,396,167 | 6 | 23,954 | 187 | 1,151,511 | 3.13 | 1.67 | 0.632 | 0.442 | 1.430 | 0.153 |  | 0.122 |  | 2.64 | <0.001 | false |
| thyroid porcine | 21 | 42,926 | 227 | 1,396,256 | 0 | 0 | 0 | 0 | 3.07 | 1.00 | 1.123 | 2.837 | 0.396 | 0.692 |  |  |  |  |  | true |
| roflumilast | 18 | 42,929 | 196 | 1,396,287 | 14 | 23,946 | 200 | 1,151,498 | 3.06 | 3.48 | -0.127 | 0.365 | -0.349 | 0.727 |  | 0.747 |  | 3.14 | <0.001 | false |
| teriflunomide | 233 | 42,714 | 2,523 | 1,393,960 | 48 | 23,912 | 1,247 | 1,150,451 | 3.02 | 1.87 | 0.479 | 0.162 | 2.961 | 0.003 |  | 0.003 |  | 2.72 | <0.001 | false |
| lenalidomide | 544 | 42,403 | 5,957 | 1,390,526 | 501 | 23,459 | 6,632 | 1,145,066 | 3.00 | 3.69 | -0.208 | 0.065 | -3.205 | 0.001 |  | 0.001 |  | 3.29 | <0.001 | false |
| varenicline | 67 | 42,880 | 735 | 1,395,748 | 34 | 23,926 | 492 | 1,151,206 | 2.99 | 3.37 | -0.121 | 0.217 | -0.555 | 0.579 |  | 0.602 |  | 3.08 | <0.001 | false |
| apremilast | 268 | 42,679 | 2,950 | 1,393,533 | 138 | 23,822 | 2,543 | 1,149,155 | 2.97 | 2.63 | 0.123 | 0.108 | 1.138 | 0.255 |  | 0.249 |  | 2.84 | <0.001 | false |
| nirmatrelvir ritonavir | 124 | 42,823 | 1,386 | 1,395,097 | 55 | 23,905 | 1,146 | 1,150,552 | 2.93 | 2.33 | 0.228 | 0.166 | 1.367 | 0.172 |  | 0.163 |  | 2.70 | <0.001 | false |
| elexacaftor ivacaftor tezacaftor | 135 | 42,812 | 1,541 | 1,394,942 | 96 | 23,864 | 1,309 | 1,150,389 | 2.86 | 3.55 | -0.215 | 0.139 | -1.553 | 0.120 |  | 0.123 |  | 3.10 | <0.001 | false |
| nebivolol | 20 | 42,927 | 233 | 1,396,250 | 0 | 0 | 0 | 0 | 2.86 | 1.00 | 1.049 | 2.838 | 0.370 | 0.712 |  |  |  |  |  | true |
| amphetamine aspartate amphetamine aspartate amphetamine dextroamphetamine saccharate dextroamphetamine | 7 | 42,940 | 85 | 1,396,398 | 0 | 0 | 0 | 0 | 2.85 | 1.00 | 1.048 | 2.854 | 0.367 | 0.713 |  |  |  |  |  | true |
| ofatumumab | 192 | 42,755 | 2,206 | 1,394,277 | 79 | 23,881 | 1,208 | 1,150,490 | 2.85 | 3.17 | -0.108 | 0.138 | -0.780 | 0.435 |  | 0.451 |  | 2.92 | <0.001 | false |
| dichlorphenamide | 24 | 42,923 | 281 | 1,396,202 | 15 | 23,945 | 251 | 1,151,447 | 2.83 | 2.96 | -0.046 | 0.336 | -0.136 | 0.892 |  | 0.921 |  | 2.81 | <0.001 | false |
| tirzepatide | 77 | 42,870 | 894 | 1,395,589 | 33 | 23,927 | 740 | 1,150,958 | 2.82 | 2.18 | 0.259 | 0.213 | 1.219 | 0.223 |  | 0.210 |  | 2.57 | <0.001 | false |
| sofosbuvir | 6 | 42,941 | 75 | 1,396,408 | 10 | 23,950 | 68 | 1,151,630 | 2.80 | 7.37 | -0.968 | 0.526 | -1.839 | 0.066 |  | 0.057 |  | 4.31 | <0.001 | false |
| treprostinil | 207 | 42,740 | 2,418 | 1,394,065 | 54 | 23,906 | 1,373 | 1,150,325 | 2.80 | 1.91 | 0.382 | 0.156 | 2.449 | 0.014 |  | 0.013 |  | 2.54 | <0.001 | false |
| sacubitril valsartan | 275 | 42,672 | 3,263 | 1,393,220 | 292 | 23,668 | 4,525 | 1,147,173 | 2.76 | 3.13 | -0.128 | 0.087 | -1.465 | 0.143 |  | 0.143 |  | 2.93 | <0.001 | false |
| ziprasidone | 7 | 42,940 | 88 | 1,396,395 | 0 | 0 | 0 | 0 | 2.76 | 1.00 | 1.014 | 2.854 | 0.355 | 0.722 |  |  |  |  |  | true |
| fexofenadine | 30 | 42,917 | 361 | 1,396,122 | 8 | 23,952 | 222 | 1,151,476 | 2.74 | 1.84 | 0.402 | 0.397 | 1.012 | 0.312 |  | 0.271 |  | 2.42 | <0.001 | false |
| levofloxacin | 110 | 42,837 | 1,354 | 1,395,129 | 59 | 23,901 | 1,184 | 1,150,514 | 2.66 | 2.42 | 0.094 | 0.166 | 0.568 | 0.570 |  | 0.556 |  | 2.55 | <0.001 | false |
| tofacitinib | 453 | 42,494 | 5,588 | 1,390,895 | 131 | 23,829 | 3,282 | 1,148,416 | 2.66 | 1.93 | 0.319 | 0.102 | 3.134 | 0.002 |  | 0.002 |  | 2.44 | <0.001 | false |
| duloxetine | 111 | 42,836 | 1,368 | 1,395,115 | 45 | 23,915 | 662 | 1,151,036 | 2.65 | 3.31 | -0.220 | 0.182 | -1.205 | 0.228 |  | 0.243 |  | 2.80 | <0.001 | false |
| atomoxetine | 11 | 42,936 | 141 | 1,396,342 | 11 | 23,949 | 202 | 1,151,496 | 2.64 | 2.73 | -0.033 | 0.431 | -0.075 | 0.940 |  | 0.943 |  | 2.58 | <0.001 | false |
| selexipag | 185 | 42,762 | 2,294 | 1,394,189 | 68 | 23,892 | 1,242 | 1,150,456 | 2.64 | 2.65 | -0.007 | 0.146 | -0.049 | 0.961 |  | 0.985 |  | 2.63 | <0.001 | false |
| hydroxyprogesterone caproate | 64 | 42,883 | 800 | 1,395,683 | 0 | 0 | 0 | 0 | 2.62 | 1.00 | 0.964 | 2.831 | 0.340 | 0.733 |  |  |  |  |  | true |
| nortriptyline | 7 | 42,940 | 95 | 1,396,388 | 0 | 0 | 0 | 0 | 2.55 | 1.00 | 0.938 | 2.854 | 0.329 | 0.742 |  |  |  |  |  | true |
| alemtuzumab | 119 | 42,828 | 1,537 | 1,394,946 | 32 | 23,928 | 748 | 1,150,950 | 2.53 | 2.09 | 0.192 | 0.203 | 0.948 | 0.343 |  | 0.319 |  | 2.41 | <0.001 | false |
| bupropion | 79 | 42,868 | 1,032 | 1,395,451 | 40 | 23,920 | 618 | 1,151,080 | 2.51 | 3.15 | -0.229 | 0.200 | -1.145 | 0.252 |  | 0.266 |  | 2.67 | <0.001 | false |
| hydrocodone | 11 | 42,936 | 150 | 1,396,333 | 16 | 23,944 | 167 | 1,151,531 | 2.48 | 4.74 | -0.645 | 0.400 | -1.612 | 0.107 |  | 0.101 |  | 3.34 | <0.001 | false |
| dextromethorphan guaifenesin | 30 | 42,917 | 405 | 1,396,078 | 14 | 23,946 | 269 | 1,151,429 | 2.45 | 2.59 | -0.056 | 0.329 | -0.170 | 0.865 |  | 0.910 |  | 2.44 | <0.001 | false |
| clarithromycin | 52 | 42,895 | 700 | 1,395,783 | 19 | 23,941 | 420 | 1,151,278 | 2.44 | 2.23 | 0.089 | 0.272 | 0.328 | 0.743 |  | 0.702 |  | 2.35 | <0.001 | false |
| triamcinolone acetonide | 11 | 42,936 | 157 | 1,396,326 | 0 | 0 | 0 | 0 | 2.37 | 1.00 | 0.865 | 2.845 | 0.304 | 0.761 |  |  |  |  |  | true |
| ledipasvir sofosbuvir | 34 | 42,913 | 473 | 1,396,010 | 36 | 23,924 | 480 | 1,151,218 | 2.37 | 3.66 | -0.433 | 0.246 | -1.759 | 0.079 |  | 0.078 |  | 2.86 | <0.001 | false |
| pyrimethamine | 5 | 42,942 | 75 | 1,396,408 | 5 | 23,955 | 95 | 1,151,603 | 2.37 | 2.77 | -0.156 | 0.622 | -0.251 | 0.802 |  | 0.812 |  | 2.34 | 0.007 | false |
| amphetamine dextroamphetamine | 5 | 42,942 | 76 | 1,396,407 | 0 | 0 | 0 | 0 | 2.34 | 1.00 | 0.849 | 2.863 | 0.297 | 0.767 |  |  |  |  |  | true |
| mitapivat | 5 | 42,942 | 76 | 1,396,407 | 0 | 0 | 0 | 0 | 2.34 | 1.00 | 0.849 | 2.863 | 0.297 | 0.767 |  |  |  |  |  | true |
| ombitasvir paritaprevir ritonavir | 6 | 42,941 | 90 | 1,396,393 | 0 | 0 | 0 | 0 | 2.34 | 1.00 | 0.848 | 2.857 | 0.297 | 0.767 |  |  |  |  |  | true |
| nicotine | 33 | 42,914 | 474 | 1,396,009 | 19 | 23,941 | 404 | 1,151,294 | 2.30 | 2.32 | -0.009 | 0.293 | -0.032 | 0.975 |  | 0.996 |  | 2.26 | <0.001 | false |
| water | 7 | 42,940 | 106 | 1,396,377 | 5 | 23,955 | 96 | 1,151,602 | 2.29 | 2.74 | -0.179 | 0.579 | -0.310 | 0.757 |  | 0.799 |  | 2.28 | 0.004 | false |
| nirogacestat | 6 | 42,941 | 93 | 1,396,390 | 0 | 0 | 0 | 0 | 2.26 | 1.00 | 0.816 | 2.857 | 0.285 | 0.775 |  |  |  |  |  | true |
| palbociclib | 353 | 42,594 | 5,114 | 1,391,369 | 7 | 23,953 | 498 | 1,151,200 | 2.26 | 0.72 | 1.139 | 0.372 | 3.060 | 0.002 |  | <0.001 |  | 2.16 | <0.001 | false |
| lasmiditan | 5 | 42,942 | 79 | 1,396,404 | 0 | 0 | 0 | 0 | 2.25 | 1.00 | 0.811 | 2.863 | 0.283 | 0.777 |  |  |  |  |  | true |
| cetirizine | 78 | 42,869 | 1,145 | 1,395,338 | 35 | 23,925 | 735 | 1,150,963 | 2.23 | 2.32 | -0.040 | 0.208 | -0.193 | 0.847 |  | 0.876 |  | 2.24 | <0.001 | false |
| oxybate | 17 | 42,930 | 255 | 1,396,228 | 6 | 23,954 | 192 | 1,151,506 | 2.23 | 1.62 | 0.317 | 0.469 | 0.675 | 0.500 |  | 0.447 |  | 1.94 | 0.002 | false |
| acetaminophen hydrocodone | 36 | 42,911 | 541 | 1,395,942 | 34 | 23,926 | 481 | 1,151,217 | 2.19 | 3.45 | -0.452 | 0.246 | -1.842 | 0.066 |  | 0.066 |  | 2.63 | <0.001 | false |
| galcanezumab gnlm | 64 | 42,883 | 961 | 1,395,522 | 15 | 23,945 | 326 | 1,151,372 | 2.18 | 2.28 | -0.045 | 0.290 | -0.154 | 0.878 |  | 0.944 |  | 2.18 | <0.001 | false |
| solriamfetol | 5 | 42,942 | 82 | 1,396,401 | 5 | 23,955 | 62 | 1,151,636 | 2.17 | 4.23 | -0.669 | 0.626 | -1.068 | 0.285 |  | 0.298 |  | 2.63 | 0.002 | false |
| pirfenidone | 90 | 42,857 | 1,360 | 1,395,123 | 126 | 23,834 | 1,866 | 1,149,832 | 2.17 | 3.27 | -0.412 | 0.142 | -2.893 | 0.004 |  | 0.004 |  | 2.69 | <0.001 | false |
| gabapentin | 94 | 42,853 | 1,429 | 1,395,054 | 38 | 23,922 | 1,016 | 1,150,682 | 2.15 | 1.82 | 0.167 | 0.196 | 0.851 | 0.395 |  | 0.375 |  | 2.03 | <0.001 | false |
| fingolimod | 338 | 42,609 | 5,164 | 1,391,319 | 90 | 23,870 | 2,348 | 1,149,350 | 2.14 | 1.86 | 0.143 | 0.121 | 1.178 | 0.239 |  | 0.227 |  | 2.07 | <0.001 | false |
| alendronate | 84 | 42,863 | 1,292 | 1,395,191 | 7 | 23,953 | 226 | 1,151,472 | 2.13 | 1.59 | 0.290 | 0.388 | 0.749 | 0.454 |  | 0.377 |  | 2.05 | <0.001 | false |
| leflunomide | 45 | 42,902 | 696 | 1,395,787 | 13 | 23,947 | 244 | 1,151,454 | 2.13 | 2.65 | -0.222 | 0.319 | -0.698 | 0.485 |  | 0.542 |  | 2.19 | <0.001 | false |
| sofosbuvir velpatasvir voxilaprevir | 6 | 42,941 | 99 | 1,396,384 | 5 | 23,955 | 179 | 1,151,519 | 2.12 | 1.47 | 0.366 | 0.593 | 0.618 | 0.537 |  | 0.533 |  | 1.62 | 0.112 | false |
| aripiprazole | 104 | 42,843 | 1,606 | 1,394,877 | 65 | 23,895 | 1,365 | 1,150,333 | 2.12 | 2.31 | -0.087 | 0.162 | -0.534 | 0.593 |  | 0.606 |  | 2.18 | <0.001 | false |
| lisdexamfetamine dimesylate | 33 | 42,914 | 519 | 1,395,964 | 22 | 23,938 | 433 | 1,151,265 | 2.10 | 2.50 | -0.174 | 0.280 | -0.620 | 0.535 |  | 0.555 |  | 2.20 | <0.001 | false |
| pexidartinib | 13 | 42,934 | 209 | 1,396,274 | 7 | 23,953 | 144 | 1,151,554 | 2.10 | 2.50 | -0.175 | 0.468 | -0.373 | 0.709 |  | 0.764 |  | 2.12 | <0.001 | false |
| propranolol | 37 | 42,910 | 594 | 1,395,889 | 13 | 23,947 | 348 | 1,151,350 | 2.05 | 1.86 | 0.097 | 0.325 | 0.299 | 0.765 |  | 0.714 |  | 1.96 | <0.001 | false |
| loratadine | 61 | 42,886 | 981 | 1,395,502 | 24 | 23,936 | 816 | 1,150,882 | 2.04 | 1.44 | 0.346 | 0.244 | 1.419 | 0.156 |  | 0.143 |  | 1.80 | <0.001 | false |
| bupropion dextromethorphan | 11 | 42,936 | 184 | 1,396,299 | 11 | 23,949 | 118 | 1,151,580 | 2.03 | 4.67 | -0.834 | 0.433 | -1.924 | 0.054 |  | 0.053 |  | 2.72 | <0.001 | false |
| venlafaxine | 107 | 42,840 | 1,728 | 1,394,755 | 34 | 23,926 | 909 | 1,150,789 | 2.02 | 1.82 | 0.104 | 0.200 | 0.521 | 0.603 |  | 0.571 |  | 1.96 | <0.001 | false |
| chlorhexidine | 7 | 42,940 | 121 | 1,396,362 | 0 | 0 | 0 | 0 | 2.01 | 1.00 | 0.697 | 2.853 | 0.244 | 0.807 |  |  |  |  |  | true |
| oseltamivir | 31 | 42,916 | 511 | 1,395,972 | 20 | 23,940 | 409 | 1,151,289 | 2.00 | 2.41 | -0.184 | 0.292 | -0.631 | 0.528 |  | 0.551 |  | 2.11 | <0.001 | false |
| erenumab aooe | 86 | 42,861 | 1,417 | 1,395,066 | 5 | 23,955 | 403 | 1,151,295 | 1.99 | 0.66 | 1.109 | 0.443 | 2.501 | 0.012 |  | 0.006 |  | 1.75 | <0.001 | false |
| sucralfate | 6 | 42,941 | 106 | 1,396,377 | 0 | 0 | 0 | 0 | 1.98 | 1.00 | 0.685 | 2.857 | 0.240 | 0.810 |  |  |  |  |  | true |
| sertraline | 137 | 42,810 | 2,293 | 1,394,190 | 60 | 23,900 | 1,320 | 1,150,378 | 1.95 | 2.21 | -0.122 | 0.158 | -0.769 | 0.442 |  | 0.460 |  | 2.01 | <0.001 | false |
| methylphenidate | 57 | 42,890 | 968 | 1,395,515 | 104 | 23,856 | 1,231 | 1,150,467 | 1.93 | 4.09 | -0.751 | 0.170 | -4.418 | <0.001 |  | <0.001 |  | 2.92 | <0.001 | false |
| guaifenesin | 15 | 42,932 | 261 | 1,396,222 | 10 | 23,950 | 188 | 1,151,510 | 1.93 | 2.68 | -0.329 | 0.411 | -0.800 | 0.424 |  | 0.453 |  | 2.10 | <0.001 | false |
| budesonide | 103 | 42,844 | 1,750 | 1,394,733 | 76 | 23,884 | 1,494 | 1,150,204 | 1.92 | 2.47 | -0.247 | 0.155 | -1.596 | 0.111 |  | 0.113 |  | 2.11 | <0.001 | false |
| voclosporin | 45 | 42,902 | 773 | 1,395,710 | 11 | 23,949 | 294 | 1,151,404 | 1.91 | 1.88 | 0.019 | 0.337 | 0.057 | 0.955 |  | 0.881 |  | 1.87 | <0.001 | false |
| ripretinib | 26 | 42,921 | 450 | 1,396,033 | 28 | 23,932 | 551 | 1,151,147 | 1.91 | 2.49 | -0.262 | 0.277 | -0.944 | 0.345 |  | 0.346 |  | 2.14 | <0.001 | false |
| escitalopram | 76 | 42,871 | 1,316 | 1,395,167 | 57 | 23,903 | 781 | 1,150,917 | 1.89 | 3.54 | -0.628 | 0.180 | -3.478 | <0.001 |  | <0.001 |  | 2.35 | <0.001 | false |
| paliperidone | 24 | 42,923 | 422 | 1,396,061 | 18 | 23,942 | 584 | 1,151,114 | 1.89 | 1.52 | 0.215 | 0.315 | 0.682 | 0.495 |  | 0.485 |  | 1.67 | 0.001 | false |
| oxycodone | 146 | 42,801 | 2,541 | 1,393,942 | 201 | 23,759 | 3,295 | 1,148,403 | 1.88 | 2.96 | -0.454 | 0.112 | -4.050 | <0.001 |  | <0.001 |  | 2.38 | <0.001 | false |
| citalopram | 53 | 42,894 | 930 | 1,395,553 | 29 | 23,931 | 512 | 1,151,186 | 1.87 | 2.77 | -0.392 | 0.236 | -1.662 | 0.097 |  | 0.103 |  | 2.09 | <0.001 | false |
| macitentan | 252 | 42,695 | 4,410 | 1,392,073 | 61 | 23,899 | 2,325 | 1,149,373 | 1.87 | 1.27 | 0.384 | 0.145 | 2.651 | 0.008 |  | 0.007 |  | 1.70 | <0.001 | false |
| hydromorphone | 27 | 42,920 | 482 | 1,396,001 | 23 | 23,937 | 437 | 1,151,261 | 1.85 | 2.58 | -0.332 | 0.289 | -1.150 | 0.250 |  | 0.257 |  | 2.09 | <0.001 | false |
| progesterone | 12 | 42,935 | 219 | 1,396,264 | 0 | 0 | 0 | 0 | 1.85 | 1.00 | 0.616 | 2.843 | 0.217 | 0.828 |  |  |  |  |  | true |
| rosuvastatin | 82 | 42,865 | 1,461 | 1,395,022 | 75 | 23,885 | 1,549 | 1,150,149 | 1.84 | 2.35 | -0.245 | 0.164 | -1.495 | 0.135 |  | 0.136 |  | 2.04 | <0.001 | false |
| bremelanotide | 5 | 42,942 | 97 | 1,396,386 | 0 | 0 | 0 | 0 | 1.83 | 1.00 | 0.607 | 2.862 | 0.212 | 0.832 |  |  |  |  |  | true |
| prucalopride | 5 | 42,942 | 98 | 1,396,385 | 0 | 0 | 0 | 0 | 1.82 | 1.00 | 0.596 | 2.862 | 0.208 | 0.835 |  |  |  |  |  | true |
| bismuth subcitrate metronidazole tetracycline | 5 | 42,942 | 99 | 1,396,384 | 0 | 0 | 0 | 0 | 1.80 | 1.00 | 0.586 | 2.862 | 0.205 | 0.838 |  |  |  |  |  | true |
| inotersen | 9 | 42,938 | 172 | 1,396,311 | 0 | 0 | 0 | 0 | 1.79 | 1.00 | 0.583 | 2.848 | 0.205 | 0.838 |  |  |  |  |  | true |
| brexpiprazole | 20 | 42,927 | 373 | 1,396,110 | 10 | 23,950 | 246 | 1,151,452 | 1.79 | 2.05 | -0.137 | 0.388 | -0.354 | 0.724 |  | 0.773 |  | 1.81 | 0.001 | false |
| pramipexole | 5 | 42,942 | 100 | 1,396,383 | 0 | 0 | 0 | 0 | 1.78 | 1.00 | 0.576 | 2.862 | 0.201 | 0.840 |  |  |  |  |  | true |
| belimumab | 88 | 42,859 | 1,620 | 1,394,863 | 0 | 0 | 0 | 0 | 1.78 | 1.00 | 0.575 | 2.831 | 0.203 | 0.839 |  |  |  |  |  | true |
| vonoprazan | 11 | 42,936 | 210 | 1,396,273 | 0 | 0 | 0 | 0 | 1.78 | 1.00 | 0.575 | 2.845 | 0.202 | 0.840 |  |  |  |  |  | true |
| anastrozole | 61 | 42,886 | 1,134 | 1,395,349 | 0 | 0 | 0 | 0 | 1.76 | 1.00 | 0.567 | 2.831 | 0.200 | 0.841 |  |  |  |  |  | true |
| octreotide | 116 | 42,831 | 2,174 | 1,394,309 | 82 | 23,878 | 1,991 | 1,149,707 | 1.74 | 1.99 | -0.134 | 0.147 | -0.910 | 0.363 |  | 0.370 |  | 1.83 | <0.001 | false |
| semaglutide | 171 | 42,776 | 3,203 | 1,393,280 | 96 | 23,864 | 2,796 | 1,148,902 | 1.74 | 1.66 | 0.048 | 0.130 | 0.372 | 0.710 |  | 0.698 |  | 1.71 | <0.001 | false |
| adalimumab | 810 | 42,137 | 15,262 | 1,381,221 | 455 | 23,505 | 11,918 | 1,139,780 | 1.74 | 1.85 | -0.063 | 0.060 | -1.037 | 0.300 |  | 0.303 |  | 1.78 | <0.001 | false |
| atogepant | 26 | 42,921 | 496 | 1,395,987 | 11 | 23,949 | 116 | 1,151,582 | 1.74 | 4.75 | -1.006 | 0.368 | -2.734 | 0.006 |  | 0.006 |  | 2.10 | <0.001 | false |
| liothyronine | 7 | 42,940 | 143 | 1,396,340 | 0 | 0 | 0 | 0 | 1.70 | 1.00 | 0.530 | 2.853 | 0.186 | 0.853 |  |  |  |  |  | true |
| vortioxetine | 43 | 42,904 | 835 | 1,395,648 | 27 | 23,933 | 463 | 1,151,235 | 1.69 | 2.85 | -0.522 | 0.251 | -2.083 | 0.037 |  | 0.039 |  | 1.99 | <0.001 | false |
| nintedanib | 96 | 42,851 | 1,869 | 1,394,614 | 115 | 23,845 | 2,510 | 1,149,188 | 1.68 | 2.22 | -0.277 | 0.141 | -1.961 | 0.050 |  | 0.049 |  | 1.93 | <0.001 | false |
| fenfluramine | 16 | 42,931 | 321 | 1,396,162 | 24 | 23,936 | 295 | 1,151,403 | 1.67 | 3.99 | -0.871 | 0.329 | -2.651 | 0.008 |  | 0.006 |  | 2.50 | <0.001 | false |
| famotidine | 16 | 42,931 | 327 | 1,396,156 | 0 | 0 | 0 | 0 | 1.64 | 1.00 | 0.494 | 2.840 | 0.174 | 0.862 |  |  |  |  |  | true |
| budesonide formoterol dihydrate | 94 | 42,853 | 1,877 | 1,394,606 | 58 | 23,902 | 1,347 | 1,150,351 | 1.64 | 2.09 | -0.243 | 0.170 | -1.429 | 0.153 |  | 0.159 |  | 1.78 | <0.001 | false |
| tazemetostat | 7 | 42,940 | 149 | 1,396,334 | 0 | 0 | 0 | 0 | 1.63 | 1.00 | 0.489 | 2.853 | 0.172 | 0.864 |  |  |  |  |  | true |
| amitriptyline | 15 | 42,932 | 309 | 1,396,174 | 0 | 0 | 0 | 0 | 1.63 | 1.00 | 0.488 | 2.840 | 0.172 | 0.864 |  |  |  |  |  | true |
| inclisiran | 30 | 42,917 | 609 | 1,395,874 | 20 | 23,940 | 580 | 1,151,118 | 1.63 | 1.70 | -0.042 | 0.292 | -0.145 | 0.884 |  | 0.907 |  | 1.62 | <0.001 | false |
| adalimumab afzb | 20 | 42,927 | 411 | 1,396,072 | 11 | 23,949 | 297 | 1,151,401 | 1.62 | 1.86 | -0.137 | 0.376 | -0.365 | 0.715 |  | 0.758 |  | 1.65 | 0.006 | false |
| diroximel | 18 | 42,929 | 374 | 1,396,109 | 0 | 0 | 0 | 0 | 1.61 | 1.00 | 0.474 | 2.838 | 0.167 | 0.867 |  |  |  |  |  | true |
| brivaracetam | 22 | 42,925 | 458 | 1,396,025 | 11 | 23,949 | 393 | 1,151,305 | 1.60 | 1.40 | 0.128 | 0.369 | 0.346 | 0.730 |  | 0.691 |  | 1.48 | 0.026 | false |
| secukinumab | 295 | 42,652 | 6,079 | 1,390,404 | 211 | 23,749 | 5,636 | 1,146,062 | 1.58 | 1.81 | -0.133 | 0.092 | -1.446 | 0.148 |  | 0.150 |  | 1.67 | <0.001 | false |
| acyclovir | 28 | 42,919 | 587 | 1,395,896 | 0 | 0 | 0 | 0 | 1.58 | 1.00 | 0.456 | 2.835 | 0.161 | 0.872 |  |  |  |  |  | true |
| cannabidiol | 25 | 42,922 | 529 | 1,395,954 | 21 | 23,939 | 618 | 1,151,080 | 1.57 | 1.67 | -0.065 | 0.299 | -0.218 | 0.828 |  | 0.840 |  | 1.58 | 0.002 | false |
| fluticasone | 6 | 42,941 | 135 | 1,396,348 | 0 | 0 | 0 | 0 | 1.56 | 1.00 | 0.445 | 2.857 | 0.156 | 0.876 |  |  |  |  |  | true |
| fremanezumab vfrm | 38 | 42,909 | 803 | 1,395,680 | 0 | 0 | 0 | 0 | 1.56 | 1.00 | 0.444 | 2.833 | 0.157 | 0.876 |  |  |  |  |  | true |
| ethinyl estradiol norethindrone | 14 | 42,933 | 303 | 1,396,180 | 0 | 0 | 0 | 0 | 1.55 | 1.00 | 0.441 | 2.841 | 0.155 | 0.877 |  |  |  |  |  | true |
| guanfacine | 8 | 42,939 | 178 | 1,396,305 | 6 | 23,954 | 238 | 1,151,460 | 1.55 | 1.31 | 0.167 | 0.530 | 0.315 | 0.753 |  | 0.733 |  | 1.34 | 0.277 | false |
| cyclobenzaprine | 6 | 42,941 | 136 | 1,396,347 | 0 | 0 | 0 | 0 | 1.55 | 1.00 | 0.437 | 2.857 | 0.153 | 0.878 |  |  |  |  |  | true |
| rimegepant | 24 | 42,923 | 517 | 1,395,966 | 8 | 23,952 | 179 | 1,151,519 | 1.54 | 2.28 | -0.391 | 0.407 | -0.960 | 0.337 |  | 0.396 |  | 1.63 | 0.006 | false |
| glycopyrrolate | 15 | 42,932 | 327 | 1,396,156 | 6 | 23,954 | 237 | 1,151,461 | 1.54 | 1.32 | 0.157 | 0.475 | 0.330 | 0.741 |  | 0.678 |  | 1.40 | 0.128 | false |
| erenumab | 7 | 42,940 | 158 | 1,396,325 | 0 | 0 | 0 | 0 | 1.54 | 1.00 | 0.431 | 2.853 | 0.151 | 0.880 |  |  |  |  |  | true |
| fluticasone furoate umeclidinium bromide vilanterol trifenatate | 31 | 42,916 | 667 | 1,395,816 | 16 | 23,944 | 546 | 1,151,152 | 1.53 | 1.45 | 0.056 | 0.309 | 0.180 | 0.857 |  | 0.822 |  | 1.47 | 0.009 | false |
| epoprostenol | 56 | 42,891 | 1,199 | 1,395,284 | 11 | 23,949 | 524 | 1,151,174 | 1.53 | 1.05 | 0.374 | 0.328 | 1.142 | 0.254 |  | 0.217 |  | 1.40 | 0.006 | false |
| tizanidine | 7 | 42,940 | 159 | 1,396,324 | 0 | 0 | 0 | 0 | 1.53 | 1.00 | 0.425 | 2.853 | 0.149 | 0.882 |  |  |  |  |  | true |
| paroxetine | 36 | 42,911 | 779 | 1,395,704 | 20 | 23,940 | 493 | 1,151,205 | 1.52 | 2.00 | -0.271 | 0.282 | -0.962 | 0.336 |  | 0.359 |  | 1.64 | <0.001 | false |
| asfotase alfa | 13 | 42,934 | 289 | 1,396,194 | 0 | 0 | 0 | 0 | 1.52 | 1.00 | 0.416 | 2.842 | 0.146 | 0.884 |  |  |  |  |  | true |
| buprenorphine | 44 | 42,903 | 959 | 1,395,524 | 50 | 23,910 | 873 | 1,150,825 | 1.51 | 2.78 | -0.612 | 0.211 | -2.901 | 0.004 |  | 0.003 |  | 1.98 | <0.001 | false |
| icatibant | 41 | 42,906 | 905 | 1,395,578 | 10 | 23,950 | 344 | 1,151,354 | 1.49 | 1.47 | 0.017 | 0.351 | 0.049 | 0.961 |  | 0.882 |  | 1.46 | 0.008 | false |
| budesonide formoterol | 19 | 42,928 | 425 | 1,396,058 | 7 | 23,953 | 296 | 1,151,402 | 1.49 | 1.22 | 0.204 | 0.436 | 0.466 | 0.641 |  | 0.583 |  | 1.35 | 0.130 | false |
| dimethyl | 121 | 42,826 | 2,694 | 1,393,789 | 30 | 23,930 | 1,325 | 1,150,373 | 1.47 | 1.11 | 0.283 | 0.205 | 1.376 | 0.169 |  | 0.153 |  | 1.37 | <0.001 | false |
| esketamine | 14 | 42,933 | 321 | 1,396,162 | 7 | 23,953 | 238 | 1,151,460 | 1.47 | 1.51 | -0.030 | 0.458 | -0.066 | 0.947 |  | 0.995 |  | 1.42 | 0.115 | false |
| amifampridine | 25 | 42,922 | 568 | 1,395,915 | 17 | 23,943 | 390 | 1,151,308 | 1.46 | 2.15 | -0.390 | 0.317 | -1.229 | 0.219 |  | 0.233 |  | 1.64 | 0.001 | false |
| caplacizumab yhdp | 19 | 42,928 | 435 | 1,396,048 | 0 | 0 | 0 | 0 | 1.46 | 1.00 | 0.376 | 2.838 | 0.132 | 0.895 |  |  |  |  |  | true |
| quetiapine | 128 | 42,819 | 2,886 | 1,393,597 | 93 | 23,867 | 1,974 | 1,149,724 | 1.45 | 2.28 | -0.454 | 0.139 | -3.259 | 0.001 |  | 0.001 |  | 1.71 | <0.001 | false |
| dextroamphetamine | 5 | 42,942 | 123 | 1,396,360 | 0 | 0 | 0 | 0 | 1.45 | 1.00 | 0.370 | 2.862 | 0.129 | 0.897 |  |  |  |  |  | true |
| moxifloxacin | 23 | 42,924 | 529 | 1,395,954 | 20 | 23,940 | 551 | 1,151,147 | 1.44 | 1.79 | -0.214 | 0.308 | -0.693 | 0.488 |  | 0.499 |  | 1.55 | 0.004 | false |
| paliperidone palmitate | 76 | 42,871 | 1,731 | 1,394,752 | 143 | 23,817 | 2,556 | 1,149,142 | 1.44 | 2.71 | -0.633 | 0.145 | -4.364 | <0.001 |  | <0.001 |  | 2.07 | <0.001 | false |
| telotristat ethyl | 8 | 42,939 | 192 | 1,396,291 | 8 | 23,952 | 152 | 1,151,546 | 1.44 | 2.68 | -0.624 | 0.497 | -1.255 | 0.209 |  | 0.215 |  | 1.77 | 0.024 | false |
| fluticasone propionate | 27 | 42,920 | 623 | 1,395,860 | 23 | 23,937 | 509 | 1,151,189 | 1.43 | 2.22 | -0.436 | 0.287 | -1.517 | 0.129 |  | 0.133 |  | 1.68 | <0.001 | false |
| pomalidomide | 127 | 42,820 | 2,901 | 1,393,582 | 145 | 23,815 | 3,140 | 1,148,558 | 1.43 | 2.23 | -0.446 | 0.124 | -3.590 | <0.001 |  | <0.001 |  | 1.77 | <0.001 | false |
| ribociclib | 208 | 42,739 | 4,772 | 1,391,711 | 0 | 0 | 0 | 0 | 1.42 | 1.00 | 0.352 | 2.829 | 0.125 | 0.901 |  |  |  |  |  | true |
| eslicarbazepine | 12 | 42,935 | 287 | 1,396,196 | 10 | 23,950 | 203 | 1,151,495 | 1.41 | 2.48 | -0.562 | 0.429 | -1.312 | 0.190 |  | 0.200 |  | 1.69 | 0.015 | false |
| dexlansoprazole | 11 | 42,936 | 265 | 1,396,218 | 0 | 0 | 0 | 0 | 1.41 | 1.00 | 0.343 | 2.844 | 0.120 | 0.904 |  |  |  |  |  | true |
| anifrolumab fnia | 9 | 42,938 | 219 | 1,396,264 | 0 | 0 | 0 | 0 | 1.41 | 1.00 | 0.342 | 2.848 | 0.120 | 0.904 |  |  |  |  |  | true |
| aclidinium bromide | 11 | 42,936 | 266 | 1,396,217 | 5 | 23,955 | 151 | 1,151,547 | 1.40 | 1.75 | -0.218 | 0.528 | -0.413 | 0.680 |  | 0.758 |  | 1.41 | 0.172 | false |
| tocilizumab | 197 | 42,750 | 4,631 | 1,391,852 | 40 | 23,920 | 2,526 | 1,149,172 | 1.39 | 0.77 | 0.589 | 0.174 | 3.378 | <0.001 |  | <0.001 |  | 1.22 | 0.003 | false |
| prednisone | 49 | 42,898 | 1,161 | 1,395,322 | 28 | 23,932 | 1,186 | 1,150,512 | 1.39 | 1.15 | 0.183 | 0.239 | 0.765 | 0.444 |  | 0.429 |  | 1.28 | 0.036 | false |
| vibegron | 8 | 42,939 | 199 | 1,396,284 | 0 | 0 | 0 | 0 | 1.39 | 1.00 | 0.326 | 2.850 | 0.114 | 0.909 |  |  |  |  |  | true |
| mirtazapine | 46 | 42,901 | 1,109 | 1,395,374 | 30 | 23,930 | 761 | 1,150,937 | 1.36 | 1.93 | -0.346 | 0.238 | -1.454 | 0.146 |  | 0.153 |  | 1.52 | <0.001 | false |
| norethindrone | 5 | 42,942 | 131 | 1,396,352 | 0 | 0 | 0 | 0 | 1.36 | 1.00 | 0.307 | 2.862 | 0.107 | 0.914 |  |  |  |  |  | true |
| ambrisentan | 66 | 42,881 | 1,594 | 1,394,889 | 24 | 23,936 | 1,042 | 1,150,656 | 1.36 | 1.13 | 0.183 | 0.240 | 0.763 | 0.445 |  | 0.417 |  | 1.27 | 0.024 | false |
| raltegravir | 9 | 42,938 | 228 | 1,396,255 | 0 | 0 | 0 | 0 | 1.35 | 1.00 | 0.302 | 2.848 | 0.106 | 0.916 |  |  |  |  |  | true |
| sparsentan | 12 | 42,935 | 301 | 1,396,182 | 18 | 23,942 | 333 | 1,151,365 | 1.35 | 2.67 | -0.682 | 0.375 | -1.821 | 0.069 |  | 0.063 |  | 1.86 | <0.001 | false |
| fentanyl | 49 | 42,898 | 1,202 | 1,395,281 | 39 | 23,921 | 940 | 1,150,758 | 1.34 | 2.02 | -0.411 | 0.218 | -1.888 | 0.059 |  | 0.060 |  | 1.56 | <0.001 | false |
| tamoxifen | 21 | 42,926 | 522 | 1,395,961 | 0 | 0 | 0 | 0 | 1.34 | 1.00 | 0.291 | 2.837 | 0.103 | 0.918 |  |  |  |  |  | true |
| rituximab pvvr | 27 | 42,920 | 670 | 1,395,813 | 8 | 23,952 | 478 | 1,151,220 | 1.33 | 0.85 | 0.446 | 0.397 | 1.124 | 0.261 |  | 0.226 |  | 1.14 | 0.431 | false |
| tramadol | 6 | 42,941 | 158 | 1,396,325 | 0 | 0 | 0 | 0 | 1.33 | 1.00 | 0.288 | 2.857 | 0.101 | 0.920 |  |  |  |  |  | true |
| umeclidinium bromide vilanterol trifenatate | 15 | 42,932 | 378 | 1,396,105 | 0 | 0 | 0 | 0 | 1.33 | 1.00 | 0.286 | 2.840 | 0.101 | 0.920 |  |  |  |  |  | true |
| acetaminophen oxycodone | 20 | 42,927 | 501 | 1,395,982 | 18 | 23,942 | 361 | 1,151,337 | 1.33 | 2.46 | -0.616 | 0.328 | -1.877 | 0.061 |  | 0.061 |  | 1.66 | 0.002 | false |
| etanercept szzs | 30 | 42,917 | 746 | 1,395,737 | 7 | 23,953 | 412 | 1,151,286 | 1.33 | 0.87 | 0.419 | 0.412 | 1.016 | 0.309 |  | 0.263 |  | 1.17 | 0.339 | false |
| hydrochlorothiazide losartan | 10 | 42,937 | 262 | 1,396,221 | 0 | 0 | 0 | 0 | 1.30 | 1.00 | 0.263 | 2.846 | 0.092 | 0.926 |  |  |  |  |  | true |
| triptorelin | 9 | 42,938 | 237 | 1,396,246 | 0 | 0 | 0 | 0 | 1.30 | 1.00 | 0.263 | 2.848 | 0.092 | 0.926 |  |  |  |  |  | true |
| rituximab | 192 | 42,755 | 4,818 | 1,391,665 | 43 | 23,917 | 3,568 | 1,148,130 | 1.30 | 0.59 | 0.798 | 0.170 | 4.710 | <0.001 |  | <0.001 |  | 1.06 | 0.417 | false |
| glecaprevir pibrentasvir | 49 | 42,898 | 1,241 | 1,395,242 | 41 | 23,919 | 1,397 | 1,150,301 | 1.30 | 1.43 | -0.096 | 0.214 | -0.450 | 0.653 |  | 0.661 |  | 1.34 | 0.006 | false |
| levonorgestrel | 19 | 42,928 | 489 | 1,395,994 | 0 | 0 | 0 | 0 | 1.30 | 1.00 | 0.259 | 2.838 | 0.091 | 0.927 |  |  |  |  |  | true |
| carvedilol | 9 | 42,938 | 239 | 1,396,244 | 0 | 0 | 0 | 0 | 1.29 | 1.00 | 0.255 | 2.848 | 0.089 | 0.929 |  |  |  |  |  | true |
| budesonide formoterol glycopyrronium | 16 | 42,931 | 419 | 1,396,064 | 8 | 23,952 | 402 | 1,151,296 | 1.28 | 1.02 | 0.231 | 0.428 | 0.540 | 0.589 |  | 0.551 |  | 1.13 | 0.559 | false |
| albuterol | 38 | 42,909 | 979 | 1,395,504 | 13 | 23,947 | 664 | 1,151,034 | 1.28 | 0.98 | 0.269 | 0.320 | 0.841 | 0.401 |  | 0.365 |  | 1.16 | 0.295 | false |
| methylprednisolone | 101 | 42,846 | 2,599 | 1,393,884 | 47 | 23,913 | 2,213 | 1,149,485 | 1.27 | 1.03 | 0.208 | 0.178 | 1.167 | 0.243 |  | 0.232 |  | 1.17 | 0.054 | false |
| maribavir | 5 | 42,942 | 142 | 1,396,341 | 6 | 23,954 | 154 | 1,151,544 | 1.26 | 2.02 | -0.477 | 0.591 | -0.807 | 0.419 |  | 0.421 |  | 1.45 | 0.219 | false |
| morphine | 30 | 42,917 | 791 | 1,395,692 | 28 | 23,932 | 785 | 1,150,913 | 1.25 | 1.74 | -0.331 | 0.265 | -1.247 | 0.212 |  | 0.216 |  | 1.43 | 0.007 | false |
| alprazolam | 53 | 42,894 | 1,392 | 1,395,091 | 26 | 23,934 | 927 | 1,150,771 | 1.25 | 1.37 | -0.095 | 0.241 | -0.392 | 0.695 |  | 0.726 |  | 1.27 | 0.035 | false |
| adalimumab adaz | 35 | 42,912 | 925 | 1,395,558 | 24 | 23,936 | 680 | 1,151,018 | 1.25 | 1.73 | -0.328 | 0.268 | -1.225 | 0.221 |  | 0.232 |  | 1.39 | 0.013 | false |
| rifaximin | 40 | 42,907 | 1,058 | 1,395,425 | 26 | 23,934 | 927 | 1,150,771 | 1.24 | 1.37 | -0.099 | 0.254 | -0.389 | 0.697 |  | 0.718 |  | 1.27 | 0.052 | false |
| clofazimine | 9 | 42,938 | 248 | 1,396,235 | 8 | 23,952 | 199 | 1,151,499 | 1.24 | 2.05 | -0.499 | 0.482 | -1.037 | 0.300 |  | 0.315 |  | 1.45 | 0.133 | false |
| vandetanib | 7 | 42,940 | 197 | 1,396,286 | 0 | 0 | 0 | 0 | 1.23 | 1.00 | 0.211 | 2.853 | 0.074 | 0.941 |  |  |  |  |  | true |
| eltrombopag olamine | 60 | 42,887 | 1,614 | 1,394,869 | 30 | 23,930 | 1,399 | 1,150,299 | 1.22 | 1.05 | 0.151 | 0.225 | 0.672 | 0.502 |  | 0.481 |  | 1.14 | 0.213 | false |
| migalastat | 6 | 42,941 | 173 | 1,396,310 | 0 | 0 | 0 | 0 | 1.22 | 1.00 | 0.197 | 2.857 | 0.069 | 0.945 |  |  |  |  |  | true |
| alendronic acid | 11 | 42,936 | 308 | 1,396,175 | 0 | 0 | 0 | 0 | 1.21 | 1.00 | 0.192 | 2.844 | 0.068 | 0.946 |  |  |  |  |  | true |
| obeticholic acid | 37 | 42,910 | 1,006 | 1,395,477 | 6 | 23,954 | 216 | 1,151,482 | 1.21 | 1.44 | -0.175 | 0.431 | -0.405 | 0.685 |  | 0.805 |  | 1.21 | 0.211 | false |
| ozanimod | 48 | 42,899 | 1,302 | 1,395,181 | 39 | 23,921 | 910 | 1,150,788 | 1.21 | 2.09 | -0.544 | 0.219 | -2.488 | 0.013 |  | 0.013 |  | 1.48 | <0.001 | false |
| estradiol norethindrone | 21 | 42,926 | 579 | 1,395,904 | 0 | 0 | 0 | 0 | 1.21 | 1.00 | 0.188 | 2.837 | 0.066 | 0.947 |  |  |  |  |  | true |
| lecanemab irmb | 11 | 42,936 | 310 | 1,396,173 | 0 | 0 | 0 | 0 | 1.20 | 1.00 | 0.186 | 2.844 | 0.065 | 0.948 |  |  |  |  |  | true |
| anakinra | 39 | 42,908 | 1,067 | 1,395,416 | 10 | 23,950 | 745 | 1,150,953 | 1.20 | 0.68 | 0.575 | 0.351 | 1.641 | 0.101 |  | 0.083 |  | 1.01 | 0.929 | false |
| rasagiline | 7 | 42,940 | 203 | 1,396,280 | 0 | 0 | 0 | 0 | 1.20 | 1.00 | 0.181 | 2.853 | 0.063 | 0.949 |  |  |  |  |  | true |
| rizatriptan benzoate | 5 | 42,942 | 149 | 1,396,334 | 0 | 0 | 0 | 0 | 1.20 | 1.00 | 0.179 | 2.862 | 0.063 | 0.950 |  |  |  |  |  | true |
| onabotulinumtoxina | 44 | 42,903 | 1,210 | 1,395,273 | 7 | 23,953 | 533 | 1,151,165 | 1.20 | 0.68 | 0.571 | 0.398 | 1.433 | 0.152 |  | 0.120 |  | 1.05 | 0.707 | false |
| tapentadol | 9 | 42,938 | 258 | 1,396,225 | 5 | 23,955 | 198 | 1,151,500 | 1.20 | 1.33 | -0.108 | 0.544 | -0.199 | 0.842 |  | 0.905 |  | 1.16 | 0.581 | false |
| metformin vildagliptin | 12 | 42,935 | 340 | 1,396,143 | 14 | 23,946 | 394 | 1,151,304 | 1.19 | 1.77 | -0.392 | 0.393 | -0.998 | 0.318 |  | 0.317 |  | 1.40 | 0.093 | false |
| bamlanivimab | 12 | 42,935 | 341 | 1,396,142 | 7 | 23,953 | 300 | 1,151,398 | 1.19 | 1.20 | -0.008 | 0.469 | -0.017 | 0.987 |  | 0.967 |  | 1.14 | 0.584 | false |
| fluticasone propionate salmeterol xinafoate | 30 | 42,917 | 836 | 1,395,647 | 7 | 23,953 | 589 | 1,151,109 | 1.19 | 0.61 | 0.662 | 0.411 | 1.611 | 0.107 |  | 0.085 |  | 0.97 | 0.871 | false |
| telmisartan | 8 | 42,939 | 233 | 1,396,250 | 0 | 0 | 0 | 0 | 1.18 | 1.00 | 0.169 | 2.850 | 0.059 | 0.953 |  |  |  |  |  | true |
| methotrexate | 135 | 42,812 | 3,743 | 1,392,740 | 46 | 23,914 | 2,557 | 1,149,141 | 1.18 | 0.87 | 0.298 | 0.172 | 1.734 | 0.083 |  | 0.076 |  | 1.07 | 0.339 | false |
| amikacin | 33 | 42,914 | 930 | 1,395,553 | 15 | 23,945 | 644 | 1,151,054 | 1.17 | 1.16 | 0.013 | 0.312 | 0.041 | 0.968 |  | 0.924 |  | 1.14 | 0.362 | false |
| topiramate | 52 | 42,895 | 1,460 | 1,395,023 | 17 | 23,943 | 557 | 1,151,141 | 1.17 | 1.51 | -0.255 | 0.281 | -0.910 | 0.363 |  | 0.404 |  | 1.22 | 0.101 | false |
| divalproex | 17 | 42,930 | 487 | 1,395,996 | 14 | 23,946 | 484 | 1,151,214 | 1.17 | 1.44 | -0.209 | 0.361 | -0.579 | 0.562 |  | 0.579 |  | 1.24 | 0.240 | false |
| siponimod | 56 | 42,891 | 1,574 | 1,394,909 | 23 | 23,937 | 840 | 1,150,858 | 1.17 | 1.34 | -0.141 | 0.249 | -0.567 | 0.571 |  | 0.608 |  | 1.20 | 0.111 | false |
| fluticasone furoate vilanterol trifenatate | 23 | 42,924 | 657 | 1,395,826 | 14 | 23,946 | 441 | 1,151,257 | 1.16 | 1.58 | -0.306 | 0.340 | -0.902 | 0.367 |  | 0.393 |  | 1.26 | 0.165 | false |
| abatacept | 233 | 42,714 | 6,537 | 1,389,946 | 25 | 23,935 | 2,740 | 1,148,958 | 1.16 | 0.45 | 0.956 | 0.210 | 4.555 | <0.001 |  | <0.001 |  | 1.00 | 0.983 | false |
| voriconazole | 23 | 42,924 | 659 | 1,395,824 | 32 | 23,928 | 1,038 | 1,150,660 | 1.16 | 1.50 | -0.261 | 0.275 | -0.949 | 0.343 |  | 0.335 |  | 1.32 | 0.045 | false |
| cedazuridine decitabine | 6 | 42,941 | 182 | 1,396,301 | 0 | 0 | 0 | 0 | 1.16 | 1.00 | 0.147 | 2.856 | 0.051 | 0.959 |  |  |  |  |  | true |
| teprotumumab trbw | 14 | 42,933 | 408 | 1,396,075 | 12 | 23,948 | 197 | 1,151,501 | 1.15 | 3.04 | -0.969 | 0.396 | -2.450 | 0.014 |  | 0.013 |  | 1.57 | 0.024 | false |
| lanreotide | 45 | 42,902 | 1,283 | 1,395,200 | 23 | 23,937 | 1,102 | 1,150,596 | 1.15 | 1.02 | 0.118 | 0.257 | 0.458 | 0.647 |  | 0.621 |  | 1.09 | 0.485 | false |
| epirubicin | 30 | 42,917 | 866 | 1,395,617 | 0 | 0 | 0 | 0 | 1.14 | 1.00 | 0.135 | 2.834 | 0.048 | 0.962 |  |  |  |  |  | true |
| lorazepam | 32 | 42,915 | 925 | 1,395,558 | 18 | 23,942 | 587 | 1,151,111 | 1.14 | 1.51 | -0.282 | 0.296 | -0.952 | 0.341 |  | 0.365 |  | 1.23 | 0.148 | false |
| trazodone | 7 | 42,940 | 214 | 1,396,269 | 0 | 0 | 0 | 0 | 1.14 | 1.00 | 0.128 | 2.853 | 0.045 | 0.964 |  |  |  |  |  | true |
| ponatinib | 31 | 42,916 | 904 | 1,395,579 | 33 | 23,927 | 1,077 | 1,150,621 | 1.13 | 1.50 | -0.278 | 0.252 | -1.101 | 0.271 |  | 0.272 |  | 1.28 | 0.055 | false |
| nilotinib | 56 | 42,891 | 1,622 | 1,394,861 | 34 | 23,926 | 1,852 | 1,149,846 | 1.13 | 0.89 | 0.235 | 0.219 | 1.075 | 0.282 |  | 0.273 |  | 1.02 | 0.872 | false |
| leuprolide | 40 | 42,907 | 1,163 | 1,395,320 | 230 | 23,730 | 5,310 | 1,146,388 | 1.13 | 2.10 | -0.616 | 0.174 | -3.551 | <0.001 |  | <0.001 |  | 1.85 | <0.001 | false |
| fluoxetine | 38 | 42,909 | 1,111 | 1,395,372 | 21 | 23,939 | 492 | 1,151,206 | 1.13 | 2.10 | -0.623 | 0.275 | -2.266 | 0.023 |  | 0.025 |  | 1.33 | 0.031 | false |
| eptinezumab jjmr | 38 | 42,909 | 1,113 | 1,395,370 | 11 | 23,949 | 407 | 1,151,291 | 1.12 | 1.36 | -0.188 | 0.341 | -0.551 | 0.582 |  | 0.651 |  | 1.15 | 0.341 | false |
| evolocumab | 48 | 42,899 | 1,405 | 1,395,078 | 28 | 23,932 | 1,509 | 1,150,189 | 1.12 | 0.91 | 0.212 | 0.239 | 0.889 | 0.374 |  | 0.360 |  | 1.02 | 0.877 | false |
| candesartan cilexetil | 16 | 42,931 | 480 | 1,396,003 | 0 | 0 | 0 | 0 | 1.12 | 1.00 | 0.110 | 2.839 | 0.039 | 0.969 |  |  |  |  |  | true |
| levocetirizine dihydrochloride | 6 | 42,941 | 189 | 1,396,294 | 0 | 0 | 0 | 0 | 1.12 | 1.00 | 0.109 | 2.856 | 0.038 | 0.970 |  |  |  |  |  | true |
| icosapent ethyl | 11 | 42,936 | 335 | 1,396,148 | 5 | 23,955 | 435 | 1,151,263 | 1.11 | 0.61 | 0.608 | 0.524 | 1.161 | 0.246 |  | 0.218 |  | 0.83 | 0.448 | false |
| abemaciclib | 90 | 42,857 | 2,644 | 1,393,839 | 0 | 0 | 0 | 0 | 1.11 | 1.00 | 0.107 | 2.830 | 0.038 | 0.970 |  |  |  |  |  | true |
| infliximab | 230 | 42,717 | 6,750 | 1,389,733 | 178 | 23,782 | 6,085 | 1,145,613 | 1.11 | 1.41 | -0.241 | 0.102 | -2.368 | 0.018 |  | 0.018 |  | 1.22 | <0.001 | false |
| ibrutinib | 126 | 42,821 | 3,725 | 1,392,758 | 161 | 23,799 | 5,698 | 1,146,000 | 1.10 | 1.36 | -0.212 | 0.121 | -1.751 | 0.080 |  | 0.079 |  | 1.23 | <0.001 | false |
| ixazomib | 70 | 42,877 | 2,079 | 1,394,404 | 64 | 23,896 | 2,265 | 1,149,433 | 1.10 | 1.37 | -0.217 | 0.175 | -1.238 | 0.216 |  | 0.218 |  | 1.21 | 0.031 | false |
| exemestane | 25 | 42,922 | 753 | 1,395,730 | 0 | 0 | 0 | 0 | 1.10 | 1.00 | 0.096 | 2.836 | 0.034 | 0.973 |  |  |  |  |  | true |
| risedronate | 11 | 42,936 | 340 | 1,396,143 | 0 | 0 | 0 | 0 | 1.10 | 1.00 | 0.094 | 2.844 | 0.033 | 0.974 |  |  |  |  |  | true |
| simvastatin | 22 | 42,925 | 667 | 1,395,816 | 21 | 23,939 | 716 | 1,150,982 | 1.10 | 1.44 | -0.275 | 0.306 | -0.897 | 0.370 |  | 0.376 |  | 1.22 | 0.207 | false |
| umeclidinium bromide | 7 | 42,940 | 223 | 1,396,260 | 0 | 0 | 0 | 0 | 1.09 | 1.00 | 0.087 | 2.853 | 0.031 | 0.976 |  |  |  |  |  | true |
| pacritinib | 6 | 42,941 | 194 | 1,396,289 | 11 | 23,949 | 204 | 1,151,494 | 1.09 | 2.70 | -0.912 | 0.501 | -1.820 | 0.069 |  | 0.058 |  | 1.67 | 0.036 | false |
| sarilumab | 62 | 42,885 | 1,899 | 1,394,584 | 17 | 23,943 | 831 | 1,150,867 | 1.07 | 1.01 | 0.056 | 0.274 | 0.205 | 0.838 |  | 0.782 |  | 1.04 | 0.708 | false |
| mepolizumab | 47 | 42,900 | 1,449 | 1,395,034 | 24 | 23,936 | 848 | 1,150,850 | 1.07 | 1.39 | -0.265 | 0.253 | -1.047 | 0.295 |  | 0.316 |  | 1.14 | 0.270 | false |
| metoclopramide | 6 | 42,941 | 199 | 1,396,284 | 0 | 0 | 0 | 0 | 1.06 | 1.00 | 0.058 | 2.856 | 0.020 | 0.984 |  |  |  |  |  | true |
| human immunoglobulin g | 181 | 42,766 | 5,595 | 1,390,888 | 78 | 23,882 | 4,554 | 1,147,144 | 1.05 | 0.83 | 0.242 | 0.137 | 1.772 | 0.076 |  | 0.072 |  | 0.97 | 0.631 | false |
| aripiprazole lauroxil | 16 | 42,931 | 509 | 1,395,974 | 22 | 23,938 | 464 | 1,151,234 | 1.05 | 2.33 | -0.794 | 0.331 | -2.402 | 0.016 |  | 0.014 |  | 1.50 | 0.013 | false |
| fruquintinib | 7 | 42,940 | 232 | 1,396,251 | 8 | 23,952 | 254 | 1,151,444 | 1.05 | 1.61 | -0.426 | 0.509 | -0.836 | 0.403 |  | 0.406 |  | 1.21 | 0.468 | false |
| formoterol glycopyrrolate | 7 | 42,940 | 237 | 1,396,246 | 0 | 0 | 0 | 0 | 1.03 | 1.00 | 0.026 | 2.853 | 0.009 | 0.993 |  |  |  |  |  | true |
| adalimumab isopropyl alcohol | 12 | 42,935 | 396 | 1,396,087 | 0 | 0 | 0 | 0 | 1.03 | 1.00 | 0.025 | 2.843 | 0.009 | 0.993 |  |  |  |  |  | true |
| bempedoic acid | 5 | 42,942 | 174 | 1,396,309 | 0 | 0 | 0 | 0 | 1.02 | 1.00 | 0.025 | 2.861 | 0.009 | 0.993 |  |  |  |  |  | true |
| alirocumab | 31 | 42,916 | 1,003 | 1,395,480 | 27 | 23,933 | 905 | 1,150,793 | 1.02 | 1.46 | -0.358 | 0.265 | -1.351 | 0.177 |  | 0.181 |  | 1.17 | 0.242 | false |
| bosutinib | 23 | 42,924 | 752 | 1,395,731 | 15 | 23,945 | 780 | 1,150,918 | 1.02 | 0.95 | 0.062 | 0.331 | 0.187 | 0.852 |  | 0.827 |  | 0.97 | 0.830 | false |
| drospirenone | 7 | 42,940 | 240 | 1,396,243 | 0 | 0 | 0 | 0 | 1.01 | 1.00 | 0.014 | 2.853 | 0.005 | 0.996 |  |  |  |  |  | true |
| sulfamethoxazole trimethoprim | 16 | 42,931 | 529 | 1,395,954 | 6 | 23,954 | 634 | 1,151,064 | 1.01 | 0.49 | 0.722 | 0.467 | 1.546 | 0.122 |  | 0.102 |  | 0.75 | 0.172 | false |
| mirabegron | 21 | 42,926 | 690 | 1,395,793 | 17 | 23,943 | 493 | 1,151,205 | 1.01 | 1.70 | -0.521 | 0.327 | -1.592 | 0.111 |  | 0.116 |  | 1.21 | 0.249 | false |
| interferon beta 1a | 110 | 42,837 | 3,550 | 1,392,933 | 41 | 23,919 | 1,390 | 1,150,308 | 1.01 | 1.44 | -0.349 | 0.185 | -1.889 | 0.059 |  | 0.064 |  | 1.09 | 0.275 | false |
| amlodipine besylate hydrochlorothiazide valsartan | 9 | 42,938 | 305 | 1,396,178 | 0 | 0 | 0 | 0 | 1.01 | 1.00 | 0.011 | 2.848 | 0.004 | 0.997 |  |  |  |  |  | true |
| losartan | 33 | 42,914 | 1,078 | 1,395,405 | 21 | 23,939 | 817 | 1,150,881 | 1.01 | 1.26 | -0.225 | 0.280 | -0.801 | 0.423 |  | 0.444 |  | 1.08 | 0.589 | false |
| vedolizumab | 207 | 42,740 | 6,681 | 1,389,802 | 127 | 23,833 | 5,988 | 1,145,710 | 1.01 | 1.02 | -0.013 | 0.114 | -0.117 | 0.907 |  | 0.917 |  | 1.01 | 0.829 | false |
| beclomethasone dipropionate | 5 | 42,942 | 179 | 1,396,304 | 0 | 0 | 0 | 0 | 1.00 | 1.00 | -0.004 | 2.861 | -0.001 | 0.999 |  |  |  |  |  | true |
| pravastatin | 6 | 42,941 | 213 | 1,396,270 | 5 | 23,955 | 212 | 1,151,486 | 0.99 | 1.24 | -0.229 | 0.587 | -0.389 | 0.697 |  | 0.728 |  | 1.00 | 0.989 | false |
| avacopan | 15 | 42,932 | 509 | 1,395,974 | 12 | 23,948 | 431 | 1,151,267 | 0.99 | 1.39 | -0.342 | 0.386 | -0.887 | 0.375 |  | 0.393 |  | 1.10 | 0.633 | false |
| cholestyramine | 6 | 42,941 | 214 | 1,396,269 | 0 | 0 | 0 | 0 | 0.99 | 1.00 | -0.015 | 2.856 | -0.005 | 0.996 |  |  |  |  |  | true |
| risankizumab rzaa | 115 | 42,832 | 3,815 | 1,392,668 | 95 | 23,865 | 3,869 | 1,147,829 | 0.98 | 1.19 | -0.187 | 0.140 | -1.334 | 0.182 |  | 0.185 |  | 1.06 | 0.389 | false |
| fesoterodine | 8 | 42,939 | 283 | 1,396,200 | 0 | 0 | 0 | 0 | 0.97 | 1.00 | -0.025 | 2.850 | -0.009 | 0.993 |  |  |  |  |  | true |
| hydrochlorothiazide valsartan | 13 | 42,934 | 451 | 1,396,032 | 0 | 0 | 0 | 0 | 0.97 | 1.00 | -0.028 | 2.842 | -0.010 | 0.992 |  |  |  |  |  | true |
| bosentan | 50 | 42,897 | 1,689 | 1,394,794 | 12 | 23,948 | 733 | 1,150,965 | 0.97 | 0.82 | 0.171 | 0.319 | 0.536 | 0.592 |  | 0.534 |  | 0.92 | 0.530 | false |
| ipratropium bromide | 7 | 42,940 | 253 | 1,396,230 | 0 | 0 | 0 | 0 | 0.96 | 1.00 | -0.039 | 2.853 | -0.014 | 0.989 |  |  |  |  |  | true |
| elagolix estradiol norethindrone | 6 | 42,941 | 220 | 1,396,263 | 0 | 0 | 0 | 0 | 0.96 | 1.00 | -0.042 | 2.856 | -0.015 | 0.988 |  |  |  |  |  | true |
| mavacamten | 14 | 42,933 | 493 | 1,395,990 | 9 | 23,951 | 337 | 1,151,361 | 0.96 | 1.35 | -0.348 | 0.423 | -0.822 | 0.411 |  | 0.445 |  | 1.04 | 0.860 | false |
| upadacitinib | 177 | 42,770 | 6,049 | 1,390,434 | 75 | 23,885 | 3,835 | 1,147,863 | 0.95 | 0.95 | 0.008 | 0.139 | 0.060 | 0.952 |  | 0.931 |  | 0.95 | 0.402 | false |
| alpha 1 proteinase inhibitor human | 25 | 42,922 | 875 | 1,395,608 | 21 | 23,939 | 677 | 1,151,021 | 0.95 | 1.53 | -0.477 | 0.297 | -1.604 | 0.109 |  | 0.112 |  | 1.12 | 0.436 | false |
| baclofen | 8 | 42,939 | 292 | 1,396,191 | 0 | 0 | 0 | 0 | 0.94 | 1.00 | -0.057 | 2.850 | -0.020 | 0.984 |  |  |  |  |  | true |
| teduglutide water | 5 | 42,942 | 189 | 1,396,294 | 0 | 0 | 0 | 0 | 0.94 | 1.00 | -0.058 | 2.861 | -0.020 | 0.984 |  |  |  |  |  | true |
| vigabatrin | 18 | 42,929 | 642 | 1,395,841 | 24 | 23,936 | 630 | 1,151,068 | 0.94 | 1.87 | -0.691 | 0.313 | -2.207 | 0.027 |  | 0.025 |  | 1.28 | 0.113 | false |
| sildenafil | 39 | 42,908 | 1,375 | 1,395,108 | 24 | 23,936 | 1,753 | 1,149,945 | 0.93 | 0.67 | 0.330 | 0.260 | 1.270 | 0.204 |  | 0.195 |  | 0.80 | 0.078 | false |
| idelalisib | 7 | 42,940 | 261 | 1,396,222 | 0 | 0 | 0 | 0 | 0.93 | 1.00 | -0.070 | 2.853 | -0.024 | 0.980 |  |  |  |  |  | true |
| letrozole | 54 | 42,893 | 1,915 | 1,394,568 | 0 | 0 | 0 | 0 | 0.93 | 1.00 | -0.078 | 2.832 | -0.028 | 0.978 |  |  |  |  |  | true |
| fluticasone furoate | 6 | 42,941 | 228 | 1,396,255 | 6 | 23,954 | 146 | 1,151,552 | 0.92 | 2.13 | -0.836 | 0.565 | -1.479 | 0.139 |  | 0.143 |  | 1.20 | 0.540 | false |
| velaglucerase alfa | 10 | 42,937 | 372 | 1,396,111 | 0 | 0 | 0 | 0 | 0.92 | 1.00 | -0.087 | 2.846 | -0.031 | 0.976 |  |  |  |  |  | true |
| lisinopril | 28 | 42,919 | 1,018 | 1,395,465 | 20 | 23,940 | 1,199 | 1,150,499 | 0.91 | 0.82 | 0.102 | 0.293 | 0.349 | 0.727 |  | 0.712 |  | 0.85 | 0.276 | false |
| pancrelipase amylase pancrelipase lipase pancrelipase protease | 39 | 42,908 | 1,412 | 1,395,071 | 15 | 23,945 | 1,178 | 1,150,520 | 0.91 | 0.63 | 0.364 | 0.302 | 1.203 | 0.229 |  | 0.208 |  | 0.79 | 0.093 | false |
| mometasone furoate | 8 | 42,939 | 304 | 1,396,179 | 0 | 0 | 0 | 0 | 0.91 | 1.00 | -0.097 | 2.850 | -0.034 | 0.973 |  |  |  |  |  | true |
| dulaglutide | 41 | 42,906 | 1,491 | 1,394,992 | 29 | 23,931 | 1,397 | 1,150,301 | 0.90 | 1.01 | -0.115 | 0.244 | -0.471 | 0.638 |  | 0.655 |  | 0.93 | 0.576 | false |
| polyethylene glycol 3350 | 31 | 42,916 | 1,132 | 1,395,351 | 30 | 23,930 | 917 | 1,150,781 | 0.90 | 1.60 | -0.570 | 0.258 | -2.208 | 0.027 |  | 0.027 |  | 1.13 | 0.334 | false |
| acalabrutinib | 17 | 42,930 | 631 | 1,395,852 | 26 | 23,934 | 856 | 1,150,842 | 0.90 | 1.49 | -0.501 | 0.313 | -1.604 | 0.109 |  | 0.103 |  | 1.16 | 0.345 | false |
| apixaban | 110 | 42,837 | 4,003 | 1,392,480 | 82 | 23,878 | 4,145 | 1,147,553 | 0.90 | 0.96 | -0.064 | 0.147 | -0.434 | 0.665 |  | 0.673 |  | 0.92 | 0.236 | false |
| terbinafine | 8 | 42,939 | 311 | 1,396,172 | 0 | 0 | 0 | 0 | 0.89 | 1.00 | -0.120 | 2.850 | -0.042 | 0.967 |  |  |  |  |  | true |
| metoprolol | 28 | 42,919 | 1,046 | 1,395,437 | 13 | 23,947 | 870 | 1,150,828 | 0.89 | 0.75 | 0.172 | 0.334 | 0.517 | 0.606 |  | 0.570 |  | 0.82 | 0.195 | false |
| azelastine | 6 | 42,941 | 239 | 1,396,244 | 5 | 23,955 | 170 | 1,151,528 | 0.88 | 1.55 | -0.564 | 0.588 | -0.959 | 0.338 |  | 0.365 |  | 1.01 | 0.970 | false |
| satralizumab mwge | 9 | 42,938 | 350 | 1,396,133 | 0 | 0 | 0 | 0 | 0.88 | 1.00 | -0.126 | 2.847 | -0.044 | 0.965 |  |  |  |  |  | true |
| ibandronate | 15 | 42,932 | 572 | 1,395,911 | 0 | 0 | 0 | 0 | 0.88 | 1.00 | -0.127 | 2.840 | -0.045 | 0.964 |  |  |  |  |  | true |
| voxelotor | 38 | 42,909 | 1,428 | 1,395,055 | 25 | 23,935 | 1,221 | 1,150,477 | 0.88 | 1.00 | -0.136 | 0.258 | -0.525 | 0.600 |  | 0.621 |  | 0.91 | 0.454 | false |
| prednisolone | 31 | 42,916 | 1,173 | 1,395,310 | 27 | 23,933 | 1,013 | 1,150,685 | 0.87 | 1.30 | -0.402 | 0.265 | -1.519 | 0.129 |  | 0.132 |  | 1.02 | 0.906 | false |
| cladribine | 37 | 42,910 | 1,407 | 1,395,076 | 12 | 23,948 | 540 | 1,151,158 | 0.87 | 1.11 | -0.249 | 0.331 | -0.755 | 0.450 |  | 0.507 |  | 0.90 | 0.463 | false |
| atorvastatin | 67 | 42,880 | 2,540 | 1,393,943 | 31 | 23,929 | 2,243 | 1,149,455 | 0.86 | 0.67 | 0.247 | 0.218 | 1.135 | 0.256 |  | 0.242 |  | 0.78 | 0.017 | false |
| brigatinib | 16 | 42,931 | 623 | 1,395,860 | 6 | 23,954 | 544 | 1,151,154 | 0.86 | 0.57 | 0.405 | 0.467 | 0.868 | 0.385 |  | 0.342 |  | 0.72 | 0.127 | false |
| estradiol levonorgestrel | 7 | 42,940 | 285 | 1,396,198 | 0 | 0 | 0 | 0 | 0.85 | 1.00 | -0.158 | 2.853 | -0.055 | 0.956 |  |  |  |  |  | true |
| omadacycline | 6 | 42,941 | 247 | 1,396,236 | 0 | 0 | 0 | 0 | 0.85 | 1.00 | -0.158 | 2.856 | -0.055 | 0.956 |  |  |  |  |  | true |
| certolizumab pegol | 163 | 42,784 | 6,254 | 1,390,229 | 21 | 23,939 | 2,761 | 1,148,937 | 0.85 | 0.37 | 0.821 | 0.231 | 3.560 | <0.001 |  | <0.001 |  | 0.74 | <0.001 | false |
| bictegravir emtricitabine tenofovir alafenamide | 13 | 42,934 | 518 | 1,395,965 | 42 | 23,918 | 1,201 | 1,150,497 | 0.85 | 1.70 | -0.698 | 0.317 | -2.203 | 0.028 |  | 0.022 |  | 1.35 | 0.029 | false |
| vismodegib | 15 | 42,932 | 599 | 1,395,884 | 21 | 23,939 | 800 | 1,150,898 | 0.84 | 1.29 | -0.429 | 0.338 | -1.271 | 0.204 |  | 0.197 |  | 1.03 | 0.871 | false |
| etanercept | 128 | 42,819 | 5,008 | 1,391,475 | 47 | 23,913 | 2,832 | 1,148,866 | 0.83 | 0.81 | 0.034 | 0.172 | 0.200 | 0.842 |  | 0.813 |  | 0.82 | 0.010 | false |
| tramadol | 29 | 42,918 | 1,153 | 1,395,330 | 27 | 23,933 | 930 | 1,150,768 | 0.83 | 1.42 | -0.536 | 0.269 | -1.994 | 0.046 |  | 0.046 |  | 1.02 | 0.866 | false |
| bimekizumab | 6 | 42,941 | 254 | 1,396,229 | 0 | 0 | 0 | 0 | 0.83 | 1.00 | -0.186 | 2.856 | -0.065 | 0.948 |  |  |  |  |  | true |
| somatropin | 70 | 42,877 | 2,775 | 1,393,708 | 51 | 23,909 | 3,225 | 1,148,473 | 0.83 | 0.77 | 0.074 | 0.185 | 0.398 | 0.691 |  | 0.681 |  | 0.79 | 0.012 | false |
| natalizumab | 47 | 42,900 | 1,870 | 1,394,613 | 10 | 23,950 | 708 | 1,150,990 | 0.83 | 0.71 | 0.148 | 0.344 | 0.429 | 0.668 |  | 0.597 |  | 0.79 | 0.076 | false |
| alpelisib | 40 | 42,907 | 1,600 | 1,394,883 | 0 | 0 | 0 | 0 | 0.82 | 1.00 | -0.195 | 2.833 | -0.069 | 0.945 |  |  |  |  |  | true |
| etonogestrel | 8 | 42,939 | 336 | 1,396,147 | 0 | 0 | 0 | 0 | 0.82 | 1.00 | -0.197 | 2.850 | -0.069 | 0.945 |  |  |  |  |  | true |
| sulfasalazine | 11 | 42,936 | 455 | 1,396,028 | 0 | 0 | 0 | 0 | 0.82 | 1.00 | -0.197 | 2.844 | -0.069 | 0.945 |  |  |  |  |  | true |
| peginterferon alfa 2a | 5 | 42,942 | 218 | 1,396,265 | 5 | 23,955 | 229 | 1,151,469 | 0.82 | 1.15 | -0.342 | 0.610 | -0.560 | 0.576 |  | 0.591 |  | 0.87 | 0.670 | false |
| diazepam | 17 | 42,930 | 702 | 1,395,781 | 7 | 23,953 | 567 | 1,151,131 | 0.81 | 0.64 | 0.243 | 0.440 | 0.552 | 0.581 |  | 0.531 |  | 0.72 | 0.107 | false |
| tiotropium bromide | 15 | 42,932 | 623 | 1,395,860 | 5 | 23,955 | 510 | 1,151,188 | 0.81 | 0.52 | 0.445 | 0.500 | 0.891 | 0.373 |  | 0.324 |  | 0.67 | 0.075 | false |
| abrocitinib | 10 | 42,937 | 423 | 1,396,060 | 5 | 23,955 | 409 | 1,151,289 | 0.81 | 0.65 | 0.222 | 0.531 | 0.419 | 0.676 |  | 0.625 |  | 0.70 | 0.163 | false |
| rotigotine | 17 | 42,930 | 708 | 1,395,775 | 26 | 23,934 | 777 | 1,150,921 | 0.80 | 1.64 | -0.713 | 0.312 | -2.283 | 0.022 |  | 0.020 |  | 1.14 | 0.411 | false |
| teriparatide | 49 | 42,898 | 2,012 | 1,394,471 | 5 | 23,955 | 660 | 1,151,038 | 0.80 | 0.40 | 0.692 | 0.452 | 1.532 | 0.125 |  | 0.091 |  | 0.71 | 0.014 | false |
| olodaterol tiotropium bromide | 5 | 42,942 | 224 | 1,396,259 | 0 | 0 | 0 | 0 | 0.80 | 1.00 | -0.227 | 2.861 | -0.079 | 0.937 |  |  |  |  |  | true |
| isotretinoin | 14 | 42,933 | 592 | 1,395,891 | 18 | 23,942 | 504 | 1,151,194 | 0.80 | 1.76 | -0.796 | 0.356 | -2.235 | 0.025 |  | 0.023 |  | 1.12 | 0.535 | false |
| mitotane | 5 | 42,942 | 226 | 1,396,257 | 0 | 0 | 0 | 0 | 0.79 | 1.00 | -0.236 | 2.861 | -0.083 | 0.934 |  |  |  |  |  | true |
| benralizumab | 51 | 42,896 | 2,123 | 1,394,360 | 20 | 23,940 | 1,217 | 1,150,481 | 0.79 | 0.81 | -0.026 | 0.264 | -0.099 | 0.921 |  | 0.966 |  | 0.78 | 0.041 | false |
| human immunoglobulin g hyaluronidase human recombinant | 17 | 42,930 | 726 | 1,395,757 | 5 | 23,955 | 452 | 1,151,246 | 0.78 | 0.58 | 0.293 | 0.493 | 0.595 | 0.552 |  | 0.481 |  | 0.69 | 0.087 | false |
| lacosamide | 24 | 42,923 | 1,018 | 1,395,465 | 19 | 23,941 | 951 | 1,150,747 | 0.78 | 0.99 | -0.231 | 0.307 | -0.752 | 0.452 |  | 0.467 |  | 0.84 | 0.264 | false |
| tucatinib | 8 | 42,939 | 353 | 1,396,130 | 0 | 0 | 0 | 0 | 0.78 | 1.00 | -0.246 | 2.850 | -0.086 | 0.931 |  |  |  |  |  | true |
| estradiol norethindrone relugolix | 6 | 42,941 | 271 | 1,396,212 | 0 | 0 | 0 | 0 | 0.78 | 1.00 | -0.250 | 2.856 | -0.088 | 0.930 |  |  |  |  |  | true |
| fedratinib | 8 | 42,939 | 355 | 1,396,128 | 0 | 0 | 0 | 0 | 0.78 | 1.00 | -0.252 | 2.850 | -0.088 | 0.930 |  |  |  |  |  | true |
| sunitinib | 23 | 42,924 | 986 | 1,395,497 | 26 | 23,934 | 1,503 | 1,150,195 | 0.77 | 0.85 | -0.090 | 0.286 | -0.313 | 0.755 |  | 0.751 |  | 0.80 | 0.112 | false |
| ivacaftor tezacaftor | 15 | 42,932 | 651 | 1,395,832 | 5 | 23,955 | 533 | 1,151,165 | 0.77 | 0.50 | 0.446 | 0.500 | 0.891 | 0.373 |  | 0.323 |  | 0.64 | 0.048 | false |
| levetiracetam | 46 | 42,901 | 1,961 | 1,394,522 | 44 | 23,916 | 1,819 | 1,149,879 | 0.77 | 1.18 | -0.423 | 0.212 | -1.990 | 0.047 |  | 0.047 |  | 0.92 | 0.421 | false |
| teduglutide | 45 | 42,902 | 1,926 | 1,394,557 | 22 | 23,938 | 1,613 | 1,150,085 | 0.77 | 0.67 | 0.136 | 0.260 | 0.524 | 0.600 |  | 0.574 |  | 0.72 | 0.008 | false |
| ticagrelor | 26 | 42,921 | 1,122 | 1,395,361 | 58 | 23,902 | 1,846 | 1,149,852 | 0.77 | 1.52 | -0.686 | 0.237 | -2.890 | 0.004 |  | 0.003 |  | 1.15 | 0.194 | false |
| regorafenib | 17 | 42,930 | 741 | 1,395,742 | 30 | 23,930 | 1,048 | 1,150,650 | 0.77 | 1.40 | -0.600 | 0.304 | -1.976 | 0.048 |  | 0.043 |  | 1.06 | 0.714 | false |
| valsartan | 19 | 42,928 | 832 | 1,395,651 | 16 | 23,944 | 827 | 1,150,871 | 0.76 | 0.96 | -0.230 | 0.338 | -0.680 | 0.497 |  | 0.511 |  | 0.82 | 0.240 | false |
| atenolol | 10 | 42,937 | 448 | 1,396,035 | 5 | 23,955 | 316 | 1,151,382 | 0.76 | 0.84 | -0.093 | 0.532 | -0.175 | 0.861 |  | 0.933 |  | 0.74 | 0.240 | false |
| omeprazole | 60 | 42,887 | 2,621 | 1,393,862 | 32 | 23,928 | 2,014 | 1,149,684 | 0.75 | 0.78 | -0.033 | 0.220 | -0.150 | 0.881 |  | 0.907 |  | 0.75 | 0.006 | false |
| diclofenac | 26 | 42,921 | 1,150 | 1,395,333 | 7 | 23,953 | 1,023 | 1,150,675 | 0.75 | 0.35 | 0.755 | 0.416 | 1.815 | 0.070 |  | 0.054 |  | 0.58 | 0.002 | false |
| bimekizumab bkzx | 6 | 42,941 | 282 | 1,396,201 | 0 | 0 | 0 | 0 | 0.75 | 1.00 | -0.290 | 2.856 | -0.102 | 0.919 |  |  |  |  |  | true |
| pegvaliase pqpz | 10 | 42,937 | 456 | 1,396,027 | 6 | 23,954 | 372 | 1,151,326 | 0.75 | 0.84 | -0.115 | 0.504 | -0.227 | 0.820 |  | 0.872 |  | 0.74 | 0.221 | false |
| doxycycline | 16 | 42,931 | 720 | 1,395,763 | 0 | 0 | 0 | 0 | 0.74 | 1.00 | -0.295 | 2.839 | -0.104 | 0.917 |  |  |  |  |  | true |
| eltrombopag | 11 | 42,936 | 502 | 1,395,981 | 0 | 0 | 0 | 0 | 0.74 | 1.00 | -0.296 | 2.844 | -0.104 | 0.917 |  |  |  |  |  | true |
| ruxolitinib | 9 | 42,938 | 417 | 1,396,066 | 0 | 0 | 0 | 0 | 0.74 | 1.00 | -0.301 | 2.847 | -0.106 | 0.916 |  |  |  |  |  | true |
| capecitabine | 65 | 42,882 | 2,921 | 1,393,562 | 31 | 23,929 | 2,184 | 1,149,514 | 0.73 | 0.69 | 0.051 | 0.219 | 0.231 | 0.817 |  | 0.789 |  | 0.71 | <0.001 | false |
| ravulizumab cwvz | 8 | 42,939 | 381 | 1,396,102 | 13 | 23,947 | 354 | 1,151,344 | 0.72 | 1.83 | -0.927 | 0.444 | -2.088 | 0.037 |  | 0.031 |  | 1.10 | 0.657 | false |
| linaclotide | 14 | 42,933 | 653 | 1,395,830 | 7 | 23,953 | 383 | 1,151,315 | 0.72 | 0.94 | -0.265 | 0.454 | -0.583 | 0.560 |  | 0.620 |  | 0.75 | 0.188 | false |
| haloperidol | 8 | 42,939 | 384 | 1,396,099 | 9 | 23,951 | 528 | 1,151,170 | 0.72 | 0.86 | -0.184 | 0.477 | -0.386 | 0.700 |  | 0.698 |  | 0.75 | 0.230 | false |
| laronidase | 7 | 42,940 | 339 | 1,396,144 | 5 | 23,955 | 323 | 1,151,375 | 0.72 | 0.82 | -0.129 | 0.567 | -0.228 | 0.820 |  | 0.862 |  | 0.70 | 0.218 | false |
| metronidazole | 15 | 42,932 | 703 | 1,395,780 | 6 | 23,954 | 426 | 1,151,272 | 0.72 | 0.73 | -0.022 | 0.471 | -0.047 | 0.962 |  | 0.960 |  | 0.69 | 0.089 | false |
| hydrocortisone | 9 | 42,938 | 432 | 1,396,051 | 0 | 0 | 0 | 0 | 0.71 | 1.00 | -0.337 | 2.847 | -0.118 | 0.906 |  |  |  |  |  | true |
| casirivimab imdevimab | 7 | 42,940 | 343 | 1,396,140 | 0 | 0 | 0 | 0 | 0.71 | 1.00 | -0.343 | 2.852 | -0.120 | 0.904 |  |  |  |  |  | true |
| amlodipine besylate | 42 | 42,905 | 1,947 | 1,394,536 | 38 | 23,922 | 1,597 | 1,150,101 | 0.71 | 1.16 | -0.491 | 0.225 | -2.179 | 0.029 |  | 0.029 |  | 0.86 | 0.182 | false |
| lorlatinib | 17 | 42,930 | 804 | 1,395,679 | 16 | 23,944 | 737 | 1,150,961 | 0.71 | 1.08 | -0.419 | 0.347 | -1.208 | 0.227 |  | 0.233 |  | 0.82 | 0.272 | false |
| insulin glargine | 34 | 42,913 | 1,595 | 1,394,888 | 15 | 23,945 | 1,391 | 1,150,307 | 0.70 | 0.54 | 0.273 | 0.308 | 0.885 | 0.376 |  | 0.350 |  | 0.63 | 0.001 | false |
| sapropterin dihydrochloride | 10 | 42,937 | 486 | 1,395,997 | 0 | 0 | 0 | 0 | 0.70 | 1.00 | -0.354 | 2.846 | -0.124 | 0.901 |  |  |  |  |  | true |
| amoxicillin clavulanate | 12 | 42,935 | 581 | 1,395,902 | 0 | 0 | 0 | 0 | 0.70 | 1.00 | -0.358 | 2.843 | -0.126 | 0.900 |  |  |  |  |  | true |
| rivastigmine | 13 | 42,934 | 635 | 1,395,848 | 14 | 23,946 | 463 | 1,151,235 | 0.69 | 1.50 | -0.778 | 0.383 | -2.031 | 0.042 |  | 0.040 |  | 0.93 | 0.699 | false |
| dasatinib | 39 | 42,908 | 1,870 | 1,394,613 | 30 | 23,930 | 1,903 | 1,149,795 | 0.69 | 0.77 | -0.115 | 0.243 | -0.472 | 0.637 |  | 0.650 |  | 0.71 | 0.005 | false |
| candesartan | 6 | 42,941 | 308 | 1,396,175 | 0 | 0 | 0 | 0 | 0.69 | 1.00 | -0.378 | 2.856 | -0.132 | 0.895 |  |  |  |  |  | true |
| diphenhydramine | 8 | 42,939 | 406 | 1,396,077 | 0 | 0 | 0 | 0 | 0.68 | 1.00 | -0.386 | 2.850 | -0.135 | 0.892 |  |  |  |  |  | true |
| everolimus | 44 | 42,903 | 2,138 | 1,394,345 | 17 | 23,943 | 1,503 | 1,150,195 | 0.68 | 0.56 | 0.190 | 0.284 | 0.669 | 0.503 |  | 0.469 |  | 0.63 | <0.001 | false |
| dexamethasone | 25 | 42,922 | 1,227 | 1,395,256 | 18 | 23,942 | 1,323 | 1,150,375 | 0.68 | 0.67 | 0.005 | 0.308 | 0.018 | 0.986 |  | 0.967 |  | 0.66 | 0.006 | false |
| lapatinib ditosylate | 8 | 42,939 | 410 | 1,396,073 | 0 | 0 | 0 | 0 | 0.67 | 1.00 | -0.396 | 2.850 | -0.139 | 0.890 |  |  |  |  |  | true |
| tezepelumab ekko | 8 | 42,939 | 410 | 1,396,073 | 0 | 0 | 0 | 0 | 0.67 | 1.00 | -0.396 | 2.850 | -0.139 | 0.890 |  |  |  |  |  | true |
| tafamidis | 7 | 42,940 | 362 | 1,396,121 | 21 | 23,939 | 797 | 1,150,901 | 0.67 | 1.30 | -0.656 | 0.429 | -1.529 | 0.126 |  | 0.105 |  | 1.01 | 0.951 | false |
| donepezil | 11 | 42,936 | 556 | 1,395,927 | 7 | 23,953 | 368 | 1,151,330 | 0.67 | 0.98 | -0.376 | 0.474 | -0.792 | 0.428 |  | 0.469 |  | 0.73 | 0.180 | false |
| romosozumab aqqg | 22 | 42,925 | 1,089 | 1,395,394 | 0 | 0 | 0 | 0 | 0.67 | 1.00 | -0.398 | 2.836 | -0.140 | 0.888 |  |  |  |  |  | true |
| itraconazole | 5 | 42,942 | 266 | 1,396,217 | 0 | 0 | 0 | 0 | 0.67 | 1.00 | -0.399 | 2.861 | -0.139 | 0.889 |  |  |  |  |  | true |
| exenatide | 24 | 42,923 | 1,194 | 1,395,289 | 16 | 23,944 | 1,026 | 1,150,672 | 0.67 | 0.77 | -0.147 | 0.321 | -0.458 | 0.647 |  | 0.674 |  | 0.69 | 0.019 | false |
| aprepitant | 5 | 42,942 | 271 | 1,396,212 | 0 | 0 | 0 | 0 | 0.66 | 1.00 | -0.418 | 2.861 | -0.146 | 0.884 |  |  |  |  |  | true |
| celecoxib | 21 | 42,926 | 1,062 | 1,395,421 | 12 | 23,948 | 545 | 1,151,153 | 0.66 | 1.10 | -0.516 | 0.360 | -1.433 | 0.152 |  | 0.169 |  | 0.75 | 0.102 | false |
| alectinib | 32 | 42,915 | 1,614 | 1,394,869 | 11 | 23,949 | 1,214 | 1,150,484 | 0.65 | 0.45 | 0.364 | 0.345 | 1.053 | 0.292 |  | 0.261 |  | 0.57 | <0.001 | false |
| cabozantinib s | 35 | 42,912 | 1,769 | 1,394,714 | 97 | 23,863 | 3,118 | 1,148,580 | 0.65 | 1.50 | -0.836 | 0.198 | -4.214 | <0.001 |  | <0.001 |  | 1.11 | 0.240 | false |
| liraglutide | 36 | 42,911 | 1,821 | 1,394,662 | 14 | 23,946 | 948 | 1,150,750 | 0.65 | 0.73 | -0.120 | 0.313 | -0.385 | 0.700 |  | 0.754 |  | 0.66 | 0.003 | false |
| parathyroid hormone | 10 | 42,937 | 524 | 1,395,959 | 0 | 0 | 0 | 0 | 0.65 | 1.00 | -0.429 | 2.846 | -0.151 | 0.880 |  |  |  |  |  | true |
| naproxen | 24 | 42,923 | 1,238 | 1,395,245 | 7 | 23,953 | 851 | 1,150,847 | 0.64 | 0.42 | 0.418 | 0.420 | 0.997 | 0.319 |  | 0.276 |  | 0.56 | <0.001 | false |
| insulin degludec | 17 | 42,930 | 885 | 1,395,598 | 10 | 23,950 | 590 | 1,151,108 | 0.64 | 0.85 | -0.285 | 0.394 | -0.724 | 0.469 |  | 0.507 |  | 0.68 | 0.049 | false |
| dapagliflozin propanediol | 18 | 42,929 | 938 | 1,395,545 | 15 | 23,945 | 1,000 | 1,150,698 | 0.64 | 0.74 | -0.150 | 0.347 | -0.432 | 0.666 |  | 0.682 |  | 0.66 | 0.019 | false |
| drospirenone ethinyl estradiol | 20 | 42,927 | 1,041 | 1,395,442 | 0 | 0 | 0 | 0 | 0.64 | 1.00 | -0.447 | 2.837 | -0.157 | 0.875 |  |  |  |  |  | true |
| risperidone | 30 | 42,917 | 1,549 | 1,394,934 | 124 | 23,836 | 3,509 | 1,148,189 | 0.64 | 1.71 | -0.982 | 0.204 | -4.804 | <0.001 |  | <0.001 |  | 1.28 | 0.002 | false |
| paricalcitol | 6 | 42,941 | 330 | 1,396,153 | 7 | 23,953 | 369 | 1,151,329 | 0.64 | 0.98 | -0.422 | 0.541 | -0.781 | 0.435 |  | 0.437 |  | 0.73 | 0.259 | false |
| molnupiravir | 17 | 42,930 | 894 | 1,395,589 | 11 | 23,949 | 717 | 1,150,981 | 0.64 | 0.77 | -0.192 | 0.383 | -0.500 | 0.617 |  | 0.651 |  | 0.66 | 0.028 | false |
| avatrombopag | 6 | 42,941 | 332 | 1,396,151 | 5 | 23,955 | 246 | 1,151,452 | 0.64 | 1.07 | -0.523 | 0.585 | -0.894 | 0.372 |  | 0.401 |  | 0.72 | 0.275 | false |
| apomorphine | 7 | 42,940 | 384 | 1,396,099 | 17 | 23,943 | 544 | 1,151,154 | 0.63 | 1.55 | -0.891 | 0.442 | -2.017 | 0.044 |  | 0.034 |  | 1.04 | 0.849 | false |
| lenvatinib | 78 | 42,869 | 4,030 | 1,392,453 | 69 | 23,891 | 4,307 | 1,147,391 | 0.63 | 0.77 | -0.203 | 0.166 | -1.219 | 0.223 |  | 0.226 |  | 0.69 | <0.001 | false |
| sorafenib | 7 | 42,940 | 389 | 1,396,094 | 8 | 23,952 | 885 | 1,150,813 | 0.63 | 0.46 | 0.306 | 0.505 | 0.605 | 0.545 |  | 0.566 |  | 0.49 | 0.006 | false |
| clonidine | 8 | 42,939 | 441 | 1,396,042 | 6 | 23,954 | 276 | 1,151,422 | 0.63 | 1.13 | -0.591 | 0.527 | -1.121 | 0.262 |  | 0.288 |  | 0.73 | 0.233 | false |
| hydroxychloroquine | 11 | 42,936 | 599 | 1,395,884 | 0 | 0 | 0 | 0 | 0.62 | 1.00 | -0.472 | 2.844 | -0.166 | 0.868 |  |  |  |  |  | true |
| belumosudil | 6 | 42,941 | 340 | 1,396,143 | 7 | 23,953 | 387 | 1,151,311 | 0.62 | 0.93 | -0.405 | 0.541 | -0.748 | 0.454 |  | 0.456 |  | 0.70 | 0.205 | false |
| ubrogepant | 5 | 42,942 | 288 | 1,396,195 | 0 | 0 | 0 | 0 | 0.62 | 1.00 | -0.478 | 2.861 | -0.167 | 0.867 |  |  |  |  |  | true |
| irbesartan | 9 | 42,938 | 511 | 1,395,972 | 0 | 0 | 0 | 0 | 0.60 | 1.00 | -0.504 | 2.847 | -0.177 | 0.859 |  |  |  |  |  | true |
| zoledronic acid | 51 | 42,896 | 2,787 | 1,393,696 | 0 | 0 | 0 | 0 | 0.60 | 1.00 | -0.510 | 2.832 | -0.180 | 0.857 |  |  |  |  |  | true |
| abobotulinumtoxina | 6 | 42,941 | 354 | 1,396,129 | 0 | 0 | 0 | 0 | 0.60 | 1.00 | -0.517 | 2.856 | -0.181 | 0.856 |  |  |  |  |  | true |
| glutamine | 10 | 42,937 | 574 | 1,395,909 | 0 | 0 | 0 | 0 | 0.59 | 1.00 | -0.521 | 2.846 | -0.183 | 0.855 |  |  |  |  |  | true |
| glatiramer | 48 | 42,899 | 2,661 | 1,393,822 | 16 | 23,944 | 1,070 | 1,150,628 | 0.59 | 0.74 | -0.224 | 0.287 | -0.779 | 0.436 |  | 0.483 |  | 0.61 | <0.001 | false |
| brodalumab | 8 | 42,939 | 468 | 1,396,015 | 7 | 23,953 | 581 | 1,151,117 | 0.59 | 0.62 | -0.050 | 0.505 | -0.098 | 0.922 |  | 0.937 |  | 0.57 | 0.027 | false |
| pantoprazole | 31 | 42,916 | 1,736 | 1,394,747 | 21 | 23,939 | 1,451 | 1,150,247 | 0.59 | 0.71 | -0.188 | 0.282 | -0.668 | 0.504 |  | 0.525 |  | 0.62 | <0.001 | false |
| thalidomide | 16 | 42,931 | 914 | 1,395,569 | 9 | 23,951 | 978 | 1,150,720 | 0.59 | 0.47 | 0.229 | 0.410 | 0.559 | 0.576 |  | 0.546 |  | 0.52 | <0.001 | false |
| spironolactone | 11 | 42,936 | 638 | 1,395,845 | 0 | 0 | 0 | 0 | 0.59 | 1.00 | -0.535 | 2.844 | -0.188 | 0.851 |  |  |  |  |  | true |
| fostamatinib | 13 | 42,934 | 750 | 1,395,733 | 11 | 23,949 | 589 | 1,151,109 | 0.58 | 0.94 | -0.472 | 0.405 | -1.165 | 0.244 |  | 0.256 |  | 0.68 | 0.060 | false |
| lamotrigine | 27 | 42,920 | 1,528 | 1,394,955 | 21 | 23,939 | 674 | 1,151,024 | 0.58 | 1.53 | -0.964 | 0.292 | -3.303 | <0.001 |  | <0.001 |  | 0.79 | 0.102 | false |
| tenofovir alafenamide | 6 | 42,941 | 364 | 1,396,119 | 0 | 0 | 0 | 0 | 0.58 | 1.00 | -0.545 | 2.856 | -0.191 | 0.849 |  |  |  |  |  | true |
| diltiazem | 10 | 42,937 | 592 | 1,395,891 | 5 | 23,955 | 356 | 1,151,342 | 0.58 | 0.74 | -0.252 | 0.531 | -0.475 | 0.634 |  | 0.708 |  | 0.59 | 0.038 | false |
| phenytoin | 6 | 42,941 | 368 | 1,396,115 | 8 | 23,952 | 431 | 1,151,267 | 0.57 | 0.95 | -0.501 | 0.526 | -0.953 | 0.340 |  | 0.334 |  | 0.69 | 0.168 | false |
| riociguat | 17 | 42,930 | 992 | 1,395,491 | 8 | 23,952 | 657 | 1,151,041 | 0.57 | 0.62 | -0.081 | 0.421 | -0.191 | 0.848 |  | 0.909 |  | 0.57 | 0.004 | false |
| tadalafil | 22 | 42,925 | 1,278 | 1,395,205 | 12 | 23,948 | 1,246 | 1,150,452 | 0.57 | 0.48 | 0.172 | 0.355 | 0.484 | 0.629 |  | 0.598 |  | 0.52 | <0.001 | false |
| lanadelumab | 11 | 42,936 | 654 | 1,395,829 | 0 | 0 | 0 | 0 | 0.57 | 1.00 | -0.560 | 2.844 | -0.197 | 0.844 |  |  |  |  |  | true |
| dornase alfa | 8 | 42,939 | 486 | 1,395,997 | 6 | 23,954 | 404 | 1,151,294 | 0.57 | 0.77 | -0.307 | 0.525 | -0.585 | 0.559 |  | 0.596 |  | 0.60 | 0.055 | false |
| denosumab | 57 | 42,890 | 3,337 | 1,393,146 | 10 | 23,950 | 1,041 | 1,150,657 | 0.56 | 0.48 | 0.144 | 0.338 | 0.428 | 0.669 |  | 0.593 |  | 0.54 | <0.001 | false |
| azithromycin | 17 | 42,930 | 1,018 | 1,395,465 | 14 | 23,946 | 777 | 1,150,921 | 0.56 | 0.90 | -0.473 | 0.358 | -1.320 | 0.187 |  | 0.196 |  | 0.65 | 0.018 | false |
| zolpidem | 10 | 42,937 | 617 | 1,395,866 | 10 | 23,950 | 453 | 1,151,245 | 0.55 | 1.11 | -0.700 | 0.441 | -1.587 | 0.112 |  | 0.114 |  | 0.71 | 0.120 | false |
| olaparib | 40 | 42,907 | 2,403 | 1,394,080 | 9 | 23,951 | 736 | 1,150,962 | 0.55 | 0.62 | -0.124 | 0.363 | -0.342 | 0.732 |  | 0.823 |  | 0.55 | <0.001 | false |
| insulin human | 17 | 42,930 | 1,042 | 1,395,441 | 9 | 23,951 | 898 | 1,150,800 | 0.55 | 0.51 | 0.071 | 0.406 | 0.176 | 0.860 |  | 0.816 |  | 0.51 | <0.001 | false |
| encorafenib | 17 | 42,930 | 1,047 | 1,395,436 | 13 | 23,947 | 1,084 | 1,150,614 | 0.54 | 0.60 | -0.097 | 0.365 | -0.265 | 0.791 |  | 0.813 |  | 0.55 | <0.001 | false |
| hyaluronidase zzxf pertuzumab trastuzumab | 8 | 42,939 | 509 | 1,395,974 | 0 | 0 | 0 | 0 | 0.54 | 1.00 | -0.612 | 2.849 | -0.215 | 0.830 |  |  |  |  |  | true |
| clindamycin | 12 | 42,935 | 749 | 1,395,734 | 0 | 0 | 0 | 0 | 0.54 | 1.00 | -0.612 | 2.843 | -0.215 | 0.829 |  |  |  |  |  | true |
| venetoclax | 65 | 42,882 | 3,952 | 1,392,531 | 73 | 23,887 | 5,508 | 1,146,190 | 0.54 | 0.64 | -0.174 | 0.171 | -1.014 | 0.311 |  | 0.310 |  | 0.58 | <0.001 | false |
| peginterferon beta 1a | 14 | 42,933 | 878 | 1,395,605 | 5 | 23,955 | 253 | 1,151,445 | 0.54 | 1.04 | -0.665 | 0.506 | -1.314 | 0.189 |  | 0.242 |  | 0.59 | 0.021 | false |
| budesonide formoterol glycopyrrolate | 5 | 42,942 | 336 | 1,396,147 | 0 | 0 | 0 | 0 | 0.53 | 1.00 | -0.632 | 2.861 | -0.221 | 0.825 |  |  |  |  |  | true |
| olanzapine | 22 | 42,925 | 1,380 | 1,395,103 | 42 | 23,918 | 1,395 | 1,150,303 | 0.53 | 1.46 | -1.017 | 0.264 | -3.858 | <0.001 |  | <0.001 |  | 0.90 | 0.392 | false |
| sitagliptin | 19 | 42,928 | 1,199 | 1,395,284 | 17 | 23,943 | 1,217 | 1,150,481 | 0.53 | 0.69 | -0.268 | 0.332 | -0.807 | 0.420 |  | 0.430 |  | 0.58 | <0.001 | false |
| cyclophosphamide | 27 | 42,920 | 1,696 | 1,394,787 | 5 | 23,955 | 1,175 | 1,150,523 | 0.53 | 0.22 | 0.852 | 0.469 | 1.818 | 0.069 |  | 0.049 |  | 0.42 | <0.001 | false |
| medroxyprogesterone | 10 | 42,937 | 650 | 1,395,833 | 0 | 0 | 0 | 0 | 0.52 | 1.00 | -0.645 | 2.845 | -0.227 | 0.821 |  |  |  |  |  | true |
| metformin sitagliptin | 10 | 42,937 | 655 | 1,395,828 | 11 | 23,949 | 683 | 1,151,015 | 0.52 | 0.81 | -0.440 | 0.430 | -1.023 | 0.307 |  | 0.309 |  | 0.61 | 0.024 | false |
| ramipril | 13 | 42,934 | 846 | 1,395,637 | 5 | 23,955 | 891 | 1,150,807 | 0.52 | 0.30 | 0.559 | 0.508 | 1.100 | 0.271 |  | 0.236 |  | 0.40 | <0.001 | false |
| bisoprolol | 10 | 42,937 | 663 | 1,395,820 | 5 | 23,955 | 675 | 1,151,023 | 0.51 | 0.39 | 0.274 | 0.529 | 0.517 | 0.605 |  | 0.559 |  | 0.44 | <0.001 | false |
| insulin lispro | 20 | 42,927 | 1,311 | 1,395,172 | 13 | 23,947 | 1,203 | 1,150,495 | 0.51 | 0.54 | -0.059 | 0.353 | -0.167 | 0.867 |  | 0.898 |  | 0.50 | <0.001 | false |
| romiplostim | 5 | 42,942 | 355 | 1,396,128 | 0 | 0 | 0 | 0 | 0.50 | 1.00 | -0.687 | 2.861 | -0.240 | 0.810 |  |  |  |  |  | true |
| interferon beta 1b | 9 | 42,938 | 615 | 1,395,868 | 0 | 0 | 0 | 0 | 0.50 | 1.00 | -0.690 | 2.847 | -0.242 | 0.809 |  |  |  |  |  | true |
| ascorbic acid polyethylene glycol 3350 chloride ascorbate chloride | 5 | 42,942 | 357 | 1,396,126 | 0 | 0 | 0 | 0 | 0.50 | 1.00 | -0.693 | 2.861 | -0.242 | 0.809 |  |  |  |  |  | true |
| cilastatin imipenem | 8 | 42,939 | 552 | 1,395,931 | 12 | 23,948 | 690 | 1,151,008 | 0.50 | 0.87 | -0.554 | 0.448 | -1.235 | 0.217 |  | 0.207 |  | 0.64 | 0.045 | false |
| bedaquiline | 7 | 42,940 | 489 | 1,395,994 | 6 | 23,954 | 690 | 1,151,008 | 0.50 | 0.45 | 0.096 | 0.539 | 0.179 | 0.858 |  | 0.847 |  | 0.44 | 0.003 | false |
| imatinib | 26 | 42,921 | 1,755 | 1,394,728 | 20 | 23,940 | 1,889 | 1,149,809 | 0.49 | 0.52 | -0.060 | 0.296 | -0.204 | 0.838 |  | 0.855 |  | 0.49 | <0.001 | false |
| osimertinib | 39 | 42,908 | 2,648 | 1,393,835 | 21 | 23,939 | 1,827 | 1,149,871 | 0.48 | 0.57 | -0.154 | 0.270 | -0.570 | 0.568 |  | 0.599 |  | 0.50 | <0.001 | false |
| fluconazole | 9 | 42,938 | 644 | 1,395,839 | 6 | 23,954 | 519 | 1,151,179 | 0.48 | 0.60 | -0.227 | 0.512 | -0.443 | 0.658 |  | 0.704 |  | 0.49 | 0.005 | false |
| canakinumab | 16 | 42,931 | 1,119 | 1,395,364 | 11 | 23,949 | 879 | 1,150,819 | 0.48 | 0.63 | -0.271 | 0.387 | -0.701 | 0.483 |  | 0.512 |  | 0.51 | <0.001 | false |
| droxidopa | 14 | 42,933 | 994 | 1,395,489 | 19 | 23,941 | 868 | 1,150,830 | 0.47 | 1.08 | -0.823 | 0.350 | -2.352 | 0.019 |  | 0.016 |  | 0.68 | 0.027 | false |
| oxcarbazepine | 6 | 42,941 | 453 | 1,396,030 | 7 | 23,953 | 412 | 1,151,286 | 0.47 | 0.87 | -0.629 | 0.540 | -1.164 | 0.244 |  | 0.246 |  | 0.58 | 0.047 | false |
| human c1 esterase inhibitor | 23 | 42,924 | 1,662 | 1,394,821 | 0 | 0 | 0 | 0 | 0.46 | 1.00 | -0.778 | 2.836 | -0.274 | 0.784 |  |  |  |  |  | true |
| enasidenib | 9 | 42,938 | 674 | 1,395,809 | 9 | 23,951 | 871 | 1,150,827 | 0.46 | 0.52 | -0.135 | 0.462 | -0.291 | 0.771 |  | 0.777 |  | 0.46 | <0.001 | false |
| tobramycin | 7 | 42,940 | 535 | 1,395,948 | 0 | 0 | 0 | 0 | 0.46 | 1.00 | -0.787 | 2.852 | -0.276 | 0.783 |  |  |  |  |  | true |
| pembrolizumab | 46 | 42,901 | 3,317 | 1,393,166 | 37 | 23,923 | 3,475 | 1,148,223 | 0.46 | 0.52 | -0.129 | 0.221 | -0.584 | 0.559 |  | 0.569 |  | 0.48 | <0.001 | false |
| pazopanib | 15 | 42,932 | 1,115 | 1,395,368 | 17 | 23,943 | 1,414 | 1,150,284 | 0.45 | 0.59 | -0.275 | 0.351 | -0.782 | 0.434 |  | 0.434 |  | 0.50 | <0.001 | false |
| esomeprazole | 45 | 42,902 | 3,284 | 1,393,199 | 18 | 23,942 | 2,700 | 1,148,998 | 0.45 | 0.33 | 0.314 | 0.277 | 1.132 | 0.258 |  | 0.237 |  | 0.40 | <0.001 | false |
| efavirenz emtricitabine tenofovir disoproxil | 11 | 42,936 | 831 | 1,395,652 | 40 | 23,920 | 1,724 | 1,149,974 | 0.45 | 1.13 | -0.921 | 0.337 | -2.733 | 0.006 |  | 0.004 |  | 0.83 | 0.192 | false |
| acetaminophen | 40 | 42,907 | 2,948 | 1,393,535 | 12 | 23,948 | 1,943 | 1,149,755 | 0.45 | 0.31 | 0.368 | 0.325 | 1.132 | 0.258 |  | 0.228 |  | 0.40 | <0.001 | false |
| cyclosporine | 15 | 42,932 | 1,130 | 1,395,353 | 11 | 23,949 | 1,254 | 1,150,444 | 0.45 | 0.44 | 0.012 | 0.391 | 0.030 | 0.976 |  | 0.953 |  | 0.43 | <0.001 | false |
| tolvaptan | 15 | 42,932 | 1,133 | 1,395,350 | 26 | 23,934 | 1,052 | 1,150,646 | 0.44 | 1.21 | -1.002 | 0.323 | -3.105 | 0.002 |  | 0.001 |  | 0.72 | 0.040 | false |
| ezetimibe | 8 | 42,939 | 622 | 1,395,861 | 6 | 23,954 | 572 | 1,151,126 | 0.44 | 0.55 | -0.206 | 0.524 | -0.394 | 0.694 |  | 0.730 |  | 0.45 | 0.002 | false |
| ustekinumab | 98 | 42,849 | 7,254 | 1,389,229 | 59 | 23,901 | 6,465 | 1,145,233 | 0.44 | 0.44 | -0.002 | 0.165 | -0.010 | 0.992 |  | 0.992 |  | 0.44 | <0.001 | false |
| tacrolimus | 28 | 42,919 | 2,121 | 1,394,362 | 19 | 23,941 | 2,292 | 1,149,406 | 0.44 | 0.41 | 0.066 | 0.296 | 0.225 | 0.822 |  | 0.802 |  | 0.42 | <0.001 | false |
| amiodarone | 10 | 42,937 | 785 | 1,395,698 | 15 | 23,945 | 1,111 | 1,150,587 | 0.43 | 0.67 | -0.433 | 0.402 | -1.076 | 0.282 |  | 0.271 |  | 0.53 | 0.001 | false |
| ranitidine | 14 | 42,933 | 1,086 | 1,395,397 | 9 | 23,951 | 910 | 1,150,788 | 0.43 | 0.50 | -0.145 | 0.420 | -0.345 | 0.730 |  | 0.769 |  | 0.44 | <0.001 | false |
| dapagliflozin | 18 | 42,929 | 1,398 | 1,395,085 | 16 | 23,944 | 1,465 | 1,150,233 | 0.43 | 0.54 | -0.230 | 0.341 | -0.674 | 0.500 |  | 0.512 |  | 0.46 | <0.001 | false |
| gadoterate meglumine | 9 | 42,938 | 731 | 1,395,752 | 6 | 23,954 | 331 | 1,151,367 | 0.42 | 0.94 | -0.803 | 0.513 | -1.564 | 0.118 |  | 0.134 |  | 0.51 | 0.009 | false |
| trastuzumab | 43 | 42,904 | 3,367 | 1,393,116 | 0 | 0 | 0 | 0 | 0.42 | 1.00 | -0.869 | 2.833 | -0.307 | 0.759 |  |  |  |  |  | true |
| metformin | 34 | 42,913 | 2,704 | 1,393,779 | 20 | 23,940 | 2,309 | 1,149,389 | 0.41 | 0.43 | -0.028 | 0.280 | -0.100 | 0.920 |  | 0.949 |  | 0.41 | <0.001 | false |
| lansoprazole | 14 | 42,933 | 1,158 | 1,395,325 | 0 | 0 | 0 | 0 | 0.41 | 1.00 | -0.900 | 2.841 | -0.317 | 0.752 |  |  |  |  |  | true |
| insulin detemir | 9 | 42,938 | 765 | 1,395,718 | 5 | 23,955 | 513 | 1,151,185 | 0.40 | 0.51 | -0.244 | 0.539 | -0.452 | 0.651 |  | 0.717 |  | 0.41 | <0.001 | false |
| warfarin | 7 | 42,940 | 606 | 1,395,877 | 0 | 0 | 0 | 0 | 0.40 | 1.00 | -0.911 | 2.852 | -0.320 | 0.749 |  |  |  |  |  | true |
| docetaxel | 28 | 42,919 | 2,332 | 1,394,151 | 0 | 0 | 0 | 0 | 0.40 | 1.00 | -0.924 | 2.835 | -0.326 | 0.744 |  |  |  |  |  | true |
| rivaroxaban | 87 | 42,860 | 7,150 | 1,389,333 | 54 | 23,906 | 7,412 | 1,144,286 | 0.40 | 0.35 | 0.120 | 0.174 | 0.690 | 0.490 |  | 0.479 |  | 0.38 | <0.001 | false |
| mesalamine | 7 | 42,940 | 618 | 1,395,865 | 0 | 0 | 0 | 0 | 0.39 | 1.00 | -0.931 | 2.852 | -0.326 | 0.744 |  |  |  |  |  | true |
| axicabtagene ciloleucel | 12 | 42,935 | 1,034 | 1,395,449 | 6 | 23,954 | 1,431 | 1,150,267 | 0.39 | 0.22 | 0.588 | 0.485 | 1.212 | 0.225 |  | 0.203 |  | 0.29 | <0.001 | false |
| ivacaftor | 6 | 42,941 | 539 | 1,395,944 | 5 | 23,955 | 410 | 1,151,288 | 0.39 | 0.64 | -0.497 | 0.583 | -0.853 | 0.394 |  | 0.424 |  | 0.44 | 0.005 | false |
| emtricitabine tenofovir disoproxil | 18 | 42,929 | 1,536 | 1,394,947 | 49 | 23,911 | 2,747 | 1,148,951 | 0.39 | 0.87 | -0.794 | 0.274 | -2.894 | 0.004 |  | 0.003 |  | 0.64 | <0.001 | false |
| tralokinumab ldrm | 5 | 42,942 | 459 | 1,396,024 | 0 | 0 | 0 | 0 | 0.39 | 1.00 | -0.944 | 2.861 | -0.330 | 0.741 |  |  |  |  |  | true |
| fulvestrant | 25 | 42,922 | 2,134 | 1,394,349 | 0 | 0 | 0 | 0 | 0.39 | 1.00 | -0.947 | 2.835 | -0.334 | 0.739 |  |  |  |  |  | true |
| sirolimus | 6 | 42,941 | 546 | 1,395,937 | 5 | 23,955 | 667 | 1,151,031 | 0.39 | 0.40 | -0.024 | 0.582 | -0.041 | 0.968 |  | 0.989 |  | 0.36 | <0.001 | false |
| perampanel | 5 | 42,942 | 465 | 1,396,018 | 8 | 23,952 | 444 | 1,151,254 | 0.38 | 0.92 | -0.872 | 0.551 | -1.583 | 0.114 |  | 0.103 |  | 0.55 | 0.031 | false |
| golimumab | 92 | 42,855 | 7,834 | 1,388,649 | 18 | 23,942 | 4,402 | 1,147,296 | 0.38 | 0.20 | 0.642 | 0.256 | 2.512 | 0.012 |  | 0.009 |  | 0.33 | <0.001 | false |
| pertuzumab | 16 | 42,931 | 1,424 | 1,395,059 | 0 | 0 | 0 | 0 | 0.38 | 1.00 | -0.977 | 2.839 | -0.344 | 0.731 |  |  |  |  |  | true |
| mycophenolate mofetil | 13 | 42,934 | 1,175 | 1,395,308 | 12 | 23,948 | 1,117 | 1,150,581 | 0.37 | 0.54 | -0.365 | 0.395 | -0.923 | 0.356 |  | 0.367 |  | 0.42 | <0.001 | false |
| risdiplam | 5 | 42,942 | 480 | 1,396,003 | 0 | 0 | 0 | 0 | 0.37 | 1.00 | -0.989 | 2.861 | -0.346 | 0.730 |  |  |  |  |  | true |
| insulin aspart | 22 | 42,925 | 1,964 | 1,394,519 | 7 | 23,953 | 1,532 | 1,150,166 | 0.37 | 0.23 | 0.460 | 0.423 | 1.086 | 0.277 |  | 0.240 |  | 0.31 | <0.001 | false |
| crizotinib | 10 | 42,937 | 931 | 1,395,552 | 7 | 23,953 | 877 | 1,150,821 | 0.37 | 0.41 | -0.114 | 0.480 | -0.237 | 0.812 |  | 0.850 |  | 0.36 | <0.001 | false |
| hydrochlorothiazide | 5 | 42,942 | 491 | 1,395,992 | 0 | 0 | 0 | 0 | 0.36 | 1.00 | -1.011 | 2.861 | -0.353 | 0.724 |  |  |  |  |  | true |
| valproate | 7 | 42,940 | 670 | 1,395,813 | 6 | 23,954 | 756 | 1,150,942 | 0.36 | 0.41 | -0.127 | 0.539 | -0.236 | 0.814 |  | 0.836 |  | 0.36 | <0.001 | false |
| deferasirox | 7 | 42,940 | 675 | 1,395,808 | 7 | 23,953 | 693 | 1,151,005 | 0.36 | 0.52 | -0.365 | 0.519 | -0.702 | 0.483 |  | 0.495 |  | 0.40 | <0.001 | false |
| trametinib dimethyl sulfoxide | 9 | 42,938 | 873 | 1,395,610 | 10 | 23,950 | 842 | 1,150,856 | 0.35 | 0.60 | -0.527 | 0.450 | -1.170 | 0.242 |  | 0.244 |  | 0.43 | <0.001 | false |
| axitinib | 9 | 42,938 | 884 | 1,395,599 | 33 | 23,927 | 1,559 | 1,150,139 | 0.35 | 1.03 | -1.084 | 0.370 | -2.930 | 0.003 |  | 0.002 |  | 0.71 | 0.024 | false |
| tipiracil trifluridine | 12 | 42,935 | 1,170 | 1,395,313 | 10 | 23,950 | 1,468 | 1,150,230 | 0.35 | 0.34 | 0.011 | 0.421 | 0.025 | 0.980 |  | 0.965 |  | 0.33 | <0.001 | false |
| ado trastuzumab emtansine | 9 | 42,938 | 892 | 1,395,591 | 0 | 0 | 0 | 0 | 0.35 | 1.00 | -1.061 | 2.847 | -0.373 | 0.709 |  |  |  |  |  | true |
| daratumumab | 17 | 42,930 | 1,655 | 1,394,828 | 9 | 23,951 | 2,018 | 1,149,680 | 0.34 | 0.23 | 0.419 | 0.404 | 1.036 | 0.300 |  | 0.279 |  | 0.28 | <0.001 | false |
| valacyclovir | 6 | 42,941 | 615 | 1,395,868 | 0 | 0 | 0 | 0 | 0.34 | 1.00 | -1.069 | 2.856 | -0.374 | 0.708 |  |  |  |  |  | true |
| ivacaftor lumacaftor | 6 | 42,941 | 623 | 1,395,860 | 5 | 23,955 | 594 | 1,151,104 | 0.34 | 0.44 | -0.271 | 0.582 | -0.466 | 0.641 |  | 0.673 |  | 0.35 | <0.001 | false |
| ondansetron | 7 | 42,940 | 739 | 1,395,744 | 6 | 23,954 | 385 | 1,151,313 | 0.33 | 0.81 | -0.899 | 0.540 | -1.667 | 0.096 |  | 0.101 |  | 0.42 | 0.001 | false |
| tenofovir disoproxil | 13 | 42,934 | 1,343 | 1,395,140 | 33 | 23,927 | 2,195 | 1,149,503 | 0.33 | 0.73 | -0.809 | 0.324 | -2.493 | 0.013 |  | 0.010 |  | 0.53 | <0.001 | false |
| sacituzumab govitecan hziy | 6 | 42,941 | 656 | 1,395,827 | 0 | 0 | 0 | 0 | 0.32 | 1.00 | -1.134 | 2.856 | -0.397 | 0.691 |  |  |  |  |  | true |
| bortezomib | 8 | 42,939 | 859 | 1,395,624 | 13 | 23,947 | 1,186 | 1,150,512 | 0.32 | 0.55 | -0.531 | 0.440 | -1.206 | 0.228 |  | 0.215 |  | 0.41 | <0.001 | false |
| chloride | 12 | 42,935 | 1,315 | 1,395,168 | 6 | 23,954 | 776 | 1,150,922 | 0.31 | 0.40 | -0.264 | 0.486 | -0.544 | 0.586 |  | 0.653 |  | 0.32 | <0.001 | false |
| darbepoetin alfa | 5 | 42,942 | 579 | 1,395,904 | 5 | 23,955 | 641 | 1,151,057 | 0.31 | 0.41 | -0.289 | 0.606 | -0.477 | 0.633 |  | 0.648 |  | 0.32 | <0.001 | false |
| nivolumab | 40 | 42,907 | 4,268 | 1,392,215 | 76 | 23,884 | 6,167 | 1,145,531 | 0.31 | 0.59 | -0.659 | 0.196 | -3.369 | <0.001 |  | <0.001 |  | 0.45 | <0.001 | false |
| dabrafenib | 13 | 42,934 | 1,457 | 1,395,026 | 17 | 23,943 | 1,574 | 1,150,124 | 0.30 | 0.53 | -0.573 | 0.364 | -1.574 | 0.115 |  | 0.111 |  | 0.39 | <0.001 | false |
| gemcitabine | 8 | 42,939 | 921 | 1,395,562 | 0 | 0 | 0 | 0 | 0.30 | 1.00 | -1.205 | 2.849 | -0.423 | 0.672 |  |  |  |  |  | true |
| ibuprofen | 25 | 42,922 | 2,767 | 1,393,716 | 20 | 23,940 | 2,267 | 1,149,431 | 0.30 | 0.43 | -0.372 | 0.298 | -1.248 | 0.212 |  | 0.221 |  | 0.34 | <0.001 | false |
| palivizumab | 6 | 42,941 | 708 | 1,395,775 | 13 | 23,947 | 763 | 1,150,935 | 0.30 | 0.85 | -1.047 | 0.480 | -2.180 | 0.029 |  | 0.021 |  | 0.51 | 0.003 | false |
| durvalumab | 11 | 42,936 | 1,263 | 1,395,220 | 9 | 23,951 | 1,910 | 1,149,788 | 0.30 | 0.24 | 0.214 | 0.440 | 0.487 | 0.626 |  | 0.619 |  | 0.25 | <0.001 | false |
| eculizumab | 10 | 42,937 | 1,170 | 1,395,313 | 16 | 23,944 | 776 | 1,150,922 | 0.29 | 1.02 | -1.254 | 0.398 | -3.154 | 0.002 |  | <0.001 |  | 0.50 | <0.001 | false |
| fluorouracil | 6 | 42,941 | 740 | 1,395,743 | 0 | 0 | 0 | 0 | 0.29 | 1.00 | -1.254 | 2.856 | -0.439 | 0.661 |  |  |  |  |  | true |
| ixekizumab | 17 | 42,930 | 1,996 | 1,394,487 | 8 | 23,952 | 1,717 | 1,149,981 | 0.28 | 0.24 | 0.181 | 0.419 | 0.431 | 0.666 |  | 0.621 |  | 0.26 | <0.001 | false |
| carbamazepine | 8 | 42,939 | 983 | 1,395,500 | 11 | 23,949 | 846 | 1,150,852 | 0.28 | 0.65 | -0.843 | 0.455 | -1.854 | 0.064 |  | 0.058 |  | 0.40 | <0.001 | false |
| ferric carboxymaltose | 11 | 42,936 | 1,345 | 1,395,138 | 0 | 0 | 0 | 0 | 0.28 | 1.00 | -1.281 | 2.844 | -0.450 | 0.652 |  |  |  |  |  | true |
| amoxicillin | 11 | 42,936 | 1,420 | 1,395,063 | 6 | 23,954 | 1,025 | 1,150,673 | 0.26 | 0.30 | -0.146 | 0.492 | -0.297 | 0.766 |  | 0.828 |  | 0.26 | <0.001 | false |
| iron sucrose | 5 | 42,942 | 737 | 1,395,746 | 0 | 0 | 0 | 0 | 0.24 | 1.00 | -1.417 | 2.861 | -0.495 | 0.620 |  |  |  |  |  | true |
| agalsidase beta | 5 | 42,942 | 743 | 1,395,740 | 5 | 23,955 | 876 | 1,150,822 | 0.24 | 0.30 | -0.226 | 0.605 | -0.374 | 0.709 |  | 0.721 |  | 0.24 | <0.001 | false |
| gefitinib | 5 | 42,942 | 773 | 1,395,710 | 0 | 0 | 0 | 0 | 0.23 | 1.00 | -1.465 | 2.861 | -0.512 | 0.609 |  |  |  |  |  | true |
| paclitaxel | 22 | 42,925 | 3,394 | 1,393,089 | 0 | 0 | 0 | 0 | 0.22 | 1.00 | -1.537 | 2.836 | -0.542 | 0.588 |  |  |  |  |  | true |
| guselkumab | 14 | 42,933 | 2,211 | 1,394,272 | 8 | 23,952 | 1,889 | 1,149,809 | 0.21 | 0.22 | -0.014 | 0.433 | -0.033 | 0.974 |  | 0.980 |  | 0.20 | <0.001 | false |
| furosemide | 7 | 42,940 | 1,163 | 1,395,320 | 5 | 23,955 | 1,100 | 1,150,598 | 0.21 | 0.24 | -0.136 | 0.563 | -0.242 | 0.809 |  | 0.851 |  | 0.20 | <0.001 | false |
| bevacizumab | 15 | 42,932 | 2,404 | 1,394,079 | 14 | 23,946 | 2,119 | 1,149,579 | 0.21 | 0.33 | -0.450 | 0.367 | -1.229 | 0.219 |  | 0.226 |  | 0.25 | <0.001 | false |
| clozapine | 23 | 42,924 | 3,657 | 1,392,826 | 37 | 23,923 | 4,572 | 1,147,126 | 0.21 | 0.39 | -0.635 | 0.264 | -2.402 | 0.016 |  | 0.014 |  | 0.29 | <0.001 | false |
| atezolizumab | 15 | 42,932 | 2,636 | 1,393,847 | 13 | 23,947 | 2,988 | 1,148,710 | 0.19 | 0.22 | -0.127 | 0.373 | -0.340 | 0.734 |  | 0.748 |  | 0.20 | <0.001 | false |
| pegfilgrastim | 5 | 42,942 | 944 | 1,395,539 | 0 | 0 | 0 | 0 | 0.19 | 1.00 | -1.665 | 2.861 | -0.582 | 0.561 |  |  |  |  |  | true |
| fam trastuzumab deruxtecan nxki | 6 | 42,941 | 1,120 | 1,395,363 | 0 | 0 | 0 | 0 | 0.19 | 1.00 | -1.669 | 2.856 | -0.584 | 0.559 |  |  |  |  |  | true |
| aztreonam lysine | 5 | 42,942 | 956 | 1,395,527 | 0 | 0 | 0 | 0 | 0.19 | 1.00 | -1.677 | 2.861 | -0.586 | 0.558 |  |  |  |  |  | true |
| doxorubicin | 7 | 42,940 | 1,338 | 1,395,145 | 0 | 0 | 0 | 0 | 0.18 | 1.00 | -1.703 | 2.852 | -0.597 | 0.550 |  |  |  |  |  | true |
| obinutuzumab | 6 | 42,941 | 1,188 | 1,395,295 | 7 | 23,953 | 1,538 | 1,150,160 | 0.18 | 0.23 | -0.276 | 0.537 | -0.513 | 0.608 |  | 0.606 |  | 0.19 | <0.001 | false |
| pentosan polysulfate | 5 | 42,942 | 1,024 | 1,395,459 | 0 | 0 | 0 | 0 | 0.17 | 1.00 | -1.746 | 2.861 | -0.610 | 0.542 |  |  |  |  |  | true |
| clopidogrel bisulfate | 5 | 42,942 | 1,116 | 1,395,367 | 7 | 23,953 | 1,619 | 1,150,079 | 0.16 | 0.22 | -0.329 | 0.563 | -0.584 | 0.559 |  | 0.543 |  | 0.18 | <0.001 | false |
| temozolomide | 5 | 42,942 | 1,135 | 1,395,348 | 0 | 0 | 0 | 0 | 0.16 | 1.00 | -1.849 | 2.861 | -0.646 | 0.518 |  |  |  |  |  | true |
| carboplatin | 9 | 42,938 | 2,224 | 1,394,259 | 11 | 23,949 | 1,403 | 1,150,295 | 0.14 | 0.39 | -1.043 | 0.440 | -2.372 | 0.018 |  | 0.015 |  | 0.21 | <0.001 | false |
| empagliflozin | 7 | 42,940 | 1,812 | 1,394,671 | 13 | 23,947 | 2,361 | 1,149,337 | 0.13 | 0.27 | -0.714 | 0.457 | -1.563 | 0.118 |  | 0.105 |  | 0.19 | <0.001 | false |
| dabigatran etexilate | 6 | 42,941 | 1,730 | 1,394,753 | 0 | 0 | 0 | 0 | 0.12 | 1.00 | -2.104 | 2.856 | -0.737 | 0.461 |  |  |  |  |  | true |
| baricitinib | 6 | 42,941 | 1,753 | 1,394,730 | 0 | 0 | 0 | 0 | 0.12 | 1.00 | -2.117 | 2.856 | -0.741 | 0.458 |  |  |  |  |  | true |
| dexmethylphenidate serdexmethylphenidate chloride | 0 | 0 | 0 | 0 | 8 | 23,952 | 44 | 1,151,654 | 1.00 | 9.18 | -2.217 | 2.853 | -0.777 | 0.437 |  |  |  |  |  | true |
| finasteride | 0 | 0 | 0 | 0 | 160 | 23,800 | 1,064 | 1,150,634 | 1.00 | 7.29 | -1.986 | 2.830 | -0.702 | 0.483 |  |  |  |  |  | true |
| relugolix | 0 | 0 | 0 | 0 | 53 | 23,907 | 485 | 1,151,213 | 1.00 | 5.31 | -1.669 | 2.832 | -0.589 | 0.556 |  |  |  |  |  | true |
| buspirone | 0 | 0 | 0 | 0 | 7 | 23,953 | 85 | 1,151,613 | 1.00 | 4.22 | -1.439 | 2.854 | -0.504 | 0.614 |  |  |  |  |  | true |
| maralixibat chloride | 0 | 0 | 0 | 0 | 5 | 23,955 | 65 | 1,151,633 | 1.00 | 4.04 | -1.395 | 2.863 | -0.487 | 0.626 |  |  |  |  |  | true |
| vamorolone | 0 | 0 | 0 | 0 | 5 | 23,955 | 79 | 1,151,619 | 1.00 | 3.33 | -1.202 | 2.863 | -0.420 | 0.675 |  |  |  |  |  | true |
| deflazacort | 0 | 0 | 0 | 0 | 12 | 23,948 | 181 | 1,151,517 | 1.00 | 3.31 | -1.197 | 2.844 | -0.421 | 0.674 |  |  |  |  |  | true |
| abacavir dolutegravir lamivudine | 0 | 0 | 0 | 0 | 20 | 23,940 | 300 | 1,151,398 | 1.00 | 3.28 | -1.188 | 2.838 | -0.419 | 0.675 |  |  |  |  |  | true |
| dexmethylphenidate | 0 | 0 | 0 | 0 | 6 | 23,954 | 95 | 1,151,603 | 1.00 | 3.27 | -1.185 | 2.857 | -0.415 | 0.678 |  |  |  |  |  | true |
| enzalutamide | 0 | 0 | 0 | 0 | 178 | 23,782 | 2,947 | 1,148,751 | 1.00 | 2.93 | -1.073 | 2.829 | -0.379 | 0.704 |  |  |  |  |  | true |
| efavirenz | 0 | 0 | 0 | 0 | 5 | 23,955 | 95 | 1,151,603 | 1.00 | 2.77 | -1.018 | 2.862 | -0.356 | 0.722 |  |  |  |  |  | true |
| desmopressin | 0 | 0 | 0 | 0 | 8 | 23,952 | 160 | 1,151,538 | 1.00 | 2.55 | -0.935 | 2.850 | -0.328 | 0.743 |  |  |  |  |  | true |
| buprenorphine naloxone | 0 | 0 | 0 | 0 | 6 | 23,954 | 143 | 1,151,555 | 1.00 | 2.18 | -0.778 | 2.857 | -0.272 | 0.785 |  |  |  |  |  | true |
| acetaminophen tramadol | 0 | 0 | 0 | 0 | 7 | 23,953 | 167 | 1,151,531 | 1.00 | 2.15 | -0.767 | 2.853 | -0.269 | 0.788 |  |  |  |  |  | true |
| cobicistat elvitegravir emtricitabine tenofovir | 0 | 0 | 0 | 0 | 8 | 23,952 | 190 | 1,151,508 | 1.00 | 2.15 | -0.763 | 2.850 | -0.268 | 0.789 |  |  |  |  |  | true |
| emtricitabine rilpivirine tenofovir alafenamide | 0 | 0 | 0 | 0 | 6 | 23,954 | 181 | 1,151,517 | 1.00 | 1.72 | -0.543 | 2.856 | -0.190 | 0.849 |  |  |  |  |  | true |
| eplerenone | 0 | 0 | 0 | 0 | 6 | 23,954 | 185 | 1,151,513 | 1.00 | 1.68 | -0.521 | 2.856 | -0.183 | 0.855 |  |  |  |  |  | true |
| tesamorelin | 0 | 0 | 0 | 0 | 5 | 23,955 | 159 | 1,151,539 | 1.00 | 1.66 | -0.505 | 2.861 | -0.177 | 0.860 |  |  |  |  |  | true |
| darolutamide | 0 | 0 | 0 | 0 | 17 | 23,943 | 508 | 1,151,190 | 1.00 | 1.65 | -0.504 | 2.839 | -0.177 | 0.859 |  |  |  |  |  | true |
| memantine | 0 | 0 | 0 | 0 | 5 | 23,955 | 161 | 1,151,537 | 1.00 | 1.64 | -0.493 | 2.861 | -0.172 | 0.863 |  |  |  |  |  | true |
| clobazam | 0 | 0 | 0 | 0 | 8 | 23,952 | 252 | 1,151,446 | 1.00 | 1.62 | -0.481 | 2.850 | -0.169 | 0.866 |  |  |  |  |  | true |
| abiraterone | 0 | 0 | 0 | 0 | 116 | 23,844 | 3,489 | 1,148,209 | 1.00 | 1.61 | -0.475 | 2.830 | -0.168 | 0.867 |  |  |  |  |  | true |
| dextromethorphan | 0 | 0 | 0 | 0 | 6 | 23,954 | 194 | 1,151,504 | 1.00 | 1.61 | -0.474 | 2.856 | -0.166 | 0.868 |  |  |  |  |  | true |
| tamsulosin | 0 | 0 | 0 | 0 | 32 | 23,928 | 1,058 | 1,150,640 | 1.00 | 1.48 | -0.390 | 2.834 | -0.137 | 0.891 |  |  |  |  |  | true |
| emtricitabine tenofovir | 0 | 0 | 0 | 0 | 10 | 23,950 | 355 | 1,151,343 | 1.00 | 1.42 | -0.351 | 2.846 | -0.123 | 0.902 |  |  |  |  |  | true |
| ritonavir | 0 | 0 | 0 | 0 | 5 | 23,955 | 193 | 1,151,505 | 1.00 | 1.37 | -0.312 | 2.861 | -0.109 | 0.913 |  |  |  |  |  | true |
| apalutamide | 0 | 0 | 0 | 0 | 83 | 23,877 | 3,005 | 1,148,693 | 1.00 | 1.34 | -0.290 | 2.831 | -0.102 | 0.918 |  |  |  |  |  | true |
| cabotegravir | 0 | 0 | 0 | 0 | 6 | 23,954 | 234 | 1,151,464 | 1.00 | 1.33 | -0.287 | 2.856 | -0.100 | 0.920 |  |  |  |  |  | true |
| elotuzumab | 0 | 0 | 0 | 0 | 5 | 23,955 | 200 | 1,151,498 | 1.00 | 1.32 | -0.277 | 2.861 | -0.097 | 0.923 |  |  |  |  |  | true |
| cobicistat darunavir emtricitabine tenofovir alafenamide | 0 | 0 | 0 | 0 | 7 | 23,953 | 274 | 1,151,424 | 1.00 | 1.31 | -0.273 | 2.853 | -0.096 | 0.924 |  |  |  |  |  | true |
| idursulfase | 0 | 0 | 0 | 0 | 32 | 23,928 | 1,215 | 1,150,483 | 1.00 | 1.29 | -0.251 | 2.834 | -0.089 | 0.929 |  |  |  |  |  | true |
| pralsetinib | 0 | 0 | 0 | 0 | 7 | 23,953 | 280 | 1,151,418 | 1.00 | 1.29 | -0.251 | 2.853 | -0.088 | 0.930 |  |  |  |  |  | true |
| ranolazine | 0 | 0 | 0 | 0 | 8 | 23,952 | 333 | 1,151,365 | 1.00 | 1.23 | -0.203 | 2.850 | -0.071 | 0.943 |  |  |  |  |  | true |
| cabotegravir rilpivirine | 0 | 0 | 0 | 0 | 6 | 23,954 | 255 | 1,151,443 | 1.00 | 1.22 | -0.201 | 2.856 | -0.070 | 0.944 |  |  |  |  |  | true |
| rabeprazole | 0 | 0 | 0 | 0 | 6 | 23,954 | 255 | 1,151,443 | 1.00 | 1.22 | -0.201 | 2.856 | -0.070 | 0.944 |  |  |  |  |  | true |
| emtricitabine tenofovir alafenamide | 0 | 0 | 0 | 0 | 6 | 23,954 | 257 | 1,151,441 | 1.00 | 1.21 | -0.193 | 2.856 | -0.068 | 0.946 |  |  |  |  |  | true |
| capmatinib | 0 | 0 | 0 | 0 | 7 | 23,953 | 303 | 1,151,395 | 1.00 | 1.19 | -0.172 | 2.852 | -0.060 | 0.952 |  |  |  |  |  | true |
| methadone | 0 | 0 | 0 | 0 | 5 | 23,955 | 227 | 1,151,471 | 1.00 | 1.16 | -0.150 | 2.861 | -0.052 | 0.958 |  |  |  |  |  | true |
| dutasteride | 0 | 0 | 0 | 0 | 7 | 23,953 | 317 | 1,151,381 | 1.00 | 1.14 | -0.127 | 2.852 | -0.045 | 0.964 |  |  |  |  |  | true |
| dolutegravir | 0 | 0 | 0 | 0 | 6 | 23,954 | 286 | 1,151,412 | 1.00 | 1.09 | -0.087 | 2.856 | -0.030 | 0.976 |  |  |  |  |  | true |
| testosterone | 0 | 0 | 0 | 0 | 8 | 23,952 | 376 | 1,151,322 | 1.00 | 1.09 | -0.082 | 2.850 | -0.029 | 0.977 |  |  |  |  |  | true |
| edaravone | 0 | 0 | 0 | 0 | 5 | 23,955 | 254 | 1,151,444 | 1.00 | 1.04 | -0.038 | 2.861 | -0.013 | 0.989 |  |  |  |  |  | true |
| cobicistat elvitegravir emtricitabine tenofovir disoproxil | 0 | 0 | 0 | 0 | 11 | 23,949 | 548 | 1,151,150 | 1.00 | 1.01 | -0.008 | 2.844 | -0.003 | 0.998 |  |  |  |  |  | true |
| rilpivirine | 0 | 0 | 0 | 0 | 10 | 23,950 | 507 | 1,151,191 | 1.00 | 0.99 | 0.006 | 2.846 | 0.002 | 0.998 |  |  |  |  |  | true |
| deucravacitinib | 0 | 0 | 0 | 0 | 6 | 23,954 | 319 | 1,151,379 | 1.00 | 0.98 | 0.022 | 2.856 | 0.008 | 0.994 |  |  |  |  |  | true |
| cobicistat elvitegravir emtricitabine tenofovir alafenamide | 0 | 0 | 0 | 0 | 5 | 23,955 | 276 | 1,151,422 | 1.00 | 0.96 | 0.045 | 2.861 | 0.016 | 0.987 |  |  |  |  |  | true |
| pegvisomant | 0 | 0 | 0 | 0 | 6 | 23,954 | 352 | 1,151,346 | 1.00 | 0.89 | 0.121 | 2.856 | 0.042 | 0.966 |  |  |  |  |  | true |
| onasemnogene abeparvovec xioi | 0 | 0 | 0 | 0 | 6 | 23,954 | 409 | 1,151,289 | 1.00 | 0.76 | 0.271 | 2.856 | 0.095 | 0.924 |  |  |  |  |  | true |
| bicalutamide | 0 | 0 | 0 | 0 | 5 | 23,955 | 361 | 1,151,337 | 1.00 | 0.73 | 0.313 | 2.861 | 0.109 | 0.913 |  |  |  |  |  | true |
| dapagliflozin propanediol metformin | 0 | 0 | 0 | 0 | 5 | 23,955 | 395 | 1,151,303 | 1.00 | 0.67 | 0.403 | 2.861 | 0.141 | 0.888 |  |  |  |  |  | true |
| brentuximab vedotin | 0 | 0 | 0 | 0 | 13 | 23,947 | 1,077 | 1,150,621 | 1.00 | 0.60 | 0.508 | 2.842 | 0.179 | 0.858 |  |  |  |  |  | true |
| emtricitabine rilpivirine tenofovir disoproxil | 0 | 0 | 0 | 0 | 5 | 23,955 | 508 | 1,151,190 | 1.00 | 0.52 | 0.654 | 2.861 | 0.229 | 0.819 |  |  |  |  |  | true |
| tafamidis meglumine | 0 | 0 | 0 | 0 | 8 | 23,952 | 828 | 1,150,870 | 1.00 | 0.49 | 0.707 | 2.849 | 0.248 | 0.804 |  |  |  |  |  | true |
| panitumumab | 0 | 0 | 0 | 0 | 6 | 23,954 | 749 | 1,150,949 | 1.00 | 0.42 | 0.875 | 2.856 | 0.307 | 0.759 |  |  |  |  |  | true |
| patiromer | 0 | 0 | 0 | 0 | 6 | 23,954 | 756 | 1,150,942 | 1.00 | 0.41 | 0.885 | 2.856 | 0.310 | 0.757 |  |  |  |  |  | true |
| linezolid | 0 | 0 | 0 | 0 | 9 | 23,951 | 1,194 | 1,150,504 | 1.00 | 0.38 | 0.962 | 2.847 | 0.338 | 0.735 |  |  |  |  |  | true |
| tisagenlecleucel | 0 | 0 | 0 | 0 | 6 | 23,954 | 923 | 1,150,775 | 1.00 | 0.34 | 1.084 | 2.856 | 0.380 | 0.704 |  |  |  |  |  | true |
| ipilimumab | 0 | 0 | 0 | 0 | 10 | 23,950 | 1,610 | 1,150,088 | 1.00 | 0.31 | 1.161 | 2.845 | 0.408 | 0.683 |  |  |  |  |  | true |
| canagliflozin | 0 | 0 | 0 | 0 | 7 | 23,953 | 1,291 | 1,150,407 | 1.00 | 0.28 | 1.277 | 2.852 | 0.448 | 0.654 |  |  |  |  |  | true |
| emicizumab kxwh | 0 | 0 | 0 | 0 | 8 | 23,952 | 1,490 | 1,150,208 | 1.00 | 0.27 | 1.295 | 2.849 | 0.455 | 0.649 |  |  |  |  |  | true |
| oxaliplatin | 0 | 0 | 0 | 0 | 8 | 23,952 | 2,012 | 1,149,686 | 1.00 | 0.20 | 1.596 | 2.849 | 0.560 | 0.575 |  |  |  |  |  | true |

Notes: Sex-stratified PS analysis. logROR_diff = log(ROR_F) − log(ROR_M). Breslow–Day test assesses homogeneity of ORs across sex strata; Mantel–Haenszel provides a common OR. BH-FDR correction applied separately to p_diff and BD_p. Sex-specific drugs (exposure in only one sex) are flagged and not interpreted as interaction.

## Table S4b. Compact summary of sex heterogeneity results (PS analysis).

| **Parent systemic drug** | **a_F** | **b_F** | **c_F** | **d_F** | **a_M** | **b_M** | **c_M** | **d_M** | **ROR_F** | **ROR_M** | **logROR_diff** |  | **se_diff** | **z_diff** | **p_diff** | **p_diff_FDR** | **BD_p** | **BD_FDR** | **MH_OR** | **MH_p** |
| --- | --- | --- | --- | --- | --- | --- | --- | --- | --- | --- | --- | --- | --- | --- | --- | --- | --- | --- | --- | --- |
| oxybate | 49 | 42,898 | 109 | 1,396,374 | 11 | 23,949 | 58 | 1,151,640 | 14.71 | 9.45 | 0.443 |  | 0.365 | 1.211 | 0.226 | 0.226 | 0.201 | 0.201 | 13.16 | <0.001 |
| loratadine pseudoephedrine | 94 | 42,853 | 275 | 1,396,208 | 24 | 23,936 | 194 | 1,151,504 | 11.18 | 6.06 | 0.612 |  | 0.245 | 2.494 | 0.013 |  | 0.011 |  | 9.45 | <0.001 |
| trofinetide | 88 | 42,859 | 265 | 1,396,218 | 0 | 0 | 0 | 0 | 10.86 | 1.00 | 2.385 |  | 2.831 | 0.842 | 0.400 |  |  |  |  |  |
| niraparib | 364 | 42,583 | 1,134 | 1,395,349 | 0 | 0 | 0 | 0 | 10.53 | 1.00 | 2.354 |  | 2.829 | 0.832 | 0.405 |  |  |  |  |  |
| diphenhydramine naproxen | 17 | 42,930 | 57 | 1,396,426 | 0 | 0 | 0 | 0 | 9.90 | 1.00 | 2.293 |  | 2.842 | 0.807 | 0.420 |  |  |  |  |  |
| viloxazine | 15 | 42,932 | 52 | 1,396,431 | 12 | 23,948 | 49 | 1,151,649 | 9.60 | 12.14 | -0.235 |  | 0.429 | -0.547 | 0.584 |  | 0.601 |  | 10.32 | <0.001 |
| ropeginterferon alfa 2b njft | 23 | 42,924 | 91 | 1,396,392 | 8 | 23,952 | 72 | 1,151,626 | 8.36 | 5.64 | 0.394 |  | 0.430 | 0.915 | 0.360 |  | 0.325 |  | 7.21 | <0.001 |
| flibanserin | 99 | 42,848 | 422 | 1,396,061 | 0 | 0 | 0 | 0 | 7.67 | 1.00 | 2.038 |  | 2.831 | 0.720 | 0.472 |  |  |  |  |  |
| pseudoephedrine | 44 | 42,903 | 210 | 1,396,273 | 24 | 23,936 | 143 | 1,151,555 | 6.88 | 8.21 | -0.177 |  | 0.274 | -0.647 | 0.518 |  | 0.540 |  | 7.22 | <0.001 |
| suvorexant | 26 | 42,921 | 125 | 1,396,358 | 15 | 23,945 | 85 | 1,151,613 | 6.87 | 8.72 | -0.238 |  | 0.349 | -0.683 | 0.495 |  | 0.521 |  | 7.31 | <0.001 |
| eszopiclone | 21 | 42,926 | 107 | 1,396,376 | 10 | 23,950 | 68 | 1,151,630 | 6.51 | 7.37 | -0.125 |  | 0.407 | -0.307 | 0.759 |  | 0.805 |  | 6.59 | <0.001 |
| levoketoconazole | 8 | 42,939 | 44 | 1,396,439 | 0 | 0 | 0 | 0 | 6.21 | 1.00 | 1.826 |  | 2.853 | 0.640 | 0.522 |  |  |  |  |  |
| dupilumab | 869 | 42,078 | 4,652 | 1,391,831 | 605 | 23,355 | 4,244 | 1,147,454 | 6.18 | 7.01 | -0.126 |  | 0.058 | -2.179 | 0.029 |  | 0.030 |  | 6.49 | <0.001 |
| guaifenesin pseudoephedrine | 13 | 42,934 | 71 | 1,396,412 | 12 | 23,948 | 51 | 1,151,647 | 6.14 | 11.67 | -0.642 |  | 0.433 | -1.483 | 0.138 |  | 0.140 |  | 7.72 | <0.001 |
| pimavanserin | 241 | 42,706 | 1,354 | 1,395,129 | 327 | 23,633 | 1,524 | 1,150,174 | 5.82 | 10.45 | -0.585 |  | 0.093 | -6.288 | <0.001 |  | <0.001 |  | 7.82 | <0.001 |
| estrogens medroxyprogesterone | 31 | 42,916 | 188 | 1,396,295 | 0 | 0 | 0 | 0 | 5.44 | 1.00 | 1.693 |  | 2.835 | 0.597 | 0.550 |  |  |  |  |  |
| levothyroxine liothyronine | 39 | 42,908 | 238 | 1,396,245 | 0 | 0 | 0 | 0 | 5.39 | 1.00 | 1.684 |  | 2.834 | 0.594 | 0.552 |  |  |  |  |  |
| valbenazine | 98 | 42,849 | 598 | 1,395,885 | 48 | 23,912 | 511 | 1,151,187 | 5.36 | 4.56 | 0.161 |  | 0.186 | 0.866 | 0.386 |  | 0.373 |  | 5.04 | <0.001 |
| rucaparib | 113 | 42,834 | 732 | 1,395,751 | 0 | 0 | 0 | 0 | 5.05 | 1.00 | 1.619 |  | 2.830 | 0.572 | 0.567 |  |  |  |  |  |
| avapritinib | 130 | 42,817 | 861 | 1,395,622 | 92 | 23,868 | 903 | 1,150,795 | 4.94 | 4.94 | 0.000 |  | 0.144 | 0.002 | 0.999 |  | 0.990 |  | 4.92 | <0.001 |
| atovaquone proguanil | 6 | 42,941 | 44 | 1,396,439 | 0 | 0 | 0 | 0 | 4.75 | 1.00 | 1.558 |  | 2.859 | 0.545 | 0.586 |  |  |  |  |  |
| montelukast | 178 | 42,769 | 1,226 | 1,395,257 | 130 | 23,830 | 880 | 1,150,818 | 4.75 | 7.16 | -0.410 |  | 0.124 | -3.320 | <0.001 |  | <0.001 |  | 5.52 | <0.001 |
| pregabalin | 386 | 42,561 | 2,666 | 1,393,817 | 179 | 23,781 | 1,943 | 1,149,755 | 4.75 | 4.47 | 0.061 |  | 0.095 | 0.640 | 0.522 |  | 0.513 |  | 4.65 | <0.001 |
| lumateperone | 45 | 42,902 | 318 | 1,396,165 | 30 | 23,930 | 194 | 1,151,504 | 4.65 | 7.55 | -0.484 |  | 0.251 | -1.928 | 0.054 |  | 0.056 |  | 5.44 | <0.001 |
| abaloparatide | 171 | 42,776 | 1,209 | 1,395,274 | 9 | 23,951 | 216 | 1,151,482 | 4.63 | 2.11 | 0.785 |  | 0.341 | 2.299 | 0.022 |  | 0.014 |  | 4.33 | <0.001 |
| bupropion naltrexone | 87 | 42,860 | 639 | 1,395,844 | 12 | 23,948 | 117 | 1,151,581 | 4.46 | 5.12 | -0.138 |  | 0.319 | -0.433 | 0.665 |  | 0.742 |  | 4.49 | <0.001 |
| estrogens | 47 | 42,900 | 348 | 1,396,135 | 0 | 0 | 0 | 0 | 4.44 | 1.00 | 1.490 |  | 2.833 | 0.526 | 0.599 |  |  |  |  |  |
| carbidopa levodopa | 93 | 42,854 | 709 | 1,395,774 | 73 | 23,887 | 663 | 1,151,035 | 4.29 | 5.34 | -0.218 |  | 0.165 | -1.320 | 0.187 |  | 0.190 |  | 4.67 | <0.001 |
| vilazodone | 24 | 42,923 | 189 | 1,396,294 | 12 | 23,948 | 80 | 1,151,618 | 4.21 | 7.47 | -0.574 |  | 0.372 | -1.542 | 0.123 |  | 0.136 |  | 4.82 | <0.001 |
| elagolix | 77 | 42,870 | 609 | 1,395,874 | 0 | 0 | 0 | 0 | 4.14 | 1.00 | 1.421 |  | 2.831 | 0.502 | 0.616 |  |  |  |  |  |
| omalizumab | 504 | 42,443 | 4,047 | 1,392,436 | 239 | 23,721 | 2,150 | 1,149,548 | 4.09 | 5.40 | -0.277 |  | 0.083 | -3.332 | <0.001 |  | <0.001 |  | 4.43 | <0.001 |
| corticotropin | 88 | 42,859 | 708 | 1,395,775 | 46 | 23,914 | 402 | 1,151,296 | 4.07 | 5.56 | -0.313 |  | 0.192 | -1.631 | 0.103 |  | 0.108 |  | 4.46 | <0.001 |
| amphetamine aspartate amphetamine dextroamphetamine saccharate dextroamphetamine | 79 | 42,868 | 639 | 1,395,844 | 32 | 23,928 | 475 | 1,151,223 | 4.05 | 3.29 | 0.208 |  | 0.217 | 0.958 | 0.338 |  | 0.320 |  | 3.76 | <0.001 |
| levothyroxine | 291 | 42,656 | 2,362 | 1,394,121 | 42 | 23,918 | 679 | 1,151,019 | 4.03 | 3.01 | 0.293 |  | 0.170 | 1.720 | 0.085 |  | 0.076 |  | 3.85 | <0.001 |
| ofloxacin | 8 | 42,939 | 69 | 1,396,414 | 0 | 0 | 0 | 0 | 3.98 | 1.00 | 1.381 |  | 2.852 | 0.484 | 0.628 |  |  |  |  |  |
| phentermine topiramate | 56 | 42,891 | 466 | 1,396,017 | 17 | 23,943 | 85 | 1,151,613 | 3.94 | 9.84 | -0.915 |  | 0.298 | -3.072 | 0.002 |  | 0.002 |  | 4.54 | <0.001 |
| lurasidone | 136 | 42,811 | 1,153 | 1,395,330 | 90 | 23,870 | 688 | 1,151,010 | 3.86 | 6.34 | -0.497 |  | 0.144 | -3.447 | <0.001 |  | <0.001 |  | 4.56 | <0.001 |
| desvenlafaxine | 50 | 42,897 | 430 | 1,396,053 | 10 | 23,950 | 126 | 1,151,572 | 3.82 | 3.99 | -0.044 |  | 0.354 | -0.125 | 0.900 |  | 0.981 |  | 3.79 | <0.001 |
| naltrexone | 144 | 42,803 | 1,248 | 1,395,235 | 200 | 23,760 | 1,415 | 1,150,283 | 3.77 | 6.86 | -0.598 |  | 0.116 | -5.146 | <0.001 |  | <0.001 |  | 5.10 | <0.001 |
| amantadine | 31 | 42,916 | 273 | 1,396,210 | 23 | 23,937 | 233 | 1,151,465 | 3.75 | 4.84 | -0.256 |  | 0.287 | -0.893 | 0.372 |  | 0.384 |  | 4.08 | <0.001 |
| mifepristone | 23 | 42,924 | 208 | 1,396,275 | 5 | 23,955 | 55 | 1,151,643 | 3.67 | 4.76 | -0.262 |  | 0.497 | -0.527 | 0.598 |  | 0.705 |  | 3.72 | <0.001 |
| milnacipran | 8 | 42,939 | 75 | 1,396,408 | 0 | 0 | 0 | 0 | 3.66 | 1.00 | 1.298 |  | 2.851 | 0.455 | 0.649 |  |  |  |  |  |
| elacestrant | 55 | 42,892 | 494 | 1,395,989 | 0 | 0 | 0 | 0 | 3.65 | 1.00 | 1.295 |  | 2.832 | 0.457 | 0.647 |  |  |  |  |  |
| cetirizine pseudoephedrine | 7 | 42,940 | 67 | 1,396,416 | 0 | 0 | 0 | 0 | 3.61 | 1.00 | 1.285 |  | 2.855 | 0.450 | 0.653 |  |  |  |  |  |
| estradiol | 107 | 42,840 | 971 | 1,395,512 | 0 | 0 | 0 | 0 | 3.60 | 1.00 | 1.282 |  | 2.830 | 0.453 | 0.651 |  |  |  |  |  |
| dalfampridine | 170 | 42,777 | 1,554 | 1,394,929 | 55 | 23,905 | 945 | 1,150,753 | 3.58 | 2.83 | 0.236 |  | 0.160 | 1.472 | 0.141 |  | 0.132 |  | 3.34 | <0.001 |
| clonazepam | 96 | 42,851 | 882 | 1,395,601 | 46 | 23,914 | 698 | 1,151,000 | 3.56 | 3.20 | 0.106 |  | 0.186 | 0.569 | 0.569 |  | 0.551 |  | 3.41 | <0.001 |
| olanzapine samidorphan l | 21 | 42,926 | 198 | 1,396,285 | 19 | 23,941 | 160 | 1,151,538 | 3.52 | 5.84 | -0.506 |  | 0.330 | -1.532 | 0.126 |  | 0.128 |  | 4.25 | <0.001 |
| diphenhydramine ibuprofen | 5 | 42,942 | 51 | 1,396,432 | 0 | 0 | 0 | 0 | 3.47 | 1.00 | 1.245 |  | 2.864 | 0.435 | 0.664 |  |  |  |  |  |
| sofosbuvir velpatasvir | 122 | 42,825 | 1,164 | 1,395,319 | 133 | 23,827 | 1,377 | 1,150,321 | 3.43 | 4.68 | -0.311 |  | 0.132 | -2.366 | 0.018 |  | 0.018 |  | 3.97 | <0.001 |
| deutetrabenazine | 59 | 42,888 | 587 | 1,395,896 | 31 | 23,929 | 362 | 1,151,336 | 3.30 | 4.18 | -0.238 |  | 0.230 | -1.032 | 0.302 |  | 0.318 |  | 3.52 | <0.001 |
| cariprazine | 46 | 42,901 | 460 | 1,396,023 | 22 | 23,938 | 318 | 1,151,380 | 3.29 | 3.40 | -0.034 |  | 0.267 | -0.125 | 0.900 |  | 0.934 |  | 3.28 | <0.001 |
| fezolinetant | 14 | 42,933 | 144 | 1,396,339 | 0 | 0 | 0 | 0 | 3.26 | 1.00 | 1.183 |  | 2.842 | 0.416 | 0.677 |  |  |  |  |  |
| patisiran | 9 | 42,938 | 95 | 1,396,388 | 8 | 23,952 | 150 | 1,151,548 | 3.24 | 2.72 | 0.175 |  | 0.490 | 0.357 | 0.721 |  | 0.715 |  | 2.81 | <0.001 |
| bazedoxifene estrogens | 7 | 42,940 | 75 | 1,396,408 | 0 | 0 | 0 | 0 | 3.23 | 1.00 | 1.173 |  | 2.854 | 0.411 | 0.681 |  |  |  |  |  |
| ciprofloxacin | 175 | 42,772 | 1,777 | 1,394,706 | 119 | 23,841 | 1,634 | 1,150,064 | 3.22 | 3.53 | -0.091 |  | 0.124 | -0.737 | 0.461 |  | 0.468 |  | 3.33 | <0.001 |
| ocrelizumab | 414 | 42,533 | 4,229 | 1,392,254 | 126 | 23,834 | 2,500 | 1,149,198 | 3.21 | 2.44 | 0.274 |  | 0.105 | 2.609 | 0.009 |  | 0.008 |  | 2.98 | <0.001 |
| phentermine | 7 | 42,940 | 77 | 1,396,406 | 0 | 0 | 0 | 0 | 3.15 | 1.00 | 1.146 |  | 2.854 | 0.402 | 0.688 |  |  |  |  |  |
| estradiol progesterone | 9 | 42,938 | 98 | 1,396,385 | 0 | 0 | 0 | 0 | 3.14 | 1.00 | 1.143 |  | 2.849 | 0.401 | 0.688 |  |  |  |  |  |
| ciclesonide | 30 | 42,917 | 316 | 1,396,167 | 6 | 23,954 | 187 | 1,151,511 | 3.13 | 1.67 | 0.632 |  | 0.442 | 1.430 | 0.153 |  | 0.122 |  | 2.64 | <0.001 |
| thyroid porcine | 21 | 42,926 | 227 | 1,396,256 | 0 | 0 | 0 | 0 | 3.07 | 1.00 | 1.123 |  | 2.837 | 0.396 | 0.692 |  |  |  |  |  |
| roflumilast | 18 | 42,929 | 196 | 1,396,287 | 14 | 23,946 | 200 | 1,151,498 | 3.06 | 3.48 | -0.127 |  | 0.365 | -0.349 | 0.727 |  | 0.747 |  | 3.14 | <0.001 |
| teriflunomide | 233 | 42,714 | 2,523 | 1,393,960 | 48 | 23,912 | 1,247 | 1,150,451 | 3.02 | 1.87 | 0.479 |  | 0.162 | 2.961 | 0.003 |  | 0.003 |  | 2.72 | <0.001 |
| lenalidomide | 544 | 42,403 | 5,957 | 1,390,526 | 501 | 23,459 | 6,632 | 1,145,066 | 3.00 | 3.69 | -0.208 |  | 0.065 | -3.205 | 0.001 |  | 0.001 |  | 3.29 | <0.001 |
| varenicline | 67 | 42,880 | 735 | 1,395,748 | 34 | 23,926 | 492 | 1,151,206 | 2.99 | 3.37 | -0.121 |  | 0.217 | -0.555 | 0.579 |  | 0.602 |  | 3.08 | <0.001 |
| apremilast | 268 | 42,679 | 2,950 | 1,393,533 | 138 | 23,822 | 2,543 | 1,149,155 | 2.97 | 2.63 | 0.123 |  | 0.108 | 1.138 | 0.255 |  | 0.249 |  | 2.84 | <0.001 |
| nirmatrelvir ritonavir | 124 | 42,823 | 1,386 | 1,395,097 | 55 | 23,905 | 1,146 | 1,150,552 | 2.93 | 2.33 | 0.228 |  | 0.166 | 1.367 | 0.172 |  | 0.163 |  | 2.70 | <0.001 |
| elexacaftor ivacaftor tezacaftor | 135 | 42,812 | 1,541 | 1,394,942 | 96 | 23,864 | 1,309 | 1,150,389 | 2.86 | 3.55 | -0.215 |  | 0.139 | -1.553 | 0.120 |  | 0.123 |  | 3.10 | <0.001 |
| nebivolol | 20 | 42,927 | 233 | 1,396,250 | 0 | 0 | 0 | 0 | 2.86 | 1.00 | 1.049 |  | 2.838 | 0.370 | 0.712 |  |  |  |  |  |
| amphetamine aspartate amphetamine aspartate amphetamine dextroamphetamine saccharate dextroamphetamine | 7 | 42,940 | 85 | 1,396,398 | 0 | 0 | 0 | 0 | 2.85 | 1.00 | 1.048 |  | 2.854 | 0.367 | 0.713 |  |  |  |  |  |
| ofatumumab | 192 | 42,755 | 2,206 | 1,394,277 | 79 | 23,881 | 1,208 | 1,150,490 | 2.85 | 3.17 | -0.108 |  | 0.138 | -0.780 | 0.435 |  | 0.451 |  | 2.92 | <0.001 |
| dichlorphenamide | 24 | 42,923 | 281 | 1,396,202 | 15 | 23,945 | 251 | 1,151,447 | 2.83 | 2.96 | -0.046 |  | 0.336 | -0.136 | 0.892 |  | 0.921 |  | 2.81 | <0.001 |
| tirzepatide | 77 | 42,870 | 894 | 1,395,589 | 33 | 23,927 | 740 | 1,150,958 | 2.82 | 2.18 | 0.259 |  | 0.213 | 1.219 | 0.223 |  | 0.210 |  | 2.57 | <0.001 |
| sofosbuvir | 6 | 42,941 | 75 | 1,396,408 | 10 | 23,950 | 68 | 1,151,630 | 2.80 | 7.37 | -0.968 |  | 0.526 | -1.839 | 0.066 |  | 0.057 |  | 4.31 | <0.001 |
| treprostinil | 207 | 42,740 | 2,418 | 1,394,065 | 54 | 23,906 | 1,373 | 1,150,325 | 2.80 | 1.91 | 0.382 |  | 0.156 | 2.449 | 0.014 |  | 0.013 |  | 2.54 | <0.001 |
| sacubitril valsartan | 275 | 42,672 | 3,263 | 1,393,220 | 292 | 23,668 | 4,525 | 1,147,173 | 2.76 | 3.13 | -0.128 |  | 0.087 | -1.465 | 0.143 |  | 0.143 |  | 2.93 | <0.001 |
| ziprasidone | 7 | 42,940 | 88 | 1,396,395 | 0 | 0 | 0 | 0 | 2.76 | 1.00 | 1.014 |  | 2.854 | 0.355 | 0.722 |  |  |  |  |  |
| fexofenadine | 30 | 42,917 | 361 | 1,396,122 | 8 | 23,952 | 222 | 1,151,476 | 2.74 | 1.84 | 0.402 |  | 0.397 | 1.012 | 0.312 |  | 0.271 |  | 2.42 | <0.001 |
| levofloxacin | 110 | 42,837 | 1,354 | 1,395,129 | 59 | 23,901 | 1,184 | 1,150,514 | 2.66 | 2.42 | 0.094 |  | 0.166 | 0.568 | 0.570 |  | 0.556 |  | 2.55 | <0.001 |
| tofacitinib | 453 | 42,494 | 5,588 | 1,390,895 | 131 | 23,829 | 3,282 | 1,148,416 | 2.66 | 1.93 | 0.319 |  | 0.102 | 3.134 | 0.002 |  | 0.002 |  | 2.44 | <0.001 |
| duloxetine | 111 | 42,836 | 1,368 | 1,395,115 | 45 | 23,915 | 662 | 1,151,036 | 2.65 | 3.31 | -0.220 |  | 0.182 | -1.205 | 0.228 |  | 0.243 |  | 2.80 | <0.001 |
| atomoxetine | 11 | 42,936 | 141 | 1,396,342 | 11 | 23,949 | 202 | 1,151,496 | 2.64 | 2.73 | -0.033 |  | 0.431 | -0.075 | 0.940 |  | 0.943 |  | 2.58 | <0.001 |
| selexipag | 185 | 42,762 | 2,294 | 1,394,189 | 68 | 23,892 | 1,242 | 1,150,456 | 2.64 | 2.65 | -0.007 |  | 0.146 | -0.049 | 0.961 |  | 0.985 |  | 2.63 | <0.001 |
| hydroxyprogesterone caproate | 64 | 42,883 | 800 | 1,395,683 | 0 | 0 | 0 | 0 | 2.62 | 1.00 | 0.964 |  | 2.831 | 0.340 | 0.733 |  |  |  |  |  |
| nortriptyline | 7 | 42,940 | 95 | 1,396,388 | 0 | 0 | 0 | 0 | 2.55 | 1.00 | 0.938 |  | 2.854 | 0.329 | 0.742 |  |  |  |  |  |
| alemtuzumab | 119 | 42,828 | 1,537 | 1,394,946 | 32 | 23,928 | 748 | 1,150,950 | 2.53 | 2.09 | 0.192 |  | 0.203 | 0.948 | 0.343 |  | 0.319 |  | 2.41 | <0.001 |
| bupropion | 79 | 42,868 | 1,032 | 1,395,451 | 40 | 23,920 | 618 | 1,151,080 | 2.51 | 3.15 | -0.229 |  | 0.200 | -1.145 | 0.252 |  | 0.266 |  | 2.67 | <0.001 |
| hydrocodone | 11 | 42,936 | 150 | 1,396,333 | 16 | 23,944 | 167 | 1,151,531 | 2.48 | 4.74 | -0.645 |  | 0.400 | -1.612 | 0.107 |  | 0.101 |  | 3.34 | <0.001 |
| dextromethorphan guaifenesin | 30 | 42,917 | 405 | 1,396,078 | 14 | 23,946 | 269 | 1,151,429 | 2.45 | 2.59 | -0.056 |  | 0.329 | -0.170 | 0.865 |  | 0.910 |  | 2.44 | <0.001 |
| clarithromycin | 52 | 42,895 | 700 | 1,395,783 | 19 | 23,941 | 420 | 1,151,278 | 2.44 | 2.23 | 0.089 |  | 0.272 | 0.328 | 0.743 |  | 0.702 |  | 2.35 | <0.001 |
| triamcinolone acetonide | 11 | 42,936 | 157 | 1,396,326 | 0 | 0 | 0 | 0 | 2.37 | 1.00 | 0.865 |  | 2.845 | 0.304 | 0.761 |  |  |  |  |  |
| ledipasvir sofosbuvir | 34 | 42,913 | 473 | 1,396,010 | 36 | 23,924 | 480 | 1,151,218 | 2.37 | 3.66 | -0.433 |  | 0.246 | -1.759 | 0.079 |  | 0.078 |  | 2.86 | <0.001 |
| pyrimethamine | 5 | 42,942 | 75 | 1,396,408 | 5 | 23,955 | 95 | 1,151,603 | 2.37 | 2.77 | -0.156 |  | 0.622 | -0.251 | 0.802 |  | 0.812 |  | 2.34 | 0.007 |
| amphetamine dextroamphetamine | 5 | 42,942 | 76 | 1,396,407 | 0 | 0 | 0 | 0 | 2.34 | 1.00 | 0.849 |  | 2.863 | 0.297 | 0.767 |  |  |  |  |  |
| mitapivat | 5 | 42,942 | 76 | 1,396,407 | 0 | 0 | 0 | 0 | 2.34 | 1.00 | 0.849 |  | 2.863 | 0.297 | 0.767 |  |  |  |  |  |
| ombitasvir paritaprevir ritonavir | 6 | 42,941 | 90 | 1,396,393 | 0 | 0 | 0 | 0 | 2.34 | 1.00 | 0.848 |  | 2.857 | 0.297 | 0.767 |  |  |  |  |  |
| nicotine | 33 | 42,914 | 474 | 1,396,009 | 19 | 23,941 | 404 | 1,151,294 | 2.30 | 2.32 | -0.009 |  | 0.293 | -0.032 | 0.975 |  | 0.996 |  | 2.26 | <0.001 |
| water | 7 | 42,940 | 106 | 1,396,377 | 5 | 23,955 | 96 | 1,151,602 | 2.29 | 2.74 | -0.179 |  | 0.579 | -0.310 | 0.757 |  | 0.799 |  | 2.28 | 0.004 |
| nirogacestat | 6 | 42,941 | 93 | 1,396,390 | 0 | 0 | 0 | 0 | 2.26 | 1.00 | 0.816 |  | 2.857 | 0.285 | 0.775 |  |  |  |  |  |
| palbociclib | 353 | 42,594 | 5,114 | 1,391,369 | 7 | 23,953 | 498 | 1,151,200 | 2.26 | 0.72 | 1.139 |  | 0.372 | 3.060 | 0.002 |  | <0.001 |  | 2.16 | <0.001 |
| lasmiditan | 5 | 42,942 | 79 | 1,396,404 | 0 | 0 | 0 | 0 | 2.25 | 1.00 | 0.811 |  | 2.863 | 0.283 | 0.777 |  |  |  |  |  |
| cetirizine | 78 | 42,869 | 1,145 | 1,395,338 | 35 | 23,925 | 735 | 1,150,963 | 2.23 | 2.32 | -0.040 |  | 0.208 | -0.193 | 0.847 |  | 0.876 |  | 2.24 | <0.001 |
| oxybate | 17 | 42,930 | 255 | 1,396,228 | 6 | 23,954 | 192 | 1,151,506 | 2.23 | 1.62 | 0.317 |  | 0.469 | 0.675 | 0.500 |  | 0.447 |  | 1.94 | 0.002 |
| acetaminophen hydrocodone | 36 | 42,911 | 541 | 1,395,942 | 34 | 23,926 | 481 | 1,151,217 | 2.19 | 3.45 | -0.452 |  | 0.246 | -1.842 | 0.066 |  | 0.066 |  | 2.63 | <0.001 |
| galcanezumab gnlm | 64 | 42,883 | 961 | 1,395,522 | 15 | 23,945 | 326 | 1,151,372 | 2.18 | 2.28 | -0.045 |  | 0.290 | -0.154 | 0.878 |  | 0.944 |  | 2.18 | <0.001 |
| solriamfetol | 5 | 42,942 | 82 | 1,396,401 | 5 | 23,955 | 62 | 1,151,636 | 2.17 | 4.23 | -0.669 |  | 0.626 | -1.068 | 0.285 |  | 0.298 |  | 2.63 | 0.002 |
| pirfenidone | 90 | 42,857 | 1,360 | 1,395,123 | 126 | 23,834 | 1,866 | 1,149,832 | 2.17 | 3.27 | -0.412 |  | 0.142 | -2.893 | 0.004 |  | 0.004 |  | 2.69 | <0.001 |
| gabapentin | 94 | 42,853 | 1,429 | 1,395,054 | 38 | 23,922 | 1,016 | 1,150,682 | 2.15 | 1.82 | 0.167 |  | 0.196 | 0.851 | 0.395 |  | 0.375 |  | 2.03 | <0.001 |
| fingolimod | 338 | 42,609 | 5,164 | 1,391,319 | 90 | 23,870 | 2,348 | 1,149,350 | 2.14 | 1.86 | 0.143 |  | 0.121 | 1.178 | 0.239 |  | 0.227 |  | 2.07 | <0.001 |
| alendronate | 84 | 42,863 | 1,292 | 1,395,191 | 7 | 23,953 | 226 | 1,151,472 | 2.13 | 1.59 | 0.290 |  | 0.388 | 0.749 | 0.454 |  | 0.377 |  | 2.05 | <0.001 |
| leflunomide | 45 | 42,902 | 696 | 1,395,787 | 13 | 23,947 | 244 | 1,151,454 | 2.13 | 2.65 | -0.222 |  | 0.319 | -0.698 | 0.485 |  | 0.542 |  | 2.19 | <0.001 |
| sofosbuvir velpatasvir voxilaprevir | 6 | 42,941 | 99 | 1,396,384 | 5 | 23,955 | 179 | 1,151,519 | 2.12 | 1.47 | 0.366 |  | 0.593 | 0.618 | 0.537 |  | 0.533 |  | 1.62 | 0.112 |
| aripiprazole | 104 | 42,843 | 1,606 | 1,394,877 | 65 | 23,895 | 1,365 | 1,150,333 | 2.12 | 2.31 | -0.087 |  | 0.162 | -0.534 | 0.593 |  | 0.606 |  | 2.18 | <0.001 |
| lisdexamfetamine dimesylate | 33 | 42,914 | 519 | 1,395,964 | 22 | 23,938 | 433 | 1,151,265 | 2.10 | 2.50 | -0.174 |  | 0.280 | -0.620 | 0.535 |  | 0.555 |  | 2.20 | <0.001 |
| pexidartinib | 13 | 42,934 | 209 | 1,396,274 | 7 | 23,953 | 144 | 1,151,554 | 2.10 | 2.50 | -0.175 |  | 0.468 | -0.373 | 0.709 |  | 0.764 |  | 2.12 | <0.001 |
| propranolol | 37 | 42,910 | 594 | 1,395,889 | 13 | 23,947 | 348 | 1,151,350 | 2.05 | 1.86 | 0.097 |  | 0.325 | 0.299 | 0.765 |  | 0.714 |  | 1.96 | <0.001 |
| loratadine | 61 | 42,886 | 981 | 1,395,502 | 24 | 23,936 | 816 | 1,150,882 | 2.04 | 1.44 | 0.346 |  | 0.244 | 1.419 | 0.156 |  | 0.143 |  | 1.80 | <0.001 |
| bupropion dextromethorphan | 11 | 42,936 | 184 | 1,396,299 | 11 | 23,949 | 118 | 1,151,580 | 2.03 | 4.67 | -0.834 |  | 0.433 | -1.924 | 0.054 |  | 0.053 |  | 2.72 | <0.001 |
| venlafaxine | 107 | 42,840 | 1,728 | 1,394,755 | 34 | 23,926 | 909 | 1,150,789 | 2.02 | 1.82 | 0.104 |  | 0.200 | 0.521 | 0.603 |  | 0.571 |  | 1.96 | <0.001 |
| chlorhexidine | 7 | 42,940 | 121 | 1,396,362 | 0 | 0 | 0 | 0 | 2.01 | 1.00 | 0.697 |  | 2.853 | 0.244 | 0.807 |  |  |  |  |  |
| oseltamivir | 31 | 42,916 | 511 | 1,395,972 | 20 | 23,940 | 409 | 1,151,289 | 2.00 | 2.41 | -0.184 |  | 0.292 | -0.631 | 0.528 |  | 0.551 |  | 2.11 | <0.001 |
| erenumab aooe | 86 | 42,861 | 1,417 | 1,395,066 | 5 | 23,955 | 403 | 1,151,295 | 1.99 | 0.66 | 1.109 |  | 0.443 | 2.501 | 0.012 |  | 0.006 |  | 1.75 | <0.001 |
| sucralfate | 6 | 42,941 | 106 | 1,396,377 | 0 | 0 | 0 | 0 | 1.98 | 1.00 | 0.685 |  | 2.857 | 0.240 | 0.810 |  |  |  |  |  |
| sertraline | 137 | 42,810 | 2,293 | 1,394,190 | 60 | 23,900 | 1,320 | 1,150,378 | 1.95 | 2.21 | -0.122 |  | 0.158 | -0.769 | 0.442 |  | 0.460 |  | 2.01 | <0.001 |
| methylphenidate | 57 | 42,890 | 968 | 1,395,515 | 104 | 23,856 | 1,231 | 1,150,467 | 1.93 | 4.09 | -0.751 |  | 0.170 | -4.418 | <0.001 |  | <0.001 |  | 2.92 | <0.001 |
| guaifenesin | 15 | 42,932 | 261 | 1,396,222 | 10 | 23,950 | 188 | 1,151,510 | 1.93 | 2.68 | -0.329 |  | 0.411 | -0.800 | 0.424 |  | 0.453 |  | 2.10 | <0.001 |
| budesonide | 103 | 42,844 | 1,750 | 1,394,733 | 76 | 23,884 | 1,494 | 1,150,204 | 1.92 | 2.47 | -0.247 |  | 0.155 | -1.596 | 0.111 |  | 0.113 |  | 2.11 | <0.001 |
| voclosporin | 45 | 42,902 | 773 | 1,395,710 | 11 | 23,949 | 294 | 1,151,404 | 1.91 | 1.88 | 0.019 |  | 0.337 | 0.057 | 0.955 |  | 0.881 |  | 1.87 | <0.001 |
| ripretinib | 26 | 42,921 | 450 | 1,396,033 | 28 | 23,932 | 551 | 1,151,147 | 1.91 | 2.49 | -0.262 |  | 0.277 | -0.944 | 0.345 |  | 0.346 |  | 2.14 | <0.001 |
| escitalopram | 76 | 42,871 | 1,316 | 1,395,167 | 57 | 23,903 | 781 | 1,150,917 | 1.89 | 3.54 | -0.628 |  | 0.180 | -3.478 | <0.001 |  | <0.001 |  | 2.35 | <0.001 |
| paliperidone | 24 | 42,923 | 422 | 1,396,061 | 18 | 23,942 | 584 | 1,151,114 | 1.89 | 1.52 | 0.215 |  | 0.315 | 0.682 | 0.495 |  | 0.485 |  | 1.67 | 0.001 |
| oxycodone | 146 | 42,801 | 2,541 | 1,393,942 | 201 | 23,759 | 3,295 | 1,148,403 | 1.88 | 2.96 | -0.454 |  | 0.112 | -4.050 | <0.001 |  | <0.001 |  | 2.38 | <0.001 |
| citalopram | 53 | 42,894 | 930 | 1,395,553 | 29 | 23,931 | 512 | 1,151,186 | 1.87 | 2.77 | -0.392 |  | 0.236 | -1.662 | 0.097 |  | 0.103 |  | 2.09 | <0.001 |
| macitentan | 252 | 42,695 | 4,410 | 1,392,073 | 61 | 23,899 | 2,325 | 1,149,373 | 1.87 | 1.27 | 0.384 |  | 0.145 | 2.651 | 0.008 |  | 0.007 |  | 1.70 | <0.001 |
| hydromorphone | 27 | 42,920 | 482 | 1,396,001 | 23 | 23,937 | 437 | 1,151,261 | 1.85 | 2.58 | -0.332 |  | 0.289 | -1.150 | 0.250 |  | 0.257 |  | 2.09 | <0.001 |
| progesterone | 12 | 42,935 | 219 | 1,396,264 | 0 | 0 | 0 | 0 | 1.85 | 1.00 | 0.616 |  | 2.843 | 0.217 | 0.828 |  |  |  |  |  |
| rosuvastatin | 82 | 42,865 | 1,461 | 1,395,022 | 75 | 23,885 | 1,549 | 1,150,149 | 1.84 | 2.35 | -0.245 |  | 0.164 | -1.495 | 0.135 |  | 0.136 |  | 2.04 | <0.001 |
| bremelanotide | 5 | 42,942 | 97 | 1,396,386 | 0 | 0 | 0 | 0 | 1.83 | 1.00 | 0.607 |  | 2.862 | 0.212 | 0.832 |  |  |  |  |  |
| prucalopride | 5 | 42,942 | 98 | 1,396,385 | 0 | 0 | 0 | 0 | 1.82 | 1.00 | 0.596 |  | 2.862 | 0.208 | 0.835 |  |  |  |  |  |
| bismuth subcitrate metronidazole tetracycline | 5 | 42,942 | 99 | 1,396,384 | 0 | 0 | 0 | 0 | 1.80 | 1.00 | 0.586 |  | 2.862 | 0.205 | 0.838 |  |  |  |  |  |
| inotersen | 9 | 42,938 | 172 | 1,396,311 | 0 | 0 | 0 | 0 | 1.79 | 1.00 | 0.583 |  | 2.848 | 0.205 | 0.838 |  |  |  |  |  |
| brexpiprazole | 20 | 42,927 | 373 | 1,396,110 | 10 | 23,950 | 246 | 1,151,452 | 1.79 | 2.05 | -0.137 |  | 0.388 | -0.354 | 0.724 |  | 0.773 |  | 1.81 | 0.001 |
| pramipexole | 5 | 42,942 | 100 | 1,396,383 | 0 | 0 | 0 | 0 | 1.78 | 1.00 | 0.576 |  | 2.862 | 0.201 | 0.840 |  |  |  |  |  |
| belimumab | 88 | 42,859 | 1,620 | 1,394,863 | 0 | 0 | 0 | 0 | 1.78 | 1.00 | 0.575 |  | 2.831 | 0.203 | 0.839 |  |  |  |  |  |
| vonoprazan | 11 | 42,936 | 210 | 1,396,273 | 0 | 0 | 0 | 0 | 1.78 | 1.00 | 0.575 |  | 2.845 | 0.202 | 0.840 |  |  |  |  |  |
| anastrozole | 61 | 42,886 | 1,134 | 1,395,349 | 0 | 0 | 0 | 0 | 1.76 | 1.00 | 0.567 |  | 2.831 | 0.200 | 0.841 |  |  |  |  |  |
| octreotide | 116 | 42,831 | 2,174 | 1,394,309 | 82 | 23,878 | 1,991 | 1,149,707 | 1.74 | 1.99 | -0.134 |  | 0.147 | -0.910 | 0.363 |  | 0.370 |  | 1.83 | <0.001 |
| semaglutide | 171 | 42,776 | 3,203 | 1,393,280 | 96 | 23,864 | 2,796 | 1,148,902 | 1.74 | 1.66 | 0.048 |  | 0.130 | 0.372 | 0.710 |  | 0.698 |  | 1.71 | <0.001 |
| adalimumab | 810 | 42,137 | 15,262 | 1,381,221 | 455 | 23,505 | 11,918 | 1,139,780 | 1.74 | 1.85 | -0.063 |  | 0.060 | -1.037 | 0.300 |  | 0.303 |  | 1.78 | <0.001 |
| atogepant | 26 | 42,921 | 496 | 1,395,987 | 11 | 23,949 | 116 | 1,151,582 | 1.74 | 4.75 | -1.006 |  | 0.368 | -2.734 | 0.006 |  | 0.006 |  | 2.10 | <0.001 |
| liothyronine | 7 | 42,940 | 143 | 1,396,340 | 0 | 0 | 0 | 0 | 1.70 | 1.00 | 0.530 |  | 2.853 | 0.186 | 0.853 |  |  |  |  |  |
| vortioxetine | 43 | 42,904 | 835 | 1,395,648 | 27 | 23,933 | 463 | 1,151,235 | 1.69 | 2.85 | -0.522 |  | 0.251 | -2.083 | 0.037 |  | 0.039 |  | 1.99 | <0.001 |
| nintedanib | 96 | 42,851 | 1,869 | 1,394,614 | 115 | 23,845 | 2,510 | 1,149,188 | 1.68 | 2.22 | -0.277 |  | 0.141 | -1.961 | 0.050 |  | 0.049 |  | 1.93 | <0.001 |
| fenfluramine | 16 | 42,931 | 321 | 1,396,162 | 24 | 23,936 | 295 | 1,151,403 | 1.67 | 3.99 | -0.871 |  | 0.329 | -2.651 | 0.008 |  | 0.006 |  | 2.50 | <0.001 |
| famotidine | 16 | 42,931 | 327 | 1,396,156 | 0 | 0 | 0 | 0 | 1.64 | 1.00 | 0.494 |  | 2.840 | 0.174 | 0.862 |  |  |  |  |  |
| budesonide formoterol dihydrate | 94 | 42,853 | 1,877 | 1,394,606 | 58 | 23,902 | 1,347 | 1,150,351 | 1.64 | 2.09 | -0.243 |  | 0.170 | -1.429 | 0.153 |  | 0.159 |  | 1.78 | <0.001 |
| tazemetostat | 7 | 42,940 | 149 | 1,396,334 | 0 | 0 | 0 | 0 | 1.63 | 1.00 | 0.489 |  | 2.853 | 0.172 | 0.864 |  |  |  |  |  |
| amitriptyline | 15 | 42,932 | 309 | 1,396,174 | 0 | 0 | 0 | 0 | 1.63 | 1.00 | 0.488 |  | 2.840 | 0.172 | 0.864 |  |  |  |  |  |
| inclisiran | 30 | 42,917 | 609 | 1,395,874 | 20 | 23,940 | 580 | 1,151,118 | 1.63 | 1.70 | -0.042 |  | 0.292 | -0.145 | 0.884 |  | 0.907 |  | 1.62 | <0.001 |
| adalimumab afzb | 20 | 42,927 | 411 | 1,396,072 | 11 | 23,949 | 297 | 1,151,401 | 1.62 | 1.86 | -0.137 |  | 0.376 | -0.365 | 0.715 |  | 0.758 |  | 1.65 | 0.006 |
| diroximel | 18 | 42,929 | 374 | 1,396,109 | 0 | 0 | 0 | 0 | 1.61 | 1.00 | 0.474 |  | 2.838 | 0.167 | 0.867 |  |  |  |  |  |
| brivaracetam | 22 | 42,925 | 458 | 1,396,025 | 11 | 23,949 | 393 | 1,151,305 | 1.60 | 1.40 | 0.128 |  | 0.369 | 0.346 | 0.730 |  | 0.691 |  | 1.48 | 0.026 |
| secukinumab | 295 | 42,652 | 6,079 | 1,390,404 | 211 | 23,749 | 5,636 | 1,146,062 | 1.58 | 1.81 | -0.133 |  | 0.092 | -1.446 | 0.148 |  | 0.150 |  | 1.67 | <0.001 |
| acyclovir | 28 | 42,919 | 587 | 1,395,896 | 0 | 0 | 0 | 0 | 1.58 | 1.00 | 0.456 |  | 2.835 | 0.161 | 0.872 |  |  |  |  |  |
| cannabidiol | 25 | 42,922 | 529 | 1,395,954 | 21 | 23,939 | 618 | 1,151,080 | 1.57 | 1.67 | -0.065 |  | 0.299 | -0.218 | 0.828 |  | 0.840 |  | 1.58 | 0.002 |
| fluticasone | 6 | 42,941 | 135 | 1,396,348 | 0 | 0 | 0 | 0 | 1.56 | 1.00 | 0.445 |  | 2.857 | 0.156 | 0.876 |  |  |  |  |  |
| fremanezumab vfrm | 38 | 42,909 | 803 | 1,395,680 | 0 | 0 | 0 | 0 | 1.56 | 1.00 | 0.444 |  | 2.833 | 0.157 | 0.876 |  |  |  |  |  |
| ethinyl estradiol norethindrone | 14 | 42,933 | 303 | 1,396,180 | 0 | 0 | 0 | 0 | 1.55 | 1.00 | 0.441 |  | 2.841 | 0.155 | 0.877 |  |  |  |  |  |
| guanfacine | 8 | 42,939 | 178 | 1,396,305 | 6 | 23,954 | 238 | 1,151,460 | 1.55 | 1.31 | 0.167 |  | 0.530 | 0.315 | 0.753 |  | 0.733 |  | 1.34 | 0.277 |
| cyclobenzaprine | 6 | 42,941 | 136 | 1,396,347 | 0 | 0 | 0 | 0 | 1.55 | 1.00 | 0.437 |  | 2.857 | 0.153 | 0.878 |  |  |  |  |  |
| rimegepant | 24 | 42,923 | 517 | 1,395,966 | 8 | 23,952 | 179 | 1,151,519 | 1.54 | 2.28 | -0.391 |  | 0.407 | -0.960 | 0.337 |  | 0.396 |  | 1.63 | 0.006 |
| glycopyrrolate | 15 | 42,932 | 327 | 1,396,156 | 6 | 23,954 | 237 | 1,151,461 | 1.54 | 1.32 | 0.157 |  | 0.475 | 0.330 | 0.741 |  | 0.678 |  | 1.40 | 0.128 |
| erenumab | 7 | 42,940 | 158 | 1,396,325 | 0 | 0 | 0 | 0 | 1.54 | 1.00 | 0.431 |  | 2.853 | 0.151 | 0.880 |  |  |  |  |  |
| fluticasone furoate umeclidinium bromide vilanterol trifenatate | 31 | 42,916 | 667 | 1,395,816 | 16 | 23,944 | 546 | 1,151,152 | 1.53 | 1.45 | 0.056 |  | 0.309 | 0.180 | 0.857 |  | 0.822 |  | 1.47 | 0.009 |
| epoprostenol | 56 | 42,891 | 1,199 | 1,395,284 | 11 | 23,949 | 524 | 1,151,174 | 1.53 | 1.05 | 0.374 |  | 0.328 | 1.142 | 0.254 |  | 0.217 |  | 1.40 | 0.006 |
| tizanidine | 7 | 42,940 | 159 | 1,396,324 | 0 | 0 | 0 | 0 | 1.53 | 1.00 | 0.425 |  | 2.853 | 0.149 | 0.882 |  |  |  |  |  |
| paroxetine | 36 | 42,911 | 779 | 1,395,704 | 20 | 23,940 | 493 | 1,151,205 | 1.52 | 2.00 | -0.271 |  | 0.282 | -0.962 | 0.336 |  | 0.359 |  | 1.64 | <0.001 |
| asfotase alfa | 13 | 42,934 | 289 | 1,396,194 | 0 | 0 | 0 | 0 | 1.52 | 1.00 | 0.416 |  | 2.842 | 0.146 | 0.884 |  |  |  |  |  |
| buprenorphine | 44 | 42,903 | 959 | 1,395,524 | 50 | 23,910 | 873 | 1,150,825 | 1.51 | 2.78 | -0.612 |  | 0.211 | -2.901 | 0.004 |  | 0.003 |  | 1.98 | <0.001 |
| icatibant | 41 | 42,906 | 905 | 1,395,578 | 10 | 23,950 | 344 | 1,151,354 | 1.49 | 1.47 | 0.017 |  | 0.351 | 0.049 | 0.961 |  | 0.882 |  | 1.46 | 0.008 |
| budesonide formoterol | 19 | 42,928 | 425 | 1,396,058 | 7 | 23,953 | 296 | 1,151,402 | 1.49 | 1.22 | 0.204 |  | 0.436 | 0.466 | 0.641 |  | 0.583 |  | 1.35 | 0.130 |
| dimethyl | 121 | 42,826 | 2,694 | 1,393,789 | 30 | 23,930 | 1,325 | 1,150,373 | 1.47 | 1.11 | 0.283 |  | 0.205 | 1.376 | 0.169 |  | 0.153 |  | 1.37 | <0.001 |
| esketamine | 14 | 42,933 | 321 | 1,396,162 | 7 | 23,953 | 238 | 1,151,460 | 1.47 | 1.51 | -0.030 |  | 0.458 | -0.066 | 0.947 |  | 0.995 |  | 1.42 | 0.115 |
| amifampridine | 25 | 42,922 | 568 | 1,395,915 | 17 | 23,943 | 390 | 1,151,308 | 1.46 | 2.15 | -0.390 |  | 0.317 | -1.229 | 0.219 |  | 0.233 |  | 1.64 | 0.001 |
| caplacizumab yhdp | 19 | 42,928 | 435 | 1,396,048 | 0 | 0 | 0 | 0 | 1.46 | 1.00 | 0.376 |  | 2.838 | 0.132 | 0.895 |  |  |  |  |  |
| quetiapine | 128 | 42,819 | 2,886 | 1,393,597 | 93 | 23,867 | 1,974 | 1,149,724 | 1.45 | 2.28 | -0.454 |  | 0.139 | -3.259 | 0.001 |  | 0.001 |  | 1.71 | <0.001 |
| dextroamphetamine | 5 | 42,942 | 123 | 1,396,360 | 0 | 0 | 0 | 0 | 1.45 | 1.00 | 0.370 |  | 2.862 | 0.129 | 0.897 |  |  |  |  |  |
| moxifloxacin | 23 | 42,924 | 529 | 1,395,954 | 20 | 23,940 | 551 | 1,151,147 | 1.44 | 1.79 | -0.214 |  | 0.308 | -0.693 | 0.488 |  | 0.499 |  | 1.55 | 0.004 |
| paliperidone palmitate | 76 | 42,871 | 1,731 | 1,394,752 | 143 | 23,817 | 2,556 | 1,149,142 | 1.44 | 2.71 | -0.633 |  | 0.145 | -4.364 | <0.001 |  | <0.001 |  | 2.07 | <0.001 |
| telotristat ethyl | 8 | 42,939 | 192 | 1,396,291 | 8 | 23,952 | 152 | 1,151,546 | 1.44 | 2.68 | -0.624 |  | 0.497 | -1.255 | 0.209 |  | 0.215 |  | 1.77 | 0.024 |
| fluticasone propionate | 27 | 42,920 | 623 | 1,395,860 | 23 | 23,937 | 509 | 1,151,189 | 1.43 | 2.22 | -0.436 |  | 0.287 | -1.517 | 0.129 |  | 0.133 |  | 1.68 | <0.001 |
| pomalidomide | 127 | 42,820 | 2,901 | 1,393,582 | 145 | 23,815 | 3,140 | 1,148,558 | 1.43 | 2.23 | -0.446 |  | 0.124 | -3.590 | <0.001 |  | <0.001 |  | 1.77 | <0.001 |
| ribociclib | 208 | 42,739 | 4,772 | 1,391,711 | 0 | 0 | 0 | 0 | 1.42 | 1.00 | 0.352 |  | 2.829 | 0.125 | 0.901 |  |  |  |  |  |
| eslicarbazepine | 12 | 42,935 | 287 | 1,396,196 | 10 | 23,950 | 203 | 1,151,495 | 1.41 | 2.48 | -0.562 |  | 0.429 | -1.312 | 0.190 |  | 0.200 |  | 1.69 | 0.015 |
| dexlansoprazole | 11 | 42,936 | 265 | 1,396,218 | 0 | 0 | 0 | 0 | 1.41 | 1.00 | 0.343 |  | 2.844 | 0.120 | 0.904 |  |  |  |  |  |
| anifrolumab fnia | 9 | 42,938 | 219 | 1,396,264 | 0 | 0 | 0 | 0 | 1.41 | 1.00 | 0.342 |  | 2.848 | 0.120 | 0.904 |  |  |  |  |  |
| aclidinium bromide | 11 | 42,936 | 266 | 1,396,217 | 5 | 23,955 | 151 | 1,151,547 | 1.40 | 1.75 | -0.218 |  | 0.528 | -0.413 | 0.680 |  | 0.758 |  | 1.41 | 0.172 |
| tocilizumab | 197 | 42,750 | 4,631 | 1,391,852 | 40 | 23,920 | 2,526 | 1,149,172 | 1.39 | 0.77 | 0.589 |  | 0.174 | 3.378 | <0.001 |  | <0.001 |  | 1.22 | 0.003 |
| prednisone | 49 | 42,898 | 1,161 | 1,395,322 | 28 | 23,932 | 1,186 | 1,150,512 | 1.39 | 1.15 | 0.183 |  | 0.239 | 0.765 | 0.444 |  | 0.429 |  | 1.28 | 0.036 |
| vibegron | 8 | 42,939 | 199 | 1,396,284 | 0 | 0 | 0 | 0 | 1.39 | 1.00 | 0.326 |  | 2.850 | 0.114 | 0.909 |  |  |  |  |  |
| mirtazapine | 46 | 42,901 | 1,109 | 1,395,374 | 30 | 23,930 | 761 | 1,150,937 | 1.36 | 1.93 | -0.346 |  | 0.238 | -1.454 | 0.146 |  | 0.153 |  | 1.52 | <0.001 |
| norethindrone | 5 | 42,942 | 131 | 1,396,352 | 0 | 0 | 0 | 0 | 1.36 | 1.00 | 0.307 |  | 2.862 | 0.107 | 0.914 |  |  |  |  |  |
| ambrisentan | 66 | 42,881 | 1,594 | 1,394,889 | 24 | 23,936 | 1,042 | 1,150,656 | 1.36 | 1.13 | 0.183 |  | 0.240 | 0.763 | 0.445 |  | 0.417 |  | 1.27 | 0.024 |
| raltegravir | 9 | 42,938 | 228 | 1,396,255 | 0 | 0 | 0 | 0 | 1.35 | 1.00 | 0.302 |  | 2.848 | 0.106 | 0.916 |  |  |  |  |  |
| sparsentan | 12 | 42,935 | 301 | 1,396,182 | 18 | 23,942 | 333 | 1,151,365 | 1.35 | 2.67 | -0.682 |  | 0.375 | -1.821 | 0.069 |  | 0.063 |  | 1.86 | <0.001 |
| fentanyl | 49 | 42,898 | 1,202 | 1,395,281 | 39 | 23,921 | 940 | 1,150,758 | 1.34 | 2.02 | -0.411 |  | 0.218 | -1.888 | 0.059 |  | 0.060 |  | 1.56 | <0.001 |
| tamoxifen | 21 | 42,926 | 522 | 1,395,961 | 0 | 0 | 0 | 0 | 1.34 | 1.00 | 0.291 |  | 2.837 | 0.103 | 0.918 |  |  |  |  |  |
| rituximab pvvr | 27 | 42,920 | 670 | 1,395,813 | 8 | 23,952 | 478 | 1,151,220 | 1.33 | 0.85 | 0.446 |  | 0.397 | 1.124 | 0.261 |  | 0.226 |  | 1.14 | 0.431 |
| tramadol | 6 | 42,941 | 158 | 1,396,325 | 0 | 0 | 0 | 0 | 1.33 | 1.00 | 0.288 |  | 2.857 | 0.101 | 0.920 |  |  |  |  |  |
| umeclidinium bromide vilanterol trifenatate | 15 | 42,932 | 378 | 1,396,105 | 0 | 0 | 0 | 0 | 1.33 | 1.00 | 0.286 |  | 2.840 | 0.101 | 0.920 |  |  |  |  |  |
| acetaminophen oxycodone | 20 | 42,927 | 501 | 1,395,982 | 18 | 23,942 | 361 | 1,151,337 | 1.33 | 2.46 | -0.616 |  | 0.328 | -1.877 | 0.061 |  | 0.061 |  | 1.66 | 0.002 |
| etanercept szzs | 30 | 42,917 | 746 | 1,395,737 | 7 | 23,953 | 412 | 1,151,286 | 1.33 | 0.87 | 0.419 |  | 0.412 | 1.016 | 0.309 |  | 0.263 |  | 1.17 | 0.339 |
| hydrochlorothiazide losartan | 10 | 42,937 | 262 | 1,396,221 | 0 | 0 | 0 | 0 | 1.30 | 1.00 | 0.263 |  | 2.846 | 0.092 | 0.926 |  |  |  |  |  |
| triptorelin | 9 | 42,938 | 237 | 1,396,246 | 0 | 0 | 0 | 0 | 1.30 | 1.00 | 0.263 |  | 2.848 | 0.092 | 0.926 |  |  |  |  |  |
| rituximab | 192 | 42,755 | 4,818 | 1,391,665 | 43 | 23,917 | 3,568 | 1,148,130 | 1.30 | 0.59 | 0.798 |  | 0.170 | 4.710 | <0.001 |  | <0.001 |  | 1.06 | 0.417 |
| glecaprevir pibrentasvir | 49 | 42,898 | 1,241 | 1,395,242 | 41 | 23,919 | 1,397 | 1,150,301 | 1.30 | 1.43 | -0.096 |  | 0.214 | -0.450 | 0.653 |  | 0.661 |  | 1.34 | 0.006 |
| levonorgestrel | 19 | 42,928 | 489 | 1,395,994 | 0 | 0 | 0 | 0 | 1.30 | 1.00 | 0.259 |  | 2.838 | 0.091 | 0.927 |  |  |  |  |  |
| carvedilol | 9 | 42,938 | 239 | 1,396,244 | 0 | 0 | 0 | 0 | 1.29 | 1.00 | 0.255 |  | 2.848 | 0.089 | 0.929 |  |  |  |  |  |
| budesonide formoterol glycopyrronium | 16 | 42,931 | 419 | 1,396,064 | 8 | 23,952 | 402 | 1,151,296 | 1.28 | 1.02 | 0.231 |  | 0.428 | 0.540 | 0.589 |  | 0.551 |  | 1.13 | 0.559 |
| albuterol | 38 | 42,909 | 979 | 1,395,504 | 13 | 23,947 | 664 | 1,151,034 | 1.28 | 0.98 | 0.269 |  | 0.320 | 0.841 | 0.401 |  | 0.365 |  | 1.16 | 0.295 |
| methylprednisolone | 101 | 42,846 | 2,599 | 1,393,884 | 47 | 23,913 | 2,213 | 1,149,485 | 1.27 | 1.03 | 0.208 |  | 0.178 | 1.167 | 0.243 |  | 0.232 |  | 1.17 | 0.054 |
| maribavir | 5 | 42,942 | 142 | 1,396,341 | 6 | 23,954 | 154 | 1,151,544 | 1.26 | 2.02 | -0.477 |  | 0.591 | -0.807 | 0.419 |  | 0.421 |  | 1.45 | 0.219 |
| morphine | 30 | 42,917 | 791 | 1,395,692 | 28 | 23,932 | 785 | 1,150,913 | 1.25 | 1.74 | -0.331 |  | 0.265 | -1.247 | 0.212 |  | 0.216 |  | 1.43 | 0.007 |
| alprazolam | 53 | 42,894 | 1,392 | 1,395,091 | 26 | 23,934 | 927 | 1,150,771 | 1.25 | 1.37 | -0.095 |  | 0.241 | -0.392 | 0.695 |  | 0.726 |  | 1.27 | 0.035 |
| adalimumab adaz | 35 | 42,912 | 925 | 1,395,558 | 24 | 23,936 | 680 | 1,151,018 | 1.25 | 1.73 | -0.328 |  | 0.268 | -1.225 | 0.221 |  | 0.232 |  | 1.39 | 0.013 |
| rifaximin | 40 | 42,907 | 1,058 | 1,395,425 | 26 | 23,934 | 927 | 1,150,771 | 1.24 | 1.37 | -0.099 |  | 0.254 | -0.389 | 0.697 |  | 0.718 |  | 1.27 | 0.052 |
| clofazimine | 9 | 42,938 | 248 | 1,396,235 | 8 | 23,952 | 199 | 1,151,499 | 1.24 | 2.05 | -0.499 |  | 0.482 | -1.037 | 0.300 |  | 0.315 |  | 1.45 | 0.133 |
| vandetanib | 7 | 42,940 | 197 | 1,396,286 | 0 | 0 | 0 | 0 | 1.23 | 1.00 | 0.211 |  | 2.853 | 0.074 | 0.941 |  |  |  |  |  |
| eltrombopag olamine | 60 | 42,887 | 1,614 | 1,394,869 | 30 | 23,930 | 1,399 | 1,150,299 | 1.22 | 1.05 | 0.151 |  | 0.225 | 0.672 | 0.502 |  | 0.481 |  | 1.14 | 0.213 |
| migalastat | 6 | 42,941 | 173 | 1,396,310 | 0 | 0 | 0 | 0 | 1.22 | 1.00 | 0.197 |  | 2.857 | 0.069 | 0.945 |  |  |  |  |  |
| alendronic acid | 11 | 42,936 | 308 | 1,396,175 | 0 | 0 | 0 | 0 | 1.21 | 1.00 | 0.192 |  | 2.844 | 0.068 | 0.946 |  |  |  |  |  |
| obeticholic acid | 37 | 42,910 | 1,006 | 1,395,477 | 6 | 23,954 | 216 | 1,151,482 | 1.21 | 1.44 | -0.175 |  | 0.431 | -0.405 | 0.685 |  | 0.805 |  | 1.21 | 0.211 |
| ozanimod | 48 | 42,899 | 1,302 | 1,395,181 | 39 | 23,921 | 910 | 1,150,788 | 1.21 | 2.09 | -0.544 |  | 0.219 | -2.488 | 0.013 |  | 0.013 |  | 1.48 | <0.001 |
| estradiol norethindrone | 21 | 42,926 | 579 | 1,395,904 | 0 | 0 | 0 | 0 | 1.21 | 1.00 | 0.188 |  | 2.837 | 0.066 | 0.947 |  |  |  |  |  |
| lecanemab irmb | 11 | 42,936 | 310 | 1,396,173 | 0 | 0 | 0 | 0 | 1.20 | 1.00 | 0.186 |  | 2.844 | 0.065 | 0.948 |  |  |  |  |  |
| anakinra | 39 | 42,908 | 1,067 | 1,395,416 | 10 | 23,950 | 745 | 1,150,953 | 1.20 | 0.68 | 0.575 |  | 0.351 | 1.641 | 0.101 |  | 0.083 |  | 1.01 | 0.929 |
| rasagiline | 7 | 42,940 | 203 | 1,396,280 | 0 | 0 | 0 | 0 | 1.20 | 1.00 | 0.181 |  | 2.853 | 0.063 | 0.949 |  |  |  |  |  |
| rizatriptan benzoate | 5 | 42,942 | 149 | 1,396,334 | 0 | 0 | 0 | 0 | 1.20 | 1.00 | 0.179 |  | 2.862 | 0.063 | 0.950 |  |  |  |  |  |
| onabotulinumtoxina | 44 | 42,903 | 1,210 | 1,395,273 | 7 | 23,953 | 533 | 1,151,165 | 1.20 | 0.68 | 0.571 |  | 0.398 | 1.433 | 0.152 |  | 0.120 |  | 1.05 | 0.707 |
| tapentadol | 9 | 42,938 | 258 | 1,396,225 | 5 | 23,955 | 198 | 1,151,500 | 1.20 | 1.33 | -0.108 |  | 0.544 | -0.199 | 0.842 |  | 0.905 |  | 1.16 | 0.581 |
| metformin vildagliptin | 12 | 42,935 | 340 | 1,396,143 | 14 | 23,946 | 394 | 1,151,304 | 1.19 | 1.77 | -0.392 |  | 0.393 | -0.998 | 0.318 |  | 0.317 |  | 1.40 | 0.093 |
| bamlanivimab | 12 | 42,935 | 341 | 1,396,142 | 7 | 23,953 | 300 | 1,151,398 | 1.19 | 1.20 | -0.008 |  | 0.469 | -0.017 | 0.987 |  | 0.967 |  | 1.14 | 0.584 |
| fluticasone propionate salmeterol xinafoate | 30 | 42,917 | 836 | 1,395,647 | 7 | 23,953 | 589 | 1,151,109 | 1.19 | 0.61 | 0.662 |  | 0.411 | 1.611 | 0.107 |  | 0.085 |  | 0.97 | 0.871 |
| telmisartan | 8 | 42,939 | 233 | 1,396,250 | 0 | 0 | 0 | 0 | 1.18 | 1.00 | 0.169 |  | 2.850 | 0.059 | 0.953 |  |  |  |  |  |
| methotrexate | 135 | 42,812 | 3,743 | 1,392,740 | 46 | 23,914 | 2,557 | 1,149,141 | 1.18 | 0.87 | 0.298 |  | 0.172 | 1.734 | 0.083 |  | 0.076 |  | 1.07 | 0.339 |
| amikacin | 33 | 42,914 | 930 | 1,395,553 | 15 | 23,945 | 644 | 1,151,054 | 1.17 | 1.16 | 0.013 |  | 0.312 | 0.041 | 0.968 |  | 0.924 |  | 1.14 | 0.362 |
| topiramate | 52 | 42,895 | 1,460 | 1,395,023 | 17 | 23,943 | 557 | 1,151,141 | 1.17 | 1.51 | -0.255 |  | 0.281 | -0.910 | 0.363 |  | 0.404 |  | 1.22 | 0.101 |
| divalproex | 17 | 42,930 | 487 | 1,395,996 | 14 | 23,946 | 484 | 1,151,214 | 1.17 | 1.44 | -0.209 |  | 0.361 | -0.579 | 0.562 |  | 0.579 |  | 1.24 | 0.240 |
| siponimod | 56 | 42,891 | 1,574 | 1,394,909 | 23 | 23,937 | 840 | 1,150,858 | 1.17 | 1.34 | -0.141 |  | 0.249 | -0.567 | 0.571 |  | 0.608 |  | 1.20 | 0.111 |
| fluticasone furoate vilanterol trifenatate | 23 | 42,924 | 657 | 1,395,826 | 14 | 23,946 | 441 | 1,151,257 | 1.16 | 1.58 | -0.306 |  | 0.340 | -0.902 | 0.367 |  | 0.393 |  | 1.26 | 0.165 |
| abatacept | 233 | 42,714 | 6,537 | 1,389,946 | 25 | 23,935 | 2,740 | 1,148,958 | 1.16 | 0.45 | 0.956 |  | 0.210 | 4.555 | <0.001 |  | <0.001 |  | 1.00 | 0.983 |
| voriconazole | 23 | 42,924 | 659 | 1,395,824 | 32 | 23,928 | 1,038 | 1,150,660 | 1.16 | 1.50 | -0.261 |  | 0.275 | -0.949 | 0.343 |  | 0.335 |  | 1.32 | 0.045 |
| cedazuridine decitabine | 6 | 42,941 | 182 | 1,396,301 | 0 | 0 | 0 | 0 | 1.16 | 1.00 | 0.147 |  | 2.856 | 0.051 | 0.959 |  |  |  |  |  |
| teprotumumab trbw | 14 | 42,933 | 408 | 1,396,075 | 12 | 23,948 | 197 | 1,151,501 | 1.15 | 3.04 | -0.969 |  | 0.396 | -2.450 | 0.014 |  | 0.013 |  | 1.57 | 0.024 |
| lanreotide | 45 | 42,902 | 1,283 | 1,395,200 | 23 | 23,937 | 1,102 | 1,150,596 | 1.15 | 1.02 | 0.118 |  | 0.257 | 0.458 | 0.647 |  | 0.621 |  | 1.09 | 0.485 |
| epirubicin | 30 | 42,917 | 866 | 1,395,617 | 0 | 0 | 0 | 0 | 1.14 | 1.00 | 0.135 |  | 2.834 | 0.048 | 0.962 |  |  |  |  |  |
| lorazepam | 32 | 42,915 | 925 | 1,395,558 | 18 | 23,942 | 587 | 1,151,111 | 1.14 | 1.51 | -0.282 |  | 0.296 | -0.952 | 0.341 |  | 0.365 |  | 1.23 | 0.148 |
| trazodone | 7 | 42,940 | 214 | 1,396,269 | 0 | 0 | 0 | 0 | 1.14 | 1.00 | 0.128 |  | 2.853 | 0.045 | 0.964 |  |  |  |  |  |
| ponatinib | 31 | 42,916 | 904 | 1,395,579 | 33 | 23,927 | 1,077 | 1,150,621 | 1.13 | 1.50 | -0.278 |  | 0.252 | -1.101 | 0.271 |  | 0.272 |  | 1.28 | 0.055 |
| nilotinib | 56 | 42,891 | 1,622 | 1,394,861 | 34 | 23,926 | 1,852 | 1,149,846 | 1.13 | 0.89 | 0.235 |  | 0.219 | 1.075 | 0.282 |  | 0.273 |  | 1.02 | 0.872 |
| leuprolide | 40 | 42,907 | 1,163 | 1,395,320 | 230 | 23,730 | 5,310 | 1,146,388 | 1.13 | 2.10 | -0.616 |  | 0.174 | -3.551 | <0.001 |  | <0.001 |  | 1.85 | <0.001 |
| fluoxetine | 38 | 42,909 | 1,111 | 1,395,372 | 21 | 23,939 | 492 | 1,151,206 | 1.13 | 2.10 | -0.623 |  | 0.275 | -2.266 | 0.023 |  | 0.025 |  | 1.33 | 0.031 |
| eptinezumab jjmr | 38 | 42,909 | 1,113 | 1,395,370 | 11 | 23,949 | 407 | 1,151,291 | 1.12 | 1.36 | -0.188 |  | 0.341 | -0.551 | 0.582 |  | 0.651 |  | 1.15 | 0.341 |
| evolocumab | 48 | 42,899 | 1,405 | 1,395,078 | 28 | 23,932 | 1,509 | 1,150,189 | 1.12 | 0.91 | 0.212 |  | 0.239 | 0.889 | 0.374 |  | 0.360 |  | 1.02 | 0.877 |
| candesartan cilexetil | 16 | 42,931 | 480 | 1,396,003 | 0 | 0 | 0 | 0 | 1.12 | 1.00 | 0.110 |  | 2.839 | 0.039 | 0.969 |  |  |  |  |  |
| levocetirizine dihydrochloride | 6 | 42,941 | 189 | 1,396,294 | 0 | 0 | 0 | 0 | 1.12 | 1.00 | 0.109 |  | 2.856 | 0.038 | 0.970 |  |  |  |  |  |
| icosapent ethyl | 11 | 42,936 | 335 | 1,396,148 | 5 | 23,955 | 435 | 1,151,263 | 1.11 | 0.61 | 0.608 |  | 0.524 | 1.161 | 0.246 |  | 0.218 |  | 0.83 | 0.448 |
| abemaciclib | 90 | 42,857 | 2,644 | 1,393,839 | 0 | 0 | 0 | 0 | 1.11 | 1.00 | 0.107 |  | 2.830 | 0.038 | 0.970 |  |  |  |  |  |
| infliximab | 230 | 42,717 | 6,750 | 1,389,733 | 178 | 23,782 | 6,085 | 1,145,613 | 1.11 | 1.41 | -0.241 |  | 0.102 | -2.368 | 0.018 |  | 0.018 |  | 1.22 | <0.001 |
| ibrutinib | 126 | 42,821 | 3,725 | 1,392,758 | 161 | 23,799 | 5,698 | 1,146,000 | 1.10 | 1.36 | -0.212 |  | 0.121 | -1.751 | 0.080 |  | 0.079 |  | 1.23 | <0.001 |
| ixazomib | 70 | 42,877 | 2,079 | 1,394,404 | 64 | 23,896 | 2,265 | 1,149,433 | 1.10 | 1.37 | -0.217 |  | 0.175 | -1.238 | 0.216 |  | 0.218 |  | 1.21 | 0.031 |
| exemestane | 25 | 42,922 | 753 | 1,395,730 | 0 | 0 | 0 | 0 | 1.10 | 1.00 | 0.096 |  | 2.836 | 0.034 | 0.973 |  |  |  |  |  |
| risedronate | 11 | 42,936 | 340 | 1,396,143 | 0 | 0 | 0 | 0 | 1.10 | 1.00 | 0.094 |  | 2.844 | 0.033 | 0.974 |  |  |  |  |  |
| simvastatin | 22 | 42,925 | 667 | 1,395,816 | 21 | 23,939 | 716 | 1,150,982 | 1.10 | 1.44 | -0.275 |  | 0.306 | -0.897 | 0.370 |  | 0.376 |  | 1.22 | 0.207 |
| umeclidinium bromide | 7 | 42,940 | 223 | 1,396,260 | 0 | 0 | 0 | 0 | 1.09 | 1.00 | 0.087 |  | 2.853 | 0.031 | 0.976 |  |  |  |  |  |
| pacritinib | 6 | 42,941 | 194 | 1,396,289 | 11 | 23,949 | 204 | 1,151,494 | 1.09 | 2.70 | -0.912 |  | 0.501 | -1.820 | 0.069 |  | 0.058 |  | 1.67 | 0.036 |
| sarilumab | 62 | 42,885 | 1,899 | 1,394,584 | 17 | 23,943 | 831 | 1,150,867 | 1.07 | 1.01 | 0.056 |  | 0.274 | 0.205 | 0.838 |  | 0.782 |  | 1.04 | 0.708 |
| mepolizumab | 47 | 42,900 | 1,449 | 1,395,034 | 24 | 23,936 | 848 | 1,150,850 | 1.07 | 1.39 | -0.265 |  | 0.253 | -1.047 | 0.295 |  | 0.316 |  | 1.14 | 0.270 |
| metoclopramide | 6 | 42,941 | 199 | 1,396,284 | 0 | 0 | 0 | 0 | 1.06 | 1.00 | 0.058 |  | 2.856 | 0.020 | 0.984 |  |  |  |  |  |
| human immunoglobulin g | 181 | 42,766 | 5,595 | 1,390,888 | 78 | 23,882 | 4,554 | 1,147,144 | 1.05 | 0.83 | 0.242 |  | 0.137 | 1.772 | 0.076 |  | 0.072 |  | 0.97 | 0.631 |
| aripiprazole lauroxil | 16 | 42,931 | 509 | 1,395,974 | 22 | 23,938 | 464 | 1,151,234 | 1.05 | 2.33 | -0.794 |  | 0.331 | -2.402 | 0.016 |  | 0.014 |  | 1.50 | 0.013 |
| fruquintinib | 7 | 42,940 | 232 | 1,396,251 | 8 | 23,952 | 254 | 1,151,444 | 1.05 | 1.61 | -0.426 |  | 0.509 | -0.836 | 0.403 |  | 0.406 |  | 1.21 | 0.468 |
| formoterol glycopyrrolate | 7 | 42,940 | 237 | 1,396,246 | 0 | 0 | 0 | 0 | 1.03 | 1.00 | 0.026 |  | 2.853 | 0.009 | 0.993 |  |  |  |  |  |
| adalimumab isopropyl alcohol | 12 | 42,935 | 396 | 1,396,087 | 0 | 0 | 0 | 0 | 1.03 | 1.00 | 0.025 |  | 2.843 | 0.009 | 0.993 |  |  |  |  |  |
| bempedoic acid | 5 | 42,942 | 174 | 1,396,309 | 0 | 0 | 0 | 0 | 1.02 | 1.00 | 0.025 |  | 2.861 | 0.009 | 0.993 |  |  |  |  |  |
| alirocumab | 31 | 42,916 | 1,003 | 1,395,480 | 27 | 23,933 | 905 | 1,150,793 | 1.02 | 1.46 | -0.358 |  | 0.265 | -1.351 | 0.177 |  | 0.181 |  | 1.17 | 0.242 |
| bosutinib | 23 | 42,924 | 752 | 1,395,731 | 15 | 23,945 | 780 | 1,150,918 | 1.02 | 0.95 | 0.062 |  | 0.331 | 0.187 | 0.852 |  | 0.827 |  | 0.97 | 0.830 |
| drospirenone | 7 | 42,940 | 240 | 1,396,243 | 0 | 0 | 0 | 0 | 1.01 | 1.00 | 0.014 |  | 2.853 | 0.005 | 0.996 |  |  |  |  |  |
| sulfamethoxazole trimethoprim | 16 | 42,931 | 529 | 1,395,954 | 6 | 23,954 | 634 | 1,151,064 | 1.01 | 0.49 | 0.722 |  | 0.467 | 1.546 | 0.122 |  | 0.102 |  | 0.75 | 0.172 |
| mirabegron | 21 | 42,926 | 690 | 1,395,793 | 17 | 23,943 | 493 | 1,151,205 | 1.01 | 1.70 | -0.521 |  | 0.327 | -1.592 | 0.111 |  | 0.116 |  | 1.21 | 0.249 |
| interferon beta 1a | 110 | 42,837 | 3,550 | 1,392,933 | 41 | 23,919 | 1,390 | 1,150,308 | 1.01 | 1.44 | -0.349 |  | 0.185 | -1.889 | 0.059 |  | 0.064 |  | 1.09 | 0.275 |
| amlodipine besylate hydrochlorothiazide valsartan | 9 | 42,938 | 305 | 1,396,178 | 0 | 0 | 0 | 0 | 1.01 | 1.00 | 0.011 |  | 2.848 | 0.004 | 0.997 |  |  |  |  |  |
| losartan | 33 | 42,914 | 1,078 | 1,395,405 | 21 | 23,939 | 817 | 1,150,881 | 1.01 | 1.26 | -0.225 |  | 0.280 | -0.801 | 0.423 |  | 0.444 |  | 1.08 | 0.589 |
| vedolizumab | 207 | 42,740 | 6,681 | 1,389,802 | 127 | 23,833 | 5,988 | 1,145,710 | 1.01 | 1.02 | -0.013 |  | 0.114 | -0.117 | 0.907 |  | 0.917 |  | 1.01 | 0.829 |
| beclomethasone dipropionate | 5 | 42,942 | 179 | 1,396,304 | 0 | 0 | 0 | 0 | 1.00 | 1.00 | -0.004 |  | 2.861 | -0.001 | 0.999 |  |  |  |  |  |
| pravastatin | 6 | 42,941 | 213 | 1,396,270 | 5 | 23,955 | 212 | 1,151,486 | 0.99 | 1.24 | -0.229 |  | 0.587 | -0.389 | 0.697 |  | 0.728 |  | 1.00 | 0.989 |
| avacopan | 15 | 42,932 | 509 | 1,395,974 | 12 | 23,948 | 431 | 1,151,267 | 0.99 | 1.39 | -0.342 |  | 0.386 | -0.887 | 0.375 |  | 0.393 |  | 1.10 | 0.633 |
| cholestyramine | 6 | 42,941 | 214 | 1,396,269 | 0 | 0 | 0 | 0 | 0.99 | 1.00 | -0.015 |  | 2.856 | -0.005 | 0.996 |  |  |  |  |  |
| risankizumab rzaa | 115 | 42,832 | 3,815 | 1,392,668 | 95 | 23,865 | 3,869 | 1,147,829 | 0.98 | 1.19 | -0.187 |  | 0.140 | -1.334 | 0.182 |  | 0.185 |  | 1.06 | 0.389 |
| fesoterodine | 8 | 42,939 | 283 | 1,396,200 | 0 | 0 | 0 | 0 | 0.97 | 1.00 | -0.025 |  | 2.850 | -0.009 | 0.993 |  |  |  |  |  |
| hydrochlorothiazide valsartan | 13 | 42,934 | 451 | 1,396,032 | 0 | 0 | 0 | 0 | 0.97 | 1.00 | -0.028 |  | 2.842 | -0.010 | 0.992 |  |  |  |  |  |
| bosentan | 50 | 42,897 | 1,689 | 1,394,794 | 12 | 23,948 | 733 | 1,150,965 | 0.97 | 0.82 | 0.171 |  | 0.319 | 0.536 | 0.592 |  | 0.534 |  | 0.92 | 0.530 |
| ipratropium bromide | 7 | 42,940 | 253 | 1,396,230 | 0 | 0 | 0 | 0 | 0.96 | 1.00 | -0.039 |  | 2.853 | -0.014 | 0.989 |  |  |  |  |  |
| elagolix estradiol norethindrone | 6 | 42,941 | 220 | 1,396,263 | 0 | 0 | 0 | 0 | 0.96 | 1.00 | -0.042 |  | 2.856 | -0.015 | 0.988 |  |  |  |  |  |
| mavacamten | 14 | 42,933 | 493 | 1,395,990 | 9 | 23,951 | 337 | 1,151,361 | 0.96 | 1.35 | -0.348 |  | 0.423 | -0.822 | 0.411 |  | 0.445 |  | 1.04 | 0.860 |
| upadacitinib | 177 | 42,770 | 6,049 | 1,390,434 | 75 | 23,885 | 3,835 | 1,147,863 | 0.95 | 0.95 | 0.008 |  | 0.139 | 0.060 | 0.952 |  | 0.931 |  | 0.95 | 0.402 |
| alpha 1 proteinase inhibitor human | 25 | 42,922 | 875 | 1,395,608 | 21 | 23,939 | 677 | 1,151,021 | 0.95 | 1.53 | -0.477 |  | 0.297 | -1.604 | 0.109 |  | 0.112 |  | 1.12 | 0.436 |
| baclofen | 8 | 42,939 | 292 | 1,396,191 | 0 | 0 | 0 | 0 | 0.94 | 1.00 | -0.057 |  | 2.850 | -0.020 | 0.984 |  |  |  |  |  |
| teduglutide water | 5 | 42,942 | 189 | 1,396,294 | 0 | 0 | 0 | 0 | 0.94 | 1.00 | -0.058 |  | 2.861 | -0.020 | 0.984 |  |  |  |  |  |
| vigabatrin | 18 | 42,929 | 642 | 1,395,841 | 24 | 23,936 | 630 | 1,151,068 | 0.94 | 1.87 | -0.691 |  | 0.313 | -2.207 | 0.027 |  | 0.025 |  | 1.28 | 0.113 |
| sildenafil | 39 | 42,908 | 1,375 | 1,395,108 | 24 | 23,936 | 1,753 | 1,149,945 | 0.93 | 0.67 | 0.330 |  | 0.260 | 1.270 | 0.204 |  | 0.195 |  | 0.80 | 0.078 |
| idelalisib | 7 | 42,940 | 261 | 1,396,222 | 0 | 0 | 0 | 0 | 0.93 | 1.00 | -0.070 |  | 2.853 | -0.024 | 0.980 |  |  |  |  |  |
| letrozole | 54 | 42,893 | 1,915 | 1,394,568 | 0 | 0 | 0 | 0 | 0.93 | 1.00 | -0.078 |  | 2.832 | -0.028 | 0.978 |  |  |  |  |  |
| fluticasone furoate | 6 | 42,941 | 228 | 1,396,255 | 6 | 23,954 | 146 | 1,151,552 | 0.92 | 2.13 | -0.836 |  | 0.565 | -1.479 | 0.139 |  | 0.143 |  | 1.20 | 0.540 |
| velaglucerase alfa | 10 | 42,937 | 372 | 1,396,111 | 0 | 0 | 0 | 0 | 0.92 | 1.00 | -0.087 |  | 2.846 | -0.031 | 0.976 |  |  |  |  |  |
| lisinopril | 28 | 42,919 | 1,018 | 1,395,465 | 20 | 23,940 | 1,199 | 1,150,499 | 0.91 | 0.82 | 0.102 |  | 0.293 | 0.349 | 0.727 |  | 0.712 |  | 0.85 | 0.276 |
| pancrelipase amylase pancrelipase lipase pancrelipase protease | 39 | 42,908 | 1,412 | 1,395,071 | 15 | 23,945 | 1,178 | 1,150,520 | 0.91 | 0.63 | 0.364 |  | 0.302 | 1.203 | 0.229 |  | 0.208 |  | 0.79 | 0.093 |
| mometasone furoate | 8 | 42,939 | 304 | 1,396,179 | 0 | 0 | 0 | 0 | 0.91 | 1.00 | -0.097 |  | 2.850 | -0.034 | 0.973 |  |  |  |  |  |
| dulaglutide | 41 | 42,906 | 1,491 | 1,394,992 | 29 | 23,931 | 1,397 | 1,150,301 | 0.90 | 1.01 | -0.115 |  | 0.244 | -0.471 | 0.638 |  | 0.655 |  | 0.93 | 0.576 |
| polyethylene glycol 3350 | 31 | 42,916 | 1,132 | 1,395,351 | 30 | 23,930 | 917 | 1,150,781 | 0.90 | 1.60 | -0.570 |  | 0.258 | -2.208 | 0.027 |  | 0.027 |  | 1.13 | 0.334 |
| acalabrutinib | 17 | 42,930 | 631 | 1,395,852 | 26 | 23,934 | 856 | 1,150,842 | 0.90 | 1.49 | -0.501 |  | 0.313 | -1.604 | 0.109 |  | 0.103 |  | 1.16 | 0.345 |
| apixaban | 110 | 42,837 | 4,003 | 1,392,480 | 82 | 23,878 | 4,145 | 1,147,553 | 0.90 | 0.96 | -0.064 |  | 0.147 | -0.434 | 0.665 |  | 0.673 |  | 0.92 | 0.236 |
| terbinafine | 8 | 42,939 | 311 | 1,396,172 | 0 | 0 | 0 | 0 | 0.89 | 1.00 | -0.120 |  | 2.850 | -0.042 | 0.967 |  |  |  |  |  |
| metoprolol | 28 | 42,919 | 1,046 | 1,395,437 | 13 | 23,947 | 870 | 1,150,828 | 0.89 | 0.75 | 0.172 |  | 0.334 | 0.517 | 0.606 |  | 0.570 |  | 0.82 | 0.195 |
| azelastine | 6 | 42,941 | 239 | 1,396,244 | 5 | 23,955 | 170 | 1,151,528 | 0.88 | 1.55 | -0.564 |  | 0.588 | -0.959 | 0.338 |  | 0.365 |  | 1.01 | 0.970 |
| satralizumab mwge | 9 | 42,938 | 350 | 1,396,133 | 0 | 0 | 0 | 0 | 0.88 | 1.00 | -0.126 |  | 2.847 | -0.044 | 0.965 |  |  |  |  |  |
| ibandronate | 15 | 42,932 | 572 | 1,395,911 | 0 | 0 | 0 | 0 | 0.88 | 1.00 | -0.127 |  | 2.840 | -0.045 | 0.964 |  |  |  |  |  |
| voxelotor | 38 | 42,909 | 1,428 | 1,395,055 | 25 | 23,935 | 1,221 | 1,150,477 | 0.88 | 1.00 | -0.136 |  | 0.258 | -0.525 | 0.600 |  | 0.621 |  | 0.91 | 0.454 |
| prednisolone | 31 | 42,916 | 1,173 | 1,395,310 | 27 | 23,933 | 1,013 | 1,150,685 | 0.87 | 1.30 | -0.402 |  | 0.265 | -1.519 | 0.129 |  | 0.132 |  | 1.02 | 0.906 |
| cladribine | 37 | 42,910 | 1,407 | 1,395,076 | 12 | 23,948 | 540 | 1,151,158 | 0.87 | 1.11 | -0.249 |  | 0.331 | -0.755 | 0.450 |  | 0.507 |  | 0.90 | 0.463 |
| atorvastatin | 67 | 42,880 | 2,540 | 1,393,943 | 31 | 23,929 | 2,243 | 1,149,455 | 0.86 | 0.67 | 0.247 |  | 0.218 | 1.135 | 0.256 |  | 0.242 |  | 0.78 | 0.017 |
| brigatinib | 16 | 42,931 | 623 | 1,395,860 | 6 | 23,954 | 544 | 1,151,154 | 0.86 | 0.57 | 0.405 |  | 0.467 | 0.868 | 0.385 |  | 0.342 |  | 0.72 | 0.127 |
| estradiol levonorgestrel | 7 | 42,940 | 285 | 1,396,198 | 0 | 0 | 0 | 0 | 0.85 | 1.00 | -0.158 |  | 2.853 | -0.055 | 0.956 |  |  |  |  |  |
| omadacycline | 6 | 42,941 | 247 | 1,396,236 | 0 | 0 | 0 | 0 | 0.85 | 1.00 | -0.158 |  | 2.856 | -0.055 | 0.956 |  |  |  |  |  |
| certolizumab pegol | 163 | 42,784 | 6,254 | 1,390,229 | 21 | 23,939 | 2,761 | 1,148,937 | 0.85 | 0.37 | 0.821 |  | 0.231 | 3.560 | <0.001 |  | <0.001 |  | 0.74 | <0.001 |
| bictegravir emtricitabine tenofovir alafenamide | 13 | 42,934 | 518 | 1,395,965 | 42 | 23,918 | 1,201 | 1,150,497 | 0.85 | 1.70 | -0.698 |  | 0.317 | -2.203 | 0.028 |  | 0.022 |  | 1.35 | 0.029 |
| vismodegib | 15 | 42,932 | 599 | 1,395,884 | 21 | 23,939 | 800 | 1,150,898 | 0.84 | 1.29 | -0.429 |  | 0.338 | -1.271 | 0.204 |  | 0.197 |  | 1.03 | 0.871 |
| etanercept | 128 | 42,819 | 5,008 | 1,391,475 | 47 | 23,913 | 2,832 | 1,148,866 | 0.83 | 0.81 | 0.034 |  | 0.172 | 0.200 | 0.842 |  | 0.813 |  | 0.82 | 0.010 |
| tramadol | 29 | 42,918 | 1,153 | 1,395,330 | 27 | 23,933 | 930 | 1,150,768 | 0.83 | 1.42 | -0.536 |  | 0.269 | -1.994 | 0.046 |  | 0.046 |  | 1.02 | 0.866 |
| bimekizumab | 6 | 42,941 | 254 | 1,396,229 | 0 | 0 | 0 | 0 | 0.83 | 1.00 | -0.186 |  | 2.856 | -0.065 | 0.948 |  |  |  |  |  |
| somatropin | 70 | 42,877 | 2,775 | 1,393,708 | 51 | 23,909 | 3,225 | 1,148,473 | 0.83 | 0.77 | 0.074 |  | 0.185 | 0.398 | 0.691 |  | 0.681 |  | 0.79 | 0.012 |
| natalizumab | 47 | 42,900 | 1,870 | 1,394,613 | 10 | 23,950 | 708 | 1,150,990 | 0.83 | 0.71 | 0.148 |  | 0.344 | 0.429 | 0.668 |  | 0.597 |  | 0.79 | 0.076 |
| alpelisib | 40 | 42,907 | 1,600 | 1,394,883 | 0 | 0 | 0 | 0 | 0.82 | 1.00 | -0.195 |  | 2.833 | -0.069 | 0.945 |  |  |  |  |  |
| etonogestrel | 8 | 42,939 | 336 | 1,396,147 | 0 | 0 | 0 | 0 | 0.82 | 1.00 | -0.197 |  | 2.850 | -0.069 | 0.945 |  |  |  |  |  |
| sulfasalazine | 11 | 42,936 | 455 | 1,396,028 | 0 | 0 | 0 | 0 | 0.82 | 1.00 | -0.197 |  | 2.844 | -0.069 | 0.945 |  |  |  |  |  |
| peginterferon alfa 2a | 5 | 42,942 | 218 | 1,396,265 | 5 | 23,955 | 229 | 1,151,469 | 0.82 | 1.15 | -0.342 |  | 0.610 | -0.560 | 0.576 |  | 0.591 |  | 0.87 | 0.670 |
| diazepam | 17 | 42,930 | 702 | 1,395,781 | 7 | 23,953 | 567 | 1,151,131 | 0.81 | 0.64 | 0.243 |  | 0.440 | 0.552 | 0.581 |  | 0.531 |  | 0.72 | 0.107 |
| tiotropium bromide | 15 | 42,932 | 623 | 1,395,860 | 5 | 23,955 | 510 | 1,151,188 | 0.81 | 0.52 | 0.445 |  | 0.500 | 0.891 | 0.373 |  | 0.324 |  | 0.67 | 0.075 |
| abrocitinib | 10 | 42,937 | 423 | 1,396,060 | 5 | 23,955 | 409 | 1,151,289 | 0.81 | 0.65 | 0.222 |  | 0.531 | 0.419 | 0.676 |  | 0.625 |  | 0.70 | 0.163 |
| rotigotine | 17 | 42,930 | 708 | 1,395,775 | 26 | 23,934 | 777 | 1,150,921 | 0.80 | 1.64 | -0.713 |  | 0.312 | -2.283 | 0.022 |  | 0.020 |  | 1.14 | 0.411 |
| teriparatide | 49 | 42,898 | 2,012 | 1,394,471 | 5 | 23,955 | 660 | 1,151,038 | 0.80 | 0.40 | 0.692 |  | 0.452 | 1.532 | 0.125 |  | 0.091 |  | 0.71 | 0.014 |
| olodaterol tiotropium bromide | 5 | 42,942 | 224 | 1,396,259 | 0 | 0 | 0 | 0 | 0.80 | 1.00 | -0.227 |  | 2.861 | -0.079 | 0.937 |  |  |  |  |  |
| isotretinoin | 14 | 42,933 | 592 | 1,395,891 | 18 | 23,942 | 504 | 1,151,194 | 0.80 | 1.76 | -0.796 |  | 0.356 | -2.235 | 0.025 |  | 0.023 |  | 1.12 | 0.535 |
| mitotane | 5 | 42,942 | 226 | 1,396,257 | 0 | 0 | 0 | 0 | 0.79 | 1.00 | -0.236 |  | 2.861 | -0.083 | 0.934 |  |  |  |  |  |
| benralizumab | 51 | 42,896 | 2,123 | 1,394,360 | 20 | 23,940 | 1,217 | 1,150,481 | 0.79 | 0.81 | -0.026 |  | 0.264 | -0.099 | 0.921 |  | 0.966 |  | 0.78 | 0.041 |
| human immunoglobulin g hyaluronidase human recombinant | 17 | 42,930 | 726 | 1,395,757 | 5 | 23,955 | 452 | 1,151,246 | 0.78 | 0.58 | 0.293 |  | 0.493 | 0.595 | 0.552 |  | 0.481 |  | 0.69 | 0.087 |
| lacosamide | 24 | 42,923 | 1,018 | 1,395,465 | 19 | 23,941 | 951 | 1,150,747 | 0.78 | 0.99 | -0.231 |  | 0.307 | -0.752 | 0.452 |  | 0.467 |  | 0.84 | 0.264 |
| tucatinib | 8 | 42,939 | 353 | 1,396,130 | 0 | 0 | 0 | 0 | 0.78 | 1.00 | -0.246 |  | 2.850 | -0.086 | 0.931 |  |  |  |  |  |
| estradiol norethindrone relugolix | 6 | 42,941 | 271 | 1,396,212 | 0 | 0 | 0 | 0 | 0.78 | 1.00 | -0.250 |  | 2.856 | -0.088 | 0.930 |  |  |  |  |  |
| fedratinib | 8 | 42,939 | 355 | 1,396,128 | 0 | 0 | 0 | 0 | 0.78 | 1.00 | -0.252 |  | 2.850 | -0.088 | 0.930 |  |  |  |  |  |
| sunitinib | 23 | 42,924 | 986 | 1,395,497 | 26 | 23,934 | 1,503 | 1,150,195 | 0.77 | 0.85 | -0.090 |  | 0.286 | -0.313 | 0.755 |  | 0.751 |  | 0.80 | 0.112 |
| ivacaftor tezacaftor | 15 | 42,932 | 651 | 1,395,832 | 5 | 23,955 | 533 | 1,151,165 | 0.77 | 0.50 | 0.446 |  | 0.500 | 0.891 | 0.373 |  | 0.323 |  | 0.64 | 0.048 |
| levetiracetam | 46 | 42,901 | 1,961 | 1,394,522 | 44 | 23,916 | 1,819 | 1,149,879 | 0.77 | 1.18 | -0.423 |  | 0.212 | -1.990 | 0.047 |  | 0.047 |  | 0.92 | 0.421 |
| teduglutide | 45 | 42,902 | 1,926 | 1,394,557 | 22 | 23,938 | 1,613 | 1,150,085 | 0.77 | 0.67 | 0.136 |  | 0.260 | 0.524 | 0.600 |  | 0.574 |  | 0.72 | 0.008 |
| ticagrelor | 26 | 42,921 | 1,122 | 1,395,361 | 58 | 23,902 | 1,846 | 1,149,852 | 0.77 | 1.52 | -0.686 |  | 0.237 | -2.890 | 0.004 |  | 0.003 |  | 1.15 | 0.194 |
| regorafenib | 17 | 42,930 | 741 | 1,395,742 | 30 | 23,930 | 1,048 | 1,150,650 | 0.77 | 1.40 | -0.600 |  | 0.304 | -1.976 | 0.048 |  | 0.043 |  | 1.06 | 0.714 |
| valsartan | 19 | 42,928 | 832 | 1,395,651 | 16 | 23,944 | 827 | 1,150,871 | 0.76 | 0.96 | -0.230 |  | 0.338 | -0.680 | 0.497 |  | 0.511 |  | 0.82 | 0.240 |
| atenolol | 10 | 42,937 | 448 | 1,396,035 | 5 | 23,955 | 316 | 1,151,382 | 0.76 | 0.84 | -0.093 |  | 0.532 | -0.175 | 0.861 |  | 0.933 |  | 0.74 | 0.240 |
| omeprazole | 60 | 42,887 | 2,621 | 1,393,862 | 32 | 23,928 | 2,014 | 1,149,684 | 0.75 | 0.78 | -0.033 |  | 0.220 | -0.150 | 0.881 |  | 0.907 |  | 0.75 | 0.006 |
| diclofenac | 26 | 42,921 | 1,150 | 1,395,333 | 7 | 23,953 | 1,023 | 1,150,675 | 0.75 | 0.35 | 0.755 |  | 0.416 | 1.815 | 0.070 |  | 0.054 |  | 0.58 | 0.002 |
| bimekizumab bkzx | 6 | 42,941 | 282 | 1,396,201 | 0 | 0 | 0 | 0 | 0.75 | 1.00 | -0.290 |  | 2.856 | -0.102 | 0.919 |  |  |  |  |  |
| pegvaliase pqpz | 10 | 42,937 | 456 | 1,396,027 | 6 | 23,954 | 372 | 1,151,326 | 0.75 | 0.84 | -0.115 |  | 0.504 | -0.227 | 0.820 |  | 0.872 |  | 0.74 | 0.221 |
| doxycycline | 16 | 42,931 | 720 | 1,395,763 | 0 | 0 | 0 | 0 | 0.74 | 1.00 | -0.295 |  | 2.839 | -0.104 | 0.917 |  |  |  |  |  |
| eltrombopag | 11 | 42,936 | 502 | 1,395,981 | 0 | 0 | 0 | 0 | 0.74 | 1.00 | -0.296 |  | 2.844 | -0.104 | 0.917 |  |  |  |  |  |
| ruxolitinib | 9 | 42,938 | 417 | 1,396,066 | 0 | 0 | 0 | 0 | 0.74 | 1.00 | -0.301 |  | 2.847 | -0.106 | 0.916 |  |  |  |  |  |
| capecitabine | 65 | 42,882 | 2,921 | 1,393,562 | 31 | 23,929 | 2,184 | 1,149,514 | 0.73 | 0.69 | 0.051 |  | 0.219 | 0.231 | 0.817 |  | 0.789 |  | 0.71 | <0.001 |
| ravulizumab cwvz | 8 | 42,939 | 381 | 1,396,102 | 13 | 23,947 | 354 | 1,151,344 | 0.72 | 1.83 | -0.927 |  | 0.444 | -2.088 | 0.037 |  | 0.031 |  | 1.10 | 0.657 |
| linaclotide | 14 | 42,933 | 653 | 1,395,830 | 7 | 23,953 | 383 | 1,151,315 | 0.72 | 0.94 | -0.265 |  | 0.454 | -0.583 | 0.560 |  | 0.620 |  | 0.75 | 0.188 |
| haloperidol | 8 | 42,939 | 384 | 1,396,099 | 9 | 23,951 | 528 | 1,151,170 | 0.72 | 0.86 | -0.184 |  | 0.477 | -0.386 | 0.700 |  | 0.698 |  | 0.75 | 0.230 |
| laronidase | 7 | 42,940 | 339 | 1,396,144 | 5 | 23,955 | 323 | 1,151,375 | 0.72 | 0.82 | -0.129 |  | 0.567 | -0.228 | 0.820 |  | 0.862 |  | 0.70 | 0.218 |
| metronidazole | 15 | 42,932 | 703 | 1,395,780 | 6 | 23,954 | 426 | 1,151,272 | 0.72 | 0.73 | -0.022 |  | 0.471 | -0.047 | 0.962 |  | 0.960 |  | 0.69 | 0.089 |
| hydrocortisone | 9 | 42,938 | 432 | 1,396,051 | 0 | 0 | 0 | 0 | 0.71 | 1.00 | -0.337 |  | 2.847 | -0.118 | 0.906 |  |  |  |  |  |
| casirivimab imdevimab | 7 | 42,940 | 343 | 1,396,140 | 0 | 0 | 0 | 0 | 0.71 | 1.00 | -0.343 |  | 2.852 | -0.120 | 0.904 |  |  |  |  |  |
| amlodipine besylate | 42 | 42,905 | 1,947 | 1,394,536 | 38 | 23,922 | 1,597 | 1,150,101 | 0.71 | 1.16 | -0.491 |  | 0.225 | -2.179 | 0.029 |  | 0.029 |  | 0.86 | 0.182 |
| lorlatinib | 17 | 42,930 | 804 | 1,395,679 | 16 | 23,944 | 737 | 1,150,961 | 0.71 | 1.08 | -0.419 |  | 0.347 | -1.208 | 0.227 |  | 0.233 |  | 0.82 | 0.272 |
| insulin glargine | 34 | 42,913 | 1,595 | 1,394,888 | 15 | 23,945 | 1,391 | 1,150,307 | 0.70 | 0.54 | 0.273 |  | 0.308 | 0.885 | 0.376 |  | 0.350 |  | 0.63 | 0.001 |
| sapropterin dihydrochloride | 10 | 42,937 | 486 | 1,395,997 | 0 | 0 | 0 | 0 | 0.70 | 1.00 | -0.354 |  | 2.846 | -0.124 | 0.901 |  |  |  |  |  |
| amoxicillin clavulanate | 12 | 42,935 | 581 | 1,395,902 | 0 | 0 | 0 | 0 | 0.70 | 1.00 | -0.358 |  | 2.843 | -0.126 | 0.900 |  |  |  |  |  |
| rivastigmine | 13 | 42,934 | 635 | 1,395,848 | 14 | 23,946 | 463 | 1,151,235 | 0.69 | 1.50 | -0.778 |  | 0.383 | -2.031 | 0.042 |  | 0.040 |  | 0.93 | 0.699 |
| dasatinib | 39 | 42,908 | 1,870 | 1,394,613 | 30 | 23,930 | 1,903 | 1,149,795 | 0.69 | 0.77 | -0.115 |  | 0.243 | -0.472 | 0.637 |  | 0.650 |  | 0.71 | 0.005 |
| candesartan | 6 | 42,941 | 308 | 1,396,175 | 0 | 0 | 0 | 0 | 0.69 | 1.00 | -0.378 |  | 2.856 | -0.132 | 0.895 |  |  |  |  |  |
| diphenhydramine | 8 | 42,939 | 406 | 1,396,077 | 0 | 0 | 0 | 0 | 0.68 | 1.00 | -0.386 |  | 2.850 | -0.135 | 0.892 |  |  |  |  |  |
| everolimus | 44 | 42,903 | 2,138 | 1,394,345 | 17 | 23,943 | 1,503 | 1,150,195 | 0.68 | 0.56 | 0.190 |  | 0.284 | 0.669 | 0.503 |  | 0.469 |  | 0.63 | <0.001 |
| dexamethasone | 25 | 42,922 | 1,227 | 1,395,256 | 18 | 23,942 | 1,323 | 1,150,375 | 0.68 | 0.67 | 0.005 |  | 0.308 | 0.018 | 0.986 |  | 0.967 |  | 0.66 | 0.006 |
| lapatinib ditosylate | 8 | 42,939 | 410 | 1,396,073 | 0 | 0 | 0 | 0 | 0.67 | 1.00 | -0.396 |  | 2.850 | -0.139 | 0.890 |  |  |  |  |  |
| tezepelumab ekko | 8 | 42,939 | 410 | 1,396,073 | 0 | 0 | 0 | 0 | 0.67 | 1.00 | -0.396 |  | 2.850 | -0.139 | 0.890 |  |  |  |  |  |
| tafamidis | 7 | 42,940 | 362 | 1,396,121 | 21 | 23,939 | 797 | 1,150,901 | 0.67 | 1.30 | -0.656 |  | 0.429 | -1.529 | 0.126 |  | 0.105 |  | 1.01 | 0.951 |
| donepezil | 11 | 42,936 | 556 | 1,395,927 | 7 | 23,953 | 368 | 1,151,330 | 0.67 | 0.98 | -0.376 |  | 0.474 | -0.792 | 0.428 |  | 0.469 |  | 0.73 | 0.180 |
| romosozumab aqqg | 22 | 42,925 | 1,089 | 1,395,394 | 0 | 0 | 0 | 0 | 0.67 | 1.00 | -0.398 |  | 2.836 | -0.140 | 0.888 |  |  |  |  |  |
| itraconazole | 5 | 42,942 | 266 | 1,396,217 | 0 | 0 | 0 | 0 | 0.67 | 1.00 | -0.399 |  | 2.861 | -0.139 | 0.889 |  |  |  |  |  |
| exenatide | 24 | 42,923 | 1,194 | 1,395,289 | 16 | 23,944 | 1,026 | 1,150,672 | 0.67 | 0.77 | -0.147 |  | 0.321 | -0.458 | 0.647 |  | 0.674 |  | 0.69 | 0.019 |
| aprepitant | 5 | 42,942 | 271 | 1,396,212 | 0 | 0 | 0 | 0 | 0.66 | 1.00 | -0.418 |  | 2.861 | -0.146 | 0.884 |  |  |  |  |  |
| celecoxib | 21 | 42,926 | 1,062 | 1,395,421 | 12 | 23,948 | 545 | 1,151,153 | 0.66 | 1.10 | -0.516 |  | 0.360 | -1.433 | 0.152 |  | 0.169 |  | 0.75 | 0.102 |
| alectinib | 32 | 42,915 | 1,614 | 1,394,869 | 11 | 23,949 | 1,214 | 1,150,484 | 0.65 | 0.45 | 0.364 |  | 0.345 | 1.053 | 0.292 |  | 0.261 |  | 0.57 | <0.001 |
| cabozantinib s | 35 | 42,912 | 1,769 | 1,394,714 | 97 | 23,863 | 3,118 | 1,148,580 | 0.65 | 1.50 | -0.836 |  | 0.198 | -4.214 | <0.001 |  | <0.001 |  | 1.11 | 0.240 |
| liraglutide | 36 | 42,911 | 1,821 | 1,394,662 | 14 | 23,946 | 948 | 1,150,750 | 0.65 | 0.73 | -0.120 |  | 0.313 | -0.385 | 0.700 |  | 0.754 |  | 0.66 | 0.003 |
| parathyroid hormone | 10 | 42,937 | 524 | 1,395,959 | 0 | 0 | 0 | 0 | 0.65 | 1.00 | -0.429 |  | 2.846 | -0.151 | 0.880 |  |  |  |  |  |
| naproxen | 24 | 42,923 | 1,238 | 1,395,245 | 7 | 23,953 | 851 | 1,150,847 | 0.64 | 0.42 | 0.418 |  | 0.420 | 0.997 | 0.319 |  | 0.276 |  | 0.56 | <0.001 |
| insulin degludec | 17 | 42,930 | 885 | 1,395,598 | 10 | 23,950 | 590 | 1,151,108 | 0.64 | 0.85 | -0.285 |  | 0.394 | -0.724 | 0.469 |  | 0.507 |  | 0.68 | 0.049 |
| dapagliflozin propanediol | 18 | 42,929 | 938 | 1,395,545 | 15 | 23,945 | 1,000 | 1,150,698 | 0.64 | 0.74 | -0.150 |  | 0.347 | -0.432 | 0.666 |  | 0.682 |  | 0.66 | 0.019 |
| drospirenone ethinyl estradiol | 20 | 42,927 | 1,041 | 1,395,442 | 0 | 0 | 0 | 0 | 0.64 | 1.00 | -0.447 |  | 2.837 | -0.157 | 0.875 |  |  |  |  |  |
| risperidone | 30 | 42,917 | 1,549 | 1,394,934 | 124 | 23,836 | 3,509 | 1,148,189 | 0.64 | 1.71 | -0.982 |  | 0.204 | -4.804 | <0.001 |  | <0.001 |  | 1.28 | 0.002 |
| paricalcitol | 6 | 42,941 | 330 | 1,396,153 | 7 | 23,953 | 369 | 1,151,329 | 0.64 | 0.98 | -0.422 |  | 0.541 | -0.781 | 0.435 |  | 0.437 |  | 0.73 | 0.259 |
| molnupiravir | 17 | 42,930 | 894 | 1,395,589 | 11 | 23,949 | 717 | 1,150,981 | 0.64 | 0.77 | -0.192 |  | 0.383 | -0.500 | 0.617 |  | 0.651 |  | 0.66 | 0.028 |
| avatrombopag | 6 | 42,941 | 332 | 1,396,151 | 5 | 23,955 | 246 | 1,151,452 | 0.64 | 1.07 | -0.523 |  | 0.585 | -0.894 | 0.372 |  | 0.401 |  | 0.72 | 0.275 |
| apomorphine | 7 | 42,940 | 384 | 1,396,099 | 17 | 23,943 | 544 | 1,151,154 | 0.63 | 1.55 | -0.891 |  | 0.442 | -2.017 | 0.044 |  | 0.034 |  | 1.04 | 0.849 |
| lenvatinib | 78 | 42,869 | 4,030 | 1,392,453 | 69 | 23,891 | 4,307 | 1,147,391 | 0.63 | 0.77 | -0.203 |  | 0.166 | -1.219 | 0.223 |  | 0.226 |  | 0.69 | <0.001 |
| sorafenib | 7 | 42,940 | 389 | 1,396,094 | 8 | 23,952 | 885 | 1,150,813 | 0.63 | 0.46 | 0.306 |  | 0.505 | 0.605 | 0.545 |  | 0.566 |  | 0.49 | 0.006 |
| clonidine | 8 | 42,939 | 441 | 1,396,042 | 6 | 23,954 | 276 | 1,151,422 | 0.63 | 1.13 | -0.591 |  | 0.527 | -1.121 | 0.262 |  | 0.288 |  | 0.73 | 0.233 |
| hydroxychloroquine | 11 | 42,936 | 599 | 1,395,884 | 0 | 0 | 0 | 0 | 0.62 | 1.00 | -0.472 |  | 2.844 | -0.166 | 0.868 |  |  |  |  |  |
| belumosudil | 6 | 42,941 | 340 | 1,396,143 | 7 | 23,953 | 387 | 1,151,311 | 0.62 | 0.93 | -0.405 |  | 0.541 | -0.748 | 0.454 |  | 0.456 |  | 0.70 | 0.205 |
| ubrogepant | 5 | 42,942 | 288 | 1,396,195 | 0 | 0 | 0 | 0 | 0.62 | 1.00 | -0.478 |  | 2.861 | -0.167 | 0.867 |  |  |  |  |  |
| irbesartan | 9 | 42,938 | 511 | 1,395,972 | 0 | 0 | 0 | 0 | 0.60 | 1.00 | -0.504 |  | 2.847 | -0.177 | 0.859 |  |  |  |  |  |
| zoledronic acid | 51 | 42,896 | 2,787 | 1,393,696 | 0 | 0 | 0 | 0 | 0.60 | 1.00 | -0.510 |  | 2.832 | -0.180 | 0.857 |  |  |  |  |  |
| abobotulinumtoxina | 6 | 42,941 | 354 | 1,396,129 | 0 | 0 | 0 | 0 | 0.60 | 1.00 | -0.517 |  | 2.856 | -0.181 | 0.856 |  |  |  |  |  |
| glutamine | 10 | 42,937 | 574 | 1,395,909 | 0 | 0 | 0 | 0 | 0.59 | 1.00 | -0.521 |  | 2.846 | -0.183 | 0.855 |  |  |  |  |  |
| glatiramer | 48 | 42,899 | 2,661 | 1,393,822 | 16 | 23,944 | 1,070 | 1,150,628 | 0.59 | 0.74 | -0.224 |  | 0.287 | -0.779 | 0.436 |  | 0.483 |  | 0.61 | <0.001 |
| brodalumab | 8 | 42,939 | 468 | 1,396,015 | 7 | 23,953 | 581 | 1,151,117 | 0.59 | 0.62 | -0.050 |  | 0.505 | -0.098 | 0.922 |  | 0.937 |  | 0.57 | 0.027 |
| pantoprazole | 31 | 42,916 | 1,736 | 1,394,747 | 21 | 23,939 | 1,451 | 1,150,247 | 0.59 | 0.71 | -0.188 |  | 0.282 | -0.668 | 0.504 |  | 0.525 |  | 0.62 | <0.001 |
| thalidomide | 16 | 42,931 | 914 | 1,395,569 | 9 | 23,951 | 978 | 1,150,720 | 0.59 | 0.47 | 0.229 |  | 0.410 | 0.559 | 0.576 |  | 0.546 |  | 0.52 | <0.001 |
| spironolactone | 11 | 42,936 | 638 | 1,395,845 | 0 | 0 | 0 | 0 | 0.59 | 1.00 | -0.535 |  | 2.844 | -0.188 | 0.851 |  |  |  |  |  |
| fostamatinib | 13 | 42,934 | 750 | 1,395,733 | 11 | 23,949 | 589 | 1,151,109 | 0.58 | 0.94 | -0.472 |  | 0.405 | -1.165 | 0.244 |  | 0.256 |  | 0.68 | 0.060 |
| lamotrigine | 27 | 42,920 | 1,528 | 1,394,955 | 21 | 23,939 | 674 | 1,151,024 | 0.58 | 1.53 | -0.964 |  | 0.292 | -3.303 | <0.001 |  | <0.001 |  | 0.79 | 0.102 |
| tenofovir alafenamide | 6 | 42,941 | 364 | 1,396,119 | 0 | 0 | 0 | 0 | 0.58 | 1.00 | -0.545 |  | 2.856 | -0.191 | 0.849 |  |  |  |  |  |
| diltiazem | 10 | 42,937 | 592 | 1,395,891 | 5 | 23,955 | 356 | 1,151,342 | 0.58 | 0.74 | -0.252 |  | 0.531 | -0.475 | 0.634 |  | 0.708 |  | 0.59 | 0.038 |
| phenytoin | 6 | 42,941 | 368 | 1,396,115 | 8 | 23,952 | 431 | 1,151,267 | 0.57 | 0.95 | -0.501 |  | 0.526 | -0.953 | 0.340 |  | 0.334 |  | 0.69 | 0.168 |
| riociguat | 17 | 42,930 | 992 | 1,395,491 | 8 | 23,952 | 657 | 1,151,041 | 0.57 | 0.62 | -0.081 |  | 0.421 | -0.191 | 0.848 |  | 0.909 |  | 0.57 | 0.004 |
| tadalafil | 22 | 42,925 | 1,278 | 1,395,205 | 12 | 23,948 | 1,246 | 1,150,452 | 0.57 | 0.48 | 0.172 |  | 0.355 | 0.484 | 0.629 |  | 0.598 |  | 0.52 | <0.001 |
| lanadelumab | 11 | 42,936 | 654 | 1,395,829 | 0 | 0 | 0 | 0 | 0.57 | 1.00 | -0.560 |  | 2.844 | -0.197 | 0.844 |  |  |  |  |  |
| dornase alfa | 8 | 42,939 | 486 | 1,395,997 | 6 | 23,954 | 404 | 1,151,294 | 0.57 | 0.77 | -0.307 |  | 0.525 | -0.585 | 0.559 |  | 0.596 |  | 0.60 | 0.055 |
| denosumab | 57 | 42,890 | 3,337 | 1,393,146 | 10 | 23,950 | 1,041 | 1,150,657 | 0.56 | 0.48 | 0.144 |  | 0.338 | 0.428 | 0.669 |  | 0.593 |  | 0.54 | <0.001 |
| azithromycin | 17 | 42,930 | 1,018 | 1,395,465 | 14 | 23,946 | 777 | 1,150,921 | 0.56 | 0.90 | -0.473 |  | 0.358 | -1.320 | 0.187 |  | 0.196 |  | 0.65 | 0.018 |
| zolpidem | 10 | 42,937 | 617 | 1,395,866 | 10 | 23,950 | 453 | 1,151,245 | 0.55 | 1.11 | -0.700 |  | 0.441 | -1.587 | 0.112 |  | 0.114 |  | 0.71 | 0.120 |
| olaparib | 40 | 42,907 | 2,403 | 1,394,080 | 9 | 23,951 | 736 | 1,150,962 | 0.55 | 0.62 | -0.124 |  | 0.363 | -0.342 | 0.732 |  | 0.823 |  | 0.55 | <0.001 |
| insulin human | 17 | 42,930 | 1,042 | 1,395,441 | 9 | 23,951 | 898 | 1,150,800 | 0.55 | 0.51 | 0.071 |  | 0.406 | 0.176 | 0.860 |  | 0.816 |  | 0.51 | <0.001 |
| encorafenib | 17 | 42,930 | 1,047 | 1,395,436 | 13 | 23,947 | 1,084 | 1,150,614 | 0.54 | 0.60 | -0.097 |  | 0.365 | -0.265 | 0.791 |  | 0.813 |  | 0.55 | <0.001 |
| hyaluronidase zzxf pertuzumab trastuzumab | 8 | 42,939 | 509 | 1,395,974 | 0 | 0 | 0 | 0 | 0.54 | 1.00 | -0.612 |  | 2.849 | -0.215 | 0.830 |  |  |  |  |  |
| clindamycin | 12 | 42,935 | 749 | 1,395,734 | 0 | 0 | 0 | 0 | 0.54 | 1.00 | -0.612 |  | 2.843 | -0.215 | 0.829 |  |  |  |  |  |
| venetoclax | 65 | 42,882 | 3,952 | 1,392,531 | 73 | 23,887 | 5,508 | 1,146,190 | 0.54 | 0.64 | -0.174 |  | 0.171 | -1.014 | 0.311 |  | 0.310 |  | 0.58 | <0.001 |
| peginterferon beta 1a | 14 | 42,933 | 878 | 1,395,605 | 5 | 23,955 | 253 | 1,151,445 | 0.54 | 1.04 | -0.665 |  | 0.506 | -1.314 | 0.189 |  | 0.242 |  | 0.59 | 0.021 |
| budesonide formoterol glycopyrrolate | 5 | 42,942 | 336 | 1,396,147 | 0 | 0 | 0 | 0 | 0.53 | 1.00 | -0.632 |  | 2.861 | -0.221 | 0.825 |  |  |  |  |  |
| olanzapine | 22 | 42,925 | 1,380 | 1,395,103 | 42 | 23,918 | 1,395 | 1,150,303 | 0.53 | 1.46 | -1.017 |  | 0.264 | -3.858 | <0.001 |  | <0.001 |  | 0.90 | 0.392 |
| sitagliptin | 19 | 42,928 | 1,199 | 1,395,284 | 17 | 23,943 | 1,217 | 1,150,481 | 0.53 | 0.69 | -0.268 |  | 0.332 | -0.807 | 0.420 |  | 0.430 |  | 0.58 | <0.001 |
| cyclophosphamide | 27 | 42,920 | 1,696 | 1,394,787 | 5 | 23,955 | 1,175 | 1,150,523 | 0.53 | 0.22 | 0.852 |  | 0.469 | 1.818 | 0.069 |  | 0.049 |  | 0.42 | <0.001 |
| medroxyprogesterone | 10 | 42,937 | 650 | 1,395,833 | 0 | 0 | 0 | 0 | 0.52 | 1.00 | -0.645 |  | 2.845 | -0.227 | 0.821 |  |  |  |  |  |
| metformin sitagliptin | 10 | 42,937 | 655 | 1,395,828 | 11 | 23,949 | 683 | 1,151,015 | 0.52 | 0.81 | -0.440 |  | 0.430 | -1.023 | 0.307 |  | 0.309 |  | 0.61 | 0.024 |
| ramipril | 13 | 42,934 | 846 | 1,395,637 | 5 | 23,955 | 891 | 1,150,807 | 0.52 | 0.30 | 0.559 |  | 0.508 | 1.100 | 0.271 |  | 0.236 |  | 0.40 | <0.001 |
| bisoprolol | 10 | 42,937 | 663 | 1,395,820 | 5 | 23,955 | 675 | 1,151,023 | 0.51 | 0.39 | 0.274 |  | 0.529 | 0.517 | 0.605 |  | 0.559 |  | 0.44 | <0.001 |
| insulin lispro | 20 | 42,927 | 1,311 | 1,395,172 | 13 | 23,947 | 1,203 | 1,150,495 | 0.51 | 0.54 | -0.059 |  | 0.353 | -0.167 | 0.867 |  | 0.898 |  | 0.50 | <0.001 |
| romiplostim | 5 | 42,942 | 355 | 1,396,128 | 0 | 0 | 0 | 0 | 0.50 | 1.00 | -0.687 |  | 2.861 | -0.240 | 0.810 |  |  |  |  |  |
| interferon beta 1b | 9 | 42,938 | 615 | 1,395,868 | 0 | 0 | 0 | 0 | 0.50 | 1.00 | -0.690 |  | 2.847 | -0.242 | 0.809 |  |  |  |  |  |
| ascorbic acid polyethylene glycol 3350 chloride ascorbate chloride | 5 | 42,942 | 357 | 1,396,126 | 0 | 0 | 0 | 0 | 0.50 | 1.00 | -0.693 |  | 2.861 | -0.242 | 0.809 |  |  |  |  |  |
| cilastatin imipenem | 8 | 42,939 | 552 | 1,395,931 | 12 | 23,948 | 690 | 1,151,008 | 0.50 | 0.87 | -0.554 |  | 0.448 | -1.235 | 0.217 |  | 0.207 |  | 0.64 | 0.045 |
| bedaquiline | 7 | 42,940 | 489 | 1,395,994 | 6 | 23,954 | 690 | 1,151,008 | 0.50 | 0.45 | 0.096 |  | 0.539 | 0.179 | 0.858 |  | 0.847 |  | 0.44 | 0.003 |
| imatinib | 26 | 42,921 | 1,755 | 1,394,728 | 20 | 23,940 | 1,889 | 1,149,809 | 0.49 | 0.52 | -0.060 |  | 0.296 | -0.204 | 0.838 |  | 0.855 |  | 0.49 | <0.001 |
| osimertinib | 39 | 42,908 | 2,648 | 1,393,835 | 21 | 23,939 | 1,827 | 1,149,871 | 0.48 | 0.57 | -0.154 |  | 0.270 | -0.570 | 0.568 |  | 0.599 |  | 0.50 | <0.001 |
| fluconazole | 9 | 42,938 | 644 | 1,395,839 | 6 | 23,954 | 519 | 1,151,179 | 0.48 | 0.60 | -0.227 |  | 0.512 | -0.443 | 0.658 |  | 0.704 |  | 0.49 | 0.005 |
| canakinumab | 16 | 42,931 | 1,119 | 1,395,364 | 11 | 23,949 | 879 | 1,150,819 | 0.48 | 0.63 | -0.271 |  | 0.387 | -0.701 | 0.483 |  | 0.512 |  | 0.51 | <0.001 |
| droxidopa | 14 | 42,933 | 994 | 1,395,489 | 19 | 23,941 | 868 | 1,150,830 | 0.47 | 1.08 | -0.823 |  | 0.350 | -2.352 | 0.019 |  | 0.016 |  | 0.68 | 0.027 |
| oxcarbazepine | 6 | 42,941 | 453 | 1,396,030 | 7 | 23,953 | 412 | 1,151,286 | 0.47 | 0.87 | -0.629 |  | 0.540 | -1.164 | 0.244 |  | 0.246 |  | 0.58 | 0.047 |
| human c1 esterase inhibitor | 23 | 42,924 | 1,662 | 1,394,821 | 0 | 0 | 0 | 0 | 0.46 | 1.00 | -0.778 |  | 2.836 | -0.274 | 0.784 |  |  |  |  |  |
| enasidenib | 9 | 42,938 | 674 | 1,395,809 | 9 | 23,951 | 871 | 1,150,827 | 0.46 | 0.52 | -0.135 |  | 0.462 | -0.291 | 0.771 |  | 0.777 |  | 0.46 | <0.001 |
| tobramycin | 7 | 42,940 | 535 | 1,395,948 | 0 | 0 | 0 | 0 | 0.46 | 1.00 | -0.787 |  | 2.852 | -0.276 | 0.783 |  |  |  |  |  |
| pembrolizumab | 46 | 42,901 | 3,317 | 1,393,166 | 37 | 23,923 | 3,475 | 1,148,223 | 0.46 | 0.52 | -0.129 |  | 0.221 | -0.584 | 0.559 |  | 0.569 |  | 0.48 | <0.001 |
| pazopanib | 15 | 42,932 | 1,115 | 1,395,368 | 17 | 23,943 | 1,414 | 1,150,284 | 0.45 | 0.59 | -0.275 |  | 0.351 | -0.782 | 0.434 |  | 0.434 |  | 0.50 | <0.001 |
| esomeprazole | 45 | 42,902 | 3,284 | 1,393,199 | 18 | 23,942 | 2,700 | 1,148,998 | 0.45 | 0.33 | 0.314 |  | 0.277 | 1.132 | 0.258 |  | 0.237 |  | 0.40 | <0.001 |
| efavirenz emtricitabine tenofovir disoproxil | 11 | 42,936 | 831 | 1,395,652 | 40 | 23,920 | 1,724 | 1,149,974 | 0.45 | 1.13 | -0.921 |  | 0.337 | -2.733 | 0.006 |  | 0.004 |  | 0.83 | 0.192 |
| acetaminophen | 40 | 42,907 | 2,948 | 1,393,535 | 12 | 23,948 | 1,943 | 1,149,755 | 0.45 | 0.31 | 0.368 |  | 0.325 | 1.132 | 0.258 |  | 0.228 |  | 0.40 | <0.001 |
| cyclosporine | 15 | 42,932 | 1,130 | 1,395,353 | 11 | 23,949 | 1,254 | 1,150,444 | 0.45 | 0.44 | 0.012 |  | 0.391 | 0.030 | 0.976 |  | 0.953 |  | 0.43 | <0.001 |
| tolvaptan | 15 | 42,932 | 1,133 | 1,395,350 | 26 | 23,934 | 1,052 | 1,150,646 | 0.44 | 1.21 | -1.002 |  | 0.323 | -3.105 | 0.002 |  | 0.001 |  | 0.72 | 0.040 |
| ezetimibe | 8 | 42,939 | 622 | 1,395,861 | 6 | 23,954 | 572 | 1,151,126 | 0.44 | 0.55 | -0.206 |  | 0.524 | -0.394 | 0.694 |  | 0.730 |  | 0.45 | 0.002 |
| ustekinumab | 98 | 42,849 | 7,254 | 1,389,229 | 59 | 23,901 | 6,465 | 1,145,233 | 0.44 | 0.44 | -0.002 |  | 0.165 | -0.010 | 0.992 |  | 0.992 |  | 0.44 | <0.001 |
| tacrolimus | 28 | 42,919 | 2,121 | 1,394,362 | 19 | 23,941 | 2,292 | 1,149,406 | 0.44 | 0.41 | 0.066 |  | 0.296 | 0.225 | 0.822 |  | 0.802 |  | 0.42 | <0.001 |
| amiodarone | 10 | 42,937 | 785 | 1,395,698 | 15 | 23,945 | 1,111 | 1,150,587 | 0.43 | 0.67 | -0.433 |  | 0.402 | -1.076 | 0.282 |  | 0.271 |  | 0.53 | 0.001 |
| ranitidine | 14 | 42,933 | 1,086 | 1,395,397 | 9 | 23,951 | 910 | 1,150,788 | 0.43 | 0.50 | -0.145 |  | 0.420 | -0.345 | 0.730 |  | 0.769 |  | 0.44 | <0.001 |
| dapagliflozin | 18 | 42,929 | 1,398 | 1,395,085 | 16 | 23,944 | 1,465 | 1,150,233 | 0.43 | 0.54 | -0.230 |  | 0.341 | -0.674 | 0.500 |  | 0.512 |  | 0.46 | <0.001 |
| gadoterate meglumine | 9 | 42,938 | 731 | 1,395,752 | 6 | 23,954 | 331 | 1,151,367 | 0.42 | 0.94 | -0.803 |  | 0.513 | -1.564 | 0.118 |  | 0.134 |  | 0.51 | 0.009 |
| trastuzumab | 43 | 42,904 | 3,367 | 1,393,116 | 0 | 0 | 0 | 0 | 0.42 | 1.00 | -0.869 |  | 2.833 | -0.307 | 0.759 |  |  |  |  |  |
| metformin | 34 | 42,913 | 2,704 | 1,393,779 | 20 | 23,940 | 2,309 | 1,149,389 | 0.41 | 0.43 | -0.028 |  | 0.280 | -0.100 | 0.920 |  | 0.949 |  | 0.41 | <0.001 |
| lansoprazole | 14 | 42,933 | 1,158 | 1,395,325 | 0 | 0 | 0 | 0 | 0.41 | 1.00 | -0.900 |  | 2.841 | -0.317 | 0.752 |  |  |  |  |  |
| insulin detemir | 9 | 42,938 | 765 | 1,395,718 | 5 | 23,955 | 513 | 1,151,185 | 0.40 | 0.51 | -0.244 |  | 0.539 | -0.452 | 0.651 |  | 0.717 |  | 0.41 | <0.001 |
| warfarin | 7 | 42,940 | 606 | 1,395,877 | 0 | 0 | 0 | 0 | 0.40 | 1.00 | -0.911 |  | 2.852 | -0.320 | 0.749 |  |  |  |  |  |
| docetaxel | 28 | 42,919 | 2,332 | 1,394,151 | 0 | 0 | 0 | 0 | 0.40 | 1.00 | -0.924 |  | 2.835 | -0.326 | 0.744 |  |  |  |  |  |
| rivaroxaban | 87 | 42,860 | 7,150 | 1,389,333 | 54 | 23,906 | 7,412 | 1,144,286 | 0.40 | 0.35 | 0.120 |  | 0.174 | 0.690 | 0.490 |  | 0.479 |  | 0.38 | <0.001 |
| mesalamine | 7 | 42,940 | 618 | 1,395,865 | 0 | 0 | 0 | 0 | 0.39 | 1.00 | -0.931 |  | 2.852 | -0.326 | 0.744 |  |  |  |  |  |
| axicabtagene ciloleucel | 12 | 42,935 | 1,034 | 1,395,449 | 6 | 23,954 | 1,431 | 1,150,267 | 0.39 | 0.22 | 0.588 |  | 0.485 | 1.212 | 0.225 |  | 0.203 |  | 0.29 | <0.001 |
| ivacaftor | 6 | 42,941 | 539 | 1,395,944 | 5 | 23,955 | 410 | 1,151,288 | 0.39 | 0.64 | -0.497 |  | 0.583 | -0.853 | 0.394 |  | 0.424 |  | 0.44 | 0.005 |
| emtricitabine tenofovir disoproxil | 18 | 42,929 | 1,536 | 1,394,947 | 49 | 23,911 | 2,747 | 1,148,951 | 0.39 | 0.87 | -0.794 |  | 0.274 | -2.894 | 0.004 |  | 0.003 |  | 0.64 | <0.001 |
| tralokinumab ldrm | 5 | 42,942 | 459 | 1,396,024 | 0 | 0 | 0 | 0 | 0.39 | 1.00 | -0.944 |  | 2.861 | -0.330 | 0.741 |  |  |  |  |  |
| fulvestrant | 25 | 42,922 | 2,134 | 1,394,349 | 0 | 0 | 0 | 0 | 0.39 | 1.00 | -0.947 |  | 2.835 | -0.334 | 0.739 |  |  |  |  |  |
| sirolimus | 6 | 42,941 | 546 | 1,395,937 | 5 | 23,955 | 667 | 1,151,031 | 0.39 | 0.40 | -0.024 |  | 0.582 | -0.041 | 0.968 |  | 0.989 |  | 0.36 | <0.001 |
| perampanel | 5 | 42,942 | 465 | 1,396,018 | 8 | 23,952 | 444 | 1,151,254 | 0.38 | 0.92 | -0.872 |  | 0.551 | -1.583 | 0.114 |  | 0.103 |  | 0.55 | 0.031 |
| golimumab | 92 | 42,855 | 7,834 | 1,388,649 | 18 | 23,942 | 4,402 | 1,147,296 | 0.38 | 0.20 | 0.642 |  | 0.256 | 2.512 | 0.012 |  | 0.009 |  | 0.33 | <0.001 |
| pertuzumab | 16 | 42,931 | 1,424 | 1,395,059 | 0 | 0 | 0 | 0 | 0.38 | 1.00 | -0.977 |  | 2.839 | -0.344 | 0.731 |  |  |  |  |  |
| mycophenolate mofetil | 13 | 42,934 | 1,175 | 1,395,308 | 12 | 23,948 | 1,117 | 1,150,581 | 0.37 | 0.54 | -0.365 |  | 0.395 | -0.923 | 0.356 |  | 0.367 |  | 0.42 | <0.001 |
| risdiplam | 5 | 42,942 | 480 | 1,396,003 | 0 | 0 | 0 | 0 | 0.37 | 1.00 | -0.989 |  | 2.861 | -0.346 | 0.730 |  |  |  |  |  |
| insulin aspart | 22 | 42,925 | 1,964 | 1,394,519 | 7 | 23,953 | 1,532 | 1,150,166 | 0.37 | 0.23 | 0.460 |  | 0.423 | 1.086 | 0.277 |  | 0.240 |  | 0.31 | <0.001 |
| crizotinib | 10 | 42,937 | 931 | 1,395,552 | 7 | 23,953 | 877 | 1,150,821 | 0.37 | 0.41 | -0.114 |  | 0.480 | -0.237 | 0.812 |  | 0.850 |  | 0.36 | <0.001 |
| hydrochlorothiazide | 5 | 42,942 | 491 | 1,395,992 | 0 | 0 | 0 | 0 | 0.36 | 1.00 | -1.011 |  | 2.861 | -0.353 | 0.724 |  |  |  |  |  |
| valproate | 7 | 42,940 | 670 | 1,395,813 | 6 | 23,954 | 756 | 1,150,942 | 0.36 | 0.41 | -0.127 |  | 0.539 | -0.236 | 0.814 |  | 0.836 |  | 0.36 | <0.001 |
| deferasirox | 7 | 42,940 | 675 | 1,395,808 | 7 | 23,953 | 693 | 1,151,005 | 0.36 | 0.52 | -0.365 |  | 0.519 | -0.702 | 0.483 |  | 0.495 |  | 0.40 | <0.001 |
| trametinib dimethyl sulfoxide | 9 | 42,938 | 873 | 1,395,610 | 10 | 23,950 | 842 | 1,150,856 | 0.35 | 0.60 | -0.527 |  | 0.450 | -1.170 | 0.242 |  | 0.244 |  | 0.43 | <0.001 |
| axitinib | 9 | 42,938 | 884 | 1,395,599 | 33 | 23,927 | 1,559 | 1,150,139 | 0.35 | 1.03 | -1.084 |  | 0.370 | -2.930 | 0.003 |  | 0.002 |  | 0.71 | 0.024 |
| tipiracil trifluridine | 12 | 42,935 | 1,170 | 1,395,313 | 10 | 23,950 | 1,468 | 1,150,230 | 0.35 | 0.34 | 0.011 |  | 0.421 | 0.025 | 0.980 |  | 0.965 |  | 0.33 | <0.001 |
| ado trastuzumab emtansine | 9 | 42,938 | 892 | 1,395,591 | 0 | 0 | 0 | 0 | 0.35 | 1.00 | -1.061 |  | 2.847 | -0.373 | 0.709 |  |  |  |  |  |
| daratumumab | 17 | 42,930 | 1,655 | 1,394,828 | 9 | 23,951 | 2,018 | 1,149,680 | 0.34 | 0.23 | 0.419 |  | 0.404 | 1.036 | 0.300 |  | 0.279 |  | 0.28 | <0.001 |
| valacyclovir | 6 | 42,941 | 615 | 1,395,868 | 0 | 0 | 0 | 0 | 0.34 | 1.00 | -1.069 |  | 2.856 | -0.374 | 0.708 |  |  |  |  |  |
| ivacaftor lumacaftor | 6 | 42,941 | 623 | 1,395,860 | 5 | 23,955 | 594 | 1,151,104 | 0.34 | 0.44 | -0.271 |  | 0.582 | -0.466 | 0.641 |  | 0.673 |  | 0.35 | <0.001 |
| ondansetron | 7 | 42,940 | 739 | 1,395,744 | 6 | 23,954 | 385 | 1,151,313 | 0.33 | 0.81 | -0.899 |  | 0.540 | -1.667 | 0.096 |  | 0.101 |  | 0.42 | 0.001 |
| tenofovir disoproxil | 13 | 42,934 | 1,343 | 1,395,140 | 33 | 23,927 | 2,195 | 1,149,503 | 0.33 | 0.73 | -0.809 |  | 0.324 | -2.493 | 0.013 |  | 0.010 |  | 0.53 | <0.001 |
| sacituzumab govitecan hziy | 6 | 42,941 | 656 | 1,395,827 | 0 | 0 | 0 | 0 | 0.32 | 1.00 | -1.134 |  | 2.856 | -0.397 | 0.691 |  |  |  |  |  |
| bortezomib | 8 | 42,939 | 859 | 1,395,624 | 13 | 23,947 | 1,186 | 1,150,512 | 0.32 | 0.55 | -0.531 |  | 0.440 | -1.206 | 0.228 |  | 0.215 |  | 0.41 | <0.001 |
| chloride | 12 | 42,935 | 1,315 | 1,395,168 | 6 | 23,954 | 776 | 1,150,922 | 0.31 | 0.40 | -0.264 |  | 0.486 | -0.544 | 0.586 |  | 0.653 |  | 0.32 | <0.001 |
| darbepoetin alfa | 5 | 42,942 | 579 | 1,395,904 | 5 | 23,955 | 641 | 1,151,057 | 0.31 | 0.41 | -0.289 |  | 0.606 | -0.477 | 0.633 |  | 0.648 |  | 0.32 | <0.001 |
| nivolumab | 40 | 42,907 | 4,268 | 1,392,215 | 76 | 23,884 | 6,167 | 1,145,531 | 0.31 | 0.59 | -0.659 |  | 0.196 | -3.369 | <0.001 |  | <0.001 |  | 0.45 | <0.001 |
| dabrafenib | 13 | 42,934 | 1,457 | 1,395,026 | 17 | 23,943 | 1,574 | 1,150,124 | 0.30 | 0.53 | -0.573 |  | 0.364 | -1.574 | 0.115 |  | 0.111 |  | 0.39 | <0.001 |
| gemcitabine | 8 | 42,939 | 921 | 1,395,562 | 0 | 0 | 0 | 0 | 0.30 | 1.00 | -1.205 |  | 2.849 | -0.423 | 0.672 |  |  |  |  |  |
| ibuprofen | 25 | 42,922 | 2,767 | 1,393,716 | 20 | 23,940 | 2,267 | 1,149,431 | 0.30 | 0.43 | -0.372 |  | 0.298 | -1.248 | 0.212 |  | 0.221 |  | 0.34 | <0.001 |
| palivizumab | 6 | 42,941 | 708 | 1,395,775 | 13 | 23,947 | 763 | 1,150,935 | 0.30 | 0.85 | -1.047 |  | 0.480 | -2.180 | 0.029 |  | 0.021 |  | 0.51 | 0.003 |
| durvalumab | 11 | 42,936 | 1,263 | 1,395,220 | 9 | 23,951 | 1,910 | 1,149,788 | 0.30 | 0.24 | 0.214 |  | 0.440 | 0.487 | 0.626 |  | 0.619 |  | 0.25 | <0.001 |
| eculizumab | 10 | 42,937 | 1,170 | 1,395,313 | 16 | 23,944 | 776 | 1,150,922 | 0.29 | 1.02 | -1.254 |  | 0.398 | -3.154 | 0.002 |  | <0.001 |  | 0.50 | <0.001 |
| fluorouracil | 6 | 42,941 | 740 | 1,395,743 | 0 | 0 | 0 | 0 | 0.29 | 1.00 | -1.254 |  | 2.856 | -0.439 | 0.661 |  |  |  |  |  |
| ixekizumab | 17 | 42,930 | 1,996 | 1,394,487 | 8 | 23,952 | 1,717 | 1,149,981 | 0.28 | 0.24 | 0.181 |  | 0.419 | 0.431 | 0.666 |  | 0.621 |  | 0.26 | <0.001 |
| carbamazepine | 8 | 42,939 | 983 | 1,395,500 | 11 | 23,949 | 846 | 1,150,852 | 0.28 | 0.65 | -0.843 |  | 0.455 | -1.854 | 0.064 |  | 0.058 |  | 0.40 | <0.001 |
| ferric carboxymaltose | 11 | 42,936 | 1,345 | 1,395,138 | 0 | 0 | 0 | 0 | 0.28 | 1.00 | -1.281 |  | 2.844 | -0.450 | 0.652 |  |  |  |  |  |
| amoxicillin | 11 | 42,936 | 1,420 | 1,395,063 | 6 | 23,954 | 1,025 | 1,150,673 | 0.26 | 0.30 | -0.146 |  | 0.492 | -0.297 | 0.766 |  | 0.828 |  | 0.26 | <0.001 |
| iron sucrose | 5 | 42,942 | 737 | 1,395,746 | 0 | 0 | 0 | 0 | 0.24 | 1.00 | -1.417 |  | 2.861 | -0.495 | 0.620 |  |  |  |  |  |
| agalsidase beta | 5 | 42,942 | 743 | 1,395,740 | 5 | 23,955 | 876 | 1,150,822 | 0.24 | 0.30 | -0.226 |  | 0.605 | -0.374 | 0.709 |  | 0.721 |  | 0.24 | <0.001 |
| gefitinib | 5 | 42,942 | 773 | 1,395,710 | 0 | 0 | 0 | 0 | 0.23 | 1.00 | -1.465 |  | 2.861 | -0.512 | 0.609 |  |  |  |  |  |
| paclitaxel | 22 | 42,925 | 3,394 | 1,393,089 | 0 | 0 | 0 | 0 | 0.22 | 1.00 | -1.537 |  | 2.836 | -0.542 | 0.588 |  |  |  |  |  |
| guselkumab | 14 | 42,933 | 2,211 | 1,394,272 | 8 | 23,952 | 1,889 | 1,149,809 | 0.21 | 0.22 | -0.014 |  | 0.433 | -0.033 | 0.974 |  | 0.980 |  | 0.20 | <0.001 |
| furosemide | 7 | 42,940 | 1,163 | 1,395,320 | 5 | 23,955 | 1,100 | 1,150,598 | 0.21 | 0.24 | -0.136 |  | 0.563 | -0.242 | 0.809 |  | 0.851 |  | 0.20 | <0.001 |
| bevacizumab | 15 | 42,932 | 2,404 | 1,394,079 | 14 | 23,946 | 2,119 | 1,149,579 | 0.21 | 0.33 | -0.450 |  | 0.367 | -1.229 | 0.219 |  | 0.226 |  | 0.25 | <0.001 |
| clozapine | 23 | 42,924 | 3,657 | 1,392,826 | 37 | 23,923 | 4,572 | 1,147,126 | 0.21 | 0.39 | -0.635 |  | 0.264 | -2.402 | 0.016 |  | 0.014 |  | 0.29 | <0.001 |
| atezolizumab | 15 | 42,932 | 2,636 | 1,393,847 | 13 | 23,947 | 2,988 | 1,148,710 | 0.19 | 0.22 | -0.127 |  | 0.373 | -0.340 | 0.734 |  | 0.748 |  | 0.20 | <0.001 |
| pegfilgrastim | 5 | 42,942 | 944 | 1,395,539 | 0 | 0 | 0 | 0 | 0.19 | 1.00 | -1.665 |  | 2.861 | -0.582 | 0.561 |  |  |  |  |  |
| fam trastuzumab deruxtecan nxki | 6 | 42,941 | 1,120 | 1,395,363 | 0 | 0 | 0 | 0 | 0.19 | 1.00 | -1.669 |  | 2.856 | -0.584 | 0.559 |  |  |  |  |  |
| aztreonam lysine | 5 | 42,942 | 956 | 1,395,527 | 0 | 0 | 0 | 0 | 0.19 | 1.00 | -1.677 |  | 2.861 | -0.586 | 0.558 |  |  |  |  |  |
| doxorubicin | 7 | 42,940 | 1,338 | 1,395,145 | 0 | 0 | 0 | 0 | 0.18 | 1.00 | -1.703 |  | 2.852 | -0.597 | 0.550 |  |  |  |  |  |
| obinutuzumab | 6 | 42,941 | 1,188 | 1,395,295 | 7 | 23,953 | 1,538 | 1,150,160 | 0.18 | 0.23 | -0.276 |  | 0.537 | -0.513 | 0.608 |  | 0.606 |  | 0.19 | <0.001 |
| pentosan polysulfate | 5 | 42,942 | 1,024 | 1,395,459 | 0 | 0 | 0 | 0 | 0.17 | 1.00 | -1.746 |  | 2.861 | -0.610 | 0.542 |  |  |  |  |  |
| clopidogrel bisulfate | 5 | 42,942 | 1,116 | 1,395,367 | 7 | 23,953 | 1,619 | 1,150,079 | 0.16 | 0.22 | -0.329 |  | 0.563 | -0.584 | 0.559 |  | 0.543 |  | 0.18 | <0.001 |
| temozolomide | 5 | 42,942 | 1,135 | 1,395,348 | 0 | 0 | 0 | 0 | 0.16 | 1.00 | -1.849 |  | 2.861 | -0.646 | 0.518 |  |  |  |  |  |
| carboplatin | 9 | 42,938 | 2,224 | 1,394,259 | 11 | 23,949 | 1,403 | 1,150,295 | 0.14 | 0.39 | -1.043 |  | 0.440 | -2.372 | 0.018 |  | 0.015 |  | 0.21 | <0.001 |
| empagliflozin | 7 | 42,940 | 1,812 | 1,394,671 | 13 | 23,947 | 2,361 | 1,149,337 | 0.13 | 0.27 | -0.714 |  | 0.457 | -1.563 | 0.118 |  | 0.105 |  | 0.19 | <0.001 |
| dabigatran etexilate | 6 | 42,941 | 1,730 | 1,394,753 | 0 | 0 | 0 | 0 | 0.12 | 1.00 | -2.104 |  | 2.856 | -0.737 | 0.461 |  |  |  |  |  |
| baricitinib | 6 | 42,941 | 1,753 | 1,394,730 | 0 | 0 | 0 | 0 | 0.12 | 1.00 | -2.117 |  | 2.856 | -0.741 | 0.458 |  |  |  |  |  |
| dexmethylphenidate serdexmethylphenidate chloride | 0 | 0 | 0 | 0 | 8 | 23,952 | 44 | 1,151,654 | 1.00 | 9.18 | -2.217 |  | 2.853 | -0.777 | 0.437 |  |  |  |  |  |
| finasteride | 0 | 0 | 0 | 0 | 160 | 23,800 | 1,064 | 1,150,634 | 1.00 | 7.29 | -1.986 |  | 2.830 | -0.702 | 0.483 |  |  |  |  |  |
| relugolix | 0 | 0 | 0 | 0 | 53 | 23,907 | 485 | 1,151,213 | 1.00 | 5.31 | -1.669 |  | 2.832 | -0.589 | 0.556 |  |  |  |  |  |
| buspirone | 0 | 0 | 0 | 0 | 7 | 23,953 | 85 | 1,151,613 | 1.00 | 4.22 | -1.439 |  | 2.854 | -0.504 | 0.614 |  |  |  |  |  |
| maralixibat chloride | 0 | 0 | 0 | 0 | 5 | 23,955 | 65 | 1,151,633 | 1.00 | 4.04 | -1.395 |  | 2.863 | -0.487 | 0.626 |  |  |  |  |  |
| vamorolone | 0 | 0 | 0 | 0 | 5 | 23,955 | 79 | 1,151,619 | 1.00 | 3.33 | -1.202 |  | 2.863 | -0.420 | 0.675 |  |  |  |  |  |
| deflazacort | 0 | 0 | 0 | 0 | 12 | 23,948 | 181 | 1,151,517 | 1.00 | 3.31 | -1.197 |  | 2.844 | -0.421 | 0.674 |  |  |  |  |  |
| abacavir dolutegravir lamivudine | 0 | 0 | 0 | 0 | 20 | 23,940 | 300 | 1,151,398 | 1.00 | 3.28 | -1.188 |  | 2.838 | -0.419 | 0.675 |  |  |  |  |  |
| dexmethylphenidate | 0 | 0 | 0 | 0 | 6 | 23,954 | 95 | 1,151,603 | 1.00 | 3.27 | -1.185 |  | 2.857 | -0.415 | 0.678 |  |  |  |  |  |
| enzalutamide | 0 | 0 | 0 | 0 | 178 | 23,782 | 2,947 | 1,148,751 | 1.00 | 2.93 | -1.073 |  | 2.829 | -0.379 | 0.704 |  |  |  |  |  |
| efavirenz | 0 | 0 | 0 | 0 | 5 | 23,955 | 95 | 1,151,603 | 1.00 | 2.77 | -1.018 |  | 2.862 | -0.356 | 0.722 |  |  |  |  |  |
| desmopressin | 0 | 0 | 0 | 0 | 8 | 23,952 | 160 | 1,151,538 | 1.00 | 2.55 | -0.935 |  | 2.850 | -0.328 | 0.743 |  |  |  |  |  |
| buprenorphine naloxone | 0 | 0 | 0 | 0 | 6 | 23,954 | 143 | 1,151,555 | 1.00 | 2.18 | -0.778 |  | 2.857 | -0.272 | 0.785 |  |  |  |  |  |
| acetaminophen tramadol | 0 | 0 | 0 | 0 | 7 | 23,953 | 167 | 1,151,531 | 1.00 | 2.15 | -0.767 |  | 2.853 | -0.269 | 0.788 |  |  |  |  |  |
| cobicistat elvitegravir emtricitabine tenofovir | 0 | 0 | 0 | 0 | 8 | 23,952 | 190 | 1,151,508 | 1.00 | 2.15 | -0.763 |  | 2.850 | -0.268 | 0.789 |  |  |  |  |  |
| emtricitabine rilpivirine tenofovir alafenamide | 0 | 0 | 0 | 0 | 6 | 23,954 | 181 | 1,151,517 | 1.00 | 1.72 | -0.543 |  | 2.856 | -0.190 | 0.849 |  |  |  |  |  |
| eplerenone | 0 | 0 | 0 | 0 | 6 | 23,954 | 185 | 1,151,513 | 1.00 | 1.68 | -0.521 |  | 2.856 | -0.183 | 0.855 |  |  |  |  |  |
| tesamorelin | 0 | 0 | 0 | 0 | 5 | 23,955 | 159 | 1,151,539 | 1.00 | 1.66 | -0.505 |  | 2.861 | -0.177 | 0.860 |  |  |  |  |  |
| darolutamide | 0 | 0 | 0 | 0 | 17 | 23,943 | 508 | 1,151,190 | 1.00 | 1.65 | -0.504 |  | 2.839 | -0.177 | 0.859 |  |  |  |  |  |
| memantine | 0 | 0 | 0 | 0 | 5 | 23,955 | 161 | 1,151,537 | 1.00 | 1.64 | -0.493 |  | 2.861 | -0.172 | 0.863 |  |  |  |  |  |
| clobazam | 0 | 0 | 0 | 0 | 8 | 23,952 | 252 | 1,151,446 | 1.00 | 1.62 | -0.481 |  | 2.850 | -0.169 | 0.866 |  |  |  |  |  |
| abiraterone | 0 | 0 | 0 | 0 | 116 | 23,844 | 3,489 | 1,148,209 | 1.00 | 1.61 | -0.475 |  | 2.830 | -0.168 | 0.867 |  |  |  |  |  |
| dextromethorphan | 0 | 0 | 0 | 0 | 6 | 23,954 | 194 | 1,151,504 | 1.00 | 1.61 | -0.474 |  | 2.856 | -0.166 | 0.868 |  |  |  |  |  |
| tamsulosin | 0 | 0 | 0 | 0 | 32 | 23,928 | 1,058 | 1,150,640 | 1.00 | 1.48 | -0.390 |  | 2.834 | -0.137 | 0.891 |  |  |  |  |  |
| emtricitabine tenofovir | 0 | 0 | 0 | 0 | 10 | 23,950 | 355 | 1,151,343 | 1.00 | 1.42 | -0.351 |  | 2.846 | -0.123 | 0.902 |  |  |  |  |  |
| ritonavir | 0 | 0 | 0 | 0 | 5 | 23,955 | 193 | 1,151,505 | 1.00 | 1.37 | -0.312 |  | 2.861 | -0.109 | 0.913 |  |  |  |  |  |
| apalutamide | 0 | 0 | 0 | 0 | 83 | 23,877 | 3,005 | 1,148,693 | 1.00 | 1.34 | -0.290 |  | 2.831 | -0.102 | 0.918 |  |  |  |  |  |
| cabotegravir | 0 | 0 | 0 | 0 | 6 | 23,954 | 234 | 1,151,464 | 1.00 | 1.33 | -0.287 |  | 2.856 | -0.100 | 0.920 |  |  |  |  |  |
| elotuzumab | 0 | 0 | 0 | 0 | 5 | 23,955 | 200 | 1,151,498 | 1.00 | 1.32 | -0.277 |  | 2.861 | -0.097 | 0.923 |  |  |  |  |  |
| cobicistat darunavir emtricitabine tenofovir alafenamide | 0 | 0 | 0 | 0 | 7 | 23,953 | 274 | 1,151,424 | 1.00 | 1.31 | -0.273 |  | 2.853 | -0.096 | 0.924 |  |  |  |  |  |
| idursulfase | 0 | 0 | 0 | 0 | 32 | 23,928 | 1,215 | 1,150,483 | 1.00 | 1.29 | -0.251 |  | 2.834 | -0.089 | 0.929 |  |  |  |  |  |
| pralsetinib | 0 | 0 | 0 | 0 | 7 | 23,953 | 280 | 1,151,418 | 1.00 | 1.29 | -0.251 |  | 2.853 | -0.088 | 0.930 |  |  |  |  |  |
| ranolazine | 0 | 0 | 0 | 0 | 8 | 23,952 | 333 | 1,151,365 | 1.00 | 1.23 | -0.203 |  | 2.850 | -0.071 | 0.943 |  |  |  |  |  |
| cabotegravir rilpivirine | 0 | 0 | 0 | 0 | 6 | 23,954 | 255 | 1,151,443 | 1.00 | 1.22 | -0.201 |  | 2.856 | -0.070 | 0.944 |  |  |  |  |  |
| rabeprazole | 0 | 0 | 0 | 0 | 6 | 23,954 | 255 | 1,151,443 | 1.00 | 1.22 | -0.201 |  | 2.856 | -0.070 | 0.944 |  |  |  |  |  |
| emtricitabine tenofovir alafenamide | 0 | 0 | 0 | 0 | 6 | 23,954 | 257 | 1,151,441 | 1.00 | 1.21 | -0.193 |  | 2.856 | -0.068 | 0.946 |  |  |  |  |  |
| capmatinib | 0 | 0 | 0 | 0 | 7 | 23,953 | 303 | 1,151,395 | 1.00 | 1.19 | -0.172 |  | 2.852 | -0.060 | 0.952 |  |  |  |  |  |
| methadone | 0 | 0 | 0 | 0 | 5 | 23,955 | 227 | 1,151,471 | 1.00 | 1.16 | -0.150 |  | 2.861 | -0.052 | 0.958 |  |  |  |  |  |
| dutasteride | 0 | 0 | 0 | 0 | 7 | 23,953 | 317 | 1,151,381 | 1.00 | 1.14 | -0.127 |  | 2.852 | -0.045 | 0.964 |  |  |  |  |  |
| dolutegravir | 0 | 0 | 0 | 0 | 6 | 23,954 | 286 | 1,151,412 | 1.00 | 1.09 | -0.087 |  | 2.856 | -0.030 | 0.976 |  |  |  |  |  |
| testosterone | 0 | 0 | 0 | 0 | 8 | 23,952 | 376 | 1,151,322 | 1.00 | 1.09 | -0.082 |  | 2.850 | -0.029 | 0.977 |  |  |  |  |  |
| edaravone | 0 | 0 | 0 | 0 | 5 | 23,955 | 254 | 1,151,444 | 1.00 | 1.04 | -0.038 |  | 2.861 | -0.013 | 0.989 |  |  |  |  |  |
| cobicistat elvitegravir emtricitabine tenofovir disoproxil | 0 | 0 | 0 | 0 | 11 | 23,949 | 548 | 1,151,150 | 1.00 | 1.01 | -0.008 |  | 2.844 | -0.003 | 0.998 |  |  |  |  |  |
| rilpivirine | 0 | 0 | 0 | 0 | 10 | 23,950 | 507 | 1,151,191 | 1.00 | 0.99 | 0.006 |  | 2.846 | 0.002 | 0.998 |  |  |  |  |  |
| deucravacitinib | 0 | 0 | 0 | 0 | 6 | 23,954 | 319 | 1,151,379 | 1.00 | 0.98 | 0.022 |  | 2.856 | 0.008 | 0.994 |  |  |  |  |  |
| cobicistat elvitegravir emtricitabine tenofovir alafenamide | 0 | 0 | 0 | 0 | 5 | 23,955 | 276 | 1,151,422 | 1.00 | 0.96 | 0.045 |  | 2.861 | 0.016 | 0.987 |  |  |  |  |  |
| pegvisomant | 0 | 0 | 0 | 0 | 6 | 23,954 | 352 | 1,151,346 | 1.00 | 0.89 | 0.121 |  | 2.856 | 0.042 | 0.966 |  |  |  |  |  |
| onasemnogene abeparvovec xioi | 0 | 0 | 0 | 0 | 6 | 23,954 | 409 | 1,151,289 | 1.00 | 0.76 | 0.271 |  | 2.856 | 0.095 | 0.924 |  |  |  |  |  |
| bicalutamide | 0 | 0 | 0 | 0 | 5 | 23,955 | 361 | 1,151,337 | 1.00 | 0.73 | 0.313 |  | 2.861 | 0.109 | 0.913 |  |  |  |  |  |
| dapagliflozin propanediol metformin | 0 | 0 | 0 | 0 | 5 | 23,955 | 395 | 1,151,303 | 1.00 | 0.67 | 0.403 |  | 2.861 | 0.141 | 0.888 |  |  |  |  |  |
| brentuximab vedotin | 0 | 0 | 0 | 0 | 13 | 23,947 | 1,077 | 1,150,621 | 1.00 | 0.60 | 0.508 |  | 2.842 | 0.179 | 0.858 |  |  |  |  |  |
| emtricitabine rilpivirine tenofovir disoproxil | 0 | 0 | 0 | 0 | 5 | 23,955 | 508 | 1,151,190 | 1.00 | 0.52 | 0.654 |  | 2.861 | 0.229 | 0.819 |  |  |  |  |  |
| tafamidis meglumine | 0 | 0 | 0 | 0 | 8 | 23,952 | 828 | 1,150,870 | 1.00 | 0.49 | 0.707 |  | 2.849 | 0.248 | 0.804 |  |  |  |  |  |
| panitumumab | 0 | 0 | 0 | 0 | 6 | 23,954 | 749 | 1,150,949 | 1.00 | 0.42 | 0.875 |  | 2.856 | 0.307 | 0.759 |  |  |  |  |  |
| patiromer | 0 | 0 | 0 | 0 | 6 | 23,954 | 756 | 1,150,942 | 1.00 | 0.41 | 0.885 |  | 2.856 | 0.310 | 0.757 |  |  |  |  |  |
| linezolid | 0 | 0 | 0 | 0 | 9 | 23,951 | 1,194 | 1,150,504 | 1.00 | 0.38 | 0.962 |  | 2.847 | 0.338 | 0.735 |  |  |  |  |  |
| tisagenlecleucel | 0 | 0 | 0 | 0 | 6 | 23,954 | 923 | 1,150,775 | 1.00 | 0.34 | 1.084 |  | 2.856 | 0.380 | 0.704 |  |  |  |  |  |
| ipilimumab | 0 | 0 | 0 | 0 | 10 | 23,950 | 1,610 | 1,150,088 | 1.00 | 0.31 | 1.161 |  | 2.845 | 0.408 | 0.683 |  |  |  |  |  |
| canagliflozin | 0 | 0 | 0 | 0 | 7 | 23,953 | 1,291 | 1,150,407 | 1.00 | 0.28 | 1.277 |  | 2.852 | 0.448 | 0.654 |  |  |  |  |  |
| emicizumab kxwh | 0 | 0 | 0 | 0 | 8 | 23,952 | 1,490 | 1,150,208 | 1.00 | 0.27 | 1.295 |  | 2.849 | 0.455 | 0.649 |  |  |  |  |  |
| oxaliplatin | 0 | 0 | 0 | 0 | 8 | 23,952 | 2,012 | 1,149,686 | 1.00 | 0.20 | 1.596 |  | 2.849 | 0.560 | 0.575 |  |  |  |  |  |

Notes: Compact view of Table S4a for reporting; ordering by BD_FDR (ascending).
