# Supplementary material for: Drug-associated insomnia and sex-specific disproportionality in the FDA adverse event reporting system (2019–Q1 2025)
Source: Front Pharmacol. 2026 Feb 23;17:1758403. doi: 10.3389/fphar.2026.1758403 (PMC12968212; doi:10.3389/fphar.2026.1758403)
Supplement: Supplementary file 2 [file DataSheet1.docx]

## Supplementary Figures

## ****Figure S1. Disproportionate reporting signals for insomnia in females identified by ANY-suspect analysis (Top 30).****


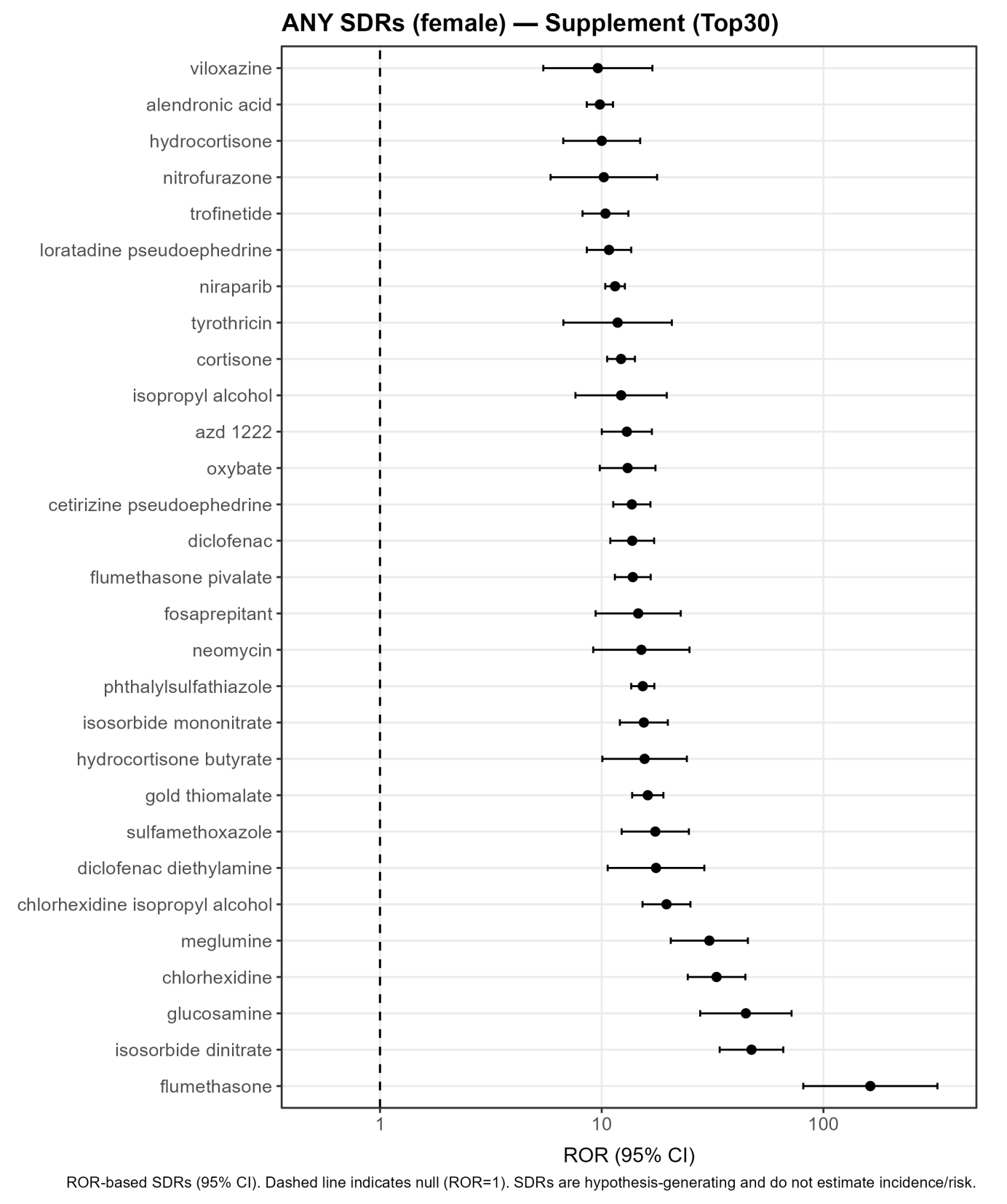


**Legend:** Forest plot of the top 30 parent systemic drugs with the highest RORs for insomnia among female reports using the ANY-suspect definition, which includes drugs recorded as either primary or secondary suspects (PS + SS). Points represent RORs and horizontal bars indicate 95% CIs. The dashed vertical line denotes ROR = 1.

## ****Figure S2. Disproportionate reporting signals for insomnia in males identified by ANY-suspect analysis (Top 30).****


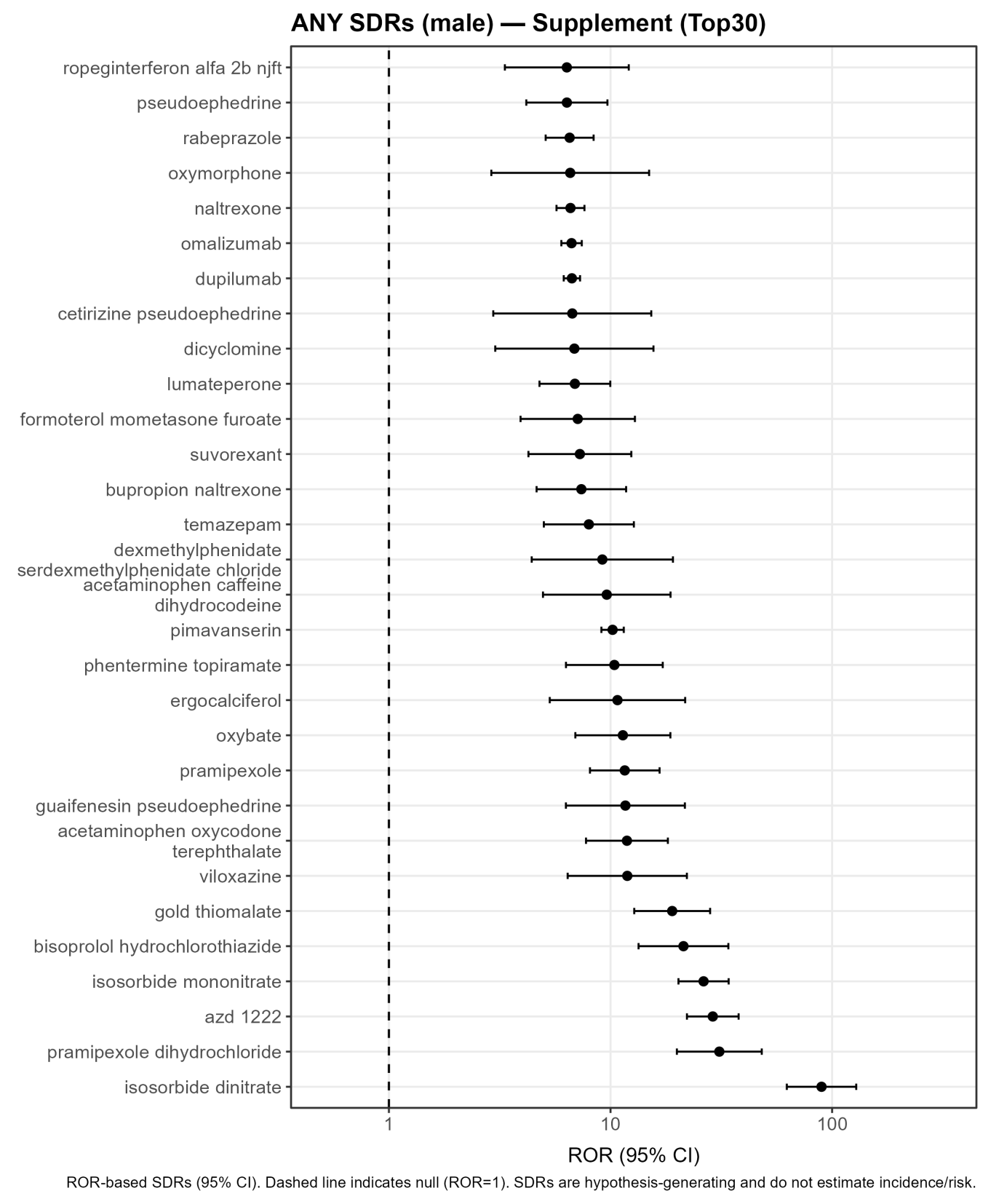


**Legend:** Forest plot of the top 30 parent systemic drugs with the highest RORs for insomnia among male reports using the ANY-suspect definition (PS + SS). RORs and 95% CIs are shown on a logarithmic scale, with the dashed vertical line indicating the null value (ROR = 1).

## ****Figure S3. Overall disproportionate reporting signals for insomnia identified by ANY-suspect analysis (Top 30).****


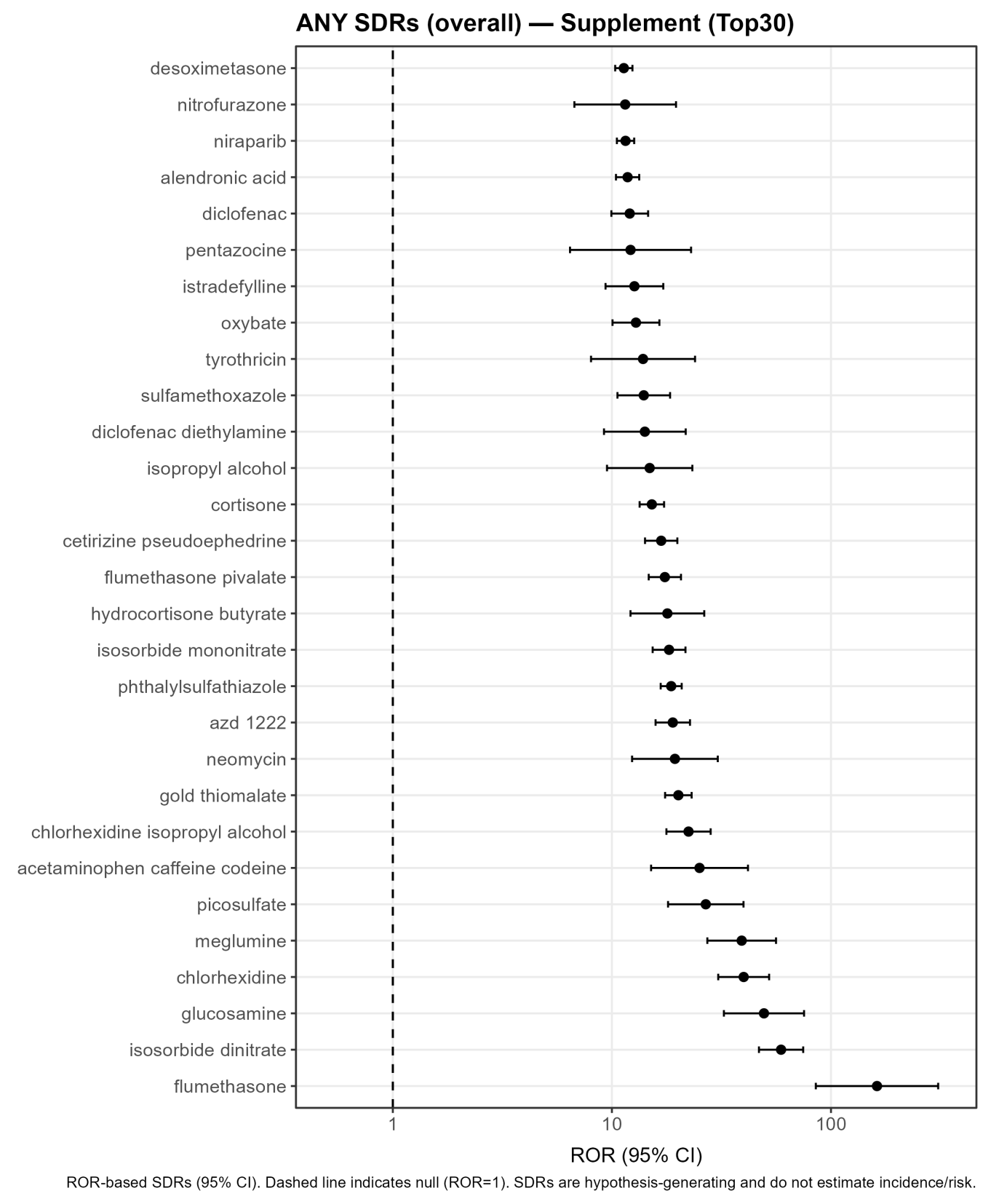


**Legend:** Forest plot of the top 30 parent systemic drugs with the highest RORs for insomnia in the overall FAERS population using the ANY-suspect definition (PS + SS). This analysis illustrates the broader, lower-specificity signal landscape when secondary-suspect drugs are included.

## ****Figure S4. Disproportionate reporting signals for insomnia in females identified by PS analysis (Top 30).****


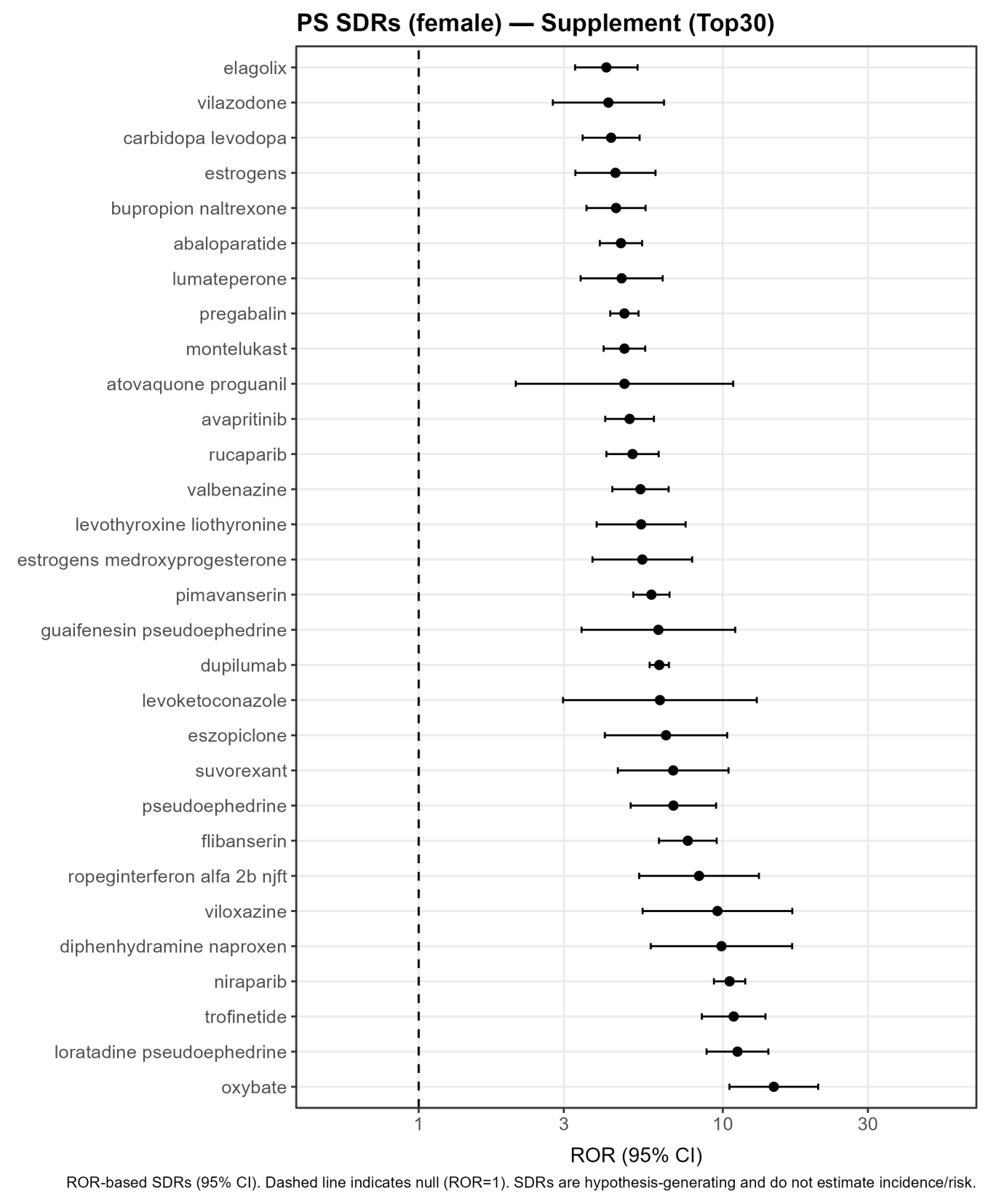


**Legend:** Forest plot of the top 30 parent systemic drugs with insomnia SDRs among female reports restricted to primary-suspect (PS) drugs. RORs and 95% CIs are displayed on a logarithmic scale, with the dashed vertical line denoting ROR = 1.

## ****Figure S5. Disproportionate reporting signals for insomnia in males identified by PS analysis (Top 30).****


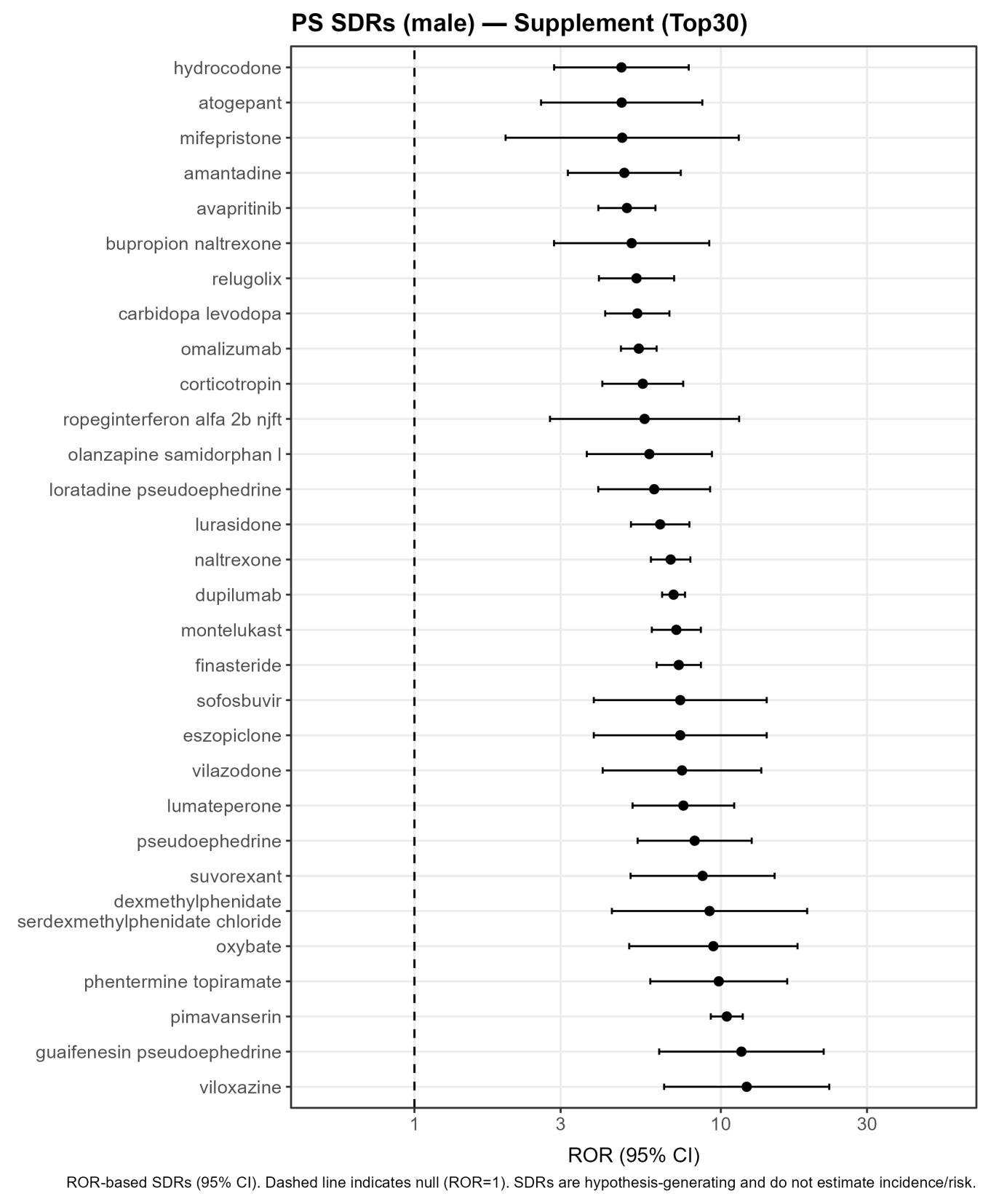


**Legend:** Forest plot of the top 30 parent systemic drugs with insomnia SDRs among male reports using PS-restricted analysis. RORs and 95% CIs are shown. This figure supports the robustness of the main sex-stratified findings when extending the threshold beyond the top 15 drugs.

**Figure S6. Overall disproportionate reporting signals for insomnia identified by PS analysis (Top 30).**

**
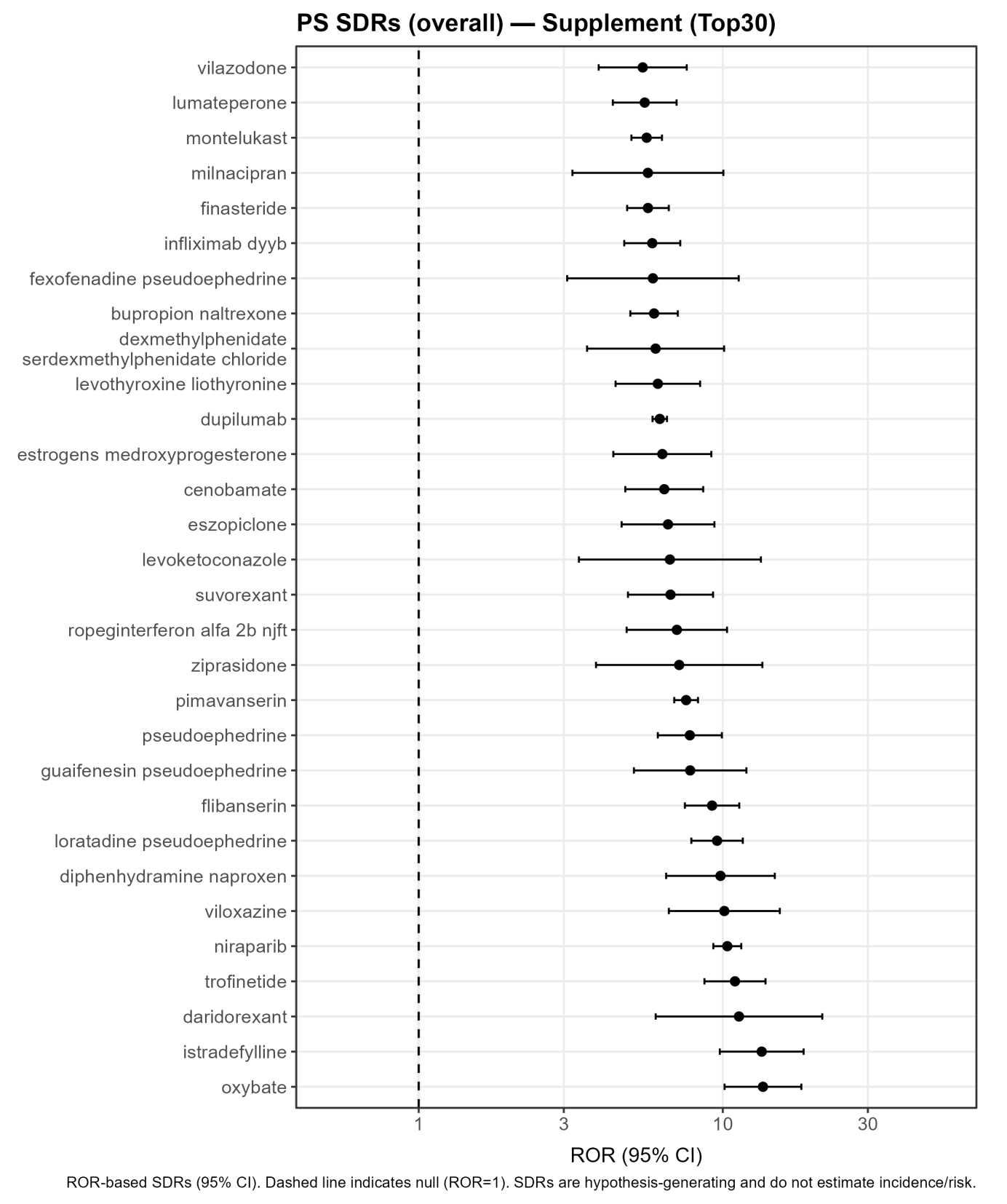
**

**Legend:** Forest plot of the top 30 parent systemic drugs with the highest RORs for insomnia in the overall FAERS population using PS-restricted analysis. Together with Figures S4 and S5, this figure demonstrates the stability of the signal structure when the ranking threshold is expanded.

**Figure S7. Volcano plot of sex heterogeneity for drug–insomnia signals based on FAERS data**

**
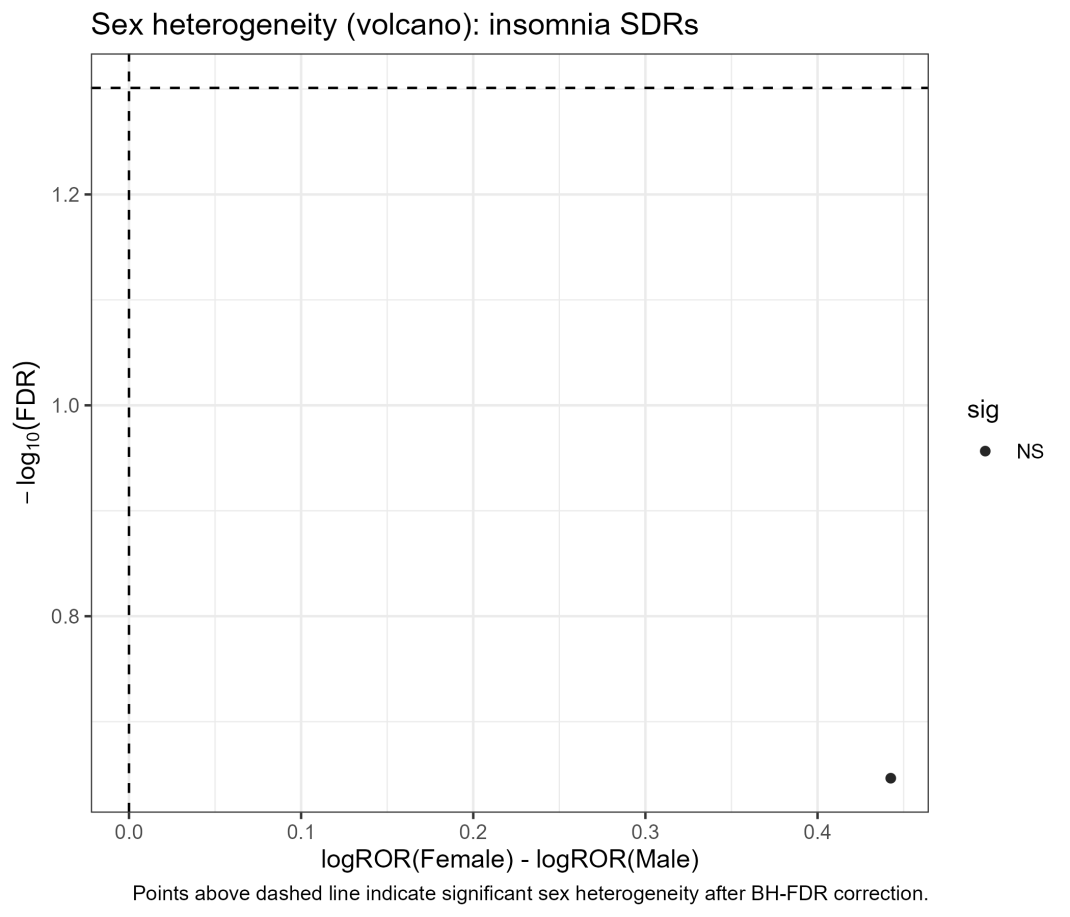
**

**Legend:** Volcano plot showing formal tests of sex heterogeneity for drug–insomnia signals identified in FAERS.
The x-axis represents the difference between sex-specific log reporting odds ratios (logRORfemale_\mathrm{female}female​ − logRORmale_\mathrm{male}male​), and the y-axis represents −log10_{10}10​ of the false discovery rate (FDR)–adjusted p-value derived from formal heterogeneity testing (Breslow–Day or equivalent interaction test).

The horizontal dashed line indicates the significance threshold after Benjamini–Hochberg FDR correction, and the vertical dashed line denotes no difference between sexes. Only a limited number of drug–event pairs met the criteria for formal heterogeneity testing, and few signals remained statistically significant after correction for multiple comparisons, reflecting limited statistical power for interaction testing in spontaneous reporting data. These results should be interpreted as exploratory and hypothesis-generating rather than confirmatory evidence of sex-specific risk differences.
